# Transcriptomic and proteomic analysis of pre-diapause and non-diapause eggs of migratory locust, *Locusta migratoria* L. (Orthoptera: Acridoidea)

**DOI:** 10.1038/srep11402

**Published:** 2015-06-19

**Authors:** Xiongbing Tu, Jie Wang, Kun Hao, Douglas W. Whitman, Yaoli Fan, Guangchun Cao, Zehua Zhang

**Affiliations:** 1 State Key Laboratory for Biology of Plant Diseases and Insect Pests, Institute of Plant Protection, Chinese Academy of Agricultural Sciences, Beijing, 100193, P.R. China; 2 School of Biological Sciences, Illinois State University, Normal, Illinois, 61761, USA

## Abstract

Low temperature induces diapause in locusts. However, the physiological processes and initiation mechanism of diapause are not well understood. To understand the molecular basis of diapause, ‘omics’ analyses were performed to examine the differences between diapause and non-diapause eggs at both transcriptional and translational levels. Results indicated that a total of 62,241 mRNAs and 212 proteins were differentially expressed. Among them, 116 transcripts had concurrent transcription and translation profiles. Up-regulated genes related to diapause included glutathiones-S-transferase *et al.*, and down-regulated genes including juvenile hormone esterase-like protein *et al.* KEGG analysis mapped 7,243 and 99 differentially expressed genes and proteins, to 83 and 25 pathways, respectively. Correlation enriched pathways indicated that there were nine identical pathways related to diapause. Gene Ontology analysis placed these genes and proteins into three categories, and a higher proportion of genes related to metabolism was up-regulated than down-regulated. Furthermore, three up-regulated pathways were linked to cryoprotection. This study demonstrates the applicability of high-throughput omics tools to identify molecules linked to diapause in the locust. In addition, it reveals cellular metabolism in diapause eggs is more active than in non-diapause eggs, and up-regulated enzymes may play roles in cryoprotection and storing energy for diapause and post-diapause stages.

Diapause is an adaptation to seasonality that is widespread across invertebrate taxa^1^,^2^. There are two forms of insect diapause: facultative diapause, which is a response to key stimuli from the environment, andobligatory diapause, which is a fixed component of ontogeny^1^. The migratory locust is an embryonic diapause insect of the facultative diapause type^3^,^4^. These pests can move and settle in new habitats, which may be a fixed behavioral strategy that results from long-term evolutionary adaptation^5^. Diapause may serve to adapt the migratory locust to factors such as temperature, photoperiod and latitude^6^,^7^. For example, the diapause rate of the migratory locust in high latitude locations is higher than that observed in low latitude locations^8^, and short photoperiod and low temperatures are known to increase the number of locusts entering diapause^6^,^7^,^9^. More importantly, incubation temperature influences the diapause rate of locust eggs; the proportion of embryo reversal is strengthened upon temperature increase^10^,^11^. In addition, we identified an interesting phenomenon in Tianjin, a location that has supported two generations of migratory locusts, *locusta migratoria* L., according to historical records. Most hatchlings of the 3^rd^ generation emerged in autumn due to the overwintering of eggs, but this phenomenon decreased dramatically after A.D. 1999^12^,^13^ and may have been linked to temperature variation during autumn, as high temperatures have been shown to promote egg development and to result in the emergence of 3^rd^-generation hatchlings. However, the influence of temperature on locust diapause is unknown.

Locust eggs are extremely sensitive to temperature before anatrepsis, which leads parts of the embryo to hatch upon exposure to high temperatures during this period^6^,^7^,^14^. When degree-day (DD) was higher than 150 heat units prior to anatrepsis, all of the eggs transformed into hatchlings. When DD was only 66 heat units, 64.3% of embryos entered diapause^11^. In this study, we reared locusts at high and low temperatures until adults oviposited, then overwintered eggs were collected when DD reached 50 heat units^9^,^11^. Thus, diapause eggs were exposed to low temperatures, and non-diapause eggs were maintained at high temperatures. We compared diapause and non-diapause eggs at the transcriptome (RNA-seq) and proteome (iTRAQ) levels^15^,^16^ to enhance our understanding of the genetic and molecular mechanisms underlying diapause in an agriculturally important insect pest.

## Results

### Omics analyses

Diapause and non-diapause locust egg transcriptomes were sequenced individually, which generated approximately 135–138 million clean reads, 12.2–12.4 billion nucleotides, 210–223 thousand contigs, and 94–118 thousand unigenes for each library (Table 1). To uncover the molecular events underlying these transcriptomic profiles, we aligned unigene sequences to protein databases, including NR, Swiss-Prot, KEGG and COG (e-value < 0.00001) by blastx, and nucleotide database NT (e-value < 0.00001) by blastn, retrieving proteins with the highest sequence similarity to the given unigenes along with their functional annotations. Of the 100,490 unigenes, we found that 36,765 were annotated.

The total number of sequences identified by mass spectrometry of locust egg proteomes was 271,268, which represented 2,787 peptide spectra and 2,634 distinct peptides (Table 2). Of the 2,634 peptides identified, more than 40% (1,078) were assigned to a putative protein by homology search against the non-redundant (NR) database, leaving approximately 60% (1,556) of the peptides unidentified. Among these annotated proteins, 447 were hypothetical, putative, or predicted.

### Differentially expressed genes (DEGs) between diapause and non-diapause locust eggs

**Figure 1:**
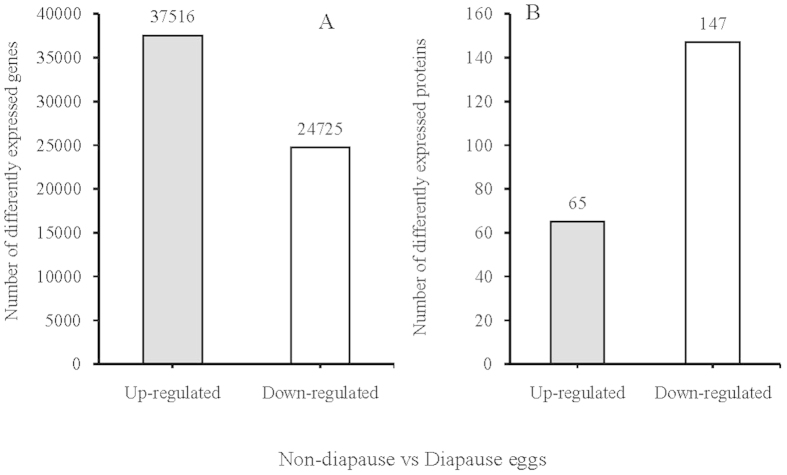
Statistics of Differentially Expressed Genes and Proteins. (**A**)The distribution of differentially expressed genes (DEGs). (**B**) The distribution of differentially expressed proteins. The X-axis indicates control-vs-treat, and the Y-axis indicates the number of the DEGs or proteins. The red bar indicates up-regulated genes or proteins, and the green bar indicates down-regulated genes or proteins.

**Figure 2:**
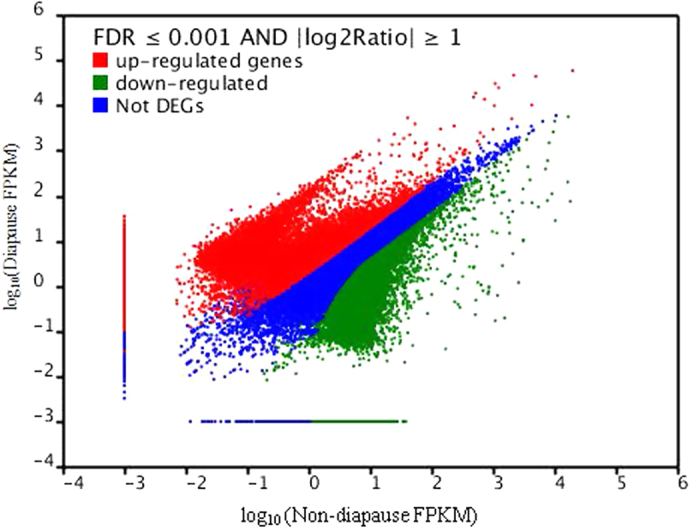
Expression levels in diapause vs non-diapause locust eggs. Genes were divided among three classes:red genes are up-regulated in the right sample vs. the left sample, green genes are down-regulated in the right samplevs. the left sample, and blue genes are not differentially expressed. The horizontal coordinate indicatesexpression level in non-diapause eggs, while the vertical coordinates indicate expression level in diapause eggs.

Following temperature treatment, a total of 37,516 and 24,725 up- and down-regulated transcripts, respectively, were differentially expressed (FDR ≤ 0.001 and |log_2_Ratio ≥ 1) between diapause and non-diapause locust eggs (Fig. 1A). Most of these transcripts (15,896 and 16054, 66%), however, were expressed within a 2- to 10-fold difference (Fig. 2).

**Figure 3:**
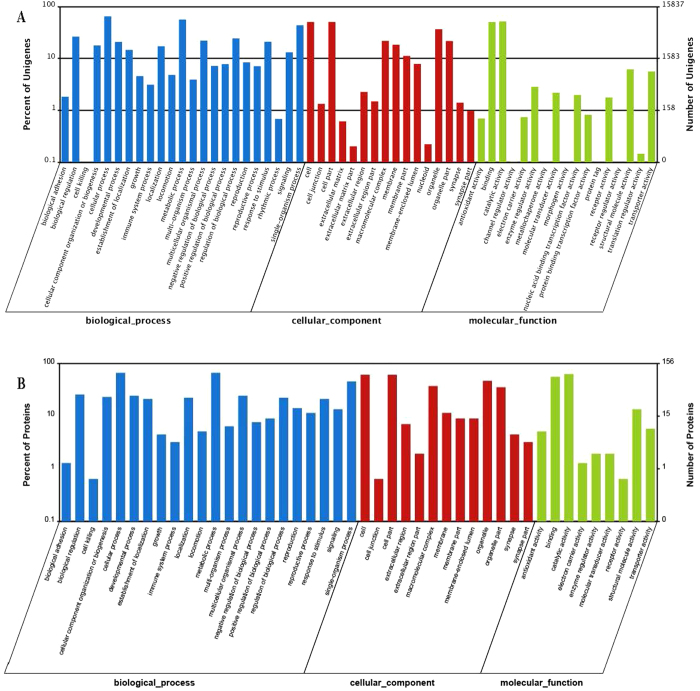
Gene Ontological classification of differentially expressed genes and proteins between Non-diapause and Diapause locust eggs. The differentially expressed genes or proteins are grouped into three hierarchically structured GO terms, biological process, cellular component, and molecular function. The y-axis indicates the number of genes or proteins in each GO term. (**A**) Differentially expressed genes identified by RNA-seq. (**B**) Differentially expressed proteins identified by iTRAQ.

Table S1 shows the GO classification of 62,241 transcripts that were differentially expressed between diapause and non-diapause locust eggs (≥2-fold change, FDR ≤ 0.001). With Blast2GO, 9,696 differentially expressed transcripts were assigned to 56 GO classes (Fig. 3A). The majority of these genes were assigned to categories such as biological process, cellular component, molecular function, response to biological regulation, cellular process, metabolic process, single-organism process, binding, catalytic activity, organelle part, cell, and cell part (Table S1). In the up-regulated group, most genes were assigned to the mucin, heat shock protein, ATP-binding cassette, transcription initiation factor, and DNA-directed RNA polymerase categories. To investigate their biological functions, 10,351 differentially expressed genes were mapped to 259 pathways in the KEGG database. To investigate which biological pathways were active when exposed to diapause treatment, 7,243 differently expressed genes were assigned to reference pathways in KEGG. As a result, 83 pathways were substantially enriched (p-value < 0.05), including “Metabolic Pathway” and “Ribosome” (Table 3). Specifically, 154 genes encoding enzymes involved in starch and sucrose metabolism pathways were highly enriched, including trehalose, 6-phosphate phosphatase, trehalase, glucokinase and starch phosphorylase (Table S2). Interestingly, we also found that 2,459 up-regulated and 365 down-regulated genes were linked to metabolic pathways (Table S3). The up-regulated transcripts included glutathiones-S-transferase (*GST*), UDP-glucuronosyl transferase (*UGT*), transforming growth factor-β-receptor (*TGF*), insulin-like growth factor receptor (*IGF*), nuclear receptor, fork head transcription factor (*FOXO*), transient receptor potential cation channel subfamily A member 1(*TRPA1*), inositol-3-phosphate synthase (*PIP3*), acetyl-Coenzyme A acyltransferase (*ACAA*), catalase, glucose dehydrogenase, heat shock proteins (*HSPs*), glycerol kinase, and the cytochrome c oxidase, cytochrome p450 (Table S2, S3). The down-regulated genes included juvenile hormone esterase-like protein, hemocyanin subunit, hexamerin-like protein, and NADH-dehydrogenase (Table S2, S3).

### Temperature-dependent protein expression in diapause and non-diapause locust eggs

After temperature challenge, 212 differentially expressed proteins (p-value ≤ 0.05) were identified between diapause and non-diapause locust eggs. Among them, 65 proteins were up-regulated (≥1.2-fold, p-value ≤ 0.05), and 147 were down-regulated (≤0.8-fold, p-value ≤ 0.05) (Fig. 1B). Following in-gel digestion by trypsin, the peptides were identified by liquid chromatography-electrospray ionization multi stage mass spectrometry (LC-ESI-MS/MS; Table S4). Cu/Zn superoxide dismutase and peroxiredoxin-1, which are found in the peroxisome, were up-regulated by 3.233- and 1.393-fold, respectively, in diapause eggs relative to non-diapause eggs^17^. Other up-regulated peptides in diapause eggs included juvenile hormone esterase (11.211-fold), which demethylates the insect juvenile hormones JH (1) and JH (3) but does not hydrolyze the analogous ethyl or isopropyl esters^18^; (1,3)-β-glucan synthase (2.801-fold), which plays an important role in catalyzing the transfer of sugar moieties from activated donor molecules to specific acceptor molecules to form glycosidic bonds^19^; eukaryotic translation initiation factor 4A (1.936- fold), which is associated with protein translation initiation and elongation^20^; and lipase (1.795-fold), which performs essential roles in the digestion, transport and processing of dietary lipids^21^. In addition, proteins related to energy regulation, protein transport and metabolism were differentially expressed between diapause and non-diapause locust eggs (Table S5). Peptides that were down-regulated in the diapause eggs included hemocyanin subunit type 2 (−7.143-fold), which is involved in energy storage, osmotic pressure maintenance and molt regulation^22^; fatty acid-binding protein (−4.255-fold), which may play an important role in up-regulating heat shock proteins during diapause^23^; phenoloxidase subunit 1 (−3.300-fold), which performs essential roles in the synthesis of hemocyanin C, M, N and tyrosinase^24^; and alcohol dehydrogenase class-3-like (−1.212-fold),which is associated with aromatic compound and fatty acid degradation, glycolysis, gluconeogenesis, and tyrosine, retinol and xenobiotic metabolism^25^.

**Figure 4:**
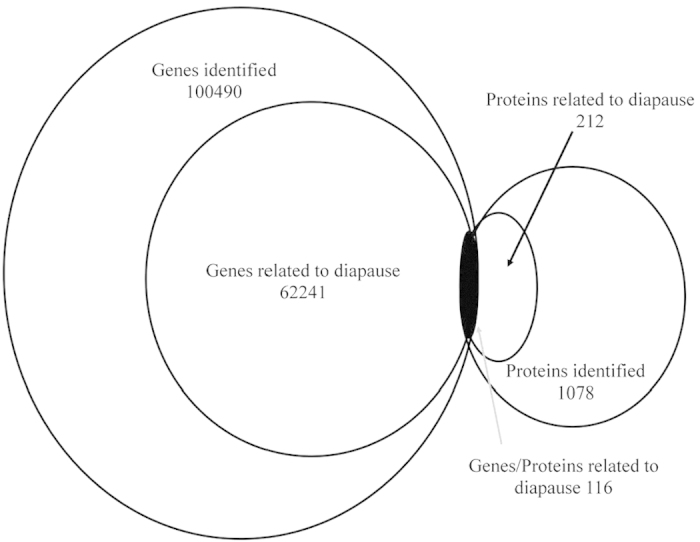
Correlation between differently expressed proteins and genes. The numerical value in each circle represents the quantity of genes or proteins, including identified genes and proteins and genes or proteins related to diapause, respectively, and genes/proteins related to diapause together.

**Figure 5:**
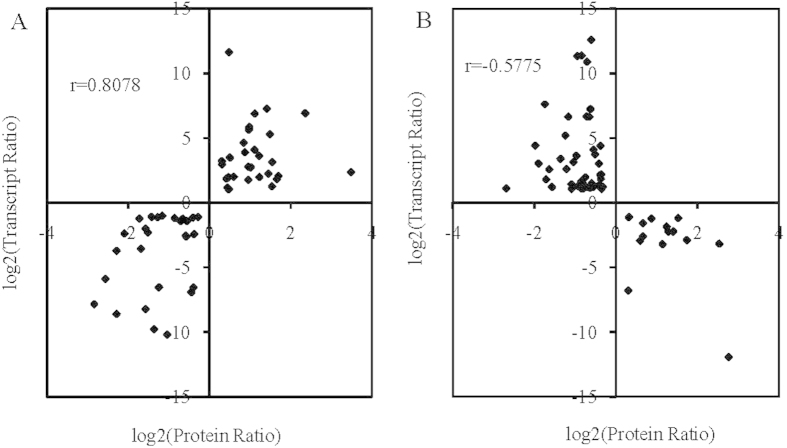
Variation intrends between differentially expressed mRNA and protein from the same locus. (**A**) represents differentially expressed mRNA and protein with the same trend, while (**B**) represents differentially expressed mRNA and protein with opposite trends.Scatter plots illustrate the distribution of differentially expressed proteins and related genes. The Pearson correlation coefficient between protein and mRNA expression profiles is shown in the upper left corner of the plot.

To correlate protein and mRNA expression profiles, accession numbers from the proteomic dataset were extracted and compared with the annotated RNA-seq libraries. Correlation between the differentially expressed proteins and genes showed that there were only 116 genes/proteins related to diapause (Fig. 4). Tables S6 and S7 show the correlation between mRNA and protein, and the correlation coefficients between the protein and gene expression profiles were 0.8078 and −0.5775 (Fig. 5, Table S6, S7).

### Gene ontology and pathway analysis

Among the 212 differentially expressed proteins, 156 were subcategorized into 44 hierarchically structured GO classes, including 22 Biological Process, 13 Cellular Component, and 9 Molecular Function (Fig. 3B) categories. Specifically, “cellular process” (104, 66.7%), “metabolic process” (104, 66.7%), and “single-organism process” (71, 45.5%) were highly represented in “Biological Process”, whereas “cell” (95, 60.9%), “cell part” (95, 60.9%), and “organelle” (73, 46.8%) were the most common categories in “Cellular Component”. In “Molecular Function”, the top three categories were “catalytic activity” (98, 62.8%), “binding” (87, 55.8%) and “structural molecule activity” (21, 13.5%) (Fig. 3B, Table S6). Ninety-nine differentially expressed proteins were allocated to the reference pathways in KEGG (Table 4). As a result, 25 pathways were enriched (p-value ≤ 0.05, Table 4). Correlation of the enriched pathways for differentially expressed genes and proteins showed that there were 9 identical pathways related to diapause, including those mediating, peroxisome, glyoxylate and dicarboxylate metabolism, melanogenesis, tyrosine metabolism, riboflavin metabolism, retinol metabolism, pentose and glucuronate interconversion, and glutathione metabolism (Tables 3,4).

We investigated which biological pathways were active following diapause treatment and found that 3 pathways played an important role, including (i) Starch and sucrose metabolism (KEGG: Map00500), which involved α, α’-D-Trehalose synthesis from glycogen and included the up-regulation of maltase-glucoamylase, glycogen debranching enzyme, hexokinase, starch phosphorylase, glycogen synthase, glucose-6-phosphate isomerase, phosphoglucomutase, UTP-glucose-1-phosphate uridylyltransferase, trehalose 6-phosphate synthase, and trehalose 6-phosphate phosphatase; (ii) Glycolysis and gluconeogenesis (KEGG: Map00010); and (iii) Glycerolipid metabolism (KEGG: Map00561), which involves glycerol synthesis from glucose and the up-regulation of aldose-1-epimerase, hexokinase, glucokinase, ADP-dependent glucokinase, phosphoglucomutase, glucose-6-phosphate 1-epimerase, glucose-6-phosphate isomerase, 6-phosphofructokinase, triosephosphate isomerase, glycerate kinase, aldehyde dehydrogenase, alcohol dehydrogenase, and aldehyde reductase during the pre-diapause stage. These up-regulated enzymes enabled diapause eggs to synthesize cryoprotectants (*e.g.*, trehalose and glycerinum) and to store energy for diapause and post-diapause stages.

## Discussion

Insect diapause can be divided into the pre-diapause, diapause, and post-diapause stages^26^, and insects in the pre-diapause stage are able to synthesize cryoprotectants and store energy for later developmental stages^27^,^28^. However, the definition and naming of the pre-diapause stage are unsettled and occasionally ambiguous in the literature, particularly for *Locusta migratoria* L^9^. The induction factors in obligative diapause insects and facultative diapause insects (*e.g.*, *L. migratoria*) were analyzed, and a comparison of the results suggested that the entrance of the latter to diapause was determined by the photoperiod and incubation temperature^29^. It is a fixed component of ontogeny, *e.g.*, *Sarcophaga crassipalpis* is pupal diapause^30^, whereas *Drosophila montana* is adult reproductive diapause^31^. Overall, 100% of them entered diapause and remained in the stage until stimuli triggered further development in the spring^1^. However, for *L. migratoria*, photoperiod only influenced the development of ovary^7^, which was not the most important factor influencing the diapause rate of locust eggs. Therefore temperature plays a determinative role, and the effects of temperature can be subdivided as follows: first, low temperature influenced the development of adults;for example, if the temperature was lower than 21 °C^32^, females could not support the reproductive process, and the fertilization process in adults may be affected^9^. Second, incubation temperature influenced the development of eggs during the anatrepsis stage^11^. Hence, we speculated that the pre-diapause stage of *L. migratoria* began at the point of female fertilization and ended at embryo anatrepsis. This theory could explain why only parts of eggs would enter diapause and why the diapause rate varied from northern to southern China, as incubation temperature differed during the processes of fertilization and embryo anatrepsis^8^,^11^. Thus, overwintered eggs treated at 50 DD, which coincided with the pre-diapause stage of the migratory locust, were collected and prepared for omics analyses.

Transcriptome sequencing indicated that physiological metabolism had been activated, and up-regulated transcripts (37,516) were more abundant than down-regulated transcripts (24,725) (Fig. 2). These results can be used to study the mechanisms underlying diapause in *L. migratoria*^33^. A correlation analysis of differentially expressed proteins and genes showed that a subset ofgenes and proteins were expressed with the same trend during the pre-diapause stage (Fig. 5, Tables S6, S7). Genes including glutathiones-S-transferase, UDP-glucuronosyl transferase, and transforming growth factor-β-receptor were up-regulated, whereas hemocyanin subunit, hexamerin-like protein, and NADH dehydrogenase were among the genes down-regulated at both the transcriptional and translational levels (Table S2, S3). Physiologically similar phenotypes were identified in *Sarcophag acrassipalpis*, *Drosophila melanogaster* and *Caenorhabditis elegans* transcriptomes^34^. However, we found that some genes, including juvenile hormone esterase-like protein, were up-regulated at the translational level but down-regulated at the transcriptional level. This effect can be attributed to differences in expression time^34^. Changes in KEGG pathways and enzymes suggest that all enzymes in the three pathways related to cryoprotection were up-regulated, in contrast to the data obtained from *Bombyx mori*, although the silkworm utilizes the same cryoprotectants^28^. For example, sorbitol dehydrogenase expression was inhibited and glycogen phosphorylase A was enhanced for *B. mori*. However, we did not detect sorbitol dehydrogenase gene expression in locust eggs^35^,^36^. Diapause eggs produced trehalose and glycerinum, as supported by data from Li *et al.*^37^, but we detected no sorbitol dehydrogenase^38^,^39^. These differences may be due to differences in geographical sampling and diapause stage^40^,^41^.

Insect diapause-related endocrine regulation includes diapause hormone and insulin pathways. For example, diapause hormone and G-couple receptor in the ovary combine to synthesize cryoprotectants in *B. mori*^28^,^42^,^43^,^44^, and the insulin signaling system promotes *Drosophila melanogaster* diapause via FOXO phosphorylation in the PI3K/Akt pathway^45^, whereas juvenile hormone synthesis promoted *Culex pipiens* diapause^46^. We discovered that insulin growth factor and insulin receptor were up-regulated in the migratory locust, which enhances the proportion of juvenile hormone. This may pertain to diapause^47^, but not via the same mechanism as in the mosquito, *Culex pipiens*^45^,^48^. Studies have reported that many temperature-related genes, including fork head transcription factor, heat shock protein, and transient receptor potential cation channel subfamily A member 1 might play a role in insect diapause metabolism^46^,^49^,^50^. We found that these genes were up-regulated (Figs 2,3), but how they affect migratory locust diapause is unknown. Additionally, genes and proteins expressed during the diapause and post-diapause stages require further study to understand the mechanism of locust diapause.

## Materials & Methods

### Ethics statement

The migratory locust, *Locusta migratoria* L. strains used in this study were initially collected in the field at Tianjin in 2007 and have since been maintained in a greenhouse at the Institute of Plant Protection, Chinese Academy of Agricultural Sciences. Species of the genus Acridoidea are common agricultural pests and are not included in the “List of Protected Animals in China”. No specific permits were required for the described field studies.

### Temperature treatment

To obtain diapause and non-diapause eggs, we raised the oriental migratory locust in growth cabinets (PRX-350B-30) under low and high temperature regimes^6^. We recorded the daily temperature at Tianjian, from 16 July to 6 November 2012, as a reference (reference value), then used two different cabinets to mimic natural the natural daily temperature cycles of (reference value+1) for the high temperature regime, and (reference value-1) for low temperature regime^11^. The photoperiod regime used in the experiment was L:D = 12:12, and the relative humidity (RH) was kept at ~60% for eggs and ~80% for nymph and adults. We recorded 24-h temperature data on each day for each growth cabinet using a HOBO Pro v2 logger, and the results showed that the DD of the high temperature regime was 840 heat units and that DD of the low temperature regime was 678 heat units^32^.

### Sample preparation

More than 2,000 locust eggs were collected from the same generation, divided into two groups and reared in the two growth cabinets for a whole generation^11^. After adults oviposited, we recorded the hatchlings on each day in each of the two growth cabinets. We found that the diapause rate of overwintering eggs at the low temperature regime was 64.3% (diapause eggs, only 35.7% hatchlings emergence in the growth cabinet), whereas all eggs hatched at the high temperature regime (non-diapause eggs, 100% hatchling emergence in the growth cabinet)^11^. Diapause and non-diapause eggs were collected when DD reached 50 heat units^9^,^11^ and were immediately snap-frozen in liquid nitrogen and stored at −80 °C.

### RNA-seq library preparation and sequencing

Total RNA was isolated from diapause and non-diapause locust egg samples, respectively, with Trizol (Invitrogen) according to the manufacturer’s protocol. The quantity and quality of RNA were determined with a Nanodrop ND-1000. To remove residual DNA contamination, total RNA was treated with RNase-free DNase I (New England BioLabs). mRNA was purified from 6 μg of total RNA from each sample with Dynaloligo (dT) beads (Invitrogen) and was then fragmented using an RNA fragmentation kit (Ambion). The first cDNA strand was synthesized using random hexamer primers. The double-stranded cDNA fragments were processed by end repair using T4 DNA polymerase, Klenow DNA polymerase, and T4 polynucleotide kinase (NEB), followed by a single adenine base addition using Klenow 39 to 59 exo-polymerase, and was concluded by ligation with Illumina’s adaptor. The products were purified using a QiaQuick PCR extraction kit (QiaGen) and enriched by PCR amplification. Finally, the library products were subjected to sequencing analysis on the Illumina HiSeq^TM^ 2500 platform.

### Annotation and de novo gene expression

Raw reads were transformed into clean reads by removing the adaptor sequences, reads with unknown nucleotides larger than 5%, empty sequences (sequences with an adaptor but no reads), and low-quality sequences (the rate of reads for which quality value ≤ 10 is more than 20%)^51^. Transcriptome de novo assembly was carried out with the Trinity short read assembly program. Trinity combines three independent software modules: Inchworm, Chrysalis, and Butterfly, which are applied sequentially to process large volumes of RNA-seq reads^52^. Trinity partitions the sequence data into many individual de Bruijn graphs, each representing the transcriptional complexity at a given gene or locus, and then processes each graph independently to extract full-length splicing isoforms and to tease apart transcripts derived from paralogous genes. The calculation of Unigene expression uses the FPKM (RPKM) method (Fragments Per kb per Million reads)^53^. The formulas for computing FPKM and RPKM are the same. The only difference between them is the method used to compute the parameters of N and C. If both pairs of reads were aligned to a gene, we treated them as 1 fragment with FPKM but as two reads with RPKM. Both algorithms are rational. For functional annotation, distinct sequences were searched via BLAST against the NCBI NR database with a cut-off E-value of 10^−5^. In addition, Blast2GO (http://www.blast2go.org) was used to assign Gene Ontology terms (http://www.geneontology.org), while the Kyoto Encyclopedia of Genes and Genomes (KEGG, http://www.genome.jp/kegg/ or http://www.kegg.jp/), a database resource that integrates genomic, chemical, and systemic functional information, was adopted to annotate molecular networks (pathways).

### Screening for differentially expressed genes

Referring to “The significance of digital gene expression profiles”, which has been cited hundreds of times^54^, we developed a rigorous algorithm to identify differentially expressed genes between two samples. The null hypothesis and alternative hypothesis to identify differentially expressed genes are defined as follows:

*H*_0_:a gene has the same expression level in two samples

*H*_1_:a gene has different expression levels in two samples

We denote x as the number of fragments that uniquely map to gene A. For each transcript representing a small fraction of the library, p(x) follows the Poisson distribution closely.


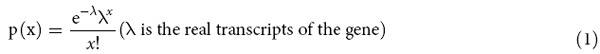


The total fragment number of sample 1 is N1, and the total fragment number of sample 2 is N2; gene A comprises x fragments in sample 1 and y fragments in sample 2. The probability of gene A being expressed equally between two samples can be calculated using the following formula:


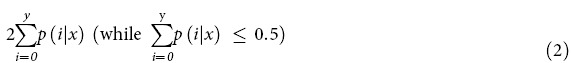


Or


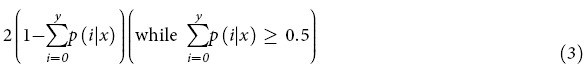


Here


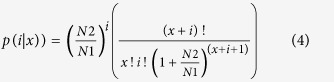


When we perform thousands of hypothesis tests, a suitable P-value for an individual test is not sufficient to guarantee a low rate of false discovery; thus, we must perform a multiple testing correction for each individual hypothesis test to guarantee a low false discovery rate overall. FDR (False Discovery Rate) control is a statistical method used in multiple hypothesis testing to correct for p-value. In practical terms, the FDR is the expected false discovery rate; for example, if 1000 observations were experimentally predicted to differ and the maximum FDR for these observations was 0.1, then 100 of these observations would be expected to be false discoveries (refer to Benjamini (2001) for details). When we have an FDR, we use the ratio of FPKMs of the two samples concurrently. The smaller the FDR is and the larger the ratio is, the larger the difference of the expression level between the two samples will be. In our analysis, we chose those samples with FDR ≤ 0.001 and a ratio larger than 2 DEGs and then carried out GO functional analysis and KEGG Pathway analysis^55^.

### Protein quantification and database search using iTRAQ labeling

Locust egg samples were dissected in lysis buffer (7 M urea, 2 Mthiourea, 4% CHAPS, 40 mMTris-HCl, pH 8.5), and 1 mM PMSF (phenyl methane sulfonyl fluoride) and 2 mM EDTA (ethylene diamine tetraacetic acid) were added after dissection. After 5 min, 10 mM DTT was added to the lysis solution, which was then centrifuged at 4 °C, 30,000 × *g* for 15 min. The supernatant was collected, and the concentration of total proteins was determined using a 2DQuantification Kit (GE Healthcare)^56^. For quality check, 30 mg of total protein from each sample was subjected to SDS-PAGE analysis. After, 100 ml protein from each sample was digested with trypsin gold (Promega) (protein: trypsin = 30:1) at 37 °C for 16 h, and the resultant peptides were dried by vacuum centrifugation. The peptides were reconstituted in 0.5 M TEAB and processed according to the manufacturer’s protocol for 8-plex iTRAQ (Applied Biosystems, Inc)^57^. Samples (100 mg total protein/sample) from non-diapause and diapause locust eggs were labeled with iTRAQ tags 115 and 119, respectively. Then, pooled mixtures of iTRAQ-labeled peptides were fractionated by SCX chromatography (Phenomenex, Inc, USA) using a Shimadzu LC-20AB HPLC Pump system. Collected fractions were pooled into 10 final fractions and analyzed by nano LC-MS/MS analysis after desalting by Strata XC18 column (Phenomenex) and vacuum dried. Nano LC-MS/MS analysis of each of these fractions was performed using a LTQ-OrbitrapVelos mass spectrometer (Thermo Fisher Scientific Inc. Rockford, IL, USA) equipped with nano electrospray ionization^58^,^59^. Peptides were identified by searching against a specified database containing 41,407 mRNA sequences using a MS/MS data interpretation algorithm within Mascot. A peptide mass tolerance of 2 ppm and fragment mass tolerance of 0.02 Da were allowed. When the Mascot software was used to search the database, 1,005 proteins were identified, with a false discovery rate (FDR) of less than 1%. Differential expression ratios for proteins were obtained from Mascot software (http://www.matrixscience.com), which calculates protein ratios using only ratios from the spectra that are distinct for each protein and excluding the shared peptides of protein isoforms. To calculate differential expression ratios, all identified spectra from a protein were used to obtain an average protein ratio relative to the control label (i.e., fold change). Student’s t-test was used to analyze the differential expression of proteins between diapause and non-diapause locust eggs. The P-value was calculated using the confidence intervals from the error factor generated in Mascot as


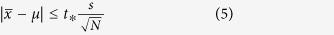


where N is the number of peptide ratios, s is the standard deviation, and x represents the mean of the peptide ratios. In this study, we used P ≤0.05 and fold change >1.2 or <0.8 as the thresholds to judge the significance level of differentiated protein expression^60^.

### GO Classification of Differentially Expressed RNA and Proteins and Pathway Analysis

Functional annotation of transcripts and proteins identified in locust egg samples was carried out using Blast2GO, an integrated GO annotation and data mining tool that assigns gene ontology through BLAST searches against nucleotide and/or protein databases^61^. GO enrichment analysis provides all GO terms that are significantly enriched for differentially expressed genes and proteins in comparison to locust eggs. This method first maps all differentially expressed genes and proteins to GO terms in the database (http://www.geneontology.org/), calculates gene numbers for every term, then uses a hypergeometric test to find significantly enriched GO terms for differentially expressed genes and proteins compared to a locust egg transcriptome/proteome background. The formula used in this calculation is


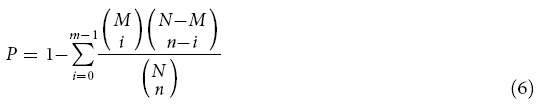


where N is the number of all genes or proteins with a GO annotation; n is the number of differentially expressed genes or proteins in N; M is the number of all genes or proteins that are notated to the given GO term; and m is the number of differentially expressed genes or proteins in M. The calculated P-value was subjected to a Bonferroni Correction, taking a corrected P-value of 0.05 as a threshold. GO terms fulfilling this condition were defined as significantly enriched for differentially expressed genes or proteins. All identified transcripts and proteins were mapped to a pathway in the Kyoto Encyclopedia of Genes and Genomes (KEGG) database^62^. Significantly enriched metabolic pathways or signal transduction pathways containing differentially expressed genes and proteins were identified using the same formula as in GO analysis. Here N represents the number of all genes or proteins with KEGG annotation, n is the number of differentially expressed genes or proteins in N, M is the number of all genes or proteins annotated to specific pathways, and m is number of differentially expressed genes or proteins in M.

### Correlation between protein and mRNA expression

To assess the correlation between transcriptomic and proteomic platforms, we first designated cutoff values to select subsets of genes and proteins with distinctive expression signals. All protein sequences identified by iTRAQ were analyzed and loaded into a searchable database. For each protein, we queried the RNA-seq data for expression patterns of matching transcripts (P-value < 0.05). The significance level of the overlap between detected proteins and transcripts was determined using Pearson’s Chi-squared test with Yates’ continuity correction^63^.

## Additional Information

**How to cite this article**: Tu, X. *et al.* Transcriptomic and proteomic analysis of pre-diapause and non-diapause eggs of migratory locust, *Locusta migratoria* L. (Orthoptera: Acridoidea). *Sci. Rep.* **5**, 11402; doi: 10.1038/srep11402 (2015).

**Accession codes**: Locust eggs transcriptome datasets are available at NCBI project PRJNA271501 with accession number SRP051668, and SRA with accession number SRR1738245, SRR1738246. Locust eggs proteome datasets are available at Peptide Atlas under a submission number PASS00694.

## Supplementary Material

Supplementary Information

## Figures and Tables

**Table 1:** Summary of RNA-seq metrics from locust eggs transcriptomes.

**Metric**	**Non-diapause**	**Diapause**
Clean Reads	135,882,798	138,727,698
Nucleotides (nt)	12,229,451,820	12,485,492,820
Contig	210,692	223,501
Unigene	94,118	118,651
Total Unigene	100,490	
Annotation	36,765	

**Table 2:** Summary of iTRAQ metrics form locust eggs proteomes.

Total spectra	271,628
Unique spectra	8,614
Matched protein	1,078
Differentially expressed protein	212

**Table 3:** Significantly enriched KEGG pathways in transcriptome.

	**Pathway**	**DEGs genes with pathway annotation (Percent, %)**	**All genes with pathway annotation (Percent, %)**	**P-value**
1	Vibrio cholerae infection	721 (5.14%)	887 (3.9%)	4.32E-38
2	Metabolic pathways	2825 (20.13%)	4089 (17.96%)	6.11E-28
3	Amoebiasis	650 (4.63%)	856 (3.76%)	1.14E-19
4	Ribosome	670 (4.77%)	923 (4.05%)	6.61E-13
5	Peroxisome	305 (2.17%)	400 (1.76%)	2.81E-10
6	Glyoxylate and dicarboxylate metabolism	164 (1.17%)	201 (0.88%)	6.65E-10
7	Fatty acid metabolism	198 (1.41%)	249 (1.09%)	8.99E-10
8	Proteasome	203 (1.45%)	258 (1.13%)	2.95E-09
9	Salivary secretion	280 (1.99%)	370 (1.63%)	5.70E-09
10	Prion diseases	159 (1.13%)	198 (0.87%)	1.11E-08
11	Synaptic vesicle cycle	175 (1.25%)	224 (0.98%)	9.04E-08
12	Collecting duct acid secretion	94 (0.67%)	111 (0.49%)	9.05E-08
13	Valine, leucine and isoleucine degradation	216 (1.54%)	283 (1.24%)	9.45E-08
14	Rheumatoid arthritis	113 (0.81%)	137 (0.6%)	9.48E-08
15	Oxidative phosphorylation	352 (2.51%)	485 (2.13%)	2.09E-07
16	Shigellosis	267 (1.9%)	360 (1.58%)	2.97E-07
17	Alanine, aspartate and glutamate metabolism	164 (1.17%)	211 (0.93%)	4.18E-07
18	Antigen processing and presentation	133 (0.95%)	167 (0.73%)	4.28E-07
19	PPAR signaling pathway	191 (1.36%)	250 (1.1%)	4.54E-07
20	Epithelial cell signaling in Helicobacter pylori infection	156 (1.11%)	200 (0.88%)	5.34E-07
21	Bacterial invasion of epithelial cells	282 (2.01%)	386 (1.7%)	1.31E-06
22	Fructose and mannose metabolism	142 (1.01%)	183 (0.8%)	2.90E-06
23	Glycolysis/Gluconeogenesis	227 (1.62%)	307 (1.35%)	3.26E-06
24	Phagosome	305 (2.17%)	423 (1.86%)	3.39E-06
25	Renin-angiotensin system	43 (0.31%)	47 (0.21%)	4.10E-06
26	Pathogenic Escherichia coli infection	315 (2.24%)	441 (1.94%)	8.89E-06
27	Butanoate metabolism	101 (0.72%)	127 (0.56%)	1.15E-05
28	Propanoate metabolism	149 (1.06%)	198 (0.87%)	3.32E-05
29	Melanogenesis	162 (1.15%)	219 (0.96%)	7.59E-05
30	Arginine and proline metabolism	202 (1.44%)	279 (1.23%)	9.67E-05
31	Pyruvate metabolism	214 (1.52%)	297 (1.3%)	9.90E-05
32	Parkinson’s disease	331 (2.36%)	474 (2.08%)	0.000105848
33	Tyrosine metabolism	140 (1%)	188 (0.83%)	0.000134091
34	Glycine, serine and threonine metabolism	127 (0.9%)	169 (0.74%)	0.000136795
35	Citrate cycle (TCA cycle)	204 (1.45%)	284 (1.25%)	0.000189872
36	Tryptophan metabolism	137 (0.98%)	185 (0.81%)	0.000241699
37	beta-Alanine metabolism	127 (0.9%)	171 (0.75%)	0.000326199
38	Synthesis and degradation of ketone bodies	37 (0.26%)	43 (0.19%)	0.000428008
39	Legionellosis	156 (1.11%)	215 (0.94%)	0.000485139
40	Riboflavin metabolism	36 (0.26%)	42 (0.18%)	0.000602506
41	GABAergic synapse	125 (0.89%)	171 (0.75%)	0.001053885
42	Dopaminergic synapse	179 (1.28%)	252 (1.11%)	0.0010958
43	MAPK signaling pathway	308 (2.19%)	449 (1.97%)	0.001181529
44	Tuberculosis	242 (1.72%)	348 (1.53%)	0.001212063
45	Adipocytokine signaling pathway	103 (0.73%)	139 (0.61%)	0.001311472
46	Measles	160 (1.14%)	224 (0.98%)	0.001326528
47	Influenza A	327 (2.33%)	479 (2.1%)	0.001386636
48	Epstein-Barr virus infection	515 (3.67%)	772 (3.39%)	0.001735867
49	Wnt signaling pathway	228 (1.62%)	329 (1.45%)	0.002178141
50	Starch and sucrose metabolism	154 (1.1%)	217 (0.95%)	0.002486196
51	Alzheimer’s disease	354 (2.52%)	526 (2.31%)	0.003783211
52	Amino sugar and nucleotide sugar metabolism	156 (1.11%)	223 (0.98%)	0.005776157
53	Amphetamine addiction	99 (0.71%)	137 (0.6%)	0.005909079
54	Pertussis	76 (0.54%)	103 (0.45%)	0.006425784
55	Pentose phosphate pathway	108 (0.77%)	151 (0.66%)	0.006995646
56	Adherens junction	274 (1.95%)	406 (1.78%)	0.008035088
57	Phototransduction - fly	82 (0.58%)	113 (0.5%)	0.009757659
58	Toxoplasmosis	169 (1.2%)	245 (1.08%)	0.009908632
59	HTLV-I infection	351 (2.5%)	528 (2.32%)	0.01145809
60	Insulin signaling pathway	241 (1.72%)	358 (1.57%)	0.01451352
61	Primary bile acid biosynthesis	40 (0.28%)	52 (0.23%)	0.0146295
62	Endocrine and other factor-regulated calcium reabsorption	89 (0.63%)	125 (0.55%)	0.01622075
63	Huntington’s disease	589 (4.2%)	905 (3.98%)	0.01628533
64	Osteoclast differentiation	77 (0.55%)	107 (0.47%)	0.01652479
65	Retinol metabolism	100 (0.71%)	142 (0.62%)	0.01807061
66	Glycerolipid metabolism	162 (1.15%)	237 (1.04%)	0.0185793
67	Pentose and glucuronate interconversions	147 (1.05%)	214 (0.94%)	0.01893007
68	Alcoholism	169 (1.2%)	248 (1.09%)	0.01942268
69	Biosynthesis of unsaturated fatty acids	80 (0.57%)	112 (0.49%)	0.01948457
70	Protein processing in endoplasmic reticulum	430 (3.06%)	656 (2.88%)	0.02020514
71	Pancreatic secretion	192 (1.37%)	284 (1.25%)	0.02119797
72	Valine, leucine and isoleucine biosynthesis	34 (0.24%)	44 (0.19%)	0.02127704
73	alpha-Linolenic acid metabolism	67 (0.48%)	93 (0.41%)	0.02344569
74	Phenylalanine metabolism	72 (0.51%)	101 (0.44%)	0.02755099
75	Endocytosis	363 (2.59%)	554 (2.43%)	0.03150932
76	Vitamin B6 metabolism	16 (0.11%)	19 (0.08%)	0.03185256
77	Glutathione metabolism	126 (0.9%)	184 (0.81%)	0.03210199
78	Arachidonic acid metabolism	71 (0.51%)	100 (0.44%)	0.03246063
79	Retrograde endocannabinoid signaling	94 (0.67%)	135 (0.59%)	0.03279646
80	Fc gamma R-mediated phagocytosis	313 (2.23%)	476 (2.09%)	0.03438166
81	Nicotine addiction	34 (0.24%)	45 (0.2%)	0.03585109
82	Terpenoid backbone biosynthesis	58 (0.41%)	81 (0.36%)	0.03984046
83	Galactose metabolism	99 (0.71%)	144 (0.63%)	0.04608303

**Table 4:** Significantly enriched KEGG pathways in proteome.

	**Pathway**	**Diff Proteins with pathway annotation (Percent, %)**	**All Proteins with pathway annotation (Percent, %)**	**P-value**
1	Metabolism of xenobiotics by cytochrome P450	10 (5.41%)	15 (1.69%)	0.000141533
2	Metabolic pathways	60 (32.43%)	201 (22.66%)	0.000365654
3	Riboflavin metabolism	11 (5.95%)	20 (2.25%)	0.000706352
4	Drug metabolism - cytochrome P450	8 (4.32%)	12 (1.35%)	0.000714683
5	Melanogenesis	13 (7.03%)	26 (2.93%)	0.000759894
6	Tyrosine metabolism	12 (6.49%)	25 (2.82%)	0.001936295
7	Peroxisome	10 (5.41%)	19 (2.14%)	0.001966556
8	Prostate cancer	7 (3.78%)	12 (1.35%)	0.004713203
9	Other glycan degradation	4 (2.16%)	5 (0.56%)	0.007708014
10	Lysosome	12 (6.49%)	30 (3.38%)	0.0118856
11	Drug metabolism - other enzymes	5 (2.7%)	8 (0.9%)	0.01213747
12	Alcoholism	6 (3.24%)	11 (1.24%)	0.0137955
13	Retinol metabolism	4 (2.16%)	6 (0.68%)	0.0193596
14	Ascorbate and aldarate metabolism	4 (2.16%)	6 (0.68%)	0.0193596
15	Glyoxylate and dicarboxylate metabolism	5 (2.7%)	9 (1.01%)	0.02273707
16	Glutathione metabolism	8 (4.32%)	19 (2.14%)	0.02806231
17	Aldosterone-regulated sodium reabsorption	3 (1.62%)	4 (0.45%)	0.03029416
18	Steroid hormone biosynthesis	3 (1.62%)	4 (0.45%)	0.03029416
19	Pentose and glucuronate interconversions	5 (2.7%)	10 (1.13%)	0.03790488
20	Regulation of actin cytoskeleton	8 (4.32%)	20 (2.25%)	0.03861341
21	Mineral absorption	2 (1.08%)	2 (0.23%)	0.04331439
22	Acute myeloid leukemia	2 (1.08%)	2 (0.23%)	0.04331439
23	Endometrial cancer	2 (1.08%)	2 (0.23%)	0.04331439
24	Carbohydrate digestion and absorption	2 (1.08%)	2 (0.23%)	0.04331439
25	Salmonella infection	7 (3.78%)	17 (1.92%)	0.04466544
