# Supplementary material for: Transcriptomic and proteomic analysis of pre-diapause and non-diapause eggs of migratory locust, Locusta migratoria L. (Orthoptera: Acridoidea)
Source: Sci Rep. 2015 Jun 19;5:11402. doi: 10.1038/srep11402 (PMC4650673; doi:10.1038/srep11402)
Supplement: Supplementary Information [file srep11402-s1.pdf]

Transcriptomic and proteomic analysis of pre-diapause and non-diapause eggs of migratory locust, *Locusta migratoria* L. (Orthoptera: Acridoidea)

Xiongbing Tu[1*], Jie Wang[1*], Kun Hao[1], Douglas W. Whitman[2], Yaoli Fan[1], Guangchun Cao[1] & Zehua Zhang[1**]

**Table S1 Gene ontology analysis of locust eggs transcriptome.**			
Ontology	Class	up number	down number
biological_process	reproduction	1	0
biological_process	locomotion	2	0
biological_process	localization	5	0
biological_process	multi-organism process	5	0
biological_process	immune system process	8	0
biological_process	multicellular organismal process	24	6
biological_process	single-organism process	32	4
biological_process	response to stimulus	42	9
biological_process	developmental process	126	23
biological_process	biological adhesion	143	21
biological_process	establishment of localization	202	33
biological_process	cellular component organization or biogenesis	514	89
biological_process	metabolic process	634	90
biological_process	biological regulation	2071	262
biological_process	cellular process	2953	330
cellular_component	macromolecular complex	2	1
cellular_component	extracellular matrix	0	2
cellular_component	cell junction	0	1
cellular_component	extracellular region	4	9
cellular_component	membrane	138	17
cellular_component	cell	484	62
molecular_function	electron carrier activity	2	0
molecular_function	enzyme regulator activity	3	1
molecular_function	antioxidant activity	5	0
molecular_function	structural molecule activity	5	6
molecular_function	transporter activity	6	2
molecular_function	binding	470	96
molecular_function	catalytic activity	644	107
	total	8525	1171

Table S2 Differentially expressed genes within protective enzymes and starch metabolism pathway in response to diapause.							
Gene	Gene ID	N_FPKM	D_FPKM	log_2Ratio(D/N)	Up-Down-Regulation(D/N)	P-value	FDR
transient receptor potential cation channel (trp)	CL5531.Contig1_All	0	2.4166	11.23876	Up	3.13E-18	1.49E-17
	Unigene69856_All	0	1.4065	10.45789	Up	0.0001153	0.0001809
	Unigene50201_All	0.042	3.0789	6.195882	Up	1.02E-21	5.74E-21
	Unigene23275_All	8.5091	34.075	2.001635	Up	0	0
	Unigene2686_All	5.0869	17.0199	1.742364	Up	4.16E-203	1.82E-201
	Unigene61700_All	0.8295	2.3833	1.522647	Up	0.0001537	0.0002379
	Unigene34550_All	6.2828	0.1829	-5.10228	Down	3.83E-18	1.81E-17
	CL80.Contig9_All	0.4279	0.028	-3.93377	Down	1.39E-07	3.01E-07
	CL80.Contig8_All	0.4538	0.0297	-3.93352	Down	1.39E-07	3.01E-07
	CL80.Contig5_All	0.498	0.0326	-3.9332	Down	1.39E-07	3.01E-07
	CL80.Contig7_All	0.4671	0.0306	-3.93213	Down	1.39E-07	3.01E-07
	Unigene31154_All	8.7658	0.9233	-3.24701	Down	4.45E-30	3.41E-29
	CL80.Contig6_All	0.4107	0.0538	-2.93241	Down	3.54E-06	6.64E-06
	Unigene35532_All	3.7782	0.5095	-2.89055	Down	3.58E-07	7.42E-07
	CL80.Contig4_All	0.6982	0.1281	-2.44637	Down	8.14E-08	1.81E-07
superoxide dismutase（sod）	CL7507.Contig3_All	0	11.2181	13.45354	Up	1.34E-49	1.61E-48
	Unigene58131_All	0	3.6167	11.82046	Up	6.99E-11	2.05E-10
	CL2609.Contig1_All	0	3.4629	11.75777	Up	9.92E-12	3.12E-11
	Unigene60697_All	0	3.3599	11.7142	Up	8.11E-30	6.15E-29
	Unigene60556_All	0	2.8181	11.46051	Up	2.21E-17	1.00E-16
	Unigene59531_All	0	2.7949	11.44858	Up	9.43E-10	2.51E-09
	Unigene61874_All	0	2.7352	11.41743	Up	9.91E-25	6.31E-24
	Unigene68157_All	0	2.5984	11.34341	Up	2.57E-10	7.16E-10
	Unigene73924_All	0	1.7228	10.75054	Up	4.45E-06	8.26E-06
	CL4015.Contig1_All	0	1.6866	10.7199	Up	9.43E-10	2.52E-09

	Unigene73874_All	0	1.2884	10.33136	Up	3.30E-07	6.86E-07
	Unigene79747_All	0	0.9546	9.898753	Up	0.000424	0.000628
	CL7507.Contig1_All	0	0.7642	9.577806	Up	1.34E-10	3.84E-10
	Unigene57027_All	0.0454	10.1998	7.811633	Up	7.11E-68	1.15E-66
	CL1980.Contig2_All	0.0565	10.7771	7.575502	Up	1.73E-57	2.39E-56
	CL1980.Contig1_All	0.1136	15.5674	7.098421	Up	3.44E-81	6.52E-80
	CL7507.Contig2_All	0.0241	3.2972	7.096064	Up	5.89E-41	6.00E-40
	CL235.Contig2_All	0.656	83.4462	6.991007	Up	0	0
	CL4015.Contig2_All	0.043	2.1675	5.655552	Up	8.29E-15	3.22E-14
	Unigene46655_All	0.0864	3.725	5.430065	Up	1.30E-12	4.39E-12
	Unigene58227_All	0.0881	3.6337	5.366153	Up	4.60E-12	1.48E-11
	CL424.Contig1_All	0.1663	5.5403	5.058104	Up	4.31E-27	3.00E-26
	Unigene57126_All	0.7005	9.1938	3.714204	Up	8.45E-46	9.50E-45
	Unigene41478_All	0.9151	5.4927	2.585514	Up	3.75E-21	2.05E-20
	CL235.Contig1_All	1.8617	7.821	2.070732	Up	3.99E-22	2.28E-21
	CL1425.Contig1_All	0.8643	3.5576	2.0413	Up	2.89E-22	1.67E-21
	Unigene2128_All	0.833	2.845	1.77204	Up	2.22E-23	1.34E-22
	CL1425.Contig2_All	60.3604	26.2001	-1.20403	Down	4.90E-232	2.46E-230
catalase（cat）	Unigene47515_All	0	8.3867	13.03389	Up	2.44E-47	2.82E-46
	Unigene49806_All	0	3.7239	11.8626	Up	1.21E-19	6.18E-19
	Unigene47745_All	0	3.0923	11.59446	Up	7.72E-15	3.02E-14
	Unigene64054_All	0	2.4008	11.2293	Up	3.30E-07	6.92E-07
	Unigene69985_All	0	2.3776	11.21529	Up	4.92E-10	1.35E-09
	Unigene40379_All	0	2.2554	11.13917	Up	2.70E-12	8.86E-12
	CL6650.Contig1_All	0	2.2544	11.13853	Up	1.64E-18	7.86E-18
	Unigene41260_All	0	2.1666	11.08122	Up	1.34E-23	8.16E-23
	Unigene46108_All	0	1.9621	10.93818	Up	9.43E-10	2.51E-09
	Unigene40931_All	0	1.806	10.81858	Up	4.92E-10	1.35E-09

	Unigene64133_All	0	1.4758	10.52728	Up	2.32E-06	4.43E-06
	Unigene52062_All	0	1.182	10.20701	Up	6.65E-09	1.63E-08
	Unigene47514_All	0.0414	13.759	8.376529	Up	8.98E-101	2.06E-99
	CL6994.Contig1_All	0.0198	3.6875	7.540999	Up	4.37E-56	5.91E-55
	CL8109.Contig3_All	0.0526	5.4341	6.690835	Up	2.28E-90	4.75E-89
	CL8109.Contig1_All	0.0415	2.7415	6.04571	Up	1.68E-19	8.51E-19
	CL7684.Contig1_All	0.0732	4.7627	6.023792	Up	3.18E-19	1.59E-18
	CL6650.Contig2_All	0.071	3.983	5.809893	Up	2.10E-78	3.86E-77
	Unigene59004_All	0.065	3.1591	5.60293	Up	2.94E-14	1.11E-13
	CL998.Contig1_All	0.1921	6.1642	5.003984	Up	1.07E-17	4.91E-17
	Unigene61099_All	0.0794	2.1835	4.78136	Up	5.49E-08	1.24E-07
	Unigene61757_All	0.0914	2.4731	4.757983	Up	2.30E-28	1.67E-27
	Unigene67010_All	0.0922	1.7762	4.267883	Up	1.40E-05	2.44E-05
	Unigene57001_All	0.6574	11.7542	4.160261	Up	5.71E-59	8.10E-58
	CL9880.Contig1_All	0.2047	3.5039	4.097379	Up	6.17E-13	2.13E-12
	Unigene69078_All	0.1155	1.9598	4.084742	Up	6.88E-09	1.69E-08
	Unigene78100_All	0.0682	1.0637	3.963176	Up	0.00015727	0.000243
	Unigene57753_All	0.6582	5.2674	3.000493	Up	9.08E-17	3.97E-16
	Unigene40796_All	0.3446	2.5958	2.913185	Up	2.71E-19	1.36E-18
pyruvate kinase	Unigene59194_All	0	3.7323	11.86585	Up	6.64E-22	3.76E-21
	Unigene66671_All	0	1.5734	10.61967	Up	2.32E-06	4.40E-06
	Unigene66271_All	0	1.459	10.51076	Up	8.54E-06	1.53E-05
	Unigene76011_All	0	1.3495	10.39821	Up	0.000115	0.00018
	Unigene56337_All	0.0204	4.2984	7.719087	Up	1.16E-63	1.76E-62
	Unigene42902_All	0.0643	6.5729	6.675567	Up	5.80E-60	8.37E-59
	Unigene57754_All	0.1222	3.5851	4.874697	Up	4.46E-16	1.88E-15
	Unigene41523_All	0.081	2.0804	4.682795	Up	1.89E-07	4.03E-07
acetyl	CL4656.Contig1_All	0.0385	3.7077	6.589522	Up	1.14E-28	8.35E-28

cholinesterase	Unigene42250_All	0.1073	4.4097	5.360959	Up	6.79E-56	9.16E-55
	Unigene29534_All	4.6919	0.1132	-5.37323	Down	3.76E-22	2.15E-21
	Unigene26446_All	0.207	0.0136	-3.92795	Down	0.000277	0.000417
	Unigene36353_All	2.5588	0.3754	-2.76897	Down	2.20E-05	3.75E-05
	CL119.Contig3_All	0.959	0.1811	-2.40474	Down	8.73E-16	3.61E-15
	Unigene20205_All	5.8102	1.6158	-1.84634	Down	1.10E-13	3.96E-13
	Unigene15100_All	3.4963	1.3421	-1.38134	Down	0.000379	0.000564
lactate dehydrogenase	Unigene57422_All	0	4.9866	12.28384	Up	8.11E-30	6.16E-29
	CL3345.Contig2_All	0	4.2797	12.06329	Up	2.10E-41	2.15E-40
	Unigene49233_All	0	3.6894	11.84917	Up	7.72E-15	3.02E-14
	CL8352.Contig1_All	0	3.5145	11.7791	Up	4.92E-23	2.93E-22
	Unigene59039_All	0	3.3613	11.7148	Up	8.97E-21	4.82E-20
	Unigene51445_All	0	2.9086	11.50611	Up	1.90E-24	1.20E-23
	Unigene41218_All	0	2.7273	11.41326	Up	9.92E-12	3.10E-11
	Unigene67518_All	0	2.1777	11.08859	Up	3.30E-07	6.90E-07
	Unigene51639_All	0	2.1694	11.08308	Up	6.32E-07	1.28E-06
	CL4049.Contig2_All	0	1.9814	10.9523	Up	2.00E-13	7.09E-13
	Unigene68539_All	0	1.8733	10.87137	Up	4.68E-08	1.06E-07
	Unigene70185_All	0	1.8569	10.85868	Up	1.21E-06	2.37E-06
	Unigene44333_All	0	1.8414	10.84659	Up	6.65E-09	1.64E-08
	Unigene44332_All	0	1.7185	10.74693	Up	1.81E-09	4.68E-09
	Unigene66921_All	0	1.6548	10.69244	Up	6.65E-09	1.64E-08
	Unigene42108_All	0	1.6226	10.66409	Up	3.14E-05	5.27E-05
	CL3124.Contig2_All	0	1.5955	10.63979	Up	1.64E-05	2.83E-05
	CL748.Contig2_All	0	1.4657	10.51737	Up	2.32E-06	4.44E-06
	Unigene63834_All	0	1.3938	10.44481	Up	1.64E-05	2.84E-05
	Unigene77476_All	0	1.3484	10.39703	Up	3.30E-07	6.88E-07
	Unigene70994_All	0	1.2946	10.33829	Up	1.64E-05	2.82E-05

	Unigene77433_All	0	1.1333	10.14631	Up	1.64E-05	2.83E-05
	CL4793.Contig1_All	0.2096	16.4815	6.297065	Up	7.49E-268	4.31E-266
	CL748.Contig1_All	0.0455	2.8601	5.974055	Up	4.51E-36	4.08E-35
	CL3124.Contig1_All	0.1578	8.8282	5.80595	Up	7.13E-109	1.77E-107
	CL8352.Contig2_All	0.123	5.0394	5.356522	Up	6.75E-34	5.76E-33
	Unigene67233_All	0.0503	1.7064	5.084254	Up	6.99E-10	1.88E-09
	CL3345.Contig1_All	0.2147	7.2829	5.084119	Up	6.92E-63	1.04E-61
	CL2687.Contig1_All	0.208	6.815	5.034058	Up	2.90E-94	6.25E-93
	Unigene59673_All	0.0822	2.3358	4.828635	Up	2.95E-08	6.81E-08
	Unigene58691_All	0.1246	3.4268	4.781486	Up	5.82E-22	3.31E-21
	Unigene56402_All	0.42	4.9562	3.560773	Up	7.76E-57	1.06E-55
	CL3163.Contig2_All	0.496	5.1764	3.383537	Up	2.97E-44	3.24E-43
	Unigene48752_All	0.1877	1.3769	2.874923	Up	7.01E-05	0.0001125
	Unigene56772_All	0.3975	2.6032	2.711259	Up	1.55E-08	3.69E-08
	Unigene16304_All	65.8398	145.6507	1.145481	Up	0	0
	CL3734.Contig1_All	0.6121	1.2765	1.060354	Up	1.43E-10	4.07E-10
	Unigene18153_All	12.8391	26.4592	1.043225	Up	1.02E-116	2.68E-115
	CL3734.Contig3_All	0.7561	1.5127	1.000477	Up	9.41E-12	2.96E-11
	Unigene15649_All	24.7612	9.7861	-1.33928	Down	1.33E-96	2.93E-95
	Unigene18157_All	6.3084	2.989	-1.07761	Down	9.09E-19	4.43E-18
heat shock proteins (hsps)	Unigene56850_All	0	17.9473	14.13148	Up	3.82E-52	4.83E-51
	CL6394.Contig2_All	0	4.7607	12.21696	Up	4.02E-28	2.89E-27
	Unigene60034_All	0	4.1262	12.0106	Up	7.34E-13	2.51E-12
	Unigene55965_All	0	4.0346	11.97821	Up	2.32E-45	2.59E-44
	CL7360.Contig1_All	0	3.975	11.95674	Up	1.15E-17	5.29E-17
	CL6892.Contig4_All	0	3.9481	11.94694	Up	7.72E-15	3.01E-14
	CL208.Contig3_All	0	3.8872	11.92452	Up	2.98E-29	2.22E-28
	CL7108.Contig2_All	0	3.7432	11.87006	Up	1.27E-34	1.11E-33

	CL153.Contig9_All	0	3.5318	11.78619	Up	2.57E-10	7.18E-10
	Unigene47084_All	0	3.5103	11.77738	Up	2.44E-21	1.35E-20
	Unigene41470_All	0	3.5006	11.77339	Up	3.13E-18	1.49E-17
	Unigene46488_All	0	3.3998	11.73123	Up	6.32E-20	3.26E-19
	CL3021.Contig2_All	0	3.1353	11.61439	Up	2.56E-23	1.54E-22
	Unigene58214_All	0	3.0192	11.55995	Up	2.56E-23	1.54E-22
	CL4432.Contig1_All	0	2.8771	11.4904	Up	9.43E-10	2.51E-09
	Unigene41663_All	0	2.8622	11.48291	Up	1.41E-12	4.70E-12
	CL6892.Contig2_All	0	2.6227	11.35684	Up	3.83E-13	1.34E-12
	Unigene61318_All	0	2.5826	11.33461	Up	1.34E-10	3.83E-10
	Unigene61530_All	0	2.5678	11.32632	Up	1.72E-07	3.70E-07
	Unigene62098_All	0	2.3377	11.19087	Up	4.68E-08	1.06E-07
	Unigene65221_All	0	2.2621	11.14345	Up	3.64E-11	1.09E-10
	Unigene40845_All	0	2.226	11.12024	Up	1.81E-09	4.68E-09
	CL7360.Contig2_All	0	2.053	11.00352	Up	4.68E-08	1.07E-07
	CL9748.Contig2_All	0	2.0353	10.99103	Up	3.64E-11	1.09E-10
	Unigene64461_All	0	2.0047	10.96917	Up	7.34E-13	2.51E-12
	Unigene64008_All	0	1.9737	10.94669	Up	4.45E-06	8.26E-06
	Unigene44693_All	0	1.9687	10.94303	Up	3.30E-07	6.85E-07
	Unigene67922_All	0	1.9632	10.93899	Up	2.32E-06	4.41E-06
	CL4901.Contig2_All	0	1.9621	10.93818	Up	8.53E-19	4.17E-18
	Unigene69030_All	0	1.9365	10.91924	Up	2.32E-06	4.40E-06
	Unigene50009_All	0	1.9185	10.90576	Up	2.10E-15	8.48E-15
	Unigene41472_All	0	1.8147	10.82552	Up	1.72E-07	3.69E-07
	Unigene71123_All	0	1.654	10.69174	Up	3.14E-05	5.29E-05
	Unigene54496_All	0	1.6386	10.67825	Up	1.64E-05	2.81E-05
	CL4197.Contig2_All	0	1.6226	10.66409	Up	3.14E-05	5.27E-05
	Unigene68446_All	0	1.6007	10.64449	Up	4.45E-06	8.19E-06

	Unigene64296_All	0	1.5251	10.57469	Up	9.43E-10	2.51E-09
	Unigene72538_All	0	1.4979	10.54873	Up	0.0001153	0.0001814
	Unigene74449_All	0	1.4694	10.52101	Up	3.14E-05	5.27E-05
	Unigene75918_All	0	1.4493	10.50114	Up	1.64E-05	2.82E-05
	Unigene72562_All	0	1.4403	10.49215	Up	0.0001153	0.0001814
	CL3021.Contig1_All	0	1.4325	10.48432	Up	3.14E-05	5.25E-05
	Unigene71460_All	0	1.3887	10.43952	Up	6.32E-07	1.29E-06
	CL208.Contig1_All	0	1.3507	10.39949	Up	4.45E-06	8.22E-06
	Unigene76735_All	0	1.3247	10.37145	Up	0.000221	0.0003375
	Unigene75119_All	0	1.2748	10.31606	Up	0.0001153	0.0001815
	CL9748.Contig1_All	0	1.2201	10.25278	Up	0.000221	0.0003373
	Unigene71411_All	0	1.2037	10.23326	Up	8.54E-06	1.53E-05
	CL9833.Contig1_All	0	1.1888	10.21529	Up	0.0004237	0.0006238
	Unigene77869_All	0	1.1801	10.20469	Up	6.01E-05	9.79E-05
	CL7111.Contig2_All	0.0362	8.0452	7.795995	Up	2.43E-133	7.14E-132
	CL6066.Contig1_All	0.0987	15.4621	7.29147	Up	0	0
	CL153.Contig1_All	0.0594	8.7738	7.206595	Up	6.89E-131	2.00E-129
	CL4432.Contig2_All	0.0527	7.2296	7.099969	Up	3.44E-81	6.52E-80
	Unigene40442_All	0.0593	7.7006	7.020795	Up	1.16E-114	3.02E-113
	Unigene57668_All	0.042	5.1572	6.940055	Up	9.21E-37	8.48E-36
	CL377.Contig1_All	0.0777	9.3459	6.910275	Up	1.10E-175	4.20E-174
	CL2203.Contig2_All	1.282	151.0213	6.880212	Up	0	0
	CL1146.Contig1_All	0.6135	66.9255	6.769349	Up	0	0
	CL4363.Contig1_All	0.0266	2.684	6.656815	Up	4.61E-30	3.53E-29
	CL5394.Contig1_All	0.141	13.5115	6.582349	Up	7.00E-166	2.51E-164
	CL5126.Contig1_All	0.1535	13.3976	6.447592	Up	2.69E-150	8.82E-149
	Unigene42709_All	0.053	4.5322	6.418075	Up	3.21E-74	5.63E-73
	CL4901.Contig1_All	0.0726	6.1488	6.404192	Up	2.19E-73	3.81E-72

	CL5584.Contig1_All	0.3584	30.1872	6.396222	Up	0	0
	CL7108.Contig3_All	0.2307	18.1345	6.296575	Up	9.59E-157	3.26E-155
	Unigene42130_All	0.049	3.8212	6.2851	Up	4.17E-23	2.49E-22
	CL6729.Contig1_All	0.396	30.2434	6.254976	Up	0	0
	Unigene54816_All	0.0956	7.2791	6.250606	Up	1.50E-22	8.73E-22
	CL153.Contig2_All	0.7405	55.8211	6.236167	Up	0	0
	Unigene47117_All	0.0429	2.8715	6.064683	Up	8.88E-20	4.56E-19
	CL4197.Contig1_All	0.0398	2.3371	5.875807	Up	2.74E-17	1.23E-16
	Unigene51226_All	0.1019	5.6066	5.7819	Up	3.48E-16	1.47E-15
	CL204.Contig1_All	0.3147	16.8481	5.742465	Up	1.16E-191	4.85E-190
	CL153.Contig8_All	0.0753	3.9346	5.707423	Up	2.33E-15	9.38E-15
	CL1146.Contig2_All	0.1161	5.8554	5.656328	Up	8.29E-15	3.22E-14
	CL6066.Contig2_All	0.0538	2.6132	5.602068	Up	2.94E-14	1.11E-13
	Unigene53957_All	0.0695	3.3754	5.601902	Up	1.58E-27	1.11E-26
	CL6066.Contig3_All	0.0646	3.0824	5.576376	Up	5.54E-14	2.04E-13
	Unigene40550_All	0.0184	0.862	5.54991	Up	1.04E-13	3.79E-13
	CL6340.Contig1_All	0.0703	3.0723	5.449651	Up	2.33E-36	2.13E-35
	Unigene41469_All	0.0968	4.1732	5.430003	Up	1.30E-12	4.39E-12
	Unigene42764_All	0.0556	2.2927	5.365819	Up	4.60E-12	1.48E-11
	Unigene58340_All	0.0844	3.173	5.232461	Up	5.85E-21	3.18E-20
	Unigene49012_All	0.0728	2.4913	5.096816	Up	6.62E-28	4.73E-27
	Unigene65119_All	0.054	1.7832	5.045365	Up	1.31E-09	3.43E-09
	Unigene54240_All	0.2258	6.9718	4.948414	Up	6.31E-25	4.05E-24
	Unigene58396_All	0.127	3.6975	4.86365	Up	8.75E-31	6.84E-30
	Unigene40844_All	0.2842	7.5325	4.72815	Up	2.00E-67	3.22E-66
	CL9833.Contig2_All	0.2242	5.8584	4.707649	Up	2.36E-66	3.74E-65
	CL2896.Contig1_All	0.1289	2.8768	4.480141	Up	2.04E-17	9.28E-17
	CL6892.Contig1_All	0.1748	3.8069	4.44484	Up	3.18E-43	3.40E-42

	Unigene58522_All	0.1713	3.2589	4.249788	Up	4.68E-19	2.31E-18
	CL6394.Contig1_All	0.1414	2.3344	4.045198	Up	1.25E-08	3.02E-08
	CL9833.Contig3_All	0.3415	4.3145	3.659236	Up	3.90E-25	2.53E-24
	CL3.Contig2_All	0.6974	7.674	3.45992	Up	6.38E-30	4.86E-29
	Unigene57785_All	0.3736	3.3468	3.163216	Up	8.14E-23	4.79E-22
	Unigene56350_All	0.6646	5.6954	3.099239	Up	1.38E-41	1.42E-40
	Unigene74331_All	0.1837	1.5164	3.045227	Up	0.0004573	0.0006708
	Unigene17140_All	11.0517	89.9212	3.024393	Up	0	0
	CL9833.Contig4_All	0.6097	4.4729	2.875039	Up	1.97E-28	1.43E-27
	Unigene59502_All	0.3801	2.7881	2.874831	Up	2.03E-07	4.32E-07
	Unigene48015_All	0.3857	2.7412	2.829257	Up	6.39E-06	1.16E-05
	Unigene62869_All	0.2945	2.0251	2.781654	Up	1.09E-05	1.93E-05
	CL153.Contig3_All	0.8508	3.9379	2.210535	Up	1.68E-13	6.04E-13
	CL377.Contig2_All	0.6543	2.5332	1.952937	Up	5.47E-05	8.94E-05
	Unigene12226_All	54.7162	210.3161	1.942519	Up	0	0
	CL4882.Contig1_All	0.6384	2.4499	1.94019	Up	2.50E-12	8.23E-12
	Unigene42714_All	0.6667	2.4926	1.902542	Up	2.55E-06	4.80E-06
	Unigene65296_All	0.5818	2.1339	1.874897	Up	0.0001341	0.0002085
	CL7694.Contig4_All	6.0122	18.205	1.59837	Up	3.57E-146	1.14E-144
	Unigene9692_All	26.1439	78.4381	1.58508	Up	0	0
	CL3644.Contig1_All	9.3832	26.9907	1.52431	Up	4.12E-125	1.15E-123
	CL8530.Contig1_All	37.0273	102.2088	1.464858	Up	0	0
	Unigene14809_All	343.1219	819.1321	1.255375	Up	0	0
	Unigene10755_All	4.8086	10.2887	1.097372	Up	2.42E-11	7.36E-11
	CL4882.Contig2_All	2.1303	4.3286	1.022844	Up	1.16E-08	2.80E-08
	Unigene34110_All	7.6676	0.3057	-4.64859	Down	8.78E-25	5.60E-24
	Unigene30005_All	2.917	0.214	-3.7688	Down	1.04E-06	2.06E-06
	Unigene35830_All	5.8516	0.5172	-3.50004	Down	1.40E-18	6.75E-18

	Unigene31013_All	1.7454	0.2371	-2.87999	Down	6.54E-06	1.19E-05
	Unigene23676_All	34.2175	6.0109	-2.50908	Down	2.86E-78	5.25E-77
	Unigene7093_All	32.3627	5.9815	-2.43575	Down	4.11E-201	1.79E-199
	Unigene22635_All	44.4853	8.4275	-2.40015	Down	3.79E-225	1.84E-223
	Unigene29629_All	3.3179	0.8113	-2.03196	Down	0.0001021	0.000162
	Unigene24990_All	35.6333	10.9091	-1.70769	Down	3.37E-57	4.63E-56
	Unigene4498_All	197.2806	72.5761	-1.44268	Down	0	0
	Unigene19734_All	2655.767	990.8269	-1.42242	Down	0	0
	CL7629.Contig1_All	448.1632	174.5098	-1.36072	Down	0	0
period circadian（per）	Unigene54712_All	0	2.4356	11.25006	Up	1.27E-08	3.06E-08
	Unigene66073_All	0	1.5816	10.62717	Up	1.64E-05	2.83E-05
	Unigene69117_All	0.1687	1.5469	3.196848	Up	2.52E-06	4.75E-06
	Unigene13592_All	2.4649	8.219	1.737434	Up	1.08E-24	6.83E-24
	Unigene22608_All	14.9783	4.3023	-1.79969	Down	1.29E-106	3.13E-105
timeless（time）	Unigene54182_All	0.3326	2.5922	2.962317	Up	2.22E-09	5.70E-09
	Unigene55167_All	0.5736	3.488	2.604283	Up	8.04E-19	3.94E-18
	Unigene54181_All	0.718	2.6636	1.891322	Up	1.05E-09	2.78E-09
	Unigene58410_All	0.9381	3.2259	1.781888	Up	3.81E-09	9.58E-09
	CL2933.Contig1_All	1.7979	4.1174	1.195421	Up	4.34E-25	2.81E-24
glutathione reductase	CL3288.Contig1_All	0	4.1011	12.0018	Up	2.98E-29	2.22E-28
	CL8333.Contig1_All	0	2.8711	11.48739	Up	8.53E-19	4.17E-18
	CL8333.Contig2_All	0	2.2853	11.15817	Up	6.32E-07	1.28E-06
	CL3143.Contig2_All	0	2.0655	11.01228	Up	8.97E-08	1.98E-07
	Unigene54122_All	0	1.9677	10.94229	Up	1.72E-07	3.70E-07
	CL3143.Contig1_All	0	1.3418	10.38995	Up	4.45E-06	8.22E-06
	CL5933.Contig1_All	0.6531	8.1443	3.640415	Up	3.54E-79	6.57E-78
glutathione peroxidase	CL1620.Contig1_All	0	5.7672	12.49366	Up	6.62E-74	1.16E-72
	Unigene50933_All	0	2.4908	11.28239	Up	2.32E-19	1.17E-18

	Unigene43915_All	0	2.0519	11.00274	Up	1.34E-10	3.84E-10
	Unigene64514_All	0	1.9608	10.93723	Up	3.30E-07	6.87E-07
	Unigene70846_All	0	1.5137	10.56386	Up	6.32E-07	1.28E-06
	Unigene75564_All	0	1.3776	10.42794	Up	6.01E-05	9.79E-05
	CL6956.Contig2_All	0.0259	16.1795	9.286999	Up	6.15E-191	2.56E-189
	Unigene56972_All	0.042	15.4717	8.525027	Up	4.94E-112	1.26E-110
	Unigene50378_All	0.0641	5.1145	6.318125	Up	1.16E-23	7.10E-23
	Unigene41092_All	0.3889	12.4826	5.004375	Up	1.33E-75	2.37E-74
	CL1620.Contig2_All	0.0302	0.5544	4.198307	Up	2.57E-05	4.35E-05
	CL532.Contig1_All	0.8862	3.8825	2.131282	Up	5.41E-06	9.87E-06
	CL90.Contig2_All	3.0789	9.2568	1.588099	Up	3.58E-45	3.99E-44
	CL8706.Contig1_All	6.057	13.5358	1.160105	Up	8.53E-48	9.98E-47
	CL90.Contig1_All	267.0165	45.5817	-2.5504	Down	0	0
	CL4137.Contig1_All	43.4555	10.2837	-2.07918	Down	0	0
trehalase	Unigene55562_All	0	4.0347	11.97825	Up	7.33E-39	7.12E-38
	Unigene59612_All	0	2.7056	11.40173	Up	1.41E-25	9.25E-25
	Unigene59306_All	0	2.5215	11.30007	Up	3.64E-11	1.09E-10
	Unigene43089_All	0	2.4428	11.25432	Up	8.97E-08	1.99E-07
	Unigene41190_All	0	2.1788	11.08932	Up	1.27E-08	3.06E-08
	Unigene42174_All	0	2.1338	11.05921	Up	1.34E-10	3.83E-10
	Unigene70780_All	0	1.5114	10.56167	Up	4.45E-06	8.19E-06
	Unigene77838_All	0	1.4266	10.47837	Up	6.01E-05	9.72E-05
	CL4624.Contig1_All	0	1.4115	10.46301	Up	8.97E-08	1.99E-07
	Unigene77426_All	0	1.1262	10.13725	Up	0.0001153	0.0001815
	Unigene56161_All	0.0245	4.2169	7.427257	Up	7.04E-52	8.86E-51
	CL4624.Contig2_All	0.0237	3.5375	7.221699	Up	7.11E-45	7.87E-44
	Unigene42949_All	0.0306	3.2813	6.744592	Up	5.15E-32	4.17E-31
	Unigene52554_All	0.1029	2.9815	4.856723	Up	5.58E-38	5.30E-37

	Unigene57468_All	0.4276	5.1536	3.591247	Up	1.04E-18	5.04E-18
	Unigene61545_All	0.3779	2.0792	2.459952	Up	0.0002545	0.0003839
	CL6093.Contig1_All	0.0936	0.4863	2.377266	Up	1.68E-05	2.88E-05
	Unigene2681_All	2.4391	9.8047	2.007125	Up	4.43E-109	1.10E-107
	CL3008.Contig10_All	1.2593	0	-10.2984	Down	2.70E-36	2.46E-35
	CL3008.Contig1_All	0.2757	0	-8.10696	Down	3.14E-05	5.23E-05
	CL3008.Contig6_All	0.2183	0	-7.77017	Down	0.0001373	0.0002133
	CL3008.Contig2_All	0.1833	0	-7.51806	Down	7.19E-06	1.30E-05
	Unigene33106_All	9.9912	0.1922	-5.69998	Down	1.15E-41	1.19E-40
	CL3008.Contig7_All	0.3935	0.02	-4.29829	Down	5.98E-10	1.62E-09
sorbitol dehydrogenase	Unigene46324_All	0	4.7783	12.22228172	Up	1.04E-13	3.78E-13
	Unigene40674_All	0	3.4561	11.75492925	Up	5.44E-14	2.02E-13
	Unigene58847_All	0	2.2525	11.13731139	Up	9.43E-10	2.51E-09
	Unigene74137_All	0	1.2184	10.25077213	Up	3.14E-05	5.23E-05
	Unigene42085_All	0.0622	2.0543	5.045588491	Up	1.31E-09	3.43E-09
	Unigene42230_All	0.0605	1.9957	5.043815914	Up	1.31E-09	3.43E-09
	Unigene67507_All	0.0711	2.1529	4.920287939	Up	8.50E-09	2.07E-08
	Unigene32223_All	16.5888	0.2215	-6.226759019	Down	0	0
	Unigene8518_All	53.1004	2.0849	-4.670672636	Down	0	0
	Unigene17477_All	25.1652	3.5941	-2.807727609	Down	9.21E-210	4.16E-208
glycogen phosphorylase	CL7318.Contig3_All	0	2.0831	11.02452	Up	6.32E-07	1.27E-06
	CL7318.Contig2_All	0	0.7336	9.51885	Up	2.98E-29	2.22E-28
	CL7318.Contig1_All	0.008	1.1409	7.155957	Up	6.48E-43	6.86E-42
	Unigene56680_All	0.1735	1.591	3.196926	Up	0.000148	0.00023
fork head transcription factor (*foxo*)	Unigene24607_All	2.4513	7.7772	1.665704	Up	5.37E-15	2.11E-14
	Unigene10429_All	2.019	5.4872	1.442429	Up	2.14E-27	1.50E-26

**Table S3 Differentially expressed genes within metabolism pathways in response to diapause.**			
genenID	log 2 ratio	up-and-down	nr annotation
CL5452.Contig3_All	13.90434297	Up	NADH dehydrogenase [ubiquinone] 1 alpha subcomplex subunit 2 [Bombyx mori] >gi|87248185|gb|ABD36145.1| NADH-ubiquinone oxidoreductase B8 subunit [Bombyx mori]
CL3202.Contig3_All	13.60691409	Up	transketolase [Acanthamoeba castellanii str. Neff] >gi|440804204|gb|ELR25081.1| transketolase [Acanthamoeba castellanii str. Neff]
Unigene50489_All	13.34735838	Up	hCG1793893 [Homo sapiens]
Unigene57155_All	13.01600734	Up	uncharacterized protein LOC100216301 [Xenopus (Silurana) tropicalis]
Unigene43688_All	12.87690059	Up	succinate dehydrogenase [Schistosoma mansoni]
Unigene56688_All	12.83634628	Up	PREDICTED: UTP--glucose-1-phosphate uridylyltransferase-like [Strongylocentrotus purpuratus]
Unigene46521_All	12.79342042	Up	Triosephosphate isomerase [Harpegnathos saltator]
Unigene56038_All	12.72568578	Up	hypothetical protein BRAFLDRAFT_266022 [Branchiostoma floridae] >gi|229274753|gb|EEN45584.1| hypothetical protein BRAFLDRAFT_266022 [Branchiostoma floridae]
CL4164.Contig1_All	12.70141477	Up	hypothetical protein TRIADDRAFT_54073 [Trichoplax adhaerens] >gi|190586747|gb|EDV26800.1| hypothetical protein TRIADDRAFT_54073 [Trichoplax adhaerens]
CL9283.Contig2_All	12.69575028	Up	ATP synthase subunit beta [Oxytricha trifallax]
CL2979.Contig3_All	12.65378555	Up	CRE-GLN-3 protein [Caenorhabditis remanei] >gi|308243491|gb|EFO87443.1| CRE-GLN-3 protein [Caenorhabditis remanei]
CL8554.Contig1_All	12.64880695	Up	sigma-class glutathione S-transferase [Laternula elliptica]
Unigene43175_All	12.61776589	Up	acetyl-Coenzyme A acyltransferase 1 (peroxisomal 3-oxoacyl-Coenzyme A thiolase) [Gallus gallus]
CL3411.Contig1_All	12.60012198	Up	Chain J, Orientation Of Rna Polymerase Ii Within The Human Vp16-Mediator-Pol Ii-Tfiif Assembly
Unigene57242_All	12.56016562	Up	AMP-dependent synthetase and ligase domain-containing protein [Polysphondylium pallidum PN500]
CL2066.Contig1_All	12.55925758	Up	hCG1793893 [Homo sapiens]
CL5340.Contig2_All	12.55322129	Up	inosine monophosphate dehydrogenase 2 [Capsaspora owczarzaki ATCC 30864] >gi|320164547|gb|EFW41446.1| inosine monophosphate dehydrogenase 2 [Capsaspora owczarzaki ATCC 30864]
Unigene55652_All	12.50071742	Up	chloride channel, putative [Toxoplasma gondii GT1]
Unigene53174_All	12.49215363	Up	glycerol kinase [Culex quinquefasciatus] >gi|167867273|gb|EDS30656.1| glycerol kinase [Culex

			quinquefasciatus]
CL6593.Contig1_All	12.48675962	Up	isopentenyl pyrophosphate isomerase [Capsaspora owczarzaki ATCC 30864] >gi|320170746|gb|EFW47645.1| isopentenyl pyrophosphate isomerase [Capsaspora owczarzaki ATCC 30864]
Unigene57293_All	12.47403544	Up	PREDICTED: 2-oxoglutarate dehydrogenase-like, mitochondrial-like [Anolis carolinensis]
Unigene53252_All	12.46961638	Up	Chain I, Orientation Of Rna Polymerase Ii Within The Human Vp16-Mediator-Pol Ii-Tfiif Assembly
CL8923.Contig1_All	12.46648438	Up	aldo/keto reductase [Acanthamoeba castellanii str. Neff] >gi|440794183|gb|ELR15352.1| aldo/keto reductase [Acanthamoeba castellanii str. Neff]
CL2912.Contig1_All	12.45525015	Up	glucose phosphate isomerase a [Capsaspora owczarzaki ATCC 30864] >gi|320165556|gb|EFW42455.1| glucose phosphate isomerase a [Capsaspora owczarzaki ATCC 30864]
CL3525.Contig2_All	12.45113382	Up	NADH dehydrogenase 1 beta subcomplex subunit 9 [Salmo salar]
CL6956.Contig1_All	12.44976769	Up	unknown [Dendroctonus ponderosae]
Unigene42153_All	12.44050585	Up	PREDICTED: probable isocitrate dehydrogenase [NAD] subunit alpha, mitochondrial-like [Hydra magnipapillata]
CL9577.Contig1_All	12.42896204	Up	adenylate kinase [Dictyostelium purpureum] >gi|325079996|gb|EGC33570.1| adenylate kinase [Dictyostelium purpureum]
CL548.Contig2_All	12.42227506	Up	PREDICTED: LOW QUALITY PROTEIN: aldose reductase-like [Apis florea]
Unigene42561_All	12.40984171	Up	dihydrolipoamide acetyltransferase component of pyruvate dehydrogenase [Culex quinquefasciatus] >gi|167880446|gb|EDS43829.1| dihydrolipoamide acetyltransferase component of pyruvate dehydrogenase [Culex quinquefasciatus]
CL4629.Contig2_All	12.39716655	Up	uncharacterized protein LOC100216301 [Xenopus (Silurana) tropicalis]
Unigene41986_All	12.39513244	Up	Chain D, Chicken Cytochrome Bc1 Complex Inhibited By An Iodinated Analogue Of The Polyketide Crocacin-d >gi|196049788|pdb|3CWB|Q Chain Q, Chicken Cytochrome Bc1 Complex Inhibited By An Iodinated Analogue Of The Polyketide Crocacin-d >gi|228312416|pdb|3H1H|D Chain D, Cytochrome Bc1 Complex From Chicken >gi|228312426|pdb|3H1H|Q Chain Q, Cytochrome Bc1 Complex From Chicken >gi|228312438|pdb|3H1I|D Chain D, Stigmatellin And Antimycin Bound Cytochrome Bc1 Complex From Chicken >gi|228312448|pdb|3H1I|Q Chain Q, Stigmatellin And Antimycin Bound Cytochrome Bc1 Complex From Chicken >gi|228312461|pdb|3H1J|D Chain D, Stigmatellin-Bound

			Cytochrome Bc1 Complex From Chicken >gi|228312471|pdb|3H1J|Q Chain Q, Stigmatellin-Bound Cytochrome Bc1 Complex From Chicken >gi|228312485|pdb|3H1K|D Chain D, Chicken Cytochrome Bc1 Complex With Zn++ And An Iodinated Derivative Of Kresoxim-Methyl Bound >gi|228312495|pdb|3H1K|Q Chain Q, Chicken Cytochrome Bc1 Complex With Zn++ And An Iodinated Derivative Of Kresoxim-Methyl Bound >gi|283135346|pdb|3H1L|D Chain D, Chicken Cytochrome Bc1 Complex With Ascochlorin Bound At Qo And Qi Sites >gi|283135356|pdb|3H1L|Q Chain Q, Chicken Cytochrome Bc1 Complex With Ascochlorin Bound At Qo And Qi Sites >gi|285803641|pdb|3L70|D Chain D, Cytochrome Bc1 Complex From Chicken With Trifloxystrobin Bound >gi|285803651|pdb|3L70|Q Chain Q, Cytochrome Bc1 Complex From Chicken With Trifloxystrobin Bound >gi|285803661|pdb|3L71|D Chain D, Cytochrome Bc1 Complex From Chicken With Azoxystrobin Bound >gi|285803671|pdb|3L71|Q Chain Q, Cytochrome Bc1 Complex From Chicken With Azoxystrobin Bound >gi|285803681|pdb|3L72|D Chain D, Chicken Cytochrome Bc1 Complex With Kresoxym-I-Dimethyl Bound >gi|285803691|pdb|3L72|Q Chain Q, Chicken Cytochrome Bc1 Complex With Kresoxym-I-Dimethyl Bound >gi|285803701|pdb|3L73|D Chain D, Cytochrome Bc1 Complex From Chicken With Triazolone Inhibitor >gi|285803711|pdb|3L73|Q Chain Q, Cytochrome Bc1 Complex From Chicken With Triazolone Inhibitor >gi|285803721|pdb|3L74|D Chain D, Cytochrome Bc1 Complex From Chicken With Famoxadone Bound >gi|285803731|pdb|3L74|Q Chain Q, Cytochrome Bc1 Complex From Chicken With Famoxadone Bound >gi|285803741|pdb|3L75|D Chain D, Cytochrome Bc1 Complex From Chicken With Fenamidone Bound >gi|285803751|pdb|3L75|Q Chain Q, Cytochrome Bc1 Complex From Chicken With Fenamidone Bound >gi|393715170|pdb|3TGU|D Chain D, Cytochrome Bc1 Complex From Chicken With Pfvs-Designed Moa Inhibitor Bound >gi|393715180|pdb|3TGU|Q Chain Q, Cytochrome Bc1 Complex From Chicken With Pfvs-Designed Moa Inhibitor Bound
Unigene57243_All	12.34393519	Up	PREDICTED: cytosolic non-specific dipeptidase isoform 2 [Trichechus manatus latirostris]
Unigene47469_All	12.34190794	Up	O-methyltransferase family 3 protein [Dictyostelium fasciculatum] >gi|328865480|gb|EGG13866.1| O-methyltransferase family 3 protein [Dictyostelium fasciculatum]
Unigene42102_All	12.33102888	Up	hypothetical protein BRAFLDRAFT_66041 [Branchiostoma floridae] >gi|229281466|gb|EEN52224.1| hypothetical protein BRAFLDRAFT_66041 [Branchiostoma floridae]
CL3059.Contig1_All	12.33049678	Up	HAD-superfamily hydrolase [Capsaspora owczarzaki ATCC 30864] >gi|320168056|gb|EFW44955.1|

			HAD-superfamily hydrolase [Capsaspora owczarzaki ATCC 30864]
Unigene40948_All	12.31625324	Up	adenylsulfate kinase [Acanthamoeba castellanii str. Neff] >gi|440802140|gb|ELR23079.1| adenylsulfate kinase [Acanthamoeba castellanii str. Neff]
CL592.Contig1_All	12.3140167	Up	hypothetical protein CAEBREN_04340 [Caenorhabditis brenneri]
Unigene57540_All	12.30831059	Up	coproporphyrinogen iii oxidase [Aedes aegypti] >gi|108870125|gb|EAT34350.1| AAEL013389-PA [Aedes aegypti]
Unigene55213_All	12.30366661	Up	hypothetical protein KGM_16433 [Danaus plexippus]
CL4229.Contig2_All	12.29777606	Up	RecName: Full=Allantoicase; AltName: Full=Allantoate amidinohydrolase
Unigene47690_All	12.29091161	Up	NADH dehydrogenase [ubiquinone] iron-sulfur protein 3, mitochondrial [Cricetulus griseus]
Unigene41777_All	12.29007646	Up	inosine-5'-monophosphate dehydrogenase, putative [Toxoplasma gondii GT1]
Unigene57439_All	12.28895256	Up	L-3-hydroxyacyl-Coenzyme A dehydrogenase [Xenopus (Silurana) tropicalis] >gi|54038609|gb|AAH84457.1| L-3-hydroxyacyl-Coenzyme A dehydrogenase, short chain [Xenopus (Silurana) tropicalis] >gi|89271994|emb|CAJ82220.1| short chain 3-hydroxyacyl-CoA dehydrogenase, mitochondrial precursor [Xenopus (Silurana) tropicalis]
Unigene57422_All	12.28384077	Up	PREDICTED: lactate dehydrogenase A-like 6B [Oryctolagus cuniculus]
Unigene41286_All	12.28190106	Up	mitochondrial complex II component succinate dehydrogenase alpha subunit [Nyctotherus ovalis]
Unigene57615_All	12.27166728	Up	short-chain dehydrogenase/reductase SDR [Capsaspora owczarzaki ATCC 30864] >gi|320162883|gb|EFW39782.1| short-chain dehydrogenase/reductase SDR [Capsaspora owczarzaki ATCC 30864]
CL9363.Contig1_All	12.26784007	Up	glyceraldehyde 3-phosphate dehydrogenase [Ostrea edulis]
Unigene47225_All	12.25971384	Up	phosphofructokinase [Ascaris suum]
CL1073.Contig1_All	12.25446768	Up	malate synthase [Capsaspora owczarzaki ATCC 30864] >gi|320165110|gb|EFW42009.1| malate synthase [Capsaspora owczarzaki ATCC 30864]
Unigene57233_All	12.24623586	Up	hypothetical protein DAPPUDRAFT_213488 [Daphnia pulex]
Unigene56222_All	12.236941	Up	aldehyde dehydrogenase [Capsaspora owczarzaki ATCC 30864] >gi|320166834|gb|EFW43733.1| aldehyde dehydrogenase [Capsaspora owczarzaki ATCC 30864]
Unigene47129_All	12.23664209	Up	RecName: Full=Adenylosuccinate synthetase; Short=AMPSase; Short=AdSS; AltName: Full=IMP--aspartate ligase

Unigene57730_All	12.2326008	Up	Rieske iron-sulfur protein 1 [Capsaspora owczarzaki ATCC 30864] >gi|320168907|gb|EFW45806.1| Rieske iron-sulfur protein 1 [Capsaspora owczarzaki ATCC 30864]
Unigene57904_All	12.21953157	Up	hypothetical protein BRAFLDRAFT_126900 [Branchiostoma floridae] >gi|229289011|gb|EEN59700.1| hypothetical protein BRAFLDRAFT_126900 [Branchiostoma floridae]
CL974.Contig1_All	12.21810911	Up	hypothetical protein [Monosiga brevicollis MX1] >gi|163776061|gb|EDQ89683.1| predicted protein [Monosiga brevicollis MX1]
CL2542.Contig1_All	12.21158415	Up	Long-chain-fatty-acid--CoA ligase 5 [Oxytricha trifallax]
CL9701.Contig1_All	12.19997901	Up	uncharacterized protein LOC100216301 [Xenopus (Silurana) tropicalis]
Unigene46461_All	12.18636189	Up	sphingosine hydroxylase [Acanthamoeba castellanii str. Neff] >gi|440790695|gb|ELR11975.1| sphingosine hydroxylase [Acanthamoeba castellanii str. Neff]
CL1281.Contig1_All	12.18493731	Up	expressed hypothetical protein [Trichoplax adhaerens] >gi|190588298|gb|EDV28340.1| expressed hypothetical protein, partial [Trichoplax adhaerens]
Unigene47624_All	12.1692987	Up	hypothetical protein DAPPUDRAFT_228809 [Daphnia pulex]
Unigene55342_All	12.14315997	Up	acetyl-CoA C-acetyltransferase [Polysphondylium pallidum PN500]
Unigene54474_All	12.14012678	Up	PREDICTED: adenosylhomocysteinase A-like [Strongylocentrotus purpuratus]
Unigene51521_All	12.13545278	Up	cytochrome c oxidase subunit VIb polypeptide 1 (ubiquitous) [Xenopus laevis] >gi|124481714|gb|AAI33272.1| LOC100037163 protein [Xenopus laevis]
Unigene58199_All	12.12221326	Up	hypothetical protein TRIADDRAFT_56939 [Trichoplax adhaerens] >gi|190584710|gb|EDV24779.1| hypothetical protein TRIADDRAFT_56939 [Trichoplax adhaerens]
CL2337.Contig1_All	12.11488103	Up	Chain A, Crystal Structure Of Udp-N-Acetylglucosamine Pyrophosphorylase (Agx2) From Mus Musculus At 2.50 A Resolution >gi|55670463|pdb|1VM8|B Chain B, Crystal Structure Of Udp-N-Acetylglucosamine Pyrophosphorylase (Agx2) From Mus Musculus At 2.50 A Resolution
Unigene51264_All	12.10253348	Up	branchedchain amino acid aminotransferase [Acanthamoeba castellanii str. Neff] >gi|440796596|gb|ELR17705.1| branchedchain amino acid aminotransferase [Acanthamoeba castellanii str. Neff]
Unigene59234_All	12.09724207	Up	ATP synthase beta subunit [Penaeus monodon]
CL5978.Contig2_All	12.09572667	Up	RecName: Full=5-methyltetrahydropteroyltriglutamate--homocysteine methyltransferase; AltName: Full=Cobalamin-independent methionine synthase; AltName: Full=Methionine synthase, vitamin-B12

			independent isozyme
Unigene57419_All	12.09572667	Up	PREDICTED: UTP--glucose-1-phosphate uridylyltransferase-like [Oryzias latipes]
Unigene51543_All	12.07417492	Up	Epoxide hydrolase 2 [Myotis davidii]
CL3345.Contig2_All	12.06329395	Up	PREDICTED: similar to malate dehydrogenase [Tribolium castaneum] >gi|270008524|gb|EFA04972.1| hypothetical protein TcasGA2_TC015050 [Tribolium castaneum]
Unigene57639_All	12.06329395	Up	isocitrate lyase [Capsaspora owczarzaki ATCC 30864] >gi|320165109|gb|EFW42008.1| isocitrate lyase [Capsaspora owczarzaki ATCC 30864]
Unigene55085_All	12.04538427	Up	hypothetical protein CRE_26702 [Caenorhabditis remanei] >gi|308260317|gb|EFP04270.1| hypothetical protein CRE_26702 [Caenorhabditis remanei]
Unigene58714_All	12.04388171	Up	PREDICTED: AMP deaminase 2-like, partial [Takifugu rubripes]
Unigene41968_All	12.03651707	Up	cytochrome c oxidase subunit 3 (mitochondrion) [Reclinomonas americana] >gi|2258337|gb|AAD11871.1| cytochrome c oxidase subunit 3 (mitochondrion) [Reclinomonas americana]
Unigene46964_All	12.02655802	Up	PREDICTED: cytochrome b-c1 complex subunit 6, mitochondrial-like [Strongylocentrotus purpuratus]
CL1442.Contig2_All	12.01767838	Up	PREDICTED: v-type proton ATPase 16 kDa proteolipid subunit-like [Amphimedon queenslandica]
Unigene42065_All	12.01000352	Up	GH21465 [Drosophila grimshawi] >gi|193902624|gb|EDW01491.1| GH21465 [Drosophila grimshawi]
CL5139.Contig3_All	12.00937377	Up	RE25922p [Drosophila melanogaster]
Unigene41806_All	12.00587012	Up	CBN-KAT-1 protein [Caenorhabditis brenneri]
CL3288.Contig1_All	12.00179521	Up	Adh3, partial [Nucella lapillus]
CL6388.Contig1_All	11.99992955	Up	aspartate aminotransferase P1 [Capsaspora owczarzaki ATCC 30864] >gi|320163574|gb|EFW40473.1| aspartate aminotransferase P1 [Capsaspora owczarzaki ATCC 30864]
Unigene40775_All	11.99918967	Up	PREDICTED: peroxisomal multifunctional enzyme type 2 isoform 4 [Pan paniscus]
Unigene58001_All	11.99290294	Up	PREDICTED: trifunctional nucleotide phosphoesterase protein YfkN-like isoform 2 [Acyrthosiphon pisum]
Unigene42307_All	11.97981815	Up	Pyrroline-5-carboxylate reductase 2 [Acromyrmex echinatior]
Unigene54674_All	11.97763769	Up	ARD/ARD' family protein [Tetrahymena thermophila] >gi|89306813|gb|EAS04801.1| ARD/ARD' family protein [Tetrahymena thermophila SB210]
Unigene57658_All	11.97412774	Up	4-hydroxyphenylpyruvate dioxygenase [Crassostrea gigas]
CL4087.Contig1_All	11.96491841	Up	DNA-directed RNA polymerases I, II, and III 7.0 kDa polypeptide, putative [Toxoplasma gondii

			GT1] >gi|221504088|gb|EEE29765.1| DNA-directed RNA polymerases I, II, and III 7.0 kDa polypeptide, putative [Toxoplasma gondii VEG]
Unigene52938_All	11.95782756	Up	phosphomannomutase 2 [Salpingoeca sp. ATCC 50818]
Unigene58321_All	11.95488695	Up	nucleoside diphosphate kinase Nm23-SD1 [Suberites domuncula]
CL6627.Contig1_All	11.95019135	Up	phosphoglycerate mutase, putative [Ixodes scapularis] >gi|215502938|gb|EEC12432.1| phosphoglycerate mutase, putative [Ixodes scapularis]
Unigene54856_All	11.94986318	Up	malate synthase A, putative [Acanthamoeba castellanii str. Neff] >gi|440802061|gb|ELR23000.1| malate synthase A, putative [Acanthamoeba castellanii str. Neff]
Unigene47536_All	11.93913841	Up	hypothetical protein [Monosiga brevicollis MX1] >gi|163773567|gb|EDQ87205.1| predicted protein [Monosiga brevicollis MX1]
CL7539.Contig2_All	11.93777839	Up	choline-phosphate cytidylyltransferase [Schistosoma mansoni]
Unigene49749_All	11.9308482	Up	PREDICTED: predicted protein-like [Saccoglossus kowalevskii]
Unigene58008_All	11.9308482	Up	uracil phosphoribosyltransferase [Dictyostelium purpureum] >gi|325081105|gb|EGC34634.1| uracil phosphoribosyltransferase [Dictyostelium purpureum]
Unigene58613_All	11.92933239	Up	PREDICTED: aldehyde dehydrogenase family 3 member B1-like [Ciona intestinalis]
Unigene55324_All	11.92581403	Up	PREDICTED: aspartate aminotransferase, cytoplasmic-like, partial [Mus musculus]
CL208.Contig3_All	11.92451562	Up	predicted protein [Nematostella vectensis] >gi|156227646|gb|EDO48448.1| predicted protein [Nematostella vectensis]
Unigene55002_All	11.92440428	Up	PREDICTED: choline-phosphate cytidylyltransferase B-like [Otolemur garnettii]
CL5668.Contig1_All	11.92228706	Up	Protein FUM-1, isoform a [Caenorhabditis elegans] >gi|39931306|sp|O17214.1|FUMH_CAEEL RecName: Full=Probable fumarate hydratase, mitochondrial; Short=Fumarase; Flags: Precursor >gi|351064049|emb|CCD72341.1| Protein FUM-1, isoform a [Caenorhabditis elegans]
Unigene40943_All	11.91737208	Up	hypothetical protein NCLIV_049080 [Neospora caninum Liverpool] >gi|325118927|emb|CBZ54479.1| hypothetical protein NCLIV_049080 [Neospora caninum Liverpool]
CL3119.Contig1_All	11.91045536	Up	PREDICTED: phosphoglucomutase-1-like [Takifugu rubripes]
Unigene40826_All	11.90989308	Up	PREDICTED: 4-hydroxyphenylpyruvate dioxygenase [Sarcophilus harrisii]
CL2711.Contig2_All	11.9077169	Up	oxidase heme a,cytochrome [Salpingoeca sp. ATCC 50818]
Unigene53907_All	11.90478513	Up	PREDICTED: inactive dipeptidyl peptidase 10-like [Takifugu rubripes]

CL1045.Contig2_All	11.90459699	Up	uncharacterized protein LOC100216301 [Xenopus (Silurana) tropicalis]
CL5159.Contig1_All	11.90395714	Up	AGAP006532-PA [Anopheles gambiae str. PEST] >gi|157016306|gb|EAA11440.4| AGAP006532-PA [Anopheles gambiae str. PEST]
Unigene57874_All	11.90373124	Up	predicted protein [Nematostella vectensis] >gi|156215761|gb|EDO36713.1| predicted protein [Nematostella vectensis]
Unigene54547_All	11.90358063	Up	hypothetical protein SINV_07367 [Solenopsis invicta]
CL3478.Contig1_All	11.89811008	Up	Medium-chain specific acyl-CoA dehydrogenase, putative [Perkinsus marinus ATCC 50983] >gi|239902533|gb|EER19323.1| Medium-chain specific acyl-CoA dehydrogenase, putative [Perkinsus marinus ATCC 50983]
CL3898.Contig1_All	11.89776984	Up	PREDICTED: adenylosuccinate lyase [Metaseiulus occidentalis]
Unigene54003_All	11.89678649	Up	PREDICTED: GDP-mannose pyrophosphorylase A-like [Saccoglossus kowalevskii]
Unigene58388_All	11.89239103	Up	AcylCoA oxidase [Acanthamoeba castellanii str. Neff] >gi|440789742|gb|ELR11041.1| AcylCoA oxidase [Acanthamoeba castellanii str. Neff]
Unigene55240_All	11.88271946	Up	PREDICTED: similar to acetyl-CoA acetyltransferase, mitochondrial [Tribolium castaneum] >gi|270001523|gb|EEZ97970.1| hypothetical protein TcasGA2_TC000365 [Tribolium castaneum]
Unigene42529_All	11.87190524	Up	hypothetical protein [Paramecium tetraurelia strain d4-2] >gi|124398596|emb|CAK64093.1| unnamed protein product [Paramecium tetraurelia]
CL8769.Contig1_All	11.87167426	Up	2-oxoglutarate dehydrogenase [Capsaspora owczarzaki ATCC 30864] >gi|320166651|gb|EFW43550.1| 2-oxoglutarate dehydrogenase [Capsaspora owczarzaki ATCC 30864]
Unigene59194_All	11.86584924	Up	pyruvate kinase [Capsaspora owczarzaki ATCC 30864] >gi|320167794|gb|EFW44693.1| pyruvate kinase [Capsaspora owczarzaki ATCC 30864]
Unigene49806_All	11.86259862	Up	catalase [Acanthamoeba castellanii str. Neff] >gi|372290526|gb|AEX91750.1| catalase 2 [Acanthamoeba castellanii] >gi|440792545|gb|ELR13756.1| catalase [Acanthamoeba castellanii str. Neff]
Unigene58007_All	11.85895231	Up	mitochondrial citrate synthetase [Naegleria gruberi] >gi|284091752|gb|EFC45397.1| mitochondrial citrate synthetase [Naegleria gruberi]
Unigene41580_All	11.85291959	Up	hypothetical protein DICPUDRAFT_48509 [Dictyostelium purpureum] >gi|325080636|gb|EGC34183.1| hypothetical protein DICPUDRAFT_48509 [Dictyostelium purpureum]

CL2497.Contig1_All	11.8491705	Up	uncharacterized protein LOC100216301 [Xenopus (Silurana) tropicalis]
Unigene49233_All	11.8491705	Up	PREDICTED: malate dehydrogenase, mitochondrial-like [Hydra magnipapillata]
Unigene40690_All	11.84006978	Up	PREDICTED: aspartate aminotransferase, cytoplasmic-like, partial [Mus musculus]
CL9630.Contig2_All	11.83991236	Up	PREDICTED: mannose-6-phosphate isomerase-like [Ciona intestinalis]
CL3946.Contig1_All	11.83407595	Up	glucose-6-phosphate isomerase [Nilaparvata lugens]
Unigene56024_All	11.83091128	Up	predicted protein [Nematostella vectensis] >gi|156226598|gb|EDO47406.1| predicted protein [Nematostella vectensis]
CL5084.Contig2_All	11.82948491	Up	expressed hypothetical protein [Trichoplax adhaerens] >gi|190587654|gb|EDV27696.1| expressed hypothetical protein [Trichoplax adhaerens]
Unigene42116_All	11.82861254	Up	3-phosphoglycerate dehydrogenase [Dictyostelium discoideum AX4] >gi|74855598|sp|Q54UH8.1|SERA_DICDI RecName: Full=D-3-phosphoglycerate dehydrogenase; Short=3-PGDH >gi|60468832|gb|EAL66832.1| 3-phosphoglycerate dehydrogenase [Dictyostelium discoideum AX4]
Unigene42851_All	11.82758088	Up	hypothetical protein [Monosiga brevicollis MX1] >gi|163777819|gb|EDQ91435.1| predicted protein [Monosiga brevicollis MX1]
CL1739.Contig2_All	11.8267868	Up	UDPglucose pyrophosphorylase 2 isoform, putative [Acanthamoeba castellanii str. Neff] >gi|440797781|gb|ELR18856.1| UDPglucose pyrophosphorylase 2 isoform, putative [Acanthamoeba castellanii str. Neff]
Unigene58069_All	11.82324781	Up	wos2 [Salpingoeca sp. ATCC 50818]
CL3385.Contig1_All	11.8153833	Up	hypothetical protein SINV_13338 [Solenopsis invicta]
CL3505.Contig1_All	11.81338039	Up	H+-ATPase B subunit, partial [Bos taurus]
Unigene54248_All	11.81193657	Up	Hypothetical protein CBG12633 [Caenorhabditis briggsae]
CL1082.Contig3_All	11.80848159	Up	acyl-coa dehydrogenase, putative [Perkinsus marinus ATCC 50983] >gi|239881088|gb|EER06951.1| acyl-coa dehydrogenase, putative [Perkinsus marinus ATCC 50983]
Unigene58302_All	11.8061468	Up	homogentisate 1,2-dioxygenase [Culex quinquefasciatus] >gi|167874202|gb|EDS37585.1| homogentisate 1,2-dioxygenase [Culex quinquefasciatus]
CL7475.Contig1_All	11.80562297	Up	PREDICTED: similar to acyl-CoA dehydrogenase [Tribolium castaneum]
CL1046.Contig2_All	11.80469572	Up	PREDICTED: inosine-5'-monophosphate dehydrogenase 1 isoform 5 [Gorilla gorilla gorilla]

CL2392.Contig1_All	11.80025281	Up	oxoglutarate dehydrogenase (succinyltransferring), E1 component, putative [Acanthamoeba castellanii str. Neff] >gi|440798433|gb|ELR19501.1| oxoglutarate dehydrogenase (succinyltransferring), E1 component, putative [Acanthamoeba castellanii str. Neff]
CL5177.Contig2_All	11.78937062	Up	4-aminobutyrate transaminase [Dictyostelium discoideum AX4] >gi|74897469|sp|Q55FI1.1|GABT_DICDI RecName: Full=4-aminobutyrate aminotransferase; AltName: Full=GABA aminotransferase; Short=GABA-AT; AltName: Full=Gamma-amino-N-butyrate transaminase; Short=GABA transaminase >gi|60475570|gb|EAL73505.1| 4-aminobutyrate transaminase [Dictyostelium discoideum AX4]
CL3085.Contig1_All	11.78716797	Up	unnamed protein product [Tetraodon nigroviridis]
CL2472.Contig2_All	11.78230208	Up	hypothetical protein [Monosiga brevicollis MX1] >gi|163771876|gb|EDQ85537.1| predicted protein [Monosiga brevicollis MX1]
Unigene58557_All	11.78070379	Up	HAG group protein [Polysphondylium pallidum PN500]
CL8352.Contig1_All	11.77910374	Up	unknown [Dendroctonus ponderosae]
Unigene49818_All	11.77815929	Up	NAD(+)/NADH kinase [Acanthamoeba castellanii str. Neff] >gi|440799118|gb|ELR20179.1| NAD(+)/NADH kinase [Acanthamoeba castellanii str. Neff]
Unigene57692_All	11.76710852	Up	vacuolar ATP synthase subunit B [Cryptosporidium muris RN66]
CL5688.Contig1_All	11.76466429	Up	6-phosphogluconate dehydrogenase [Polysphondylium pallidum PN500]
Unigene41229_All	11.76171743	Up	glutamyl-tRNA synthetase family protein [Cryptosporidium muris RN66] >gi|209555419|gb|EEA05464.1| glutamyl-tRNA synthetase family protein [Cryptosporidium muris RN66]
Unigene63463_All	11.75963856	Up	nucleoside diphosphate kinase Nm23-SD1 [Suberites domuncula]
Unigene59701_All	11.75951373	Up	dihydrolipoamide S-succinyltransferase [Salpingoeca sp. ATCC 50818]
CL1167.Contig1_All	11.75780667	Up	starch branching enzyme II [Schistosoma mansoni]
Unigene40674_All	11.75492925	Up	hypothetical protein CRE_29292 [Caenorhabditis remanei] >gi|308248779|gb|EFO92731.1| hypothetical protein CRE_29292 [Caenorhabditis remanei]
CL2066.Contig2_All	11.75104187	Up	hCG1793893 [Homo sapiens]
CL7588.Contig2_All	11.74697611	Up	hypothetical protein BRAFLDRAFT_125492 [Branchiostoma floridae] >gi|229293673|gb|EEN64332.1| hypothetical protein BRAFLDRAFT_125492 [Branchiostoma floridae]
Unigene53454_All	11.74323556	Up	PREDICTED: adenine phosphoribosyltransferase-like [Gallus gallus]

Unigene55844_All	11.73990716	Up	Bile salt-activated lipase [Acromyrmex echinatior]
Unigene50621_All	11.73695151	Up	PREDICTED: isocitrate dehydrogenase [NAD] subunit gamma, mitochondrial isoform 3 [Orcinus orca] >gi|470620563|ref|XP_004318504.1| PREDICTED: isocitrate dehydrogenase [NAD] subunit gamma, mitochondrial isoform 3 [Tursiops truncatus]
Unigene48002_All	11.73509056	Up	phospholipase D1 [Naegleria gruberi] >gi|284094267|gb|EFC47902.1| phospholipase D1 [Naegleria gruberi]
CL5277.Contig1_All	11.73089465	Up	vacuolar H[+] ATPase subunit 100-1, isoform B [Drosophila melanogaster] >gi|24650967|ref|NP_733275.1| vacuolar H[+] ATPase subunit 100-1, isoform D [Drosophila melanogaster] >gi|442621618|ref|NP_733276.3| vacuolar H[+] ATPase subunit 100-1, isoform K [Drosophila melanogaster] >gi|23172538|gb|AAN14158.1| vacuolar H[+] ATPase subunit 100-1, isoform B [Drosophila melanogaster] >gi|23172539|gb|AAN14159.1| vacuolar H[+] ATPase subunit 100-1, isoform D [Drosophila melanogaster] >gi|281183445|gb|ADA53591.1| RE06427p [Drosophila melanogaster] >gi|440218005|gb|AAN14160.3| vacuolar H[+] ATPase subunit 100-1, isoform K [Drosophila melanogaster]
Unigene41718_All	11.727027	Up	PREDICTED: NADH dehydrogenase [ubiquinone] iron-sulfur protein 2, mitochondrial-like [Acyrthosiphon pisum]
Unigene60061_All	11.71990214	Up	PREDICTED: probable medium-chain specific acyl-CoA dehydrogenase, mitochondrial-like [Megachile rotundata]
Unigene41911_All	11.7198166	Up	PREDICTED: aldehyde dehydrogenase 9 family, member A1a-like [Saccoglossus kowalevskii]
Unigene40829_All	11.71831881	Up	unnamed protein product [Oikopleura dioica] >gi|313226918|emb|CBY22063.1| unnamed protein product [Oikopleura dioica]
Unigene59039_All	11.7148036	Up	PREDICTED: L-lactate dehydrogenase-like isoform 1 [Strongylocentrotus purpuratus]
Unigene41158_All	11.7141167	Up	acylCoA dehydrogenase, middle domain containing protein [Acanthamoeba castellanii str. Neff] >gi|440801832|gb|ELR22836.1| acylCoA dehydrogenase, middle domain containing protein [Acanthamoeba castellanii str. Neff]
Unigene46306_All	11.71183902	Up	PREDICTED: similar to putative nucleoside diphosphate kinase [Tribolium castaneum]
Unigene41721_All	11.71145189	Up	thymidylate kinase family protein [Trichomonas vaginalis G3] >gi|121891703|gb|EAX97024.1| thymidylate kinase family protein [Trichomonas vaginalis G3]

<table>
<tr><td>CL392.Contig1_All</td><td>11.70968696</td><td>Up</td><td>Protein F22B3.4 [Caenorhabditis elegans] >gi|3876197|emb|CAA92735.1| Protein F22B3.4 [Caenorhabditis elegans]</td></tr>
<tr><td>Unigene41354_All</td><td>11.70822173</td><td>Up</td><td>unknown [Dendroctonus ponderosae]</td></tr>
<tr><td>CL1045.Contig3_All</td><td>11.70735913</td><td>Up</td><td>uncharacterized protein LOC100216301 [Xenopus (Silurana) tropicalis]</td></tr>
<tr><td>Unigene40848_All</td><td>11.70169633</td><td>Up</td><td>hypothetical protein [Monosiga brevicollis MX1] >gi|163775817|gb|EDQ89439.1| predicted protein [Monosiga brevicollis MX1]</td></tr>
<tr><td>Unigene62766_All</td><td>11.70009287</td><td>Up</td><td>CG11951, isoform A [Drosophila melanogaster] >gi|7301770|gb|AAF56882.1| CG11951, isoform A [Drosophila melanogaster]</td></tr>
<tr><td>Unigene42881_All</td><td>11.69952908</td><td>Up</td><td>PREDICTED: bifunctional purine biosynthesis protein PURH [Sarcophilus harrisii]</td></tr>
<tr><td>CL1763.Contig3_All</td><td>11.69492371</td><td>Up</td><td>PREDICTED: WD repeat-containing protein 35 [Ciona intestinalis]</td></tr>
<tr><td>Unigene52864_All</td><td>11.68479244</td><td>Up</td><td>PREDICTED: 1,5-anhydro-D-fructose reductase [Callithrix jacchus]</td></tr>
<tr><td>CL7847.Contig2_All</td><td>11.67864415</td><td>Up</td><td>hypothetical protein [Monosiga brevicollis MX1] >gi|163776528|gb|EDQ90147.1| predicted protein [Monosiga brevicollis MX1]</td></tr>
<tr><td>CL6132.Contig2_All</td><td>11.6690179</td><td>Up</td><td>Homogentisate 1,2-dioxygenase [Oxytricha trifallax]</td></tr>
<tr><td>Unigene58207_All</td><td>11.6673777</td><td>Up</td><td>DNA-directed RNA polymerase II, 16.5 kDa polypeptide [Oxytricha trifallax]</td></tr>
<tr><td>CL9283.Contig1_All</td><td>11.66688971</td><td>Up</td><td>RecName: Full=ATP synthase subunit beta, mitochondrial; Flags: Precursor >gi|391913|dbj|BAA04178.1| H(+)-transporting ATPase beta subunit [Hemicentrotus pulcherrimus] >gi|1094359|prf||2105433A H ATPase:SUBUNIT=beta</td></tr>
<tr><td>Unigene41141_All</td><td>11.66595763</td><td>Up</td><td>PREDICTED: V-type proton ATPase subunit H-like [Hydra magnipapillata]</td></tr>
<tr><td>CL4029.Contig1_All</td><td>11.6649361</td><td>Up</td><td>4-hydroxyphenylpyruvate dioxygenase [Papilio xuthus]</td></tr>
<tr><td>CL8757.Contig2_All</td><td>11.66382492</td><td>Up</td><td>hypothetical protein CAOG_07397 [Capsaspora owczarzaki ATCC 30864] >gi|320165655|gb|EFW42554.1| hypothetical protein CAOG_07397 [Capsaspora owczarzaki ATCC 30864]</td></tr>
<tr><td>Unigene43397_All</td><td>11.66155544</td><td>Up</td><td>asparagine synthetase [Capsaspora owczarzaki ATCC 30864] >gi|320167761|gb|EFW44660.1| asparagine synthetase [Capsaspora owczarzaki ATCC 30864]</td></tr>
<tr><td>Unigene51121_All</td><td>11.66017421</td><td>Up</td><td>Ribonucleoside-diphosphate reductase subunit M2 [Cricetulus griseus]</td></tr>
<tr><td>Unigene55266_All</td><td>11.65633547</td><td>Up</td><td>Adenylosuccinate synthetase isozyme 1, partial [Columba livia]</td></tr>
<tr><td>Unigene58703_All</td><td>11.654278</td><td>Up</td><td>hypothetical protein [Monosiga brevicollis MX1] >gi|163773810|gb|EDQ87446.1| predicted protein</td></tr>
</table>

<table>
<tr><td></td><td></td><td></td><td>[Monosiga brevicollis MX1]</td></tr>
<tr><td>Unigene51506_All</td><td>11.65315857</td><td>Up</td><td>dihydrolipoamide S-succinyltransferase [Salpingoeca sp. ATCC 50818]</td></tr>
<tr><td>CL6443.Contig1_All</td><td>11.65015421</td><td>Up</td><td>hypothetical protein DAPPUDRAFT_204767 [Daphnia pulex]</td></tr>
<tr><td>Unigene60478_All</td><td>11.64538824</td><td>Up</td><td>Hypothetical protein CBG20640 [Caenorhabditis briggsae]</td></tr>
<tr><td>Unigene60042_All</td><td>11.64331508</td><td>Up</td><td>unknown [Dendroctonus ponderosae]</td></tr>
<tr><td>Unigene41186_All</td><td>11.6379833</td><td>Up</td><td>dephospho-CoA kinase [Capsaspora owczarzaki ATCC 30864] >gi|320164849|gb|EFW41748.1| dephospho-CoA kinase [Capsaspora owczarzaki ATCC 30864]</td></tr>
<tr><td>Unigene41261_All</td><td>11.63630741</td><td>Up</td><td>CD8 T-cell antigen Ta9 [Theileria annulata]</td></tr>
<tr><td>Unigene46499_All</td><td>11.63426653</td><td>Up</td><td>Gfpt1 protein [Salpingoeca sp. ATCC 50818]</td></tr>
<tr><td>Unigene50940_All</td><td>11.63363101</td><td>Up</td><td>PREDICTED: trinucleotide repeat-containing gene 6B protein isoform 1 [Saimiri boliviensis boliviensis]</td></tr>
<tr><td>CL3824.Contig1_All</td><td>11.63254088</td><td>Up</td><td>PREDICTED: ATP-citrate synthase isoform 2 [Orcinus orca]</td></tr>
<tr><td>Unigene59277_All</td><td>11.62753388</td><td>Up</td><td>homogentisate 1,2-dioxygenase HgmA [Capsaspora owczarzaki ATCC 30864] >gi|320164646|gb|EFW41545.1| homogentisate 1,2-dioxygenase HgmA [Capsaspora owczarzaki ATCC 30864]</td></tr>
<tr><td>Unigene42555_All</td><td>11.62484114</td><td>Up</td><td>--</td></tr>
<tr><td>Unigene59409_All</td><td>11.62379006</td><td>Up</td><td>Aconitate hydratase, mitochondrial [Harpegnathos saltator]</td></tr>
<tr><td>Unigene47491_All</td><td>11.62049477</td><td>Up</td><td>zinc-containing alcohol dehydrogenase [Dictyostelium fasciculatum] >gi|328873913|gb|EGG22279.1| zinc-containing alcohol dehydrogenase [Dictyostelium fasciculatum]</td></tr>
<tr><td>CL2238.Contig2_All</td><td>11.61971562</td><td>Up</td><td>ubiquitin-conjugating enzyme E2 l [Capsaspora owczarzaki ATCC 30864] >gi|320167176|gb|EFW44075.1| ubiquitin-conjugating enzyme E2 l [Capsaspora owczarzaki ATCC 30864]</td></tr>
<tr><td>Unigene55319_All</td><td>11.61452582</td><td>Up</td><td>hexokinase-2 [Capsaspora owczarzaki ATCC 30864] >gi|320170275|gb|EFW47174.1| hexokinase-2 [Capsaspora owczarzaki ATCC 30864]</td></tr>
<tr><td>Unigene40896_All</td><td>11.61415769</td><td>Up</td><td>Adenylate kinase, putative [Oxytricha trifallax]</td></tr>
<tr><td>Unigene62624_All</td><td>11.61153205</td><td>Up</td><td>PREDICTED: deoxyuridine 5'-triphosphate nucleotidohydrolase, mitochondrial [Xenopus (Silurana) tropicalis]</td></tr>
<tr><td>CL5767.Contig1_All</td><td>11.60385812</td><td>Up</td><td>PREDICTED: coiled-coil and C2 domain-containing protein 2A [Ciona intestinalis]</td></tr>
<tr><td>Unigene63325_All</td><td>11.60385812</td><td>Up</td><td>PREDICTED: leukotriene A-4 hydrolase-like [Amphimedon queenslandica]</td></tr>
</table>

Unigene59256_All	11.59674883	Up	PREDICTED: sterol 26-hydroxylase, mitochondrial-like [Danio rerio]
Unigene60510_All	11.59651591	Up	PREDICTED: 6-phosphofructokinase, muscle type [Trichechus manatus latirostris]
CL7387.Contig1_All	11.59600335	Up	PREDICTED: membrane primary amine oxidase-like [Meleagris gallopavo]
CL8836.Contig1_All	11.59516422	Up	H+-ATPase B subunit, partial [Bos taurus]
Unigene50966_All	11.5918963	Up	hypothetical protein CAPTEDRAFT_162025 [Capitella teleta]
CL7939.Contig1_All	11.5904467	Up	Phospholipid methyltransferase [Acanthamoeba castellanii str. Neff] >gi|440791405|gb|ELR12643.1| Phospholipid methyltransferase [Acanthamoeba castellanii str. Neff]
CL7532.Contig3_All	11.58937026	Up	1-pyrroline-5-carboxylate dehydrogenase [Trichinella spiralis] >gi|316975641|gb|EFV59049.1| 1-pyrroline-5-carboxylate dehydrogenase [Trichinella spiralis]
CL8251.Contig1_All	11.58796499	Up	PREDICTED: adenosylhomocysteinase A-like [Strongylocentrotus purpuratus]
CL1780.Contig2_All	11.5797402	Up	lipoic acid synthase [Capsaspora owczarzaki ATCC 30864] >gi|320169262|gb|EFW46161.1| lipoic acid synthase [Capsaspora owczarzaki ATCC 30864]
CL3837.Contig2_All	11.57568105	Up	carbamylphosphate synthetase 1 [Salpingoeca sp. ATCC 50818]
Unigene51840_All	11.5726055	Up	NAD-specific glutamate dehydrogenase [Capsaspora owczarzaki ATCC 30864] >gi|320170749|gb|EFW47648.1| NAD-specific glutamate dehydrogenase [Capsaspora owczarzaki ATCC 30864]
CL5871.Contig1_All	11.56833618	Up	cystathionine beta-synthase [Dictyostelium purpureum] >gi|325078220|gb|EGC31883.1| cystathionine beta-synthase [Dictyostelium purpureum]
Unigene67096_All	11.5676234	Up	lanosterol 14-alpha-demethylase [Rattus norvegicus]
Unigene60584_All	11.5667676	Up	PREDICTED: 3-ketoacyl-CoA thiolase B, peroxisomal-like [Strongylocentrotus purpuratus]
CL4566.Contig2_All	11.5628625	Up	PREDICTED: cytochrome c1, heme protein, mitochondrial-like [Hydra magnipapillata]
CL1749.Contig1_All	11.56171754	Up	predicted protein [Nematostella vectensis] >gi|156219134|gb|EDO40020.1| predicted protein [Nematostella vectensis]
Unigene49248_All	11.54544718	Up	hypothetical protein TRIADDRAFT_50478 [Trichoplax adhaerens] >gi|190583001|gb|EDV23072.1| hypothetical protein TRIADDRAFT_50478 [Trichoplax adhaerens]
Unigene60830_All	11.53974043	Up	cystathionine beta-lyase [Capsaspora owczarzaki ATCC 30864] >gi|320169602|gb|EFW46501.1| cystathionine beta-lyase [Capsaspora owczarzaki ATCC 30864]
CL6812.Contig3_All	11.53809191	Up	PREDICTED: peroxisomal multifunctional enzyme type 2-like isoform 1 [Ciona intestinalis]

Unigene65409_All	11.53585856	Up	PREDICTED: UDP-glucuronosyltransferase 2B10-like [Acyrthosiphon pisum]
CL6260.Contig2_All	11.53527538	Up	C30D10.14 [Salmo salar] >gi|221221092|gb|ACM09207.1| C30D10.14 [Salmo salar]
Unigene40903_All	11.53240463	Up	PREDICTED: phosphatidylserine decarboxylase proenzyme-like [Strongylocentrotus purpuratus]
Unigene60373_All	11.53055265	Up	glyceraldehyde 3-phosphate dehydrogenase [Haliotis discus discus]
Unigene53842_All	11.52899124	Up	PREDICTED: DNA polymerase epsilon catalytic subunit A-like isoform 2 [Acyrthosiphon pisum]
Unigene40645_All	11.51244419	Up	PREDICTED: LOW QUALITY PROTEIN: CAD protein, partial [Felis catus]
CL2673.Contig2_All	11.51219725	Up	3-hydroxybutyrate dehydrogenase type 2 [Caligus clemensi]
CL3777.Contig2_All	11.51076417	Up	PREDICTED: inositol monophosphatase 1-like [Strongylocentrotus purpuratus]
Unigene46997_All	11.51076417	Up	hypothetical protein ZC395.7 - Caenorhabditis elegans
Unigene58041_All	11.51076417	Up	conserved hypothetical protein [Capsaspora owczarzaki ATCC 30864] >gi|320164653|gb|EFW41552.1| conserved hypothetical protein [Capsaspora owczarzaki ATCC 30864]
Unigene53988_All	11.50769555	Up	PREDICTED: inositol-3-phosphate synthase 1-A-like [Amphimedon queenslandica]
CL3630.Contig1_All	11.50551386	Up	putative porphobilinogen deaminase [Danaus plexippus]
Unigene59443_All	11.50437211	Up	hypothetical protein DAPPUDRAFT_302108 [Daphnia pulex]
Unigene59624_All	11.50367669	Up	GK18624 [Drosophila willistoni] >gi|194171727|gb|EDW86628.1| GK18624 [Drosophila willistoni]
Unigene54088_All	11.50258321	Up	PREDICTED: gamma-enolase-like isoform 2 [Oryzias latipes]
Unigene50900_All	11.50143915	Up	defender against apopototic cell death 1 [Haliotis diversicolor]
Unigene43091_All	11.49625461	Up	rCG20683, isoform CRA_c [Rattus norvegicus]
Unigene59021_All	11.49490557	Up	PREDICTED: AMP deaminase 2 isoform 2 [Megachile rotundata]
Unigene41821_All	11.48834228	Up	hypothetical protein CAPTEDRAFT_20325 [Capitella teleta]
Unigene54825_All	11.48824184	Up	carbamoyl-phosphate synthase [Dichochrysa luctuosa]
CL8289.Contig1_All	11.48758885	Up	cytochrome p450 [Naegleria gruberi] >gi|284087785|gb|EFC41452.1| cytochrome p450 [Naegleria gruberi]
Unigene49459_All	11.48668421	Up	PREDICTED: si:dkey-256k13.1 [Danio rerio]
Unigene59148_All	11.48668421	Up	hypothetical protein [Monosiga brevicollis MX1] >gi|163770943|gb|EDQ84618.1| predicted protein [Monosiga brevicollis MX1]
CL1749.Contig2_All	11.48638254	Up	enoyl Coenzyme A hydratase [Capsaspora owczarzaki ATCC 30864] >gi|320164421|gb|EFW41320.1| enoyl Coenzyme A hydratase [Capsaspora owczarzaki ATCC 30864]

Unigene62536_All	11.4860808	Up	predicted protein [Nematostella vectensis] >gi|156218480|gb|EDO39377.1| predicted protein [Nematostella vectensis]
CL8988.Contig1_All	11.48316077	Up	leukotriene A4 hydrolase/aminopeptidase [Acanthamoeba castellanii str. Neff] >gi|440797661|gb|ELR18742.1| leukotriene A4 hydrolase/aminopeptidase [Acanthamoeba castellanii str. Neff]
CL3145.Contig1_All	11.47836516	Up	PREDICTED: ATP synthase subunit alpha, mitochondrial-like isoform 2 [Oryzias latipes]
CL4566.Contig1_All	11.47588539	Up	PREDICTED: cytochrome c1, heme protein, mitochondrial-like [Hydra magnipapillata]
CL7943.Contig1_All	11.47588539	Up	Cytosolic nonspecific dipeptidase, putative [Pediculus humanus corporis] >gi|212516890|gb|EEB18844.1| Cytosolic nonspecific dipeptidase, putative [Pediculus humanus corporis]
Unigene61737_All	11.47116713	Up	hypothetical protein DAPPUDRAFT_56427 [Daphnia pulex]
Unigene51122_All	11.47035383	Up	ribonucleotide reductase M2, gene 2 [Xenopus (Silurana) tropicalis] >gi|51258920|gb|AAH80161.1| ribonucleotide reductase M2 polypeptide [Xenopus (Silurana) tropicalis]
Unigene60577_All	11.47009958	Up	H+-ATPase B subunit, partial [Bos taurus]
Unigene41368_All	11.46964182	Up	PREDICTED: probable N-methylproline demethylase-like [Amphimedon queenslandica]
Unigene59136_All	11.46964182	Up	predicted protein [Nematostella vectensis] >gi|156209997|gb|EDO31211.1| predicted protein [Nematostella vectensis]
Unigene61168_All	11.4684204	Up	NADH dehydrogenase subunit 11 (mitochondrion) [Andalucia godoyi]
Unigene62228_All	11.46770743	Up	hypothetical protein PPL_02984 [Polysphondylium pallidum PN500]
Unigene60548_All	11.46199095	Up	carboxymethylenebutenolidase homolog [Xenopus (Silurana) tropicalis] >gi|82186538|sp|Q6P7K0.1|CMBL_XENTR RecName: Full=Carboxymethylenebutenolidase homolog >gi|38181938|gb|AAH61630.1| carboxymethylenebutenolidase homolog [Xenopus (Silurana) tropicalis] >gi|51950285|gb|AAH82501.1| carboxymethylenebutenolidase homolog [Xenopus (Silurana) tropicalis] >gi|89266784|emb|CAJ83783.1| similar to human flj23617 [Xenopus (Silurana) tropicalis]
CL1540.Contig1_All	11.46183752	Up	V-type H+-transporting ATPase subunit A [Clonorchis sinensis]
Unigene47909_All	11.46183752	Up	Kynu protein [Mus musculus]
Unigene58906_All	11.46173522	Up	Nucleoside diphosphate kinase [Harpegnathos saltator]
CL7901.Contig1_All	11.45999506	Up	PREDICTED: probable NADP-dependent mannitol dehydrogenase-like, partial [Acyrthosiphon pisum]
Unigene49827_All	11.45922668	Up	PREDICTED: squalene synthase-like [Amphimedon queenslandica]

CL3942.Contig2_All	11.45861167	Up	3-ketoacyl-CoA thiolase A, peroxisomal precursor, putative [Perkinsus marinus ATCC 50983] >gi|239890342|gb|EER12204.1| 3-ketoacyl-CoA thiolase A, peroxisomal precursor, putative [Perkinsus marinus ATCC 50983]
CL430.Contig2_All	11.45532722	Up	PREDICTED: NADH dehydrogenase [ubiquinone] flavoprotein 2, mitochondrial-like [Meleagris gallopavo]
Unigene50155_All	11.45450494	Up	GI10056 [Drosophila mojavensis] >gi|193916711|gb|EDW15578.1| GI10056 [Drosophila mojavensis]
Unigene48934_All	11.45234421	Up	carboxylesterase [Locusta migratoria manilensis]
Unigene58524_All	11.45136568	Up	C-4 methyl sterol oxidase [Dictyostelium discoideum AX4] >gi|60474307|gb|EAL72244.1| C-4 methyl sterol oxidase [Dictyostelium discoideum AX4]
Unigene41025_All	11.4482712	Up	predicted protein [Naegleria gruberi] >gi|284093522|gb|EFC47160.1| predicted protein [Naegleria gruberi]
CL4931.Contig2_All	11.44180298	Up	PREDICTED: cytochrome b-c1 complex subunit 7-like [Hydra magnipapillata]
Unigene42066_All	11.44154365	Up	hypothetical protein TRIADDRAFT_50124 [Trichoplax adhaerens] >gi|190585657|gb|EDV25725.1| hypothetical protein TRIADDRAFT_50124 [Trichoplax adhaerens]
CL6375.Contig1_All	11.44133615	Up	acylCoA synthetase [Acanthamoeba castellanii str. Neff] >gi|440797765|gb|ELR18841.1| acylCoA synthetase [Acanthamoeba castellanii str. Neff]
Unigene59201_All	11.44035012	Up	--
Unigene61305_All	11.43702378	Up	expressed hypothetical protein [Trichoplax adhaerens] >gi|190587959|gb|EDV28001.1| expressed hypothetical protein [Trichoplax adhaerens]
Unigene47947_All	11.43671154	Up	UDP-glucose 6-dehydrogenase [Aedes aegypti] >gi|108872470|gb|EAT36695.1| AAEL011242-PA [Aedes aegypti]
Unigene61075_All	11.43462823	Up	PREDICTED: retinal dehydrogenase 1 [Monodelphis domestica]
Unigene61928_All	11.43415907	Up	--
Unigene42063_All	11.43353328	Up	PREDICTED: similar to retinol dehydrogenase 11 [Tribolium castaneum] >gi|270013154|gb|EFA09602.1| hypothetical protein TcasGA2_TC011722 [Tribolium castaneum]
Unigene48388_All	11.43332463	Up	PREDICTED: cytochrome c oxidase assembly protein COX15 homolog [Strongylocentrotus purpuratus]
Unigene43305_All	11.43306377	Up	hypothetical protein DAPPUDRAFT_308098 [Daphnia pulex]
Unigene59198_All	11.43186319	Up	hypothetical protein [Monosiga brevicollis MX1] >gi|163772261|gb|EDQ85916.1| predicted protein [Monosiga brevicollis MX1]

Unigene50453_All	11.42715568	Up	hypothetical protein [Paramecium tetraurelia strain d4-2] >gi|124426047|emb|CAK90831.1| unnamed protein product [Paramecium tetraurelia]
CL4195.Contig1_All	11.42442876	Up	PREDICTED: alcohol dehydrogenase class-3-like [Takifugu rubripes]
Unigene58727_All	11.42411379	Up	isocitrate dehydrogenase [Clonorchis sinensis]
Unigene59442_All	11.41943355	Up	adenylate kinase [Theileria annulata]
Unigene62310_All	11.41669199	Up	UDP-glucose 4-epimerase [Osmerus mordax]
CL778.Contig1_All	11.41463239	Up	hypothetical protein BRAFLDRAFT_86500 [Branchiostoma floridae] >gi|229294759|gb|EEN65413.1| hypothetical protein BRAFLDRAFT_86500 [Branchiostoma floridae]
Unigene41218_All	11.41325769	Up	malate dehydrogenase [Crassostrea ariakensis]
CL7404.Contig1_All	11.41188168	Up	fumarylacetoacetase, putative [Ixodes scapularis] >gi|215502812|gb|EEC12306.1| fumarylacetoacetase, putative [Ixodes scapularis]
CL7195.Contig1_All	11.40822358	Up	PREDICTED: 3-isopropylmalate dehydratase large subunit-like [Bombus impatiens]
CL9458.Contig2_All	11.40726776	Up	4-aminobutyrate aminotransferase [Naegleria gruberi] >gi|284097104|gb|EFC50731.1| 4-aminobutyrate aminotransferase [Naegleria gruberi]
Unigene42193_All	11.4066302	Up	cytidine diphosphate-diacylglycerol synthase [Cryptosporidium hominis TU502] >gi|54655276|gb|EAL34763.1| cytidine diphosphate-diacylglycerol synthase [Cryptosporidium hominis]
Unigene62467_All	11.40098614	Up	predicted protein [Nematostella vectensis] >gi|156224063|gb|EDO44892.1| predicted protein [Nematostella vectensis]
Unigene46413_All	11.40061264	Up	alpha-amylase [Drosophila melanogaster]
Unigene60582_All	11.3984765	Up	hypothetical protein CAOG_03521 [Capsaspora owczarzaki ATCC 30864] >gi|320168638|gb|EFW45537.1| hypothetical protein CAOG_03521 [Capsaspora owczarzaki ATCC 30864]
Unigene53643_All	11.39703282	Up	putative long chain fatty Acyl CoA synthetase [Leishmania mexicana MHOM/GT/2001/U1103] >gi|322487913|emb|CBZ23157.1| putative long chain fatty Acyl CoA synthetase [Leishmania mexicana MHOM/GT/2001/U1103]
Unigene61868_All	11.39585541	Up	ATP:L-methionine S-Adenosyltransferase [Acanthamoeba castellanii str. Neff] >gi|440791419|gb|ELR12657.1| ATP:L-methionine S-Adenosyltransferase [Acanthamoeba

			castellanii str. Neff]
CL3505.Contig2_All	11.39473063	Up	H+-ATPase B subunit, partial [Bos taurus]
CL8187.Contig1_All	11.39387306	Up	hypothetical protein KGM_00281 [Danaus plexippus]
CL6466.Contig3_All	11.39274673	Up	fructose-1-6-bisphosphatase [Capsaspora owczarzaki ATCC 30864] >gi|320168762|gb|EFW45661.1| fructose-1-6-bisphosphatase [Capsaspora owczarzaki ATCC 30864]
Unigene60461_All	11.39156582	Up	4-hydroxyphenylpyruvate dioxygenase [Crassostrea gigas]
Unigene43895_All	11.39145842	Up	argininosuccinate synthase [Aedes aegypti] >gi|122115039|sp|Q0IFL5.1|ASSY_AEDAE RecName: Full=Argininosuccinate synthase; AltName: Full=Citrulline--aspartate ligase >gi|108879663|gb|EAT43888.1| AAEL004701-PA [Aedes aegypti]
Unigene50404_All	11.39086756	Up	succinate-CoA ligase [Dictyostelium fasciculatum] >gi|328875554|gb|EGG23918.1| succinate-CoA ligase [Dictyostelium fasciculatum]
Unigene61802_All	11.39054517	Up	Glutamine synthetase [Chelonia mydas]
Unigene65071_All	11.39054517	Up	Adenosine kinase 2 [Acromyrmex echinatior]
CL4532.Contig2_All	11.38914731	Up	acetyl-coenzyme A synthetase, putative [Perkinsus marinus ATCC 50983] >gi|239874724|gb|EER03060.1| acetyl-coenzyme A synthetase, putative [Perkinsus marinus ATCC 50983]
CL5096.Contig1_All	11.38521531	Up	DNA-directed RNA polymerase II subunit RPB11 [Salmo salar]
Unigene59059_All	11.38170516	Up	predicted protein [Naegleria gruberi] >gi|284097080|gb|EFC50707.1| predicted protein [Naegleria gruberi]
Unigene54383_All	11.38089392	Up	PREDICTED: magnesium transporter protein 1-like [Hydra magnipapillata]
Unigene53895_All	11.38040695	Up	glucokinase [Sparus aurata]
CL8798.Contig2_All	11.38035283	Up	PREDICTED: ATP synthase subunit O, mitochondrial-like [Takifugu rubripes]
Unigene46805_All	11.37759014	Up	alpha-amylase [Blattella germanica]
Unigene43016_All	11.37666806	Up	GH13244 [Drosophila grimshawi] >gi|193900196|gb|EDV99062.1| GH13244 [Drosophila grimshawi]
CL478.Contig1_All	11.37563681	Up	RecName: Full=Probable glycerol kinase; Short=GK; Short=Glycerokinase; AltName: Full=ATP:glycerol 3-phosphotransferase
CL5822.Contig1_All	11.37286506	Up	PREDICTED: xylulose kinase-like [Anolis carolinensis]
Unigene58786_All	11.37286506	Up	succinate-CoA ligase [Dictyostelium fasciculatum] >gi|328866700|gb|EGG15083.1| succinate-CoA ligase [Dictyostelium fasciculatum]

CL5417.Contig1_All	11.36730553	Up	ATP synthase subunit delta [Capsaspora owczarzaki ATCC 30864] >gi|320169944|gb|EFW46843.1| ATP synthase subunit delta [Capsaspora owczarzaki ATCC 30864]
CL3276.Contig1_All	11.36708708	Up	Phosphoserine phosphatase, putative [Perkinsus marinus ATCC 50983] >gi|239892114|gb|EER13235.1| Phosphoserine phosphatase, putative [Perkinsus marinus ATCC 50983]
Unigene61100_All	11.36331346	Up	PREDICTED: 6-phosphogluconolactonase-like [Saccoglossus kowalevskii]
Unigene51286_All	11.3628753	Up	reverse transcriptase [Liobuthus kessleri]
CL6097.Contig1_All	11.36216301	Up	Protein Y71H2AM.11 [Caenorhabditis elegans] >gi|373220612|emb|CCD73878.1| Protein Y71H2AM.11 [Caenorhabditis elegans]
Unigene52380_All	11.36095679	Up	dihydroorotase [Dictyostelium fasciculatum] >gi|328875482|gb|EGG23846.1| dihydroorotase [Dictyostelium fasciculatum]
Unigene61599_All	11.36013379	Up	coproporphyrinogen oxidase [Danio rerio]
Unigene63586_All	11.35667204	Up	short-chain dehydrogenase/reductase SDR [Capsaspora owczarzaki ATCC 30864] >gi|320162883|gb|EFW39782.1| short-chain dehydrogenase/reductase SDR [Capsaspora owczarzaki ATCC 30864]
Unigene60233_All	11.35093918	Up	malate synthase [Capsaspora owczarzaki ATCC 30864] >gi|320165110|gb|EFW42009.1| malate synthase [Capsaspora owczarzaki ATCC 30864]
CL1759.Contig2_All	11.34667993	Up	Isocitrate dehydrogenase [NADP] [Oxytricha trifallax]
Unigene42061_All	11.34568245	Up	hypothetical protein [Monosiga brevicollis MX1] >gi|163778508|gb|EDQ92123.1| predicted protein [Monosiga brevicollis MX1]
CL5386.Contig1_All	11.34457332	Up	hexokinase [Loa loa] >gi|307767876|gb|EFO27110.1| hexokinase [Loa loa]
Unigene62452_All	11.34290803	Up	PREDICTED: similar to AGAP011050-PA [Tribolium castaneum] >gi|270002564|gb|EEZ99011.1| hypothetical protein TcasGA2_TC004879 [Tribolium castaneum]
Unigene52027_All	11.33851356	Up	PREDICTED: dol-P-Man:Man(5)GlcNAc(2)-PP-Dol alpha-1,3-mannosyltransferase-like [Strongylocentrotus purpuratus]
Unigene64922_All	11.33382622	Up	PREDICTED: c-1-tetrahydrofolate synthase, cytoplasmic [Strongylocentrotus purpuratus]
Unigene64163_All	11.32805778	Up	homogentisate 1,2dioxygenase [Acanthamoeba castellanii str. Neff] >gi|440793959|gb|ELR15130.1| homogentisate 1,2dioxygenase [Acanthamoeba castellanii str. Neff]
CL8788.Contig2_All	11.32631712	Up	hypothetical protein UY3_09678 [Chelonia mydas]

Unigene54169_All	11.32220984	Up	PREDICTED: phosphatidylinositol-4-phosphate 3-kinase C2 domain-containing subunit beta-like, partial [Meleagris gallopavo]
Unigene62409_All	11.32170266	Up	PREDICTED: inositol-pentakisphosphate 2-kinase-like [Apis florea]
Unigene60272_All	11.32029287	Up	V-type proton ATPase subunit d [Acromyrmex echinatior]
Unigene62126_All	11.32006717	Up	squalene monooxygenase [Gallus gallus]
Unigene63081_All	11.31746914	Up	ferrochelatase, mitochondrial [Xenopus laevis] >gi|77748135|gb|AAI06397.1| LOC398014 protein [Xenopus laevis]
CL2263.Contig1_All	11.31486643	Up	adenosine kinase b [Salpingoeca sp. ATCC 50818]
Unigene62584_All	11.31282624	Up	Inositol3-phosphate synthase [Acanthamoeba castellanii str. Neff] >gi|440796106|gb|ELR17215.1| Inositol3-phosphate synthase [Acanthamoeba castellanii str. Neff]
Unigene41299_All	11.30993103	Up	Hypothetical protein CBG17698 [Caenorhabditis briggsae]
Unigene65302_All	11.30993103	Up	hypothetical protein PTSG_03958 [Salpingoeca sp. ATCC 50818]
Unigene41270_All	11.30805456	Up	transcriptional repressor TUP1, putative [Acanthamoeba castellanii str. Neff] >gi|440802037|gb|ELR22976.1| transcriptional repressor TUP1, putative [Acanthamoeba castellanii str. Neff]
Unigene51922_All	11.30799766	Up	acetyl-CoA synthetase [Culex quinquefasciatus] >gi|167880572|gb|EDS43955.1| acetyl-CoA synthetase [Culex quinquefasciatus]
Unigene43019_All	11.30628959	Up	PREDICTED: LOW QUALITY PROTEIN: 4-trimethylaminobutyraldehyde dehydrogenase [Ovis aries]
Unigene41223_All	11.3034383	Up	cytosolic non-specific dipeptidase [Rattus norvegicus] >gi|81892734|sp|Q6Q0N1.1|CNDP2_RAT RecName: Full=Cytosolic non-specific dipeptidase; AltName: Full=CNDP dipeptidase 2 >gi|45680894|gb|AAS75316.1| non-specific dipeptidase [Rattus norvegicus] >gi|63100240|gb|AAH95904.1| Cndp2 protein [Rattus norvegicus] >gi|149015878|gb|EDL75185.1| rCG20557, isoform CRA_b [Rattus norvegicus] >gi|149015879|gb|EDL75186.1| rCG20557, isoform CRA_b [Rattus norvegicus]
Unigene40876_All	11.30183911	Up	saccharopine dehydrogenase [Capsaspora owczarzaki ATCC 30864] >gi|320163854|gb|EFW40753.1| saccharopine dehydrogenase [Capsaspora owczarzaki ATCC 30864]
CL1540.Contig3_All	11.29777606	Up	putative vacuolar ATP synthase catalytic subunit A [Leishmania braziliensis MHOM/BR/75/M2904] >gi|134061641|emb|CAM38672.1| putative vacuolar ATP synthase catalytic

			subunit A [Leishmania braziliensis MHOM/BR/75/M2904]
Unigene41867_All	11.29777606	Up	hypothetical protein PTSG_12053 [Salpingoeca sp. ATCC 50818]
Unigene49117_All	11.29427611	Up	GL12387 [Drosophila persimilis] >gi|194115993|gb|EDW38036.1| GL12387 [Drosophila persimilis]
Unigene42100_All	11.29312674	Up	PREDICTED: UMP-CMP kinase-like [Anolis carolinensis]
CL9086.Contig1_All	11.29122827	Up	GI10243 [Drosophila mojavensis] >gi|193917059|gb|EDW15926.1| GI10243 [Drosophila mojavensis]
CL8287.Contig1_All	11.28794319	Up	predicted protein [Nematostella vectensis] >gi|156219968|gb|EDO40842.1| predicted protein [Nematostella vectensis]
Unigene50152_All	11.28759696	Up	phospholipase d1, putative [Ichthyophthirius multifiliis] >gi|340502022|gb|EGR28742.1| phospholipase d1, putative [Ichthyophthirius multifiliis]
Unigene41853_All	11.28719292	Up	mitochondrial citrate synthetase [Naegleria gruberi] >gi|284091752|gb|EFC45397.1| mitochondrial citrate synthetase [Naegleria gruberi]
CL5250.Contig3_All	11.28598011	Up	4nitrophenylphosphatase-like protein [Acanthamoeba castellanii str. Neff] >gi|440789564|gb|ELR10871.1| 4nitrophenylphosphatase-like protein [Acanthamoeba castellanii str. Neff]
Unigene52507_All	11.28337779	Up	hypothetical protein DICPUDRAFT_58919 [Dictyostelium purpureum] >gi|325075333|gb|EGC29234.1| hypothetical protein DICPUDRAFT_58919 [Dictyostelium purpureum]
CL8623.Contig2_All	11.28285676	Up	transaldolase family protein [Tetrahymena thermophila] >gi|89296648|gb|EAR94636.1| transaldolase family protein [Tetrahymena thermophila SB210]
Unigene63955_All	11.28268304	Up	acetyl-CoA synthetase [Salpingoeca sp. ATCC 50818]
Unigene48171_All	11.28088674	Up	hypothetical protein [Monosiga brevicollis MX1] >gi|163775093|gb|EDQ88718.1| predicted protein [Monosiga brevicollis MX1]
Unigene61625_All	11.27670602	Up	hypothetical protein TRIADDRAFT_56846 [Trichoplax adhaerens] >gi|190585105|gb|EDV25174.1| hypothetical protein TRIADDRAFT_56846 [Trichoplax adhaerens]
Unigene64040_All	11.27670602	Up	GF17037 [Drosophila ananassae] >gi|190626872|gb|EDV42396.1| GF17037 [Drosophila ananassae]
Unigene48877_All	11.27408691	Up	PREDICTED: ribose-phosphate pyrophosphokinase 1 isoform 2 [Gallus gallus] >gi|224098594|ref|XP_002187809.1| PREDICTED: ribose-phosphate pyrophosphokinase 1 isoform 1 [Taeniopygia guttata]
CL9217.Contig2_All	11.2713463	Up	PREDICTED: dolichol-phosphate mannosyltransferase subunit 3-like [Oreochromis niloticus]
Unigene42030_All	11.26848352	Up	hypothetical protein NCLIV_043880 [Neospora caninum Liverpool] >gi|325115769|emb|CBZ51324.1|

<table>
<tr><td></td><td></td><td></td><td>hypothetical protein NCLIV_043880 [Neospora caninum Liverpool]</td></tr>
<tr><td>Unigene45166_All</td><td>11.2675475</td><td>Up</td><td>diphosphomevalonate decarboxylase [Acanthamoeba castellanii str. Neff] >gi|440803951|gb|ELR24834.1| diphosphomevalonate decarboxylase [Acanthamoeba castellanii str. Neff]</td></tr>
<tr><td>Unigene41440_All</td><td>11.26631806</td><td>Up</td><td>hypothetical protein CAPTEDRAFT_107234 [Capitella teleta]</td></tr>
<tr><td>Unigene59184_All</td><td>11.2644426</td><td>Up</td><td>predicted protein [Nematostella vectensis] >gi|156219273|gb|EDO40157.1| predicted protein [Nematostella vectensis]</td></tr>
<tr><td>CL2497.Contig2_All</td><td>11.26362132</td><td>Up</td><td>uncharacterized protein LOC100216301 [Xenopus (Silurana) tropicalis]</td></tr>
<tr><td>Unigene64945_All</td><td>11.26232979</td><td>Up</td><td>hypothetical protein LOAG_08289 [Loa loa]</td></tr>
<tr><td>Unigene41152_All</td><td>11.26185986</td><td>Up</td><td>delta-aminolevulinic acid synthase [Chromera velia]</td></tr>
<tr><td>Unigene51658_All</td><td>11.26091953</td><td>Up</td><td>--</td></tr>
<tr><td>Unigene62554_All</td><td>11.26091953</td><td>Up</td><td>hypothetical protein [Monosiga brevicollis MX1] >gi|163775179|gb|EDQ88804.1| predicted protein [Monosiga brevicollis MX1]</td></tr>
<tr><td>Unigene64294_All</td><td>11.2593902</td><td>Up</td><td>hypothetical protein TRIADDRAFT_18436 [Trichoplax adhaerens] >gi|190589478|gb|EDV29500.1| hypothetical protein TRIADDRAFT_18436, partial [Trichoplax adhaerens]</td></tr>
<tr><td>Unigene48289_All</td><td>11.25750571</td><td>Up</td><td>PREDICTED: bifunctional coenzyme A synthase-like [Monodelphis domestica]</td></tr>
<tr><td>CL1847.Contig2_All</td><td>11.25496954</td><td>Up</td><td>hypothetical protein DICPUDRAFT_48737 [Dictyostelium purpureum] >gi|325080264|gb|EGC33827.1| hypothetical protein DICPUDRAFT_48737 [Dictyostelium purpureum]</td></tr>
<tr><td>Unigene61501_All</td><td>11.25378841</td><td>Up</td><td>2,3-bisphosphoglycerate-independent phosphoglycerate mutase [Wuchereria bancrofti]</td></tr>
<tr><td>Unigene59685_All</td><td>11.25177826</td><td>Up</td><td>aldehyde reductase [Salpingoeca sp. ATCC 50818]</td></tr>
<tr><td>Unigene64729_All</td><td>11.25059451</td><td>Up</td><td>PREDICTED: 6-phosphogluconolactonase-like, partial [Anolis carolinensis]</td></tr>
<tr><td>Unigene46634_All</td><td>11.2500615</td><td>Up</td><td>V-type ATPase, C subunit family protein [Tetrahymena thermophila] >gi|89295681|gb|EAR93669.1| V-type ATPase, C subunit family protein [Tetrahymena thermophila SB210]</td></tr>
<tr><td>Unigene60948_All</td><td>11.24774954</td><td>Up</td><td>hydroxysteroid dehydrogenase 4 [Capsaspora owczarzaki ATCC 30864] >gi|320167528|gb|EFW44427.1| hydroxysteroid dehydrogenase 4 [Capsaspora owczarzaki ATCC 30864]</td></tr>
<tr><td>CL6466.Contig4_All</td><td>11.24709678</td><td>Up</td><td>hypothetical protein IMG5_030200 [Ichthyophthirius multifiliis] >gi|340508176|gb|EGR33939.1| hypothetical protein IMG5_030200 [Ichthyophthirius multifiliis]</td></tr>
<tr><td>CL6197.Contig2_All</td><td>11.24543386</td><td>Up</td><td>branched-chain amino acid aminotransferase [Capsaspora owczarzaki ATCC 30864] >gi|320163336|gb|EFW40235.1| branched-chain amino acid aminotransferase [Capsaspora</td></tr>
</table>

			owczarzaki ATCC 30864]
Unigene60824_All	11.24424489	Up	--
CL2098.Contig1_All	11.24400698	Up	phosphoesterase, PA-phosphatase related-family protein [Dictyostelium discoideum AX4] >gi|74865958|sp|Q8MXL9.2|Y5547_DICDI RecName: Full=PA-phosphatase related-family protein DDB_G0275547 >gi|60471568|gb|EAL69524.1| phosphoesterase, PA-phosphatase related-family protein [Dictyostelium discoideum AX4]
Unigene66793_All	11.24365004	Up	PREDICTED: 72 kDa inositol polyphosphate 5-phosphatase-like isoform 2 [Bombus impatiens]
Unigene60892_All	11.24228095	Up	hypothetical protein DAPPUDRAFT_300069 [Daphnia pulex]
CL4428.Contig1_All	11.23703066	Up	alcohol dehydrogenase [Acanthamoeba castellanii str. Neff] >gi|440798713|gb|ELR19780.1| alcohol dehydrogenase [Acanthamoeba castellanii str. Neff]
Unigene67597_All	11.23637303	Up	PREDICTED: N-acetyllactosaminide beta-1,3-N-acetylglucosaminyltransferase-like [Nasonia vitripennis]
Unigene60194_All	11.23547577	Up	Protein ACL-2, isoform a [Caenorhabditis elegans] >gi|3879558|emb|CAA98276.1| Protein ACL-2, isoform a [Caenorhabditis elegans]
Unigene63423_All	11.2346977	Up	PREDICTED: aldehyde oxidase-like [Bombus terrestris]
Unigene51915_All	11.232301	Up	carbamoyl-phosphate synthase [Dichochrysa luctuosa]
Unigene48624_All	11.23140121	Up	Medium-chain specific acyl-CoA dehydrogenase, putative [Perkinsus marinus ATCC 50983] >gi|239902533|gb|EER19323.1| Medium-chain specific acyl-CoA dehydrogenase, putative [Perkinsus marinus ATCC 50983]
Unigene62591_All	11.22996037	Up	steroid dehydrogenase [Dictyostelium fasciculatum] >gi|328864879|gb|EGG13265.1| steroid dehydrogenase [Dictyostelium fasciculatum]
Unigene64054_All	11.22929951	Up	PREDICTED: catalase HPII-like [Bombus impatiens]
Unigene44248_All	11.22875858	Up	RNA polymerase II largest subunit [Acanthamoeba castellanii]
CL8293.Contig2_All	11.22803702	Up	asparagine synthase (glutaminehydrolyzing), putative [Acanthamoeba castellanii str. Neff] >gi|440792442|gb|ELR13664.1| asparagine synthase (glutaminehydrolyzing), putative [Acanthamoeba castellanii str. Neff]
Unigene40945_All	11.22743544	Up	Indoleamine 2,3-dioxygenase 1 [Tupaia chinensis]
Unigene40949_All	11.22683362	Up	hypothetical protein [Monosiga brevicollis MX1] >gi|163776262|gb|EDQ89882.1| predicted protein [Monosiga brevicollis MX1]

Unigene65891_All	11.22104329	Up	poly(p)/ATP nad kinase [Capsaspora owczarzaki ATCC 30864] >gi|320169897|gb|EFW46796.1| poly(p)/ATP nad kinase [Capsaspora owczarzaki ATCC 30864]
Unigene67925_All	11.22104329	Up	--
Unigene61537_All	11.22068062	Up	PREDICTED: catechol O-methyltransferase domain-containing protein 1-like [Takifugu rubripes]
Unigene60957_All	11.21529031	Up	predicted protein [Nematostella vectensis] >gi|156215160|gb|EDO36127.1| predicted protein [Nematostella vectensis]
Unigene69985_All	11.21529031	Up	catalase [Acanthamoeba castellanii str. Neff] >gi|372290526|gb|AEX91750.1| catalase 2 [Acanthamoeba castellanii] >gi|440792545|gb|ELR13756.1| catalase [Acanthamoeba castellanii str. Neff]
CL729.Contig1_All	11.21273955	Up	CDP-diacylglycerol--inositol 3-phosphatidyltransferase [Myotis davidii]
CL8258.Contig1_All	11.21188829	Up	alpha/beta hydrolase fold-1 domain-containing protein [Polysphondylium pallidum PN500]
Unigene61891_All	11.21152332	Up	Zgc:101757 protein [Danio rerio] >gi|197247074|gb|AAI65161.1| Zgc:101757 protein [Danio rerio]
Unigene63761_All	11.20463227	Up	GH13324 [Drosophila grimshawi] >gi|193900077|gb|EDV98943.1| GH13324 [Drosophila grimshawi]
Unigene59808_All	11.20426546	Up	alcohol dehydrogenase [Entamoeba dispar SAW760]
Unigene42436_All	11.2025524	Up	hypothetical protein CAPTEDRAFT_148541 [Capitella teleta]
Unigene59360_All	11.20132755	Up	PREDICTED: adenylosuccinate synthetase isozyme 1-like [Anolis carolinensis]
Unigene59710_All	11.19869059	Up	NAD-specific glutamate dehydrogenase [Capsaspora owczarzaki ATCC 30864] >gi|320170749|gb|EFW47648.1| NAD-specific glutamate dehydrogenase [Capsaspora owczarzaki ATCC 30864]
CL1046.Contig1_All	11.1945722	Up	PREDICTED: inosine-5'-monophosphate dehydrogenase 2 isoform 1 [Ciona intestinalis]
Unigene61260_All	11.1945722	Up	hypothetical protein [Monosiga brevicollis MX1] >gi|163776262|gb|EDQ89882.1| predicted protein [Monosiga brevicollis MX1]
CL2358.Contig2_All	11.19173783	Up	predicted protein [Naegleria gruberi] >gi|284093417|gb|EFC47055.1| predicted protein [Naegleria gruberi]
Unigene63978_All	11.18698083	Up	PREDICTED: NADH dehydrogenase [ubiquinone] 1 alpha subcomplex subunit 4-like [Oryzias latipes]
CL8788.Contig1_All	11.18468942	Up	PREDICTED: uncharacterized oxidoreductase ZK1290.5-like [Taeniopygia guttata]
CL5380.Contig1_All	11.18456545	Up	cytochrome P450 sterol C-22 desaturase [Capsaspora owczarzaki ATCC 30864] >gi|320162726|gb|EFW39625.1| cytochrome P450 sterol C-22 desaturase [Capsaspora owczarzaki ATCC 30864]
CL7955.Contig1_All	11.18444148	Up	vacuolar H+ ATPase B subunit [Dictyostelium purpureum] >gi|325078041|gb|EGC31715.1| vacuolar H+

			ATPase B subunit [Dictyostelium purpureum]
Unigene62474_All	11.17934932	Up	long-chain-fatty-acid--CoA ligase ACSBG2 [Gallus gallus]
Unigene63368_All	11.17579902	Up	PREDICTED: phosphopantothenoylcysteine decarboxylase-like [Oryzias latipes]
Unigene67703_All	11.17411425	Up	alpha methyl dopa-resistant, isoform A [Drosophila melanogaster] >gi|17380407|sp|P18486.2|L2AM_DROME RecName: Full=Alpha-methyldopa hypersensitive protein >gi|7298541|gb|AAF53760.1| alpha methyl dopa-resistant, isoform A [Drosophila melanogaster] >gi|374858090|gb|AEZ68802.1| FI18657p1 [Drosophila melanogaster]
CL7532.Contig1_All	11.17373959	Up	delta1-pyrroline-5-carboxylate dehydrogenase [Leishmania amazonensis]
Unigene65907_All	11.17348976	Up	PREDICTED: probable C-5 sterol desaturase-like [Metaseiulus occidentalis]
Unigene41835_All	11.17317742	Up	PREDICTED: dihydroxy-acid dehydratase-like [Amphimedon queenslandica]
Unigene66843_All	11.17280251	Up	aminomethyltransferase [Aedes aegypti] >gi|108873536|gb|EAT37761.1| AAEL010276-PA [Aedes aegypti]
CL9237.Contig2_All	11.17261502	Up	short-chain dehydrogenase/reductase SDR [Capsaspora owczarzaki ATCC 30864] >gi|320162883|gb|EFW39782.1| short-chain dehydrogenase/reductase SDR [Capsaspora owczarzaki ATCC 30864]
Unigene65279_All	11.17223997	Up	PREDICTED: branched-chain-amino-acid aminotransferase-like [Nasonia vitripennis]
Unigene62700_All	11.16842141	Up	PREDICTED: serine dehydratase-like [Saccoglossus kowalevskii]
CL2751.Contig2_All	11.16283188	Up	putative enolase protein, partial [Evergestis funalis]
CL7153.Contig1_All	11.16283188	Up	trifunctional enzyme subunit alpha [Salpingoeca sp. ATCC 50818]
Unigene48875_All	11.16283188	Up	Peroxisomalcoenzyme A synthetase [Acanthamoeba castellanii str. Neff] >gi|440792564|gb|ELR13773.1| Peroxisomalcoenzyme A synthetase [Acanthamoeba castellanii str. Neff]
Unigene52816_All	11.16283188	Up	NADP-dependent malate dehydrogenase [Polysphondylium pallidum PN500]
Unigene47759_All	11.16182471	Up	molybdopterin binding domain-containing protein [Capsaspora owczarzaki ATCC 30864] >gi|320167615|gb|EFW44514.1| molybdopterin binding domain-containing protein [Capsaspora owczarzaki ATCC 30864]
Unigene68402_All	11.16106888	Up	transketolase [Acanthamoeba castellanii str. Neff] >gi|440804204|gb|ELR25081.1| transketolase [Acanthamoeba castellanii str. Neff]
Unigene48673_All	11.15917757	Up	PREDICTED: glycine dehydrogenase [decarboxylating], mitochondrial-like [Ciona intestinalis]

Unigene52865_All	11.15564046	Up	hypothetical protein [Paramecium tetraurelia strain d4-2] >gi|124410190|emb|CAK75432.1| unnamed protein product [Paramecium tetraurelia]
Unigene42573_All	11.15367869	Up	5'-methylthioadenosine phosphorylase [Naegleria gruberi] >gi|284095312|gb|EFC48943.1| 5'-methylthioadenosine phosphorylase [Naegleria gruberi]
Unigene48191_All	11.15323534	Up	succinyl-CoA ligase alpha subunit, putative [Toxoplasma gondii ME49] >gi|211966118|gb|EEB01314.1| succinyl-CoA ligase alpha subunit, putative [Toxoplasma gondii ME49] >gi|221484277|gb|EEE22573.1| succinyl-CoA ligase alpha subunit, putative [Toxoplasma gondii GT1] >gi|221505743|gb|EEE31388.1| succinyl-CoA ligase alpha subunit, putative [Toxoplasma gondii VEG]
Unigene44883_All	11.15260174	Up	PREDICTED: glucose dehydrogenase [acceptor]-like [Acyrthosiphon pisum]
Unigene49037_All	11.15158741	Up	Mecr protein, partial [Danio rerio]
Unigene40770_All	11.15019154	Up	cystathionine beta synthase [Naegleria gruberi] >gi|284092282|gb|EFC45925.1| cystathionine beta synthase [Naegleria gruberi]
Unigene44419_All	11.14962012	Up	AGAP006654-PA [Anopheles gambiae str. PEST] >gi|157016419|gb|EAA10767.4| AGAP006654-PA [Anopheles gambiae str. PEST]
Unigene65203_All	11.14936608	Up	PREDICTED: uroporphyrinogen decarboxylase-like [Amphimedon queenslandica]
CL7797.Contig1_All	11.14822234	Up	PREDICTED: 6-phosphogluconate dehydrogenase, decarboxylating [Amphimedon queenslandica]
CL5743.Contig3_All	11.14644139	Up	PREDICTED: peroxisomal multifunctional enzyme type 2-like, partial [Oryzias latipes]
Unigene62828_All	11.14363831	Up	PREDICTED: probable methylmalonate-semialdehyde dehydrogenase [acylating], mitochondrial-like [Acyrthosiphon pisum]
Unigene43260_All	11.14038245	Up	malate synthase [Dictyostelium fasciculatum] >gi|328873562|gb|EGG21929.1| malate synthase [Dictyostelium fasciculatum]
Unigene71726_All	11.14031854	Up	Chain B, Orientation Of Rna Polymerase Ii Within The Human Vp16-Mediator-Pol Ii-Tfiif Assembly
Unigene40379_All	11.13916761	Up	catalase [Acanthamoeba castellanii str. Neff] >gi|372290526|gb|AEX91750.1| catalase 2 [Acanthamoeba castellanii] >gi|440792545|gb|ELR13756.1| catalase [Acanthamoeba castellanii str. Neff]
CL6650.Contig1_All	11.1385278	Up	catalase [Acanthamoeba castellanii str. Neff] >gi|372290526|gb|AEX91750.1| catalase 2 [Acanthamoeba castellanii] >gi|440792545|gb|ELR13756.1| catalase [Acanthamoeba castellanii str. Neff]
Unigene42593_All	11.13731139	Up	pyrroline carboxylate reductase, putative [Perkinsus marinus ATCC 50983] >gi|239868611|gb|EEQ99879.1| pyrroline carboxylate reductase, putative [Perkinsus marinus

			ATCC 50983]
Unigene58847_All	11.13731139	Up	predicted protein [Naegleria gruberi] >gi|284091150|gb|EFC44798.1| predicted protein [Naegleria gruberi]
Unigene62596_All	11.13731139	Up	putative fatty acyl CoA synthetase 2 [Leishmania major strain Friedlin] >gi|321438103|emb|CBZ11855.1| putative fatty acyl CoA synthetase 2 [Leishmania major strain Friedlin]
Unigene67376_All	11.13731139	Up	3-oxoacyl-(acyl-carrier protein) reductase,putative [Leishmania mexicana MHOM/GT/2001/U1103] >gi|322492261|emb|CBZ27535.1| 3-oxoacyl-(acyl-carrier protein) reductase,putative [Leishmania mexicana MHOM/GT/2001/U1103]
Unigene45482_All	11.13513209	Up	PREDICTED: DNA directed RNA polymerase II polypeptide G-like [Saccoglossus kowalevskii]
Unigene43158_All	11.1344905	Up	GI23155 [Drosophila mojavensis] >gi|193915876|gb|EDW14743.1| GI23155 [Drosophila mojavensis]
Unigene64422_All	11.13147116	Up	PREDICTED: pyruvate carboxylase 1-like, partial [Hydra magnipapillata]
Unigene52637_All	11.13044186	Up	hypothetical protein CAPTEDRAFT_157230 [Capitella teleta]
Unigene70056_All	11.13044186	Up	oxidoreductase, putative [Perkinsus marinus ATCC 50983] >gi|239896968|gb|EER15777.1| oxidoreductase, putative [Perkinsus marinus ATCC 50983]
CL1918.Contig3_All	11.12947622	Up	NADH dehydrogenase, putative [Ixodes scapularis] >gi|215509289|gb|EEC18742.1| NADH dehydrogenase, putative [Ixodes scapularis]
Unigene62881_All	11.12889653	Up	aminopeptidase, putative [Trypanosoma brucei gambiense DAL972]
Unigene63023_All	11.12825215	Up	PREDICTED: DNA (cytosine-5)-methyltransferase 3B-like [Bombus impatiens]
CL6694.Contig1_All	11.12786539	Up	vacuolar ATP synthase proteolipid subunit [Trichinella spiralis] >gi|316979348|gb|EFV62153.1| vacuolar ATP synthase proteolipid subunit [Trichinella spiralis]
CL7732.Contig1_All	11.12741403	Up	4-aminobutyrate transaminase [Dictyostelium discoideum AX4] >gi|74897469|sp|Q55FI1.1|GABT_DICDI RecName: Full=4-aminobutyrate aminotransferase; AltName: Full=GABA aminotransferase; Short=GABA-AT; AltName: Full=Gamma-amino-N-butyrate transaminase; Short=GABA transaminase >gi|60475570|gb|EAL73505.1| 4-aminobutyrate transaminase [Dictyostelium discoideum AX4]
CL3820.Contig1_All	11.12722055	Up	H+-ATPase B subunit, partial [Bos taurus]
CL1294.Contig2_All	11.1265109	Up	5'-methylthioadenosine phosphorylase [Naegleria gruberi] >gi|284095312|gb|EFC48943.1| 5'-methylthioadenosine phosphorylase [Naegleria gruberi]
CL4532.Contig1_All	11.12470293	Up	acetyl-coenzyme A synthetase, putative [Perkinsus marinus ATCC

<table>
<tr><td></td><td></td><td></td><td>50983] >gi|239891447|gb|EER12936.1| acetyl-coenzyme A synthetase, putative [Perkinsus marinus ATCC 50983]</td></tr>
<tr><td>Unigene65771_All</td><td>11.12470293</td><td>Up</td><td>unnamed protein product [Oikopleura dioica]</td></tr>
<tr><td>CL5438.Contig2_All</td><td>11.12127448</td><td>Up</td><td>dihydrolipoamide:NAD oxidoreductase [Dictyostelium purpureum] >gi|325084433|gb|EGC37861.1| dihydrolipoamide:NAD oxidoreductase [Dictyostelium purpureum]</td></tr>
<tr><td>Unigene66568_All</td><td>11.12088584</td><td>Up</td><td>S-adenosylmethionine decarboxylase, putative [Ixodes scapularis] >gi|215498825|gb|EEC08319.1| S-adenosylmethionine decarboxylase, putative [Ixodes scapularis]</td></tr>
<tr><td>Unigene43908_All</td><td>11.11991379</td><td>Up</td><td>uncharacterized protein LOC100216301 [Xenopus (Silurana) tropicalis]</td></tr>
<tr><td>Unigene63340_All</td><td>11.11952478</td><td>Up</td><td>PREDICTED: NADH dehydrogenase [ubiquinone] 1 alpha subcomplex subunit 11-like [Ailuropoda melanoleuca]</td></tr>
<tr><td>Unigene41934_All</td><td>11.11945993</td><td>Up</td><td>6-phosphogluconate dehydrogenase [Naegleria gruberi] >gi|284095088|gb|EFC48720.1| 6-phosphogluconate dehydrogenase [Naegleria gruberi]</td></tr>
<tr><td>Unigene69394_All</td><td>11.11673383</td><td>Up</td><td>voltage gated chloride channel domain-containing protein [Toxoplasma gondii ME49] >gi|211966343|gb|EEB01539.1| voltage gated chloride channel domain-containing protein [Toxoplasma gondii ME49]</td></tr>
<tr><td>Unigene46027_All</td><td>11.11621398</td><td>Up</td><td>PREDICTED: 2,3-bisphosphoglycerate-independent phosphoglycerate mutase-like [Strongylocentrotus purpuratus]</td></tr>
<tr><td>Unigene62437_All</td><td>11.11439305</td><td>Up</td><td>squalene monooxygenase [Polysphondylium pallidum PN500]</td></tr>
<tr><td>Unigene62713_All</td><td>11.11393746</td><td>Up</td><td>hypothetical protein [Monosiga brevicollis MX1] >gi|163778002|gb|EDQ91618.1| predicted protein [Monosiga brevicollis MX1]</td></tr>
<tr><td>CL2790.Contig1_All</td><td>11.11080953</td><td>Up</td><td>cysteine synthase [Acanthamoeba castellanii str. Neff] >gi|440802960|gb|ELR23874.1| cysteine synthase [Acanthamoeba castellanii str. Neff]</td></tr>
<tr><td>Unigene67276_All</td><td>11.10878579</td><td>Up</td><td>LAG1 longevity assurance-like protein 3, partial [Heterocephalus glaber]</td></tr>
<tr><td>Unigene62617_All</td><td>11.10368122</td><td>Up</td><td>hypothetical protein DAPPUDRAFT_299958 [Daphnia pulex]</td></tr>
<tr><td>Unigene41086_All</td><td>11.10322223</td><td>Up</td><td>PREDICTED: glycerol kinase-like, partial [Strongylocentrotus purpuratus]</td></tr>
<tr><td>CL5044.Contig2_All</td><td>11.1028287</td><td>Up</td><td>similarity to endo-1-like protein [Leishmania braziliensis MHOM/BR/75/M2904] >gi|134066644|emb|CAM44443.1| similarity to endo-1-like protein [Leishmania braziliensis MHOM/BR/75/M2904]</td></tr>
</table>

Unigene59450_All	11.09901899	Up	unnamed protein product [Tetraodon nigroviridis]
Unigene48826_All	11.09855852	Up	acetyl-CoA synthetase [Dictyostelium fasciculatum] >gi|328875333|gb|EGG23698.1| acetyl-CoA synthetase [Dictyostelium fasciculatum]
CL1987.Contig1_All	11.09776879	Up	Phosphoglucomutase-1 [Crassostrea gigas]
Unigene64198_All	11.09163373	Up	enoyl-CoA hydratase, partial [Schistocerca gregaria]
Unigene62728_All	11.09103862	Up	PREDICTED: 15-hydroxyprostaglandin dehydrogenase [NAD+]-like [Oreochromis niloticus]
Unigene63013_All	11.09064175	Up	carbamoyl-phosphate synthase large subunit [Capsaspora owczarzaki ATCC 30864] >gi|320164656|gb|EFW41555.1| carbamoyl-phosphate synthase large subunit [Capsaspora owczarzaki ATCC 30864]
Unigene64524_All	11.09024477	Up	putative acyl carrier protein [Leishmania mexicana MHOM/GT/2001/U1103] >gi|322492846|emb|CBZ28124.1| putative acyl carrier protein [Leishmania mexicana MHOM/GT/2001/U1103]
Unigene71703_All	11.08978149	Up	PREDICTED: 2-oxoisovalerate dehydrogenase subunit alpha, mitochondrial-like [Ciona intestinalis]
Unigene64223_All	11.08839075	Up	ATPase H+ transporting lysosomal V1 subunit C, partial [Branchiostoma belcheri]
Unigene51639_All	11.08308037	Up	unnamed protein product [Oikopleura dioica]
Unigene63229_All	11.07941811	Up	PREDICTED: 6-phosphofructokinase, muscle type isoform 2 [Felis catus]
Unigene44212_All	11.07881795	Up	PREDICTED: similar to glucosyl/glucuronosyl transferases [Tribolium castaneum] >gi|270013463|gb|EFA09911.1| hypothetical protein TcasGA2_TC012062 [Tribolium castaneum]
Unigene44961_All	11.07708274	Up	PREDICTED: hydroxymethylglutaryl-CoA lyase, mitochondrial-like [Bombus impatiens]
Unigene42910_All	11.0768156	Up	cytochrome p450 [Naegleria gruberi] >gi|284087785|gb|EFC41452.1| cytochrome p450 [Naegleria gruberi]
Unigene41159_All	11.0764816	Up	RNA polymerase II largest subunit [Reticulitermes speratus]
CL5177.Contig1_All	11.07534544	Up	unnamed protein product [Mus musculus]
Unigene65024_All	11.074476	Up	NADH:ubiquinone oxidoreductase, NDUFS8/23 kDa subunit, putative [Ixodes scapularis] >gi|215504281|gb|EEC13775.1| NADH:ubiquinone oxidoreductase, NDUFS8/23 kDa subunit, putative [Ixodes scapularis]
Unigene65249_All	11.074476	Up	predicted protein [Nematostella vectensis] >gi|156213599|gb|EDO34612.1| predicted protein

			[Nematostella vectensis]
Unigene63549_All	11.0732713	Up	NAD dependent epimerase/dehydratase family protein [Trichomonas vaginalis G3] >gi|121877533|gb|EAX84465.1| NAD dependent epimerase/dehydratase family protein [Trichomonas vaginalis G3]
Unigene52165_All	11.07293648	Up	phosphomannomutase [Dictyostelium fasciculatum] >gi|328875270|gb|EGG23635.1| phosphomannomutase [Dictyostelium fasciculatum]
Unigene42769_All	11.07233362	Up	PREDICTED: dihydroxy-acid dehydratase-like [Amphimedon queenslandica]
Unigene44376_All	11.0705235	Up	hypothetical protein SINV_11980 [Solenopsis invicta]
CL5784.Contig2_All	11.06985251	Up	branched chain keto acid dehydrogenase E1 [Capsaspora owczarzaki ATCC 30864] >gi|320167657|gb|EFW44556.1| branched chain keto acid dehydrogenase E1 [Capsaspora owczarzaki ATCC 30864]
Unigene63112_All	11.06958403	Up	IMP dehydrogenase [Dictyostelium purpureum] >gi|325082322|gb|EGC35807.1| IMP dehydrogenase [Dictyostelium purpureum]
Unigene67549_All	11.06958403	Up	poly(p)/ATP nad kinase [Salpingoeca sp. ATCC 50818]
Unigene41355_All	11.06931549	Up	geranylgeranyl diphosphate synthase 1, putative [Acanthamoeba castellanii str. Neff] >gi|440798190|gb|ELR19258.1| geranylgeranyl diphosphate synthase 1, putative [Acanthamoeba castellanii str. Neff]
Unigene62302_All	11.06864394	Up	PREDICTED: 5-hydroxyisourate hydrolase-like [Sus scrofa]
CL8289.Contig2_All	11.06635832	Up	cytochrome p450 [Naegleria gruberi] >gi|284087785|gb|EFC41452.1| cytochrome p450 [Naegleria gruberi]
Unigene60934_All	11.06332766	Up	PREDICTED: kynurenine 3-monooxygenase-like [Amphimedon queenslandica]
Unigene68897_All	11.06332766	Up	mitochondrial trans-2-enoyl-CoA reductase [Naegleria gruberi] >gi|284088044|gb|EFC41710.1| mitochondrial trans-2-enoyl-CoA reductase [Naegleria gruberi]
Unigene68021_All	11.05704405	Up	PREDICTED: D-aspartate oxidase-like [Strongylocentrotus purpuratus]
Unigene42821_All	11.05677317	Up	RNA polymerase I largest subunit [Ephydatia fluviatilis]
CL4259.Contig2_All	11.05609576	Up	nucleoside diphosphate kinase,related [Neospora caninum Liverpool] >gi|325114194|emb|CBZ49752.1| nucleoside diphosphate kinase,related [Neospora caninum Liverpool]
Unigene50272_All	11.05575693	Up	PREDICTED: LOW QUALITY PROTEIN: phosphatidylserine decarboxylase [Tursiops truncatus]

Unigene63595_All	11.05100496	Up	GA16231 [Drosophila pseudoobscura pseudoobscura] >gi|224493079|sp|Q290A8.2|KAD2_DROPS RecName: Full=Adenylate kinase 2, mitochondrial; Short=AK 2; AltName: Full=ATP-AMP transphosphorylase 2 >gi|198136195|gb|EAL25454.2| GA16231 [Drosophila pseudoobscura pseudoobscura]
Unigene48910_All	11.0502568	Up	Peroxisomalcoenzyme A synthetase [Acanthamoeba castellanii str. Neff] >gi|440792564|gb|ELR13773.1| Peroxisomalcoenzyme A synthetase [Acanthamoeba castellanii str. Neff]
Unigene66857_All	11.04610085	Up	PREDICTED: beta-1,3-galactosyltransferase 6-like [Monodelphis domestica]
Unigene44026_All	11.04152227	Up	heparan sulfate C5-epimerase, isoform A [Drosophila melanogaster] >gi|21645106|gb|AAM70806.1| heparan sulfate C5-epimerase, isoform A [Drosophila melanogaster]
CL6514.Contig1_All	11.0347302	Up	dihydrolipoamide S-succinyltransferase [Polysphondylium pallidum PN500]
Unigene52092_All	11.0347302	Up	Phospholipid methyltransferase [Acanthamoeba castellanii str. Neff] >gi|440791405|gb|ELR12643.1| Phospholipid methyltransferase [Acanthamoeba castellanii str. Neff]
Unigene43386_All	11.03390474	Up	PREDICTED: succinate-semialdehyde dehydrogenase, mitochondrial [Ciona intestinalis]
CL6584.Contig1_All	11.03356066	Up	carbamoyl-phosphate synthase large subunit [Capsaspora owczarzaki ATCC 30864] >gi|320164656|gb|EFW41555.1| carbamoyl-phosphate synthase large subunit [Capsaspora owczarzaki ATCC 30864]
CL7530.Contig1_All	11.03156337	Up	homogentisate 1,2dioxygenase [Acanthamoeba castellanii str. Neff] >gi|440793959|gb|ELR15130.1| homogentisate 1,2dioxygenase [Acanthamoeba castellanii str. Neff]
Unigene64968_All	11.03087401	Up	hypothetical protein BRAFLDRAFT_83616 [Branchiostoma floridae] >gi|229294991|gb|EEN65639.1| hypothetical protein BRAFLDRAFT_83616 [Branchiostoma floridae]
Unigene67015_All	11.03087401	Up	predicted protein [Nematostella vectensis] >gi|156228059|gb|EDO48859.1| predicted protein [Nematostella vectensis]
CL67.Contig2_All	11.03066714	Up	hypothetical protein BRAFLDRAFT_268929 [Branchiostoma floridae] >gi|229297136|gb|EEN67774.1| hypothetical protein BRAFLDRAFT_268929 [Branchiostoma floridae]
Unigene52867_All	11.0250011	Up	V-type proton ATPase subunit D [Crassostrea gigas]
CL5159.Contig2_All	11.02451638	Up	hypothetical protein SINV_01022 [Solenopsis invicta]
Unigene61502_All	11.02264523	Up	PREDICTED: glucosamine-6-phosphate isomerase 1-like [Ciona intestinalis]
CL2098.Contig2_All	11.02229845	Up	phosphoesterase, PA-phosphatase related-family protein [Dictyostelium discoideum

			AX4] >gi|74865958|sp|Q8MXL9.2|Y5547_DICDI RecName: Full=PA-phosphatase related-family protein DDB_G0275547 >gi|60471568|gb|EAL69524.1| phosphoesterase, PA-phosphatase related-family protein [Dictyostelium discoideum AX4]
Unigene46633_All	11.02222909	Up	V-type ATPase, C subunit family protein [Tetrahymena thermophila] >gi|89295681|gb|EAR93669.1| V-type ATPase, C subunit family protein [Tetrahymena thermophila SB210]
Unigene51637_All	11.02202097	Up	Serine hydroxymethyltransferase, mitochondrial [Dicentrarchus labrax]
Unigene42830_All	11.02104936	Up	malate synthase [Capsaspora owczarzaki ATCC 30864] >gi|320165110|gb|EFW42009.1| malate synthase [Capsaspora owczarzaki ATCC 30864]
Unigene64646_All	11.01826974	Up	NAD dependent epimerase/dehydratase family protein [Cryptosporidium muris RN66] >gi|209557458|gb|EEA07503.1| NAD dependent epimerase/dehydratase family protein [Cryptosporidium muris RN66]
Unigene71055_All	11.01178633	Up	pyruvate carboxylase [Capsaspora owczarzaki ATCC 30864] >gi|320168344|gb|EFW45243.1| pyruvate carboxylase [Capsaspora owczarzaki ATCC 30864]
Unigene61593_All	11.00828855	Up	PREDICTED: argininosuccinate lyase-like isoform 2 [Ciona intestinalis]
Unigene44550_All	11.00772811	Up	glutamate5-semialdehyde dehydrogenase [Acanthamoeba castellanii str. Neff] >gi|440804120|gb|ELR24998.1| glutamate5-semialdehyde dehydrogenase [Acanthamoeba castellanii str. Neff]
CL143.Contig2_All	11.00674683	Up	Succinyl-CoA ligase [GDP-forming] subunit alpha, mitochondrial [Camponotus floridanus]
CL6854.Contig1_All	11.00506308	Up	PREDICTED: 3-isopropylmalate dehydratase-like [Amphimedon queenslandica]
Unigene67410_All	11.00443117	Up	hypothetical protein DFA_07277 [Dictyostelium fasciculatum] >gi|328871787|gb|EGG20157.1| hypothetical protein DFA_07277 [Dictyostelium fasciculatum]
Unigene52583_All	11.00351791	Up	unnamed protein product [Oikopleura dioica]
Unigene70450_All	11.00274471	Up	Mannose-1-phosphate guanyltransferase alpha [Heterocephalus glaber]
CL8555.Contig2_All	10.99915442	Up	oligomycin sensitivity-conferring protein [Tribolium castaneum]
CL4507.Contig2_All	10.99725006	Up	RecName: Full=Lipoyl synthase, mitochondrial; AltName: Full=Lipoate synthase; Short=LS; Short=Lip-syn; AltName: Full=Lipoic acid synthase; Flags: Precursor
CL9746.Contig3_All	10.99293834	Up	diacylglycerol O-acyltransferase 2 [Dictyostelium fasciculatum] >gi|328865784|gb|EGG14170.1| diacylglycerol O-acyltransferase 2 [Dictyostelium fasciculatum]

Unigene63401_All	10.99293834	Up	cystathionine beta-lyase [Aedes aegypti] >gi|108880379|gb|EAT44604.1| AAEL004059-PA [Aedes aegypti]
CL8688.Contig1_All	10.98726401	Up	IgA-specific serine endopeptidase [Acanthamoeba castellanii str. Neff] >gi|440796239|gb|ELR17348.1| IgA-specific serine endopeptidase [Acanthamoeba castellanii str. Neff]
CL9967.Contig2_All	10.98605534	Up	predicted protein [Nematostella vectensis] >gi|156221516|gb|EDO42370.1| predicted protein [Nematostella vectensis]
Unigene41106_All	10.98491684	Up	alanine aminotransferase, mitochondrial, putative [Acanthamoeba castellanii str. Neff] >gi|440799227|gb|ELR20285.1| alanine aminotransferase, mitochondrial, putative [Acanthamoeba castellanii str. Neff]
CL282.Contig2_All	10.98484565	Up	hypothetical protein TRIADDRAFT_60142 [Trichoplax adhaerens] >gi|190581109|gb|EDV21187.1| hypothetical protein TRIADDRAFT_60142 [Trichoplax adhaerens]
Unigene63272_All	10.98484565	Up	PREDICTED: choline/ethanolamine kinase-like [Megachile rotundata]
CL5158.Contig1_All	10.98470327	Up	phosphatidylinositol synthase [Capsaspora owczarzaki ATCC 30864] >gi|320164590|gb|EFW41489.1| phosphatidylinositol synthase [Capsaspora owczarzaki ATCC 30864]
Unigene54385_All	10.98434725	Up	hypothetical protein CGI_10016135 [Crassostrea gigas]
Unigene52213_All	10.98377744	Up	V-type proton ATPase subunit E 2 [Pteropus alecto]
Unigene51010_All	10.98213797	Up	hypothetical protein BRAFLDRAFT_270056 [Branchiostoma floridae] >gi|229279940|gb|EEN50715.1| hypothetical protein BRAFLDRAFT_270056 [Branchiostoma floridae]
Unigene44094_All	10.98049664	Up	AGAP011994-PA [Anopheles gambiae str. PEST] >gi|116117098|gb|EAA00226.3| AGAP011994-PA [Anopheles gambiae str. PEST]
Unigene52632_All	10.97921081	Up	acetyl-CoA synthetase [Babesia bovis T2Bo]
CL5594.Contig1_All	10.97520312	Up	delta-aminolevulinate dehydratase [Dictyostelium discoideum AX4] >gi|122058183|sp|Q55E06.2|HEM2_DICDI RecName: Full=Delta-aminolevulinic acid dehydratase; Short=ALADH; AltName: Full=Porphobilinogen synthase >gi|90970863|gb|EAL72072.2| delta-aminolevulinate dehydratase [Dictyostelium discoideum AX4]
Unigene64341_All	10.97247727	Up	ferredoxin-glutamate synthase, putative [Ixodes scapularis] >gi|215498747|gb|EEC08241.1| ferredoxin-glutamate synthase, putative [Ixodes scapularis]
CL478.Contig2_All	10.97175908	Up	hypothetical protein DICPUDRAFT_34436 [Dictyostelium purpureum] >gi|325081249|gb|EGC34771.1|

			hypothetical protein DICPUDRAFT_34436 [Dictyostelium purpureum]
Unigene66716_All	10.97175908	Up	saccharopine dehydrogenase [Capsaspora owczarzaki ATCC 30864] >gi|320164266|gb|EFW41165.1| saccharopine dehydrogenase [Capsaspora owczarzaki ATCC 30864]
CL1214.Contig1_All	10.97017781	Up	PREDICTED: pyridoxine-5'-phosphate oxidase-like [Metaseiulus occidentalis]
Unigene42807_All	10.96816277	Up	predicted protein [Capsaspora owczarzaki ATCC 30864] >gi|320168126|gb|EFW45025.1| predicted protein [Capsaspora owczarzaki ATCC 30864]
Unigene72372_All	10.96780265	Up	PREDICTED: glutamate decarboxylase 1-like [Oryzias latipes]
Unigene65698_All	10.96758653	Up	hypothetical protein TcasGA2_TC000207 [Tribolium castaneum]
Unigene62757_All	10.96737038	Up	PREDICTED: ORMDL2-like [Saccoglossus kowalevskii]
Unigene66861_All	10.96737038	Up	GI13506 [Drosophila mojavensis] >gi|193920068|gb|EDW18935.1| GI13506 [Drosophila mojavensis]
CL5231.Contig2_All	10.96571215	Up	shortchain dehydrogenase/reductase SDR, putative [Acanthamoeba castellanii str. Neff] >gi|440794861|gb|ELR16006.1| shortchain dehydrogenase/reductase SDR, putative [Acanthamoeba castellanii str. Neff]
Unigene66986_All	10.96520709	Up	PREDICTED: glucosamine-6-phosphate isomerase 1-like [Ciona intestinalis]
Unigene67868_All	10.96123262	Up	fructose-1-6-bisphosphatase [Capsaspora owczarzaki ATCC 30864] >gi|320168762|gb|EFW45661.1| fructose-1-6-bisphosphatase [Capsaspora owczarzaki ATCC 30864]
CL8293.Contig3_All	10.95659396	Up	asparagine synthase (glutaminehydrolyzing), putative [Acanthamoeba castellanii str. Neff] >gi|440792442|gb|ELR13664.1| asparagine synthase (glutaminehydrolyzing), putative [Acanthamoeba castellanii str. Neff]
Unigene51826_All	10.95477793	Up	phosphatidylserine decarboxylase [Acanthamoeba castellanii str. Neff] >gi|440804563|gb|ELR25440.1| phosphatidylserine decarboxylase [Acanthamoeba castellanii str. Neff]
CL3539.Contig2_All	10.95419631	Up	hypothetical protein TRIADDRAFT_32492 [Trichoplax adhaerens] >gi|190579983|gb|EDV20070.1| hypothetical protein TRIADDRAFT_32492 [Trichoplax adhaerens]
Unigene44704_All	10.95368721	Up	hypothetical protein [Monosiga brevicollis MX1] >gi|163771694|gb|EDQ85356.1| predicted protein [Monosiga brevicollis MX1]
CL4049.Contig2_All	10.95230444	Up	PREDICTED: mitochondrial malate dehydrogenase [Oryctolagus cuniculus]
Unigene73257_All	10.9506288	Up	PREDICTED: nuclear elongation and deformation protein 1-like, partial [Mus musculus]
Unigene65348_All	10.94515117	Up	predicted protein [Nematostella vectensis] >gi|156212357|gb|EDO33420.1| predicted protein

			[Nematostella vectensis]
Unigene64819_All	10.94383344	Up	glucose-6-phosphate 1-dehydrogenase, putative [Trypanosoma cruzi]
Unigene69111_All	10.94339393	Up	2-methoxy-6-polyprenyl-1,4-benzoquinol methylase, mitochondrial precursor [Homo sapiens] >gi|90111987|sp|Q5HYK3.2|COQ5_HUMAN RecName: Full=2-methoxy-6-polyprenyl-1,4-benzoquinol methylase, mitochondrial; AltName: Full=Ubiquinone biosynthesis methyltransferase COQ5; Flags: Precursor >gi|81294347|gb|AAI07875.1| Coenzyme Q5 homolog, methyltransferase (S. cerevisiae) [Homo sapiens] >gi|119618604|gb|EAW98198.1| coenzyme Q5 homolog, methyltransferase (yeast), isoform CRA_a [Homo sapiens]
Unigene66318_All	10.94148783	Up	DAD family protein [Acanthamoeba castellanii str. Neff] >gi|440803227|gb|ELR24136.1| DAD family protein [Acanthamoeba castellanii str. Neff]
Unigene65690_All	10.94134111	Up	PREDICTED: 1-phosphatidylinositol-4,5-bisphosphate phosphodiesterase eta-1 [Gallus gallus]
Unigene64219_All	10.93994645	Up	4aminobutyrate aminotransferase [Acanthamoeba castellanii str. Neff] >gi|440792572|gb|ELR13781.1| 4aminobutyrate aminotransferase [Acanthamoeba castellanii str. Neff]
Unigene62855_All	10.93965267	Up	predicted protein [Nematostella vectensis] >gi|156217342|gb|EDO38261.1| predicted protein [Nematostella vectensis]
Unigene46108_All	10.93818286	Up	PREDICTED: catalase HPII-like [Bombus impatiens]
Unigene73117_All	10.93774162	Up	PREDICTED: serine hydroxymethyltransferase, cytosolic [Taeniopygia guttata]
Unigene71469_All	10.93722667	Up	PREDICTED: UDP-N-acetylhexosamine pyrophosphorylase-like [Anolis carolinensis]
Unigene64095_All	10.93707951	Up	hypothetical protein DDB_G0269848 [Dictyostelium discoideum AX4] >gi|60474337|gb|EAL72274.1| hypothetical protein DDB_G0269848 [Dictyostelium discoideum AX4]
Unigene66534_All	10.93206708	Up	pyrroline-5-carboxylate synthetase-like protein, putative [Trypanosoma cruzi marinkellei]
CL5697.Contig2_All	10.9315501	Up	carbamoyl-phosphate synthase large subunit [Capsaspora owczarzaki ATCC 30864] >gi|320164656|gb|EFW41555.1| carbamoyl-phosphate synthase large subunit [Capsaspora owczarzaki ATCC 30864]
CL6846.Contig3_All	10.9315501	Up	Fumarylacetoacetase [Crassostrea gigas]
Unigene44299_All	10.93103294	Up	Chain A, Orientation Of Rna Polymerase Ii Within The Human Vp16-Mediator-Pol Ii-Tfiif Assembly
Unigene67684_All	10.93014595	Up	CTP synthase 1 [Salmo salar]
Unigene41781_All	10.92896244	Up	PREDICTED: amine oxidase, copper containing 3 [Oryctolagus cuniculus]

Unigene64743_All	10.92577695	Up	aminotransferase, class V, putative [Acanthamoeba castellanii str. Neff] >gi|440797810|gb|ELR18885.1| aminotransferase, class V, putative [Acanthamoeba castellanii str. Neff]
Unigene65371_All	10.92577695	Up	RNA polymerase II largest subunit [Pseudobiantes japonicus]
Unigene68162_All	10.92577695	Up	alpha-esterase [Culex quinquefasciatus] >gi|167875740|gb|EDS39123.1| alpha-esterase [Culex quinquefasciatus]
Unigene70142_All	10.92577695	Up	V-ATPase subunit A4 [Paramecium tetraurelia] >gi|66863383|emb|CAH39846.1| V-ATPase subunit A3 [Paramecium tetraurelia]
CL6071.Contig2_All	10.92213837	Up	GMP synthetase, putative [Perkinsus marinus ATCC 50983] >gi|239868875|gb|EER00030.1| GMP synthetase, putative [Perkinsus marinus ATCC 50983]
Unigene62947_All	10.91923579	Up	Glutaryl-CoA dehydrogenase, mitochondrial [Acromyrmex echinatior]
Unigene48813_All	10.91893775	Up	PREDICTED: 3-isopropylmalate dehydratase-like [Amphimedon queenslandica]
CL2046.Contig3_All	10.91206599	Up	hypothetical protein [Pongo abelii]
Unigene70021_All	10.91206599	Up	PREDICTED: purine nucleoside phosphorylase-like [Oryzias latipes]
Unigene44748_All	10.91071767	Up	v-type ATPase 116kda subunit family protein, putative [Ichthyophthirius multifiliis] >gi|340504744|gb|EGR31162.1| v-type ATPase 116kda subunit family protein, putative [Ichthyophthirius multifiliis]
Unigene51090_All	10.91071767	Up	PREDICTED: similar to AGAP002711-PA [Tribolium castaneum]
Unigene64949_All	10.90906802	Up	PREDICTED: dolichyl-diphosphooligosaccharide--protein glycosyltransferase subunit STT3B-like [Oryzias latipes]
CL1714.Contig1_All	10.90794218	Up	seminal fluid protein CSSFP009 [Chilo suppressalis]
CL3942.Contig1_All	10.9077169	Up	3-ketoacyl-CoA thiolase A, peroxisomal precursor, putative [Perkinsus marinus ATCC 50983] >gi|239890342|gb|EER12204.1| 3-ketoacyl-CoA thiolase A, peroxisomal precursor, putative [Perkinsus marinus ATCC 50983]
Unigene62884_All	10.90719113	Up	hypothetical protein KGM_03341 [Danaus plexippus]
Unigene66122_All	10.90523656	Up	PREDICTED: NADH-ubiquinone oxidoreductase 75 kDa subunit, mitochondrial-like [Acyrthosiphon pisum]
Unigene42261_All	10.90410772	Up	Chain C, Orientation Of Rna Polymerase Ii Within The Human Vp16-Mediator-Pol Ii-Tfiif Assembly
CL1707.Contig1_All	10.89981005	Up	vacuolar ATPase B subunit [Salpingoeca sp. ATCC 50818]

<table>
<tr><td>CL727.Contig2_All</td><td>10.89905476</td><td>Up</td><td>6-phosphogluconate dehydrogenase, decarboxylating, partial [Chelonia mydas]</td></tr>
<tr><td>Unigene67731_All</td><td>10.89905476</td><td>Up</td><td>hypothetical protein BRAFLDRAFT_59069 [Branchiostoma floridae] >gi|229284419|gb|EEN55155.1| hypothetical protein BRAFLDRAFT_59069 [Branchiostoma floridae]</td></tr>
<tr><td>Unigene67474_All</td><td>10.89769422</td><td>Up</td><td>PREDICTED: methylmalonate-semialdehyde dehydrogenase [acylating], mitochondrial isoform 2 [Papio anubis]</td></tr>
<tr><td>Unigene67492_All</td><td>10.89466621</td><td>Up</td><td>mannitol dehydrogenase [Hyperamoeba sp. RFW-2006]</td></tr>
<tr><td>Unigene42557_All</td><td>10.89405985</td><td>Up</td><td>phosphoglycerate mutase [Gymnochlora stellata]</td></tr>
<tr><td>Unigene40790_All</td><td>10.89216331</td><td>Up</td><td>PREDICTED: hydroxysteroid (17-beta) dehydrogenase 4-like [Saccoglossus kowalevskii]</td></tr>
<tr><td>CL8364.Contig2_All</td><td>10.89170777</td><td>Up</td><td>PREDICTED: similar to coproporphirynogen oxidase [Tribolium castaneum]</td></tr>
<tr><td>Unigene62144_All</td><td>10.88699208</td><td>Up</td><td>alkaline phosphatase [Capsaspora owczarzaki ATCC 30864] >gi|320165694|gb|EFW42593.1| alkaline phosphatase [Capsaspora owczarzaki ATCC 30864]</td></tr>
<tr><td>Unigene65202_All</td><td>10.88699208</td><td>Up</td><td>hypothetical protein DFA_06886 [Dictyostelium fasciculatum] >gi|328871415|gb|EGG19785.1| hypothetical protein DFA_06886 [Dictyostelium fasciculatum]</td></tr>
<tr><td>Unigene69076_All</td><td>10.88699208</td><td>Up</td><td>predicted protein [Nematostella vectensis] >gi|156213339|gb|EDO34362.1| predicted protein [Nematostella vectensis]</td></tr>
<tr><td>Unigene70595_All</td><td>10.88699208</td><td>Up</td><td>V-type proton ATPase subunit H [Crassostrea gigas]</td></tr>
<tr><td>Unigene70509_All</td><td>10.88493365</td><td>Up</td><td>GF14111 [Drosophila ananassae] >gi|190616788|gb|EDV32312.1| GF14111 [Drosophila ananassae]</td></tr>
<tr><td>Unigene66112_All</td><td>10.88271946</td><td>Up</td><td>hypothetical protein DICPUDRAFT_48007 [Dictyostelium purpureum] >gi|325081384|gb|EGC34902.1| hypothetical protein DICPUDRAFT_48007 [Dictyostelium purpureum]</td></tr>
<tr><td>Unigene48018_All</td><td>10.88111396</td><td>Up</td><td>translation initiation factor eif-2b [Capsaspora owczarzaki ATCC 30864] >gi|320169618|gb|EFW46517.1| translation initiation factor eif-2b [Capsaspora owczarzaki ATCC 30864]</td></tr>
<tr><td>CL1289.Contig1_All</td><td>10.87428979</td><td>Up</td><td>PREDICTED: 6-phosphogluconolactonase-like [Strongylocentrotus purpuratus]</td></tr>
<tr><td>CL67.Contig1_All</td><td>10.87428979</td><td>Up</td><td>PREDICTED: acetyl-CoA acetyltransferase B, mitochondrial-like isoform 4 [Ciona intestinalis]</td></tr>
<tr><td>CL7153.Contig2_All</td><td>10.87428979</td><td>Up</td><td>trifunctional enzyme subunit alpha [Salpingoeca sp. ATCC 50818]</td></tr>
<tr><td>Unigene45235_All</td><td>10.87313647</td><td>Up</td><td>PREDICTED: LOW QUALITY PROTEIN: trifunctional purine biosynthetic protein adenosine-3-like [Metaseiulus occidentalis]</td></tr>
<tr><td>Unigene66740_All</td><td>10.87313647</td><td>Up</td><td>palmitoyl-protein thioesterase 1 [Polysphondylium pallidum PN500]</td></tr>
<tr><td>Unigene69554_All</td><td>10.86990224</td><td>Up</td><td>Pancreatic triacylglycerol lipase [Camponotus floridanus]</td></tr>
</table>

Unigene41635_All	10.86866825	Up	UTP--glucose-1-phosphate uridylyltransferase [Acromyrmex echinatior]
Unigene42028_All	10.86735597	Up	ethanolamine-phosphate cytidylyltransferase [Aedes aegypti] >gi|108878622|gb|EAT42847.1| AAEL005651-PA [Aedes aegypti]
Unigene70632_All	10.86689253	Up	PREDICTED: phosphoglycolate phosphatase-like [Oreochromis niloticus]
Unigene68174_All	10.86465046	Up	predicted protein [Nematostella vectensis] >gi|156227778|gb|EDO48580.1| predicted protein [Nematostella vectensis]
Unigene45668_All	10.86170729	Up	PREDICTED: leukotriene A-4 hydrolase [Ornithorhynchus anatinus]
Unigene52008_All	10.86170729	Up	urate oxidase [Polysphondylium pallidum PN500]
Unigene40961_All	10.85976771	Up	AAEL004178-PB [Aedes aegypti]
Unigene65793_All	10.85953479	Up	glycine dehydrogenase [Dictyostelium discoideum AX4] >gi|74853108|sp|Q54KM7.1|GCSP_DICDI RecName: Full=Glycine dehydrogenase [decarboxylating], mitochondrial; AltName: Full=Glycine cleavage system P protein; AltName: Full=Glycine decarboxylase; Flags: Precursor >gi|60465752|gb|EAL63829.1| glycine dehydrogenase [Dictyostelium discoideum AX4]
Unigene67291_All	10.85891347	Up	NAD+ kinase family protein [Polysphondylium pallidum PN500]
Unigene52302_All	10.85689234	Up	PREDICTED: proline dehydrogenase, mitochondrial-like, partial [Danio rerio]
Unigene64267_All	10.85689234	Up	PREDICTED: LOW QUALITY PROTEIN: 1-phosphatidylinositol-4,5-bisphosphate phosphodiesterase epsilon-1-like [Apis florea]
Unigene71777_All	10.85595856	Up	hypothetical protein [Monosiga brevicollis MX1] >gi|163776285|gb|EDQ89905.1| predicted protein [Monosiga brevicollis MX1]
Unigene62288_All	10.85517995	Up	PREDICTED: NADP-dependent malic enzyme, mitochondrial-like [Oreochromis niloticus]
Unigene41160_All	10.85330956	Up	Nicotinamide mononucleotide adenylyltransferase 1 [Lepeophtheirus salmonis]
Unigene69143_All	10.84830996	Up	--
Unigene44333_All	10.84658734	Up	PREDICTED: hydroxyacid oxidase 1-like [Saccoglossus kowalevskii]
Unigene44425_All	10.8460388	Up	GA16964 [Drosophila pseudoobscura pseudoobscura] >gi|198136211|gb|EAL25477.2| GA16964 [Drosophila pseudoobscura pseudoobscura]
Unigene54756_All	10.8460388	Up	GF22046 [Drosophila ananassae] >gi|190617107|gb|EDV32631.1| GF22046 [Drosophila ananassae]
Unigene42138_All	10.84462731	Up	orotidine 5'-monophosphate decarboxylase [Salpingoeca sp. ATCC 50818]
Unigene70437_All	10.84297883	Up	Dihydrolipoyl dehydrogenase [Oxytricha trifallax]

Unigene64928_All	10.84235034	Up	PREDICTED: glucosamine-6-phosphate isomerase 1 [Canis lupus familiaris]
Unigene65959_All	10.84093524	Up	malate synthase [Capsaspora owczarzaki ATCC 30864] >gi|320165110|gb|EFW42009.1| malate synthase [Capsaspora owczarzaki ATCC 30864]
Unigene40799_All	10.83676048	Up	aldehyde dehydrogenase [Dictyostelium discoideum AX4] >gi|60463966|gb|EAL62129.1| aldehyde dehydrogenase [Dictyostelium discoideum AX4]
Unigene49130_All	10.83344357	Up	dolichyl-diphosphooligosaccharide--protein glycotransferase [Branchiostoma belcheri tsingtauense]
Unigene44278_All	10.83225711	Up	V-type proton ATPase subunit C 1 [Xenopus (Silurana) tropicalis] >gi|82186435|sp|Q6P4Y9.1|VATC1_XENTR RecName: Full=V-type proton ATPase subunit C 1; Short=V-ATPase subunit C 1; AltName: Full=Vacuolar proton pump subunit C 1 >gi|38649298|gb|AAH63194.1| ATPase, H+ transporting, lysosomal V1 subunit C1 [Xenopus (Silurana) tropicalis] >gi|89269810|emb|CAJ81546.1| ATPase, H+ transporting, lysosomal, V1 subunit C, isoform 1 [Xenopus (Silurana) tropicalis]
Unigene42343_All	10.82916775	Up	hypothetical protein 2 - cabbage looper transposon TED (fragment)
Unigene43960_All	10.82885051	Up	RecName: Full=Phosphoglycerate kinase >gi|2109465|gb|AAB58242.1| phosphoglycerate kinase [Glaucoma chattoni]
Unigene65329_All	10.82885051	Up	PREDICTED: hypoxanthine-guanine phosphoribosyltransferase-like [Felis catus]
CL5822.Contig2_All	10.82829519	Up	hypothetical protein CAPTEDRAFT_218878 [Capitella teleta]
CL6158.Contig1_All	10.82766027	Up	Protein GLN-3, isoform b [Caenorhabditis elegans] >gi|5824686|emb|CAB54376.1| Protein GLN-3, isoform b [Caenorhabditis elegans]
CL7588.Contig1_All	10.82646904	Up	hypothetical protein BRAFLDRAFT_125492 [Branchiostoma floridae] >gi|229293673|gb|EEN64332.1| hypothetical protein BRAFLDRAFT_125492 [Branchiostoma floridae]
Unigene62726_All	10.82646904	Up	V-type proton ATPase subunit H [Crassostrea gigas]
Unigene68932_All	10.82583332	Up	PREDICTED: non-specific lipid-transfer protein-like, partial [Ornithorhynchus anatinus]
Unigene41455_All	10.82551535	Up	PREDICTED: dTDP-D-glucose 4,6-dehydratase-like [Meleagris gallopavo]
CL2820.Contig1_All	10.82448147	Up	Amiloride-sensitive amine oxidase [copper-containing] [Crassostrea gigas]
Unigene66217_All	10.82448147	Up	GA21870 [Drosophila pseudoobscura pseudoobscura] >gi|195156157|ref|XP_002018967.1| GL26099 [Drosophila persimilis] >gi|54645621|gb|EAL34359.1| GA21870 [Drosophila pseudoobscura pseudoobscura] >gi|194115120|gb|EDW37163.1| GL26099 [Drosophila persimilis]

CL2402.Contig1_All	10.82273015	Up	long-chain-fatty-acid--CoA ligase, putative [Entamoeba invadens IP1] >gi|440302662|gb|ELP94969.1| long-chain-fatty-acid--CoA ligase, putative [Entamoeba invadens IP1]
CL7418.Contig2_All	10.82193339	Up	adenosylmethionine decarboxylase [Acanthamoeba castellanii str. Neff] >gi|440794734|gb|ELR15889.1| adenosylmethionine decarboxylase [Acanthamoeba castellanii str. Neff]
Unigene41103_All	10.81962029	Up	unnamed protein product [Oikopleura dioica]
Unigene52329_All	10.81938079	Up	PREDICTED: short/branched chain specific acyl-CoA dehydrogenase, mitochondrial [Sus scrofa]
CL430.Contig1_All	10.8184224	Up	PREDICTED: NADH dehydrogenase [ubiquinone] flavoprotein 2, mitochondrial isoform 2 [Gallus gallus]
CL7404.Contig2_All	10.81754332	Up	fumarylacetoacetate hydrolase [Aedes aegypti] >gi|108871677|gb|EAT35902.1| AAEL011973-PA [Aedes aegypti]
Unigene71519_All	10.81450236	Up	diphosphomevalonate decarboxylase [Salpingoeca sp. ATCC 50818]
Unigene69132_All	10.81370104	Up	conserved hypothetical protein [Ixodes scapularis] >gi|215509843|gb|EEC19296.1| conserved hypothetical protein [Ixodes scapularis]
Unigene64865_All	10.80952699	Up	V-ATPase subunit C family protein [Loa loa] >gi|307765316|gb|EFO24550.1| V-type proton ATPase subunit C [Loa loa]
Unigene42563_All	10.80735492	Up	acetyl-CoenzymeA synthetase (acetate--coa ligase) (acyl-activating enzyme) [Cryptosporidium hominis TU502] >gi|54656759|gb|EAL35665.1| acetyl-CoenzymeA synthetase (acetate--coa ligase) (acyl-activating enzyme) [Cryptosporidium hominis]
Unigene67134_All	10.80663017	Up	GPI-anchor transamidase precursor, putative [Perkinsus marinus ATCC 50983] >gi|239899795|gb|EER17543.1| GPI-anchor transamidase precursor, putative [Perkinsus marinus ATCC 50983]
Unigene66917_All	10.80574387	Up	PREDICTED: 3-oxoacyl-[acyl-carrier-protein] reductase-like [Xenopus (Silurana) tropicalis]
CL5796.Contig1_All	10.80025281	Up	PREDICTED: mannose-6-phosphate isomerase-like [Strongylocentrotus purpuratus]
Unigene62936_All	10.80025281	Up	acetyl-CoA acetyltransferase [Trichinella spiralis] >gi|316976642|gb|EFV59889.1| acetyl-CoA acetyltransferase [Trichinella spiralis]
Unigene71109_All	10.80025281	Up	glyceraldehyde-3-phosphate dehydrogenase [Brachionus plicatilis]
Unigene73958_All	10.80025281	Up	serine 3-dehydrogenase [Acanthamoeba castellanii str. Neff] >gi|440794246|gb|ELR15413.1| serine 3-dehydrogenase [Acanthamoeba castellanii str. Neff]

<table>
<tr><td>Unigene41457_All</td><td>10.79579616</td><td>Up</td><td>predicted protein [Naegleria gruberi] >gi|284092320|gb|EFC45962.1| predicted protein [Naegleria gruberi]</td></tr>
<tr><td>Unigene45157_All</td><td>10.79530915</td><td>Up</td><td>THAP domain-containing protein 2 [Tupaia chinensis]</td></tr>
<tr><td>Unigene41590_All</td><td>10.79482197</td><td>Up</td><td>branched-chain amino acid aminotransferase [Capsaspora owczarzaki ATCC 30864] >gi|320163336|gb|EFW40235.1| branched-chain amino acid aminotransferase [Capsaspora owczarzaki ATCC 30864]</td></tr>
<tr><td>Unigene65962_All</td><td>10.79482197</td><td>Up</td><td>tripsin I-P1 precursor, putative [Pediculus humanus corporis] >gi|212511976|gb|EEB14823.1| tripsin I-P1 precursor, putative [Pediculus humanus corporis]</td></tr>
<tr><td>Unigene44267_All</td><td>10.79319686</td><td>Up</td><td>estradiol 17-beta-dehydrogenase 12 [Salpingoeca sp. ATCC 50818]</td></tr>
<tr><td>Unigene44386_All</td><td>10.79319686</td><td>Up</td><td>hypothetical protein CAEBREN_24389 [Caenorhabditis brenneri]</td></tr>
<tr><td>CL1218.Contig1_All</td><td>10.79279029</td><td>Up</td><td>PREDICTED: uncharacterized short-chain type dehydrogenase/reductase y4vI-like [Xenopus (Silurana) tropicalis]</td></tr>
<tr><td>Unigene42558_All</td><td>10.79108147</td><td>Up</td><td>Acyl-CoA dehydrogenase, putative [Perkinsus marinus ATCC 50983] >gi|239895928|gb|EER15269.1| Acyl-CoA dehydrogenase, putative [Perkinsus marinus ATCC 50983]</td></tr>
<tr><td>Unigene70504_All</td><td>10.79108147</td><td>Up</td><td>phosphofructokinase, partial [Polymastia tenax]</td></tr>
<tr><td>Unigene45993_All</td><td>10.7883105</td><td>Up</td><td>PREDICTED: sphingosine kinase 2-like [Oryzias latipes]</td></tr>
<tr><td>Unigene68341_All</td><td>10.7883105</td><td>Up</td><td>adenylosuccinate lyase [Sus scrofa]</td></tr>
<tr><td>CL482.Contig1_All</td><td>10.78700468</td><td>Up</td><td>Transposable element Tcb1 transposase [Salmo salar]</td></tr>
<tr><td>CL5250.Contig2_All</td><td>10.78398041</td><td>Up</td><td>4nitrophenylphosphatase-like protein [Acanthamoeba castellanii str. Neff] >gi|440789564|gb|ELR10871.1| 4nitrophenylphosphatase-like protein [Acanthamoeba castellanii str. Neff]</td></tr>
<tr><td>Unigene63677_All</td><td>10.78234303</td><td>Up</td><td>Non-specific lipid-transfer protein [Acanthamoeba castellanii str. Neff] >gi|440791513|gb|ELR12751.1| Non-specific lipid-transfer protein [Acanthamoeba castellanii str. Neff]</td></tr>
<tr><td>Unigene51701_All</td><td>10.78168756</td><td>Up</td><td>crotonase [Salpingoeca sp. ATCC 50818]</td></tr>
<tr><td>Unigene70592_All</td><td>10.77733752</td><td>Up</td><td>PREDICTED: succinate-semialdehyde dehydrogenase, mitochondrial-like, partial [Cricetulus griseus]</td></tr>
<tr><td>Unigene71435_All</td><td>10.77643303</td><td>Up</td><td>cholinephosphate cytidylyltransferase [Dictyostelium discoideum AX4] >gi|60464176|gb|EAL62336.1| cholinephosphate cytidylyltransferase [Dictyostelium discoideum AX4]</td></tr>
<tr><td>Unigene67166_All</td><td>10.77379857</td><td>Up</td><td>predicted protein [Nematostella vectensis] >gi|156222143|gb|EDO42990.1| predicted protein [Nematostella vectensis]</td></tr>
<tr><td>Unigene67265_All</td><td>10.77322164</td><td>Up</td><td>hypothetical protein CRE_01188 [Caenorhabditis remanei] >gi|308266682|gb|EFP10635.1| hypothetical</td></tr>
</table>

<table>
<tr><td></td><td></td><td></td><td>protein CRE_01188 [Caenorhabditis remanei]</td></tr>
<tr><td>Unigene44869_All</td><td>10.76901132</td><td>Up</td><td>cytochrome P450 9E1 [Diploptera punctata]</td></tr>
<tr><td>Unigene73384_All</td><td>10.76901132</td><td>Up</td><td>hypothetical protein BRAFLDRAFT_209578 [Branchiostoma floridae] >gi|229291947|gb|EEN62613.1| hypothetical protein BRAFLDRAFT_209578 [Branchiostoma floridae]</td></tr>
<tr><td>Unigene66453_All</td><td>10.76876327</td><td>Up</td><td>hydroxymethylglutaryl-CoA synthase [Dictyostelium fasciculatum] >gi|328876168|gb|EGG24531.1| hydroxymethylglutaryl-CoA synthase [Dictyostelium fasciculatum]</td></tr>
<tr><td>Unigene68592_All</td><td>10.76727408</td><td>Up</td><td>glucose-6-phosphate isomerase family protein [Tetrahymena thermophila] >gi|146142764|gb|EAR82568.2| glucose-6-phosphate isomerase family protein [Tetrahymena thermophila SB210]</td></tr>
<tr><td>Unigene73860_All</td><td>10.76727408</td><td>Up</td><td>unknown [Dendroctonus ponderosae]</td></tr>
<tr><td>Unigene55516_All</td><td>10.76686014</td><td>Up</td><td>PREDICTED: LOW QUALITY PROTEIN: aminopeptidase N-like [Apis florea]</td></tr>
<tr><td>Unigene68249_All</td><td>10.76512031</td><td>Up</td><td>PREDICTED: o-acetylhomoserine (thiol)-lyase-like [Xenopus (Silurana) tropicalis]</td></tr>
<tr><td>CL5033.Contig1_All</td><td>10.76279726</td><td>Up</td><td>endonuclease-reverse transcriptase [Bombyx mori]</td></tr>
<tr><td>Unigene69335_All</td><td>10.76071995</td><td>Up</td><td>succinate-semialdehyde dehydrogenase, putative [Toxoplasma gondii GT1]</td></tr>
<tr><td>Unigene67067_All</td><td>10.75972177</td><td>Up</td><td>hypothetical protein [Monosiga brevicollis MX1] >gi|163774593|gb|EDQ88220.1| predicted protein [Monosiga brevicollis MX1]</td></tr>
<tr><td>CL3304.Contig1_All</td><td>10.75688986</td><td>Up</td><td>hypothetical protein PPL_08064 [Polysphondylium pallidum PN500]</td></tr>
<tr><td>Unigene69706_All</td><td>10.75638953</td><td>Up</td><td>antioxidant, AhpC/TSA superfamily protein [Acanthamoeba castellanii str. Neff] >gi|440797463|gb|ELR18549.1| antioxidant, AhpC/TSA superfamily protein [Acanthamoeba castellanii str. Neff]</td></tr>
<tr><td>CL4428.Contig2_All</td><td>10.75380173</td><td>Up</td><td>alcohol dehydrogenase [Acanthamoeba castellanii str. Neff] >gi|440798713|gb|ELR19780.1| alcohol dehydrogenase [Acanthamoeba castellanii str. Neff]</td></tr>
<tr><td>Unigene67506_All</td><td>10.75363462</td><td>Up</td><td>H+-ATPase B subunit, partial [Bos taurus]</td></tr>
<tr><td>Unigene68867_All</td><td>10.75296597</td><td>Up</td><td>pyrroline-5-carboxylate reductase family protein [Tetrahymena thermophila] >gi|146145965|gb|EAR92117.2| pyrroline-5-carboxylate reductase family protein [Tetrahymena thermophila SB210]</td></tr>
<tr><td>Unigene68399_All</td><td>10.75137668</td><td>Up</td><td>pyruvate carboxylase [Capsaspora owczarzaki ATCC 30864] >gi|320168344|gb|EFW45243.1| pyruvate carboxylase [Capsaspora owczarzaki ATCC 30864]</td></tr>
</table>

Unigene40541_All	10.75028827	Up	dihydroxyacetone kinase [Polysphondylium pallidum PN500]
Unigene65773_All	10.74718597	Up	C. briggsae CBR-MEL-32 protein [Caenorhabditis briggsae]
Unigene44332_All	10.74693414	Up	peroxisomal [Culex quinquefasciatus] >gi|167876702|gb|EDS40085.1| peroxisomal [Culex quinquefasciatus]
Unigene42729_All	10.74542223	Up	Glycine dehydrogenase [decarboxylating], mitochondrial [Harpegnathos saltator]
Unigene68275_All	10.7441611	Up	enolase, partial [Haematoloechus sp.]
Unigene69503_All	10.73834564	Up	PREDICTED: inositol-3-phosphate synthase 1-like [Saccoglossus kowalevskii]
Unigene41331_All	10.73580985	Up	Protein B0513.5 [Caenorhabditis elegans] >gi|68061077|sp|O45228.2|PROD_CAEEL RecName: Full=Proline dehydrogenase 1, mitochondrial; AltName: Full=Proline oxidase; Flags: Precursor >gi|54110938|emb|CAB05117.2| Protein B0513.5 [Caenorhabditis elegans]
Unigene42284_All	10.73318484	Up	hypothetical protein [Paramecium tetraurelia strain d4-2] >gi|124425230|emb|CAK90017.1| unnamed protein product [Paramecium tetraurelia]
Unigene64188_All	10.72919586	Up	hypothetical protein TRIADDRAFT_59488 [Trichoplax adhaerens] >gi|190581891|gb|EDV21966.1| hypothetical protein TRIADDRAFT_59488 [Trichoplax adhaerens]
CL121.Contig1_All	10.72860081	Up	hypothetical protein [Monosiga brevicollis MX1] >gi|163776925|gb|EDQ90543.1| predicted protein [Monosiga brevicollis MX1]
CL803.Contig2_All	10.72860081	Up	Argininosuccinate synthase [Crassostrea gigas]
Unigene44524_All	10.72417275	Up	hydroxymethyltransferase [Naegleria gruberi] >gi|284096811|gb|EFC50438.1| hydroxymethyltransferase [Naegleria gruberi]
Unigene44215_All	10.72075728	Up	PREDICTED: inosine-5'-monophosphate dehydrogenase 1-like [Tursiops truncatus]
Unigene65789_All	10.72075728	Up	adenylosuccinate synthetase [Wuchereria bancrofti]
CL9412.Contig1_All	10.71990214	Up	PREDICTED: GTP cyclohydrolase 1-like [Xenopus (Silurana) tropicalis]
Unigene68830_All	10.71913209	Up	glutamine-tRNA ligase [Acanthamoeba castellanii str. Neff] >gi|440801829|gb|ELR22833.1| glutamine-tRNA ligase [Acanthamoeba castellanii str. Neff]
Unigene43855_All	10.71896091	Up	PREDICTED: C alpha-dehydrogenase-like [Xenopus (Silurana) tropicalis]
Unigene65455_All	10.71784775	Up	C. briggsae CBR-ERS-1 protein [Caenorhabditis briggsae]
Unigene71868_All	10.71596199	Up	hypothetical protein [Paramecium tetraurelia strain d4-2] >gi|124398596|emb|CAK64093.1| unnamed protein product [Paramecium tetraurelia]

Unigene72354_All	10.71536146	Up	phospholipase D1 [Capsaspora owczarzaki ATCC 30864] >gi|320164448|gb|EFW41347.1| phospholipase D1 [Capsaspora owczarzaki ATCC 30864]
CL1.Contig1_All	10.71407376	Up	hypothetical protein BRAFLDRAFT_284464 [Branchiostoma floridae] >gi|229277131|gb|EEN47929.1| hypothetical protein BRAFLDRAFT_284464 [Branchiostoma floridae]
Unigene67172_All	10.71278491	Up	transketolase [Salpingoeca sp. ATCC 50818]
CL8769.Contig2_All	10.71149491	Up	hypothetical protein [Monosiga brevicollis MX1] >gi|163770700|gb|EDQ84382.1| predicted protein [Monosiga brevicollis MX1]
Unigene70529_All	10.71115071	Up	oxidoreductase, zincbinding dehydrogenase, putative [Acanthamoeba castellanii str. Neff] >gi|440802498|gb|ELR23427.1| oxidoreductase, zincbinding dehydrogenase, putative [Acanthamoeba castellanii str. Neff]
Unigene70675_All	10.70968696	Up	predicted protein [Nematostella vectensis] >gi|156229015|gb|EDO49812.1| predicted protein [Nematostella vectensis]
CL3856.Contig2_All	10.70744542	Up	ribulosephosphate 3-epimerase [Acanthamoeba castellanii str. Neff] >gi|440804464|gb|ELR25341.1| ribulosephosphate 3-epimerase [Acanthamoeba castellanii str. Neff]
Unigene44107_All	10.70606427	Up	PREDICTED: aldose reductase A-like [Acyrthosiphon pisum]
Unigene68146_All	10.70511396	Up	hypothetical protein CAPTEDRAFT_149861 [Capitella teleta]
CL1066.Contig2_All	10.70450889	Up	vacuolar proton translocating ATPase subunit A, putative [Trypanosoma cruzi]
CL6854.Contig2_All	10.70277873	Up	PREDICTED: 3-isopropylmalate dehydratase-like [Amphimedon queenslandica]
Unigene72419_All	10.70269217	Up	GK20661 [Drosophila willistoni] >gi|194157614|gb|EDW72515.1| GK20661 [Drosophila willistoni]
CL5508.Contig6_All	10.70121981	Up	transposase [Salmo salar]
Unigene67008_All	10.69870467	Up	nedasin s-form [Capsaspora owczarzaki ATCC 30864] >gi|320167894|gb|EFW44793.1| nedasin s-form [Capsaspora owczarzaki ATCC 30864]
Unigene42804_All	10.69792321	Up	hypothetical protein CRE_17804 [Caenorhabditis remanei] >gi|308265116|gb|EFP09069.1| hypothetical protein CRE_17804 [Caenorhabditis remanei]
Unigene45427_All	10.69792321	Up	V-type H+-transporting ATPase subunit E [Clonorchis sinensis]
Unigene74891_All	10.69766263	Up	Nonspecific lipid-transfer protein, putative [Pediculus humanus corporis] >gi|212506022|gb|EEB10353.1| Nonspecific lipid-transfer protein, putative [Pediculus humanus corporis]
Unigene42173_All	10.69714133	Up	ATP synthase [Armadillidium vulgare]

Unigene44314_All	10.69592424	Up	cytochrome c oxidase subunit VIa/COX13 [Aedes aegypti] >gi|108881257|gb|EAT45482.1| AAEL003234-PA [Aedes aegypti]
Unigene45992_All	10.69540231	Up	allantoinase [Acanthamoeba castellanii str. Neff] >gi|440791366|gb|ELR12604.1| allantoinase [Acanthamoeba castellanii str. Neff]
Unigene71751_All	10.69488019	Up	C. briggsae CBR-MEL-32 protein [Caenorhabditis briggsae]
Unigene53555_All	10.69357407	Up	PREDICTED: dolichol-phosphate mannosyltransferase [Sarcophilus harrisii]
CL3539.Contig1_All	10.69095828	Up	H+-ATPase B subunit, partial [Bos taurus]
Unigene72045_All	10.69095828	Up	hypothetical protein NCLIV_005550 [Neospora caninum Liverpool] >gi|325114523|emb|CBZ50079.1| hypothetical protein NCLIV_005550 [Neospora caninum Liverpool]
Unigene72642_All	10.69034725	Up	allantoicase [Polysphondylium pallidum PN500]
Unigene50532_All	10.6900853	Up	conserved hypothetical protein [Culex quinquefasciatus] >gi|167881908|gb|EDS45291.1| conserved hypothetical protein [Culex quinquefasciatus]
Unigene43557_All	10.68964861	Up	--
CL3837.Contig1_All	10.68483627	Up	carbamoyl-phosphate synthetase-aspartate transcarbamoylase dihydroorotase [Microplitis sp. BJS-2011]
CL974.Contig2_All	10.68483627	Up	saccharopine dehydrogenase [Salpingoeca sp. ATCC 50818]
Unigene41651_All	10.68483627	Up	PREDICTED: 2-oxoisovalerate dehydrogenase subunit alpha, mitochondrial-like [Nasonia vitripennis]
Unigene74312_All	10.68483627	Up	GF17012 [Drosophila ananassae] >gi|308191518|sp|B3M343.1|PURA_DROAN RecName: Full=Adenylosuccinate synthetase; Short=AMPSase; Short=AdSS; AltName: Full=IMP--aspartate ligase >gi|190626919|gb|EDV42443.1| GF17012 [Drosophila ananassae]
Unigene79789_All	10.68483627	Up	PREDICTED: phospholipase D1-like isoform 2 [Megachile rotundata]
Unigene74010_All	10.68211676	Up	hypothetical protein [Monosiga brevicollis MX1] >gi|163773810|gb|EDQ87446.1| predicted protein [Monosiga brevicollis MX1]
Unigene67467_All	10.678248	Up	PREDICTED: choline kinase alpha-like [Oryzias latipes]
Unigene70162_All	10.67789578	Up	carbamoyl-phosphate synthase [Dichochrysa luctuosa]
Unigene43889_All	10.67595703	Up	triosephosphate isomerase, putative [Ichthyophthirius multifiliis] >gi|340506780|gb|EGR32851.1| triosephosphate isomerase, putative [Ichthyophthirius multifiliis]
Unigene72804_All	10.67472192	Up	acyl-CoA:1,2-diacylglycerol O-transferase [Capsaspora owczarzaki ATCC 30864] >gi|320163993|gb|EFW40892.1| acyl-CoA:1,2-diacylglycerol O-transferase [Capsaspora

			owczarzaki ATCC 30864]
CL9475.Contig1_All	10.67375074	Up	Sphingolipid delta(4)-desaturase/C4-hydroxylase DES2, partial [Columba livia]
Unigene66914_All	10.67339742	Up	ribose-5-phosphate isomerase [Dictyostelium fasciculatum] >gi|328874273|gb|EGG22639.1| ribose-5-phosphate isomerase [Dictyostelium fasciculatum]
Unigene64994_All	10.67313237	Up	LOC100125119 protein [Xenopus (Silurana) tropicalis]
Unigene62477_All	10.67180641	Up	PREDICTED: uncharacterized protein LOC100889850 [Strongylocentrotus purpuratus]
Unigene75321_All	10.67180641	Up	hypothetical protein NEMVEDRAFT_v1g152410 [Nematostella vectensis] >gi|156201566|gb|EDO26994.1| predicted protein [Nematostella vectensis]
Unigene67908_All	10.67101024	Up	Methylcrotonoyl-CoA carboxylase beta chain [Chelonia mydas]
Unigene78139_All	10.67101024	Up	NADH:ubiquinone oxidoreductase 24 kDa subunit [Acanthamoeba castellanii str. Neff] >gi|440804510|gb|ELR25387.1| NADH:ubiquinone oxidoreductase 24 kDa subunit [Acanthamoeba castellanii str. Neff]
Unigene41392_All	10.66844183	Up	isopropylmalate dehydrogenase [Capsaspora owczarzaki ATCC 30864] >gi|320169661|gb|EFW46560.1| isopropylmalate dehydrogenase [Capsaspora owczarzaki ATCC 30864]
Unigene41597_All	10.66826453	Up	hypothetical protein [Monosiga brevicollis MX1] >gi|163777401|gb|EDQ91018.1| predicted protein [Monosiga brevicollis MX1]
Unigene73300_All	10.66826453	Up	Methylcrotonoyl-CoA carboxylase beta chain, mitochondrial [Crassostrea gigas]
CL7782.Contig1_All	10.66675659	Up	PREDICTED: squalene monooxygenase [Sarcophilus harrisii]
Unigene44025_All	10.66640155	Up	PREDICTED: similar to CG3194 CG3194-PA [Tribolium castaneum] >gi|270013933|gb|EFA10381.1| hypothetical protein TcasGA2_TC012612 [Tribolium castaneum]
Unigene66845_All	10.6656024	Up	glycine cleavage system Pprotein [Acanthamoeba castellanii str. Neff] >gi|440798630|gb|ELR19697.1| glycine cleavage system Pprotein [Acanthamoeba castellanii str. Neff]
Unigene72542_All	10.66533592	Up	unnamed protein product [Tetraodon nigroviridis]
Unigene42108_All	10.66409168	Up	cytochrome b2 [Salpingoeca sp. ATCC 50818]
Unigene77973_All	10.66409168	Up	PREDICTED: maleylacetoacetate isomerase-like, partial [Hydra magnipapillata]
Unigene72095_All	10.66275737	Up	hypothetical protein DICPUDRAFT_149192 [Dictyostelium purpureum] >gi|325084747|gb|EGC38168.1| hypothetical protein DICPUDRAFT_149192 [Dictyostelium purpureum]
Unigene73846_All	10.66159998	Up	hypothetical protein TRIADDRAFT_35024 [Trichoplax adhaerens] >gi|190577454|gb|EDV18533.1|

<table>
<tr><td></td><td></td><td></td><td>hypothetical protein TRIADDRAFT_35024 [Trichoplax adhaerens]</td></tr>
<tr><td>Unigene73912_All</td><td>10.66159998</td><td>Up</td><td>PREDICTED: LOW QUALITY PROTEIN: kynureninase-like [Strongylocentrotus purpuratus]</td></tr>
<tr><td>Unigene70887_All</td><td>10.66142183</td><td>Up</td><td>4-aminobutyrate transaminase [Capsaspora owczarzaki ATCC 30864] >gi|320169641|gb|EFW46540.1| 4-aminobutyrate transaminase [Capsaspora owczarzaki ATCC 30864]</td></tr>
<tr><td>Unigene43274_All</td><td>10.66035251</td><td>Up</td><td>hydroxymethylglutarylCoA reductase (NADPH), putative [Acanthamoeba castellanii str. Neff] >gi|440794223|gb|ELR15390.1| hydroxymethylglutarylCoA reductase (NADPH), putative [Acanthamoeba castellanii str. Neff]</td></tr>
<tr><td>Unigene43806_All</td><td>10.66035251</td><td>Up</td><td>PREDICTED: 1-acyl-sn-glycerol-3-phosphate acyltransferase alpha-like isoform 3 [Acyrthosiphon pisum] >gi|328705895|ref|XP_003242936.1| PREDICTED: 1-acyl-sn-glycerol-3-phosphate acyltransferase alpha-like isoform 4 [Acyrthosiphon pisum]</td></tr>
<tr><td>Unigene65315_All</td><td>10.6593716</td><td>Up</td><td>unknown [Dendroctonus ponderosae]</td></tr>
<tr><td>Unigene71635_All</td><td>10.65892551</td><td>Up</td><td>hypothetical protein CAPTEDRAFT_178602 [Capitella teleta]</td></tr>
<tr><td>Unigene73923_All</td><td>10.65615668</td><td>Up</td><td>hypothetical protein DICPUDRAFT_77429 [Dictyostelium purpureum] >gi|325083469|gb|EGC36921.1| hypothetical protein DICPUDRAFT_77429 [Dictyostelium purpureum]</td></tr>
<tr><td>Unigene62564_All</td><td>10.65015421</td><td>Up</td><td>PREDICTED: v-type proton ATPase subunit B-like [Acyrthosiphon pisum]</td></tr>
<tr><td>Unigene43342_All</td><td>10.64943583</td><td>Up</td><td>GF22046 [Drosophila ananassae] >gi|190617107|gb|EDV32631.1| GF22046 [Drosophila ananassae]</td></tr>
<tr><td>CL8364.Contig3_All</td><td>10.64826769</td><td>Up</td><td>hypothetical protein SINV_09923 [Solenopsis invicta]</td></tr>
<tr><td>Unigene70665_All</td><td>10.64610864</td><td>Up</td><td>hypothetical protein IMG5_148050 [Ichthyophthirius multifiliis] >gi|340503209|gb|EGR29821.1| hypothetical protein IMG5_148050 [Ichthyophthirius multifiliis]</td></tr>
<tr><td>Unigene66005_All</td><td>10.64331508</td><td>Up</td><td>predicted protein [Nematostella vectensis] >gi|156210604|gb|EDO31766.1| predicted protein [Nematostella vectensis]</td></tr>
<tr><td>Unigene44114_All</td><td>10.64277376</td><td>Up</td><td>PREDICTED: probable C-5 sterol desaturase-like [Metaseiulus occidentalis]</td></tr>
<tr><td>CL3124.Contig2_All</td><td>10.63979289</td><td>Up</td><td>Probable malate dehydrogenase, mitochondrial precursor , putative [Brugia malayi] >gi|158591907|gb|EDP30510.1| Probable malate dehydrogenase, mitochondrial precursor , putative [Brugia malayi]</td></tr>
<tr><td>Unigene72654_All</td><td>10.63979289</td><td>Up</td><td>GH21178 [Drosophila grimshawi] >gi|193902108|gb|EDW00975.1| GH21178 [Drosophila grimshawi]</td></tr>
<tr><td>Unigene70883_All</td><td>10.63897886</td><td>Up</td><td>PREDICTED: ferrochelatase, mitochondrial isoform 2 [Callithrix jacchus]</td></tr>
<tr><td>Unigene71625_All</td><td>10.6387979</td><td>Up</td><td>5-aminolevulinic acid synthase, related [Neospora caninum Liverpool] >gi|325117441|emb|CBZ52993.1|</td></tr>
</table>

			5-aminolevulinic acid synthase, related [Neospora caninum Liverpool]
Unigene77727_All	10.6379833	Up	Glycerol kinase 2 [Polysphondylium pallidum PN500]
Unigene69025_All	10.63571812	Up	fumarate hydratase [Wuchereria bancrofti]
Unigene44378_All	10.63517395	Up	hypothetical protein CAPTEDRAFT_229052 [Capitella teleta]
Unigene43071_All	10.63390341	Up	aconitase, mitochondrial [Dictyostelium discoideum AX4] >gi|74856524|sp|Q54XS2.1|ACON_DICDI RecName: Full=Probable aconitate hydratase, mitochondrial; Short=Aconitase; AltName: Full=Citrate hydro-lyase; Flags: Precursor >gi|60470010|gb|EAL67991.1| aconitase, mitochondrial [Dictyostelium discoideum AX4]
Unigene63564_All	10.62853667	Up	Glycerol kinase [Crassostrea gigas]
Unigene67724_All	10.62716906	Up	PREDICTED: 2-oxoisovalerate dehydrogenase subunit beta, mitochondrial-like isoform 1 [Strongylocentrotus purpuratus] >gi|390335812|ref|XP_796781.3| PREDICTED: 2-oxoisovalerate dehydrogenase subunit beta, mitochondrial-like isoform 2 [Strongylocentrotus purpuratus]
Unigene52706_All	10.62698661	Up	hypothetical protein CAPTEDRAFT_165979 [Capitella teleta]
Unigene70689_All	10.62680414	Up	RNA polymerase II core subunit [Dictyostelium purpureum] >gi|325083799|gb|EGC37242.1| RNA polymerase II core subunit [Dictyostelium purpureum]
Unigene71862_All	10.62552621	Up	PREDICTED: serine palmitoyltransferase 1-like [Takifugu rubripes]
Unigene69810_All	10.62442994	Up	hypothetical protein TcasGA2_TC011540 [Tribolium castaneum]
Unigene71053_All	10.62196027	Up	Cytosolic nonspecific dipeptidase, putative [Pediculus humanus corporis] >gi|212516890|gb|EEB18844.1| Cytosolic nonspecific dipeptidase, putative [Pediculus humanus corporis]
Unigene73881_All	10.62113611	Up	acyl-coa dehydrogenase, putative [Perkinsus marinus ATCC 50983] >gi|239890237|gb|EER12110.1| acyl-coa dehydrogenase, putative [Perkinsus marinus ATCC 50983]
Unigene43427_All	10.6209529	Up	PREDICTED: probable medium-chain specific acyl-CoA dehydrogenase, mitochondrial-like [Nasonia vitripennis]
Unigene66671_All	10.61966977	Up	PREDICTED: pyruvate kinase isozymes M1/M2-like [Amphimedon queenslandica]
Unigene67489_All	10.61847727	Up	fatty acid synthase, putative [Pediculus humanus corporis] >gi|212517394|gb|EEB19298.1| fatty acid synthase, putative [Pediculus humanus corporis]
Unigene73100_All	10.61765112	Up	putative CAD trifunctional protein [Blattella germanica]
Unigene67921_All	10.61700823	Up	hypothetical protein AND_06389 [Anopheles darlingi]

Unigene72937_All	10.61700823	Up	NADPH-dependent D-xylose reductase [Salmo salar] >gi|209736332|gb|ACI69035.1| NADPH-dependent D-xylose reductase [Salmo salar]
Unigene75682_All	10.61268423	Up	PREDICTED: N-acetylgalactosamine kinase-like [Strongylocentrotus purpuratus]
Unigene72732_All	10.61074804	Up	PREDICTED: aspartate aminotransferase, cytoplasmic-like, partial [Mus musculus]
Unigene77632_All	10.61047123	Up	CYP4CD1 [Liposcelis bostrychophila]
CL476.Contig1_All	10.60501645	Up	PREDICTED: c-4 methylsterol oxidase-like [Monodelphis domestica]
Unigene75018_All	10.60093501	Up	6-phosphogluconate dehydrogenase [Capsaspora owczarzaki ATCC 30864] >gi|320165489|gb|EFW42388.1| 6-phosphogluconate dehydrogenase [Capsaspora owczarzaki ATCC 30864]
Unigene41329_All	10.59861085	Up	PREDICTED: ethanolamine-phosphate cytidylyltransferase-like, partial [Strongylocentrotus purpuratus]
Unigene44644_All	10.59861085	Up	hypothetical protein [Monosiga brevicollis MX1] >gi|163775212|gb|EDQ88837.1| predicted protein [Monosiga brevicollis MX1]
Unigene76185_All	10.59768014	Up	hypothetical protein [Paramecium tetraurelia strain d4-2] >gi|124412187|emb|CAK77368.1| unnamed protein product [Paramecium tetraurelia]
Unigene72356_All	10.59712143	Up	chloride channel, putative [Toxoplasma gondii GT1]
Unigene41085_All	10.59618976	Up	glycerol kinase [Culex quinquefasciatus] >gi|167867273|gb|EDS30656.1| glycerol kinase [Culex quinquefasciatus]
Unigene69995_All	10.59516422	Up	saccharopine dehydrogenase [Salpingoeca sp. ATCC 50818]
Unigene65810_All	10.59441792	Up	AMP-binding enzyme family protein [Oxytricha trifallax]
Unigene45898_All	10.5926439	Up	isocitrate lyase [Capsaspora owczarzaki ATCC 30864] >gi|320165109|gb|EFW42008.1| isocitrate lyase [Capsaspora owczarzaki ATCC 30864]
CL3202.Contig2_All	10.59077416	Up	transketolase [Salpingoeca sp. ATCC 50818]
Unigene75241_All	10.58937026	Up	Protein VHA-9 [Caenorhabditis elegans] >gi|2833318|sp|Q23680.1|VATF_CAEEL RecName: Full=Probable V-type proton ATPase subunit F; Short=V-ATPase subunit F; AltName: Full=Vacuolar proton pump subunit F >gi|3881898|emb|CAA88888.1| Protein VHA-9 [Caenorhabditis elegans]
Unigene71916_All	10.58768377	Up	PREDICTED: aldehyde dehydrogenase, mitochondrial [Metaseiulus occidentalis]
Unigene71111_All	10.58392895	Up	PREDICTED: similar to xylulokinase homolog [Tribolium castaneum] >gi|270002483|gb|EEZ98930.1| hypothetical protein TcasGA2_TC004550 [Tribolium castaneum]

Unigene46267_All	10.58148307	Up	PREDICTED: similar to zinc binding dehydrogenase [Tribolium castaneum] >gi|270012629|gb|EFA09077.1| hypothetical protein TcasGA2_TC006794 [Tribolium castaneum]
Unigene74477_All	10.57790084	Up	--
Unigene75472_All	10.57790084	Up	predicted protein [Nematostella vectensis] >gi|156219993|gb|EDO40867.1| predicted protein [Nematostella vectensis]
Unigene41808_All	10.57251076	Up	NADP-dependent malate dehydrogenase [Polysphondylium pallidum PN500]
Unigene73950_All	10.57251076	Up	predicted protein [Nematostella vectensis] >gi|156218018|gb|EDO38923.1| predicted protein [Nematostella vectensis]
Unigene45489_All	10.57146825	Up	hypothetical protein PPL_11461 [Polysphondylium pallidum PN500]
Unigene72145_All	10.57146825	Up	PREDICTED: gamma-glutamyltransferase 1-like [Saccoglossus kowalevskii]
Unigene73893_All	10.57146825	Up	p23 co-chaperone, putative [Plasmodium falciparum 3D7] >gi|255528889|gb|AAN37123.2| p23 co-chaperone, putative [Plasmodium falciparum 3D7]
CL4274.Contig2_All	10.56890615	Up	3-ketoacyl-CoA thiolase B, putative [Toxoplasma gondii GT1]
Unigene71072_All	10.56757587	Up	hypothetical protein BRAFLDRAFT_287716 [Branchiostoma floridae] >gi|229297293|gb|EEN67929.1| hypothetical protein BRAFLDRAFT_287716 [Branchiostoma floridae]
Unigene67162_All	10.56500685	Up	AGAP000260-PB [Anopheles gambiae str. PEST] >gi|347963492|ref|XP_003436958.1| AGAP000260-PC [Anopheles gambiae str. PEST] >gi|333467174|gb|EGK96488.1| AGAP000260-PB [Anopheles gambiae str. PEST] >gi|333467175|gb|EGK96489.1| AGAP000260-PC [Anopheles gambiae str. PEST]
Unigene42878_All	10.56319627	Up	homogentisate 1,2-dioxygenase [Aedes aegypti] >gi|108869866|gb|EAT34091.1| AAEL013637-PA [Aedes aegypti]
Unigene74688_All	10.56252864	Up	PREDICTED: cysteine dioxygenase type 1-like [Bombus terrestris]
Unigene73474_All	10.562147	Up	hypothetical protein CAPTEDRAFT_152418 [Capitella teleta]
Unigene70877_All	10.55870769	Up	mthfd1 protein [Capsaspora owczarzaki ATCC 30864] >gi|320168842|gb|EFW45741.1| mthfd1 protein [Capsaspora owczarzaki ATCC 30864]
CL2031.Contig1_All	10.55842071	Up	cysteine synthase [Salpingoeca sp. ATCC 50818]
CL7462.Contig2_All	10.55736797	Up	serine hydroxymethyltransferase [Loa loa]
Unigene45171_All	10.5510353	Up	pyruvate carboxylase [Capsaspora owczarzaki ATCC 30864] >gi|320168344|gb|EFW45243.1| pyruvate

			carboxylase [Capsaspora owczarzaki ATCC 30864]
Unigene42379_All	10.54728016	Up	ATP synthase, H+ transporting, mitochondrial F1 complex, gamma polypeptide 1 [Xenopus laevis] >gi|48734669|gb|AAH72367.1| Atp5c1-prov protein [Xenopus laevis]
Unigene45987_All	10.54573675	Up	GI19942 [Drosophila mojavensis] >gi|193909524|gb|EDW08391.1| GI19942 [Drosophila mojavensis]
Unigene44974_All	10.54390182	Up	V-type proton ATPase subunit d 2 [Chelonia mydas]
Unigene69594_All	10.54390182	Up	ribonucleotide reductase M1 subunit [Capsaspora owczarzaki ATCC 30864] >gi|320167592|gb|EFW44491.1| ribonucleotide reductase M1 subunit [Capsaspora owczarzaki ATCC 30864]
Unigene44456_All	10.54225805	Up	alpha mannosidase Ib [Drosophila melanogaster] >gi|7301742|gb|AAF56854.1| alpha mannosidase Ib [Drosophila melanogaster] >gi|16186123|gb|AAL14004.1| SD05769p [Drosophila melanogaster]
Unigene44231_All	10.54041868	Up	PREDICTED: inosine-5'-monophosphate dehydrogenase 2-like [Metaseiulus occidentalis]
Unigene74359_All	10.53974043	Up	PREDICTED: cytochrome c oxidase assembly protein COX15 homolog [Metaseiulus occidentalis]
CL7930.Contig1_All	10.53838296	Up	AcylCoA oxidase [Acanthamoeba castellanii str. Neff] >gi|440789742|gb|ELR11041.1| AcylCoA oxidase [Acanthamoeba castellanii str. Neff]
Unigene76179_All	10.5360529	Up	hypothetical protein [Paramecium tetraurelia strain d4-2] >gi|124428917|emb|CAK93698.1| unnamed protein product [Paramecium tetraurelia]
CL8623.Contig1_All	10.53585856	Up	PREDICTED: transaldolase-like [Oreochromis niloticus]
Unigene42266_All	10.53440016	Up	PREDICTED: lysosomal alpha-glucosidase-like [Acyrthosiphon pisum]
Unigene70672_All	10.53352441	Up	PREDICTED: lipoyl synthase, mitochondrial-like [Amphimedon queenslandica]
Unigene66419_All	10.53060141	Up	nuclear elongation and deformation protein 1 [Capsaspora owczarzaki ATCC 30864] >gi|320166327|gb|EFW43226.1| nuclear elongation and deformation protein 1 [Capsaspora owczarzaki ATCC 30864]
Unigene69136_All	10.53060141	Up	aldehyde dehydrogenase-6 [Mus musculus]
CL3104.Contig1_All	10.52728151	Up	GE20195 [Drosophila yakuba] >gi|194180552|gb|EDW94163.1| GE20195 [Drosophila yakuba]
Unigene42692_All	10.52728151	Up	PREDICTED: uridine 5'-monophosphate synthase isoform 1 [Nomascus leucogenys]
Unigene70600_All	10.52728151	Up	hypothetical protein BRAFLDRAFT_125140 [Branchiostoma floridae] >gi|229277374|gb|EEN48170.1| hypothetical protein BRAFLDRAFT_125140 [Branchiostoma floridae]
Unigene74409_All	10.52728151	Up	PREDICTED: indoleamine 2,3-dioxygenase 2 [Nomascus leucogenys]

Unigene68872_All	10.52395394	Up	hydroxysteroid dehydrogenase, putative [Acanthamoeba castellanii str. Neff] >gi|440796489|gb|ELR17598.1| hydroxysteroid dehydrogenase, putative [Acanthamoeba castellanii str. Neff]
Unigene72197_All	10.52395394	Up	hypothetical protein BRAFLDRAFT_285351 [Branchiostoma floridae] >gi|229277578|gb|EEN48371.1| hypothetical protein BRAFLDRAFT_285351 [Branchiostoma floridae]
Unigene74050_All	10.52395394	Up	FAD synthetase [Crassostrea gigas]
Unigene64278_All	10.52228727	Up	phosphoserine aminotransferase [Capsaspora owczarzaki ATCC 30864] >gi|320168494|gb|EFW45393.1| phosphoserine aminotransferase [Capsaspora owczarzaki ATCC 30864]
CL4181.Contig1_All	10.52012755	Up	hypothetical protein TRIADDRAFT_27878 [Trichoplax adhaerens] >gi|190582943|gb|EDV23014.1| hypothetical protein TRIADDRAFT_27878 [Trichoplax adhaerens]
Unigene44473_All	10.51943969	Up	glycine dehydrogenase [Polysphondylium pallidum PN500]
CL748.Contig2_All	10.51737413	Up	malate dehydrogenase [Bursaphelenchus xylophilus]
Unigene75747_All	10.51619248	Up	short-chain dehydrogenase/reductase SDR [Salpingoeca sp. ATCC 50818]
Unigene71640_All	10.51491126	Up	predicted protein [Nematostella vectensis] >gi|156217830|gb|EDO38739.1| predicted protein [Nematostella vectensis]
Unigene75310_All	10.5137276	Up	hypothetical protein [Monosiga brevicollis MX1] >gi|163771812|gb|EDQ85473.1| predicted protein [Monosiga brevicollis MX1]
Unigene67288_All	10.51274046	Up	PREDICTED: similar to Lipoamide acyltransferase component of branched-chain alpha-keto acid dehydrogenase complex, mitochondrial precursor (Dihydrolipoyllysine-residue (2-methylpropanoyl)transferase) (E2) (Dihydrolipoamide branched chain transacylase) (BCKAD [Tribolium castaneum] >gi|270015048|gb|EFA11496.1| hypothetical protein TcasGA2_TC014209 [Tribolium castaneum]
Unigene66271_All	10.51076417	Up	pyruvate kinase [Capsaspora owczarzaki ATCC 30864] >gi|320167794|gb|EFW44693.1| pyruvate kinase [Capsaspora owczarzaki ATCC 30864]
Unigene45115_All	10.50610919	Up	PREDICTED: enoyl-CoA hydratase, mitochondrial [Otolemur garnettii]
Unigene73713_All	10.50610919	Up	NAD-dependent malic enzyme, putative [Acanthamoeba castellanii str. Neff] >gi|440804624|gb|ELR25501.1| NAD-dependent malic enzyme, putative [Acanthamoeba castellanii str. Neff]

Unigene75190_All	10.50422312	Up	putative glutamyl-tRNA synthetase [Leishmania braziliensis MHOM/BR/75/M2904] >gi|134064278|emb|CAM40477.1| putative glutamyl-tRNA synthetase [Leishmania braziliensis MHOM/BR/75/M2904]
Unigene53968_All	10.50392509	Up	argininosuccinate lyase [Capsaspora owczarzaki ATCC 30864] >gi|320168757|gb|EFW45656.1| argininosuccinate lyase [Capsaspora owczarzaki ATCC 30864]
CL3831.Contig1_All	10.50362701	Up	hypothetical protein ACA1_369890 [Acanthamoeba castellanii str. Neff] >gi|440797151|gb|ELR18246.1| hypothetical protein ACA1_369890 [Acanthamoeba castellanii str. Neff]
Unigene75983_All	10.50263294	Up	hypothetical protein DICPUDRAFT_39352 [Dictyostelium purpureum] >gi|325078156|gb|EGC31823.1| hypothetical protein DICPUDRAFT_39352 [Dictyostelium purpureum]
Unigene68516_All	10.50114054	Up	hypothetical protein TRIADDRAFT_49959 [Trichoplax adhaerens] >gi|190586239|gb|EDV26292.1| hypothetical protein TRIADDRAFT_49959 [Trichoplax adhaerens]
CL729.Contig2_All	10.50034397	Up	CDP-diacylglycerol--inositol 3-phosphatidyltransferase [Myotis davidii]
Unigene77904_All	10.49775206	Up	long-chain-fatty-acid coa ligase [Culex quinquefasciatus] >gi|167879266|gb|EDS42649.1| long-chain-fatty-acid coa ligase [Culex quinquefasciatus]
Unigene69309_All	10.49545533	Up	PREDICTED: cytidine deaminase [Macaca mulatta]
Unigene75281_All	10.49545533	Up	--
Unigene71920_All	10.49515549	Up	aldehyde dehydrogenase type III-PG [Capsaspora owczarzaki ATCC 30864] >gi|320165513|gb|EFW42412.1| aldehyde dehydrogenase type III-PG [Capsaspora owczarzaki ATCC 30864]
Unigene75960_All	10.49445561	Up	hypothetical protein NAEGRDRAFT_80131 [Naegleria gruberi] >gi|284089454|gb|EFC43111.1| hypothetical protein NAEGRDRAFT_80131 [Naegleria gruberi]
Unigene75557_All	10.48974763	Up	isocitrate dehydrogenase [Polysphondylium pallidum PN500]
CL8836.Contig2_All	10.48683502	Up	H+-ATPase B subunit, partial [Bos taurus]
Unigene55294_All	10.48633225	Up	PREDICTED: RNA-directed DNA polymerase from mobile element jockey-like [Strongylocentrotus purpuratus]
CL5949.Contig1_All	10.48552746	Up	Transaldolase [Callorhinchus milii]
Unigene45763_All	10.48472221	Up	leukotriene A4 hydrolase [Bombyx mori] >gi|95102808|gb|ABF51345.1| leukotriene A4 hydrolase [Bombyx mori]

CL7797.Contig2_All	10.47836516	Up	PREDICTED: 6-phosphogluconate dehydrogenase, decarboxylating [Amphimedon queenslandica]
CL9431.Contig2_All	10.47836516	Up	PREDICTED: adenosylhomocysteinase [Ciona intestinalis]
Unigene42293_All	10.47836516	Up	fatty acid betaoxidation-related protein [Acanthamoeba castellanii str. Neff] >gi|440797859|gb|ELR18933.1| fatty acid betaoxidation-related protein [Acanthamoeba castellanii str. Neff]
Unigene43781_All	10.47836516	Up	hypothetical protein CAPTEDRAFT_151890 [Capitella teleta]
Unigene65335_All	10.47836516	Up	hypothetical protein TcasGA2_TC004950 [Tribolium castaneum]
Unigene72423_All	10.47836516	Up	hypothetical protein CAPTEDRAFT_149854 [Capitella teleta]
Unigene41829_All	10.47522678	Up	PREDICTED: ceramide synthase 4-like [Sarcophilus harrisii]
Unigene75718_All	10.47431435	Up	hypothetical protein DICPUDRAFT_9137 [Dictyostelium purpureum] >gi|325081772|gb|EGC35276.1| hypothetical protein DICPUDRAFT_9137 [Dictyostelium purpureum]
Unigene71021_All	10.47401008	Up	c-24(28) sterol reductase [Naegleria gruberi] >gi|284097172|gb|EFC50799.1| c-24(28) sterol reductase [Naegleria gruberi]
Unigene70475_All	10.47238623	Up	RecName: Full=Kynureninase; AltName: Full=L-kynurenine hydrolase
Unigene45745_All	10.46770743	Up	PREDICTED: ribose-phosphate pyrophosphokinase 1-like [Equus caballus]
Unigene70484_All	10.46413729	Up	AMP deaminase 2 [Crassostrea gigas]
Unigene72453_All	10.45450494	Up	AAEL001528-PA [Aedes aegypti]
Unigene75301_All	10.45028337	Up	PREDICTED: dihydroxy-acid dehydratase-like [Amphimedon queenslandica]
Unigene63834_All	10.44480784	Up	malate dehydrogenase [Bursaphelenchus xylophilus]
CL5250.Contig1_All	10.44377239	Up	4nitrophenylphosphatase-like protein [Acanthamoeba castellanii str. Neff] >gi|440789564|gb|ELR10871.1| 4nitrophenylphosphatase-like protein [Acanthamoeba castellanii str. Neff]
Unigene69471_All	10.4427362	Up	carbamoyl-phosphate synthase large chain [Culex quinquefasciatus] >gi|167865521|gb|EDS28904.1| carbamoyl-phosphate synthase large chain [Culex quinquefasciatus]
Unigene69418_All	10.44035012	Up	PREDICTED: phospholipase D1-like [Saccoglossus kowalevskii]
Unigene74284_All	10.44035012	Up	malate synthase A, putative [Acanthamoeba castellanii str. Neff] >gi|440802061|gb|ELR23000.1| malate synthase A, putative [Acanthamoeba castellanii str. Neff]
CL2290.Contig1_All	10.43931146	Up	glyceraldehyde 3-phosphate dehydrogenase, putative [Perkinsus marinus ATCC 50983] >gi|239904296|gb|EER20551.1| glyceraldehyde 3-phosphate dehydrogenase, putative [Perkinsus

			marinus ATCC 50983]
Unigene63063_All	10.43931146	Up	unnamed protein product [Tetraodon nigroviridis]
Unigene75026_All	10.43931146	Up	conserved hypothetical protein [Capsaspora owczarzaki ATCC 30864] >gi|320169101|gb|EFW46000.1| conserved hypothetical protein [Capsaspora owczarzaki ATCC 30864]
Unigene73842_All	10.43681563	Up	aldehyde oxidase [Aedes aegypti] >gi|108873420|gb|EAT37645.1| AAEL010367-PA [Aedes aegypti]
CL1763.Contig2_All	10.43650335	Up	ATP-citrate synthase [Harpegnathos saltator]
Unigene71145_All	10.43598272	Up	PREDICTED: uridine-cytidine kinase-like 1 [Metaseiulus occidentalis]
Unigene66690_All	10.43567026	Up	6-phosphogluconate dehydrogenase [Capsaspora owczarzaki ATCC 30864] >gi|320165489|gb|EFW42388.1| 6-phosphogluconate dehydrogenase [Capsaspora owczarzaki ATCC 30864]
Unigene77462_All	10.43107967	Up	N-D-ribosylpurine ribohydrolase [Polysphondylium pallidum PN500]
Unigene75745_All	10.42794133	Up	enoyl-CoA hydratase [Dictyostelium fasciculatum] >gi|328874800|gb|EGG23165.1| enoyl-CoA hydratase [Dictyostelium fasciculatum]
Unigene43753_All	10.42741761	Up	alanine transaminase [Dictyostelium purpureum] >gi|325078081|gb|EGC31753.1| alanine transaminase [Dictyostelium purpureum]
CL2703.Contig1_All	10.42437627	Up	knockdown, isoform B [Drosophila melanogaster] >gi|19527883|gb|AAL90056.1| AT12538p [Drosophila melanogaster] >gi|22831817|gb|AAN09169.1| knockdown, isoform B [Drosophila melanogaster]
Unigene73568_All	10.42174927	Up	serine C-palmitoyltransferase subunit [Dictyostelium discoideum AX4] >gi|74859279|sp|Q55FL5.1|SPTC1_DICDI RecName: Full=Serine palmitoyltransferase 1; AltName: Full=Long chain base biosynthesis protein 1; Short=LCB 1; AltName: Full=Serine-palmitoyl-CoA transferase 1; Short=SPT 1; Short=SPT1 >gi|60475546|gb|EAL73481.1| serine C-palmitoyltransferase subunit [Dictyostelium discoideum AX4]
Unigene43114_All	10.42038134	Up	palmitoyl-protein thioesterase 1 [Dictyostelium discoideum AX4] >gi|60466552|gb|EAL64604.1| palmitoyl-protein thioesterase 1 [Dictyostelium discoideum AX4]
CL9789.Contig3_All	10.41974955	Up	hypothetical protein DICPUDRAFT_34436 [Dictyostelium purpureum] >gi|325081249|gb|EGC34771.1| hypothetical protein DICPUDRAFT_34436 [Dictyostelium purpureum]
CL5784.Contig1_All	10.41943355	Up	2-oxoisovalerate dehydrogenase subunit alpha, mitochondrial precursor [Caligus clemensi]
CL1987.Contig2_All	10.41563615	Up	Phosphoglucomutase-1 [Crassostrea gigas]

Unigene45604_All	10.41563615	Up	hypothetical protein [Monosiga brevicollis MX1] >gi|163770723|gb|EDQ84405.1| predicted protein [Monosiga brevicollis MX1]
Unigene71839_All	10.41373369	Up	serine palmitoyltransferase 2 [Danio rerio] >gi|63101255|gb|AAH95355.1| Serine palmitoyltransferase, long chain base subunit 2 [Danio rerio] >gi|182890226|gb|AAI65370.1| Sptlc2 protein [Danio rerio]
Unigene40606_All	10.41320479	Up	predicted protein [Nematostella vectensis] >gi|156219681|gb|EDO40559.1| predicted protein [Nematostella vectensis]
Unigene66321_All	10.41119318	Up	Pyruvate carboxylase [Chelonia mydas]
Unigene70328_All	10.40620501	Up	acetyl-CoA carboxylase [Polysphondylium pallidum PN500]
CL3777.Contig1_All	10.40119953	Up	unknown [Dendroctonus ponderosae]
CL208.Contig1_All	10.39949156	Up	predicted protein [Nematostella vectensis] >gi|156227646|gb|EDO48448.1| predicted protein [Nematostella vectensis]
Unigene75795_All	10.39820926	Up	lonCoA ligase 5 [Trichinella spiralis] >gi|316976543|gb|EFV59820.1| lonCoA ligase 5 [Trichinella spiralis]
Unigene76011_All	10.39820926	Up	pyruvate kinase [Capsaspora owczarzaki ATCC 30864] >gi|320167794|gb|EFW44693.1| pyruvate kinase [Capsaspora owczarzaki ATCC 30864]
Unigene77476_All	10.39703282	Up	cytosolic malate dehydrogenase [Mytilus galloprovincialis]
Unigene70109_All	10.39221008	Up	glycerol kinase [Aedes aegypti] >gi|108879512|gb|EAT43737.1| AAEL004853-PA [Aedes aegypti]
Unigene77603_All	10.39221008	Up	C. briggsae CBR-IVD-1 protein [Caenorhabditis briggsae]
Unigene76857_All	10.39124359	Up	PREDICTED: choline-sulfatase-like [Amphimedon queenslandica]
CL6846.Contig2_All	10.38973888	Up	hypothetical protein [Paramecium tetraurelia strain d4-2] >gi|124393497|emb|CAK59024.1| unnamed protein product [Paramecium tetraurelia]
Unigene73600_All	10.37883671	Up	hypothetical protein DAPPUDRAFT_300069 [Daphnia pulex]
Unigene72338_All	10.37395266	Up	CBR-ERS-1 protein [Loa loa]
Unigene69814_All	10.37177664	Up	predicted protein [Nematostella vectensis] >gi|156211265|gb|EDO32382.1| predicted protein [Nematostella vectensis]
Unigene75159_All	10.37144996	Up	Non-specific lipid-transfer protein [Crassostrea gigas]
Unigene71271_All	10.36730553	Up	ubiquitin-conjugating enzyme E2 [Dictyostelium discoideum AX4] >gi|74936046|sp|Q94490.1|UBCB_DICDI RecName: Full=Ubiquitin conjugating enzyme E2 B;

			AltName: Full=UBC1 >gi|1553009|gb|AAB08700.1| UbcB [Dictyostelium discoideum] >gi|60466852|gb|EAL64896.1| ubiquitin-conjugating enzyme E2 [Dictyostelium discoideum AX4]
Unigene77200_All	10.36730553	Up	acyl-Coenzyme A dehydrogenase [Capsaspora owczarzaki ATCC 30864] >gi|320166428|gb|EFW43327.1| acyl-Coenzyme A dehydrogenase [Capsaspora owczarzaki ATCC 30864]
Unigene74289_All	10.36282053	Up	propionate-CoA synthetase and ligase [Acanthamoeba castellanii str. Neff] >gi|440792640|gb|ELR13849.1| propionate-CoA synthetase and ligase [Acanthamoeba castellanii str. Neff]
Unigene74467_All	10.35974956	Up	malic enzyme, putative [Perkinsus marinus ATCC 50983] >gi|239890544|gb|EER12405.1| malic enzyme, putative [Perkinsus marinus ATCC 50983]
Unigene76703_All	10.35546122	Up	PREDICTED: UDP-glucose 4-epimerase-like [Megachile rotundata]
Unigene79582_All	10.35182264	Up	chloride channel, putative [Toxoplasma gondii GT1]
Unigene41275_All	10.34773209	Up	aminoimidazole ribonucleotide synthetase [Capsaspora owczarzaki ATCC 30864] >gi|320162910|gb|EFW39809.1| aminoimidazole ribonucleotide synthetase [Capsaspora owczarzaki ATCC 30864]
Unigene73252_All	10.34340782	Up	putative nucleoside-diphosphate kinase [Entodinium caudatum]
Unigene68125_All	10.34085153	Up	hypothetical protein BRAFLDRAFT_57278 [Branchiostoma floridae] >gi|229291673|gb|EEN62342.1| hypothetical protein BRAFLDRAFT_57278 [Branchiostoma floridae]
Unigene70994_All	10.33829069	Up	peroxisomal glycolate oxidase [Capsaspora owczarzaki ATCC 30864] >gi|320168155|gb|EFW45054.1| peroxisomal glycolate oxidase [Capsaspora owczarzaki ATCC 30864]
Unigene66545_All	10.33751041	Up	D-aspartate oxidase [Dictyostelium discoideum AX4] >gi|66822775|ref|XP_644742.1| D-aspartate oxidase [Dictyostelium discoideum AX4] >gi|74857668|sp|Q556W1.1|OXDD_DICDI RecName: Full=D-aspartate oxidase; Short=DASOX; AltName: Full=DDO >gi|60472631|gb|EAL70582.1| D-aspartate oxidase [Dictyostelium discoideum AX4] >gi|60472914|gb|EAL70863.1| D-aspartate oxidase [Dictyostelium discoideum AX4]
Unigene67014_All	10.33751041	Up	PREDICTED: similar to pyrroline-5-carboxylate dehydrogenase [Tribolium castaneum] >gi|270008791|gb|EFA05239.1| hypothetical protein TcasGA2_TC015385 [Tribolium castaneum]

Unigene42403_All	10.33214847	Up	glycoside hydrolase [Entamoeba dispar SAW760]
Unigene72375_All	10.33046877	Up	Putative methylmalonate-semialdehyde dehydrogenase [acylating], mitochondrial [Crassostrea gigas]
CL3647.Contig4_All	10.32957213	Up	unnamed protein product [Tetraodon nigroviridis]
Unigene72846_All	10.32631712	Up	VWFA domain-containing protein [Naegleria gruberi] >gi|284084000|gb|EFC37697.1| VWFA domain-containing protein [Naegleria gruberi]
Unigene77019_All	10.31910758	Up	PREDICTED: infertile crescent-like [Saccoglossus kowalevskii]
Unigene78168_All	10.31752567	Up	alanine dehydrogenase [Naegleria gruberi] >gi|284092621|gb|EFC46262.1| alanine dehydrogenase [Naegleria gruberi]
Unigene75148_All	10.31083996	Up	hypothetical protein [Monosiga brevicollis MX1] >gi|163773878|gb|EDQ87514.1| predicted protein [Monosiga brevicollis MX1]
Unigene77758_All	10.30993103	Up	hypothetical protein TRIADDRAFT_22852 [Trichoplax adhaerens] >gi|190586725|gb|EDV26778.1| hypothetical protein TRIADDRAFT_22852 [Trichoplax adhaerens]
Unigene77067_All	10.30480762	Up	glutaminyl-tRNA synthetase, putative [Trypanosoma brucei gambiense DAL972]
Unigene78359_All	10.30480762	Up	alcohol dehydrogenase [NADP+], partial [Clonorchis sinensis]
CL1259.Contig2_All	10.29771876	Up	Rieske iron-sulfur protein 1 [Capsaspora owczarzaki ATCC 30864] >gi|320168907|gb|EFW45806.1| Rieske iron-sulfur protein 1 [Capsaspora owczarzaki ATCC 30864]
Unigene77533_All	10.29771876	Up	PREDICTED: DNA-directed RNA polymerases I, II, and III subunit RPABC4-like [Strongylocentrotus purpuratus] >gi|390367289|ref|XP_003731218.1| PREDICTED: DNA-directed RNA polymerases I, II, and III subunit RPABC4-like [Strongylocentrotus purpuratus]
Unigene71639_All	10.29278175	Up	defender against apopototic cell death 1 [Argopecten irradians]
Unigene78719_All	10.29128583	Up	acyl-coa dehydrogenase, putative [Perkinsus marinus ATCC 50983] >gi|239890237|gb|EER12110.1| acyl-coa dehydrogenase, putative [Perkinsus marinus ATCC 50983]
Unigene76651_All	10.29071007	Up	mitochondrial aldehyde dehydrogenase 2, partial [Rousettus leschenaultii]
Unigene55343_All	10.29001885	Up	acetyl-CoA C-acetyltransferase [Polysphondylium pallidum PN500]
Unigene40586_All	10.28840471	Up	PREDICTED: dihydroxy-acid dehydratase-like [Amphimedon queenslandica]
CL7514.Contig3_All	10.2848241	Up	HAD hydrolase, family IIA subfamily protein, partial [Acanthamoeba castellanii str. Neff] >gi|440798528|gb|ELR19595.1| HAD hydrolase, family IIA subfamily protein, partial [Acanthamoeba castellanii str. Neff]

Unigene74952_All	10.27530975	Up	aldehyde dehydrogenase [Naegleria gruberi] >gi|284094598|gb|EFC48232.1| aldehyde dehydrogenase [Naegleria gruberi]
Unigene42187_All	10.27484403	Up	hypothetical protein BRAFLDRAFT_118916 [Branchiostoma floridae] >gi|229277673|gb|EEN48464.1| hypothetical protein BRAFLDRAFT_118916 [Branchiostoma floridae]
Unigene73668_All	10.27437815	Up	Ampd2 protein [Salpingoeca sp. ATCC 50818]
Unigene79272_All	10.27367906	Up	lathosterol oxidase [Danio rerio] >gi|51859008|gb|AAH81395.1| Sterol-C5-desaturase (fungal ERG3, delta-5-desaturase) homolog (S. cerevisae) [Danio rerio]
Unigene41128_All	10.26971112	Up	PREDICTED: RNA-directed DNA polymerase from mobile element jockey-like [Hydra magnipapillata]
CL1739.Contig3_All	10.26772305	Up	UDPglucose pyrophosphorylase 2 isoform, putative [Acanthamoeba castellanii str. Neff] >gi|440797781|gb|ELR18856.1| UDPglucose pyrophosphorylase 2 isoform, putative [Acanthamoeba castellanii str. Neff]
CL5452.Contig1_All	10.26772305	Up	NADH dehydrogenase [ubiquinone] 1 alpha subcomplex subunit 2 [Bombyx mori] >gi|87248185|gb|ABD36145.1| NADH-ubiquinone oxidoreductase B8 subunit [Bombyx mori]
Unigene79296_All	10.26772305	Up	PREDICTED: delta-aminolevulinic acid dehydratase-like [Anolis carolinensis]
Unigene73332_All	10.25915477	Up	PREDICTED: DNA-directed RNA polymerase II A-like [Saccoglossus kowalevskii]
Unigene73047_All	10.25833047	Up	PREDICTED: choline/ethanolamine kinase-like [Metaseiulus occidentalis]
Unigene73643_All	10.25785923	Up	CBN-NIT-1 protein [Caenorhabditis brenneri]
CL9429.Contig2_All	10.25597274	Up	putative CAD trifunctional protein, partial [Apsilocephala sp. BMW-2012]
Unigene74137_All	10.25077213	Up	predicted protein [Nematostella vectensis] >gi|156211716|gb|EDO32810.1| predicted protein [Nematostella vectensis]
Unigene75572_All	10.2500615	Up	serine palmitoyltransferase [Salpingoeca sp. ATCC 50818]
Unigene80024_All	10.24911345	Up	imidazole glycerol phosphate synthase hisHF [Salpingoeca sp. ATCC 50818]
CL9839.Contig1_All	10.2422214	Up	PREDICTED: similar to GA15370-PA [Tribolium castaneum] >gi|270008725|gb|EFA05173.1| hypothetical protein TcasGA2_TC015302 [Tribolium castaneum]
Unigene72576_All	10.23840474	Up	vacuolar proton ATPase [Danaus plexippus]
Unigene72323_All	10.23553561	Up	hypothetical protein IMG5_168370 [Ichthyophthirius multifiliis] >gi|340502084|gb|EGR28801.1| hypothetical protein IMG5_168370 [Ichthyophthirius multifiliis]
Unigene77689_All	10.23553561	Up	hypothetical protein CAPTEDRAFT_178545 [Capitella teleta]

Unigene78807_All	10.23433845	Up	diacylglycerol O-acyltransferase, putative [Acanthamoeba castellanii str. Neff] >gi|440797747|gb|ELR18824.1| diacylglycerol O-acyltransferase, putative [Acanthamoeba castellanii str. Neff]
CL7955.Contig2_All	10.23062093	Up	vacuolar H+ ATPase B subunit [Dictyostelium purpureum] >gi|325078041|gb|EGC31715.1| vacuolar H+ ATPase B subunit [Dictyostelium purpureum]
Unigene77340_All	10.23062093	Up	AMP deaminase [Dictyostelium purpureum] >gi|325083366|gb|EGC36820.1| AMP deaminase [Dictyostelium purpureum]
Unigene79156_All	10.22773626	Up	RNA polymerase III largest subunit [Ephydatia fluviatilis]
CL9169.Contig3_All	10.22532796	Up	--
Unigene45452_All	10.22037833	Up	argininosuccinate lyase [Capsaspora owczarzaki ATCC 30864] >gi|320168757|gb|EFW45656.1| argininosuccinate lyase [Capsaspora owczarzaki ATCC 30864]
Unigene52062_All	10.20701432	Up	catalase [Acanthamoeba castellanii str. Neff] >gi|372290526|gb|AEX91750.1| catalase 2 [Acanthamoeba castellanii] >gi|440792545|gb|ELR13756.1| catalase [Acanthamoeba castellanii str. Neff]
Unigene76298_All	10.20640391	Up	Glutaryl-CoA dehydrogenase, mitochondrial [Crassostrea gigas]
Unigene74759_All	10.2039597	Up	hypothetical protein [Monosiga brevicollis MX1] >gi|163772346|gb|EDQ85999.1| predicted protein [Monosiga brevicollis MX1]
Unigene72622_All	10.20200135	Up	Xanthine dehydrogenase [Acromyrmex echinatior]
CL4013.Contig2_All	10.20126628	Up	GK16804 [Drosophila willistoni] >gi|194158242|gb|EDW73143.1| GK16804 [Drosophila willistoni]
Unigene42251_All	10.19414124	Up	Bifunctional ATP-dependent dihydroxyacetone kinase/FAD-AMP lyase (cyclizing) [Crassostrea gigas]
Unigene51093_All	10.19253941	Up	Aldose reductase A [Oxytricha trifallax]
Unigene40519_All	10.18883607	Up	GI15188 [Drosophila mojavensis] >gi|193907976|gb|EDW06843.1| GI15188 [Drosophila mojavensis]
Unigene75870_All	10.18883607	Up	PREDICTED: 3-hydroxyisobutyrate dehydrogenase, mitochondrial-like [Ciona intestinalis]
Unigene77051_All	10.18883607	Up	vacuolar H+ ATPase F subunit [Dictyostelium fasciculatum] >gi|328875574|gb|EGG23938.1| vacuolar H+ ATPase F subunit [Dictyostelium fasciculatum]
CL5009.Contig1_All	10.18797059	Up	hypothetical protein BRAFLDRAFT_218241 [Branchiostoma floridae] >gi|229290998|gb|EEN61673.1| hypothetical protein BRAFLDRAFT_218241 [Branchiostoma floridae]
Unigene78056_All	10.17878932	Up	Trifunctional enzyme subunit beta, mitochondrial precursor [Salmo salar]
CL541.Contig1_All	10.17579902	Up	V-type proton ATPase subunit D [Crassostrea gigas]

<table>
<tr><td>Unigene79785_All</td><td>10.17579902</td><td>Up</td><td>UDP-glucose dehydrogenase [Capsaspora owczarzaki ATCC 30864] >gi|320163516|gb|EFW40415.1| UDP-glucose dehydrogenase [Capsaspora owczarzaki ATCC 30864]</td></tr>
<tr><td>Unigene76772_All</td><td>10.17305246</td><td>Up</td><td>[Acyl-carrier protein] S-malonyltransferase [Acanthamoeba castellanii str. Neff] >gi|440789973|gb|ELR11263.1| [Acyl-carrier protein] S-malonyltransferase [Acanthamoeba castellanii str. Neff]</td></tr>
<tr><td>Unigene78806_All</td><td>10.17280251</td><td>Up</td><td>hypothetical protein TRIADDRAFT_29701 [Trichoplax adhaerens] >gi|190581954|gb|EDV22029.1| hypothetical protein TRIADDRAFT_29701 [Trichoplax adhaerens]</td></tr>
<tr><td>Unigene74976_All</td><td>10.15494466</td><td>Up</td><td>branched chain keto acid dehydrogenase E1 [Capsaspora owczarzaki ATCC 30864] >gi|320167657|gb|EFW44556.1| branched chain keto acid dehydrogenase E1 [Capsaspora owczarzaki ATCC 30864]</td></tr>
<tr><td>Unigene75390_All</td><td>10.15367869</td><td>Up</td><td>hypothetical protein [Monosiga brevicollis MX1] >gi|163777428|gb|EDQ91045.1| predicted protein [Monosiga brevicollis MX1]</td></tr>
<tr><td>Unigene77433_All</td><td>10.1463141</td><td>Up</td><td>cytochrome b2 [Salpingoeca sp. ATCC 50818]</td></tr>
<tr><td>Unigene77811_All</td><td>10.14274528</td><td>Up</td><td>hypothetical protein [Monosiga brevicollis MX1] >gi|163773715|gb|EDQ87353.1| predicted protein [Monosiga brevicollis MX1]</td></tr>
<tr><td>Unigene74538_All</td><td>10.13724734</td><td>Up</td><td>V-type proton ATPase subunit D [Crassostrea gigas]</td></tr>
<tr><td>Unigene71622_All</td><td>10.12476754</td><td>Up</td><td>glyceraldehyde 3-phosphate dehydrogenase [Capsaspora owczarzaki ATCC 30864] >gi|320163540|gb|EFW40439.1| glyceraldehyde 3-phosphate dehydrogenase [Capsaspora owczarzaki ATCC 30864]</td></tr>
<tr><td>Unigene72756_All</td><td>10.12295738</td><td>Up</td><td>hypothetical protein TRIADDRAFT_49690 [Trichoplax adhaerens] >gi|190589542|gb|EDV29564.1| hypothetical protein TRIADDRAFT_49690 [Trichoplax adhaerens]</td></tr>
<tr><td>Unigene78711_All</td><td>10.11517373</td><td>Up</td><td>PREDICTED: long chain base biosynthesis protein 1-like [Gorilla gorilla gorilla]</td></tr>
<tr><td>Unigene78810_All</td><td>10.11217883</td><td>Up</td><td>15-hydroxyprostaglandin DH NAD+-like protein [Anasa tristis]</td></tr>
<tr><td>Unigene74528_All</td><td>10.08759544</td><td>Up</td><td>conserved hypothetical protein [Perkinsus marinus ATCC 50983] >gi|239904298|gb|EER20553.1| conserved hypothetical protein [Perkinsus marinus ATCC 50983]</td></tr>
<tr><td>Unigene77933_All</td><td>10.0744091</td><td>Up</td><td>Spermidine synthase [Crassostrea gigas]</td></tr>
<tr><td>Unigene51095_All</td><td>10.07240061</td><td>Up</td><td>hypothetical protein KGM_09336 [Danaus plexippus]</td></tr>
<tr><td>Unigene78586_All</td><td>10.06931549</td><td>Up</td><td>PREDICTED: dihydrofolate reductase-like [Metaseiulus occidentalis]</td></tr>
</table>

Unigene76742_All	10.06326024	Up	PREDICTED: long-chain-fatty-acid--CoA ligase 4 isoform 2 [Equus caballus]
Unigene77459_All	10.05812705	Up	RNA polymerase II largest subunit [Amoeboaphelidium protococcarum]
Unigene79359_All	10.04289053	Up	Porphobilinogen deaminase [Salmo salar]
Unigene78863_All	10.03576136	Up	hypothetical protein CAPTEDRAFT_177698 [Capitella teleta]
Unigene79333_All	10.03273453	Up	PREDICTED: AMP deaminase 2-like, partial [Hydra magnipapillata]
Unigene45709_All	10.0250011	Up	Protein CYP-13A10 [Caenorhabditis elegans] >gi|54649873|emb|CAA87042.3| Protein CYP-13A10 [Caenorhabditis elegans]
Unigene43117_All	10.01959073	Up	PREDICTED: aldehyde dehydrogenase family 3 member B1-like [Takifugu rubripes]
CL5158.Contig2_All	10.00772811	Up	phosphatidylinositol synthase [Capsaspora owczarzaki ATCC 30864] >gi|320164590|gb|EFW41489.1| phosphatidylinositol synthase [Capsaspora owczarzaki ATCC 30864]
CL3647.Contig1_All	9.991663558	Up	Succinate dehydrogenase iron-sulfur subunit, mitochondrial precursor [Anoplopoma fimbria]
Unigene77355_All	9.971974574	Up	aconitate hydratase, mitochondrial, putative [Acanthamoeba castellanii str. Neff] >gi|440799601|gb|ELR20645.1| aconitate hydratase, mitochondrial, putative [Acanthamoeba castellanii str. Neff]
Unigene78638_All	9.963763096	Up	hypothetical protein CAPTEDRAFT_228579 [Capitella teleta]
CL5787.Contig1_All	9.960291601	Up	predicted protein [Naegleria gruberi] >gi|284097080|gb|EFC50707.1| predicted protein [Naegleria gruberi]
CL1540.Contig2_All	9.952304442	Up	V-type H+-transporting ATPase subunit A [Clonorchis sinensis]
CL3647.Contig3_All	9.933543133	Up	Succinate dehydrogenase iron-sulfur subunit, mitochondrial precursor [Anoplopoma fimbria]
CL7239.Contig1_All	9.877437522	Up	fumarate hydratase, class II, putative [Acanthamoeba castellanii str. Neff] >gi|440793686|gb|ELR14864.1| fumarate hydratase, class II, putative [Acanthamoeba castellanii str. Neff]
CL1052.Contig1_All	9.853933289	Up	NAD-specific glutamate dehydrogenase [Capsaspora owczarzaki ATCC 30864] >gi|320170749|gb|EFW47648.1| NAD-specific glutamate dehydrogenase [Capsaspora owczarzaki ATCC 30864]
CL4690.Contig1_All	9.836050355	Up	hypothetical protein PTSG_07232 [Salpingoeca sp. ATCC 50818]
CL4829.Contig1_All	9.783980414	Up	hypothetical protein DAPPUDRAFT_307444 [Daphnia pulex]
CL6528.Contig1_All	9.744665681	Up	PREDICTED: pantothenate kinase 2, mitochondrial-like [Apis florea]
Unigene53554_All	9.737923316	Up	PREDICTED: dolichol-phosphate mannosyltransferase [Sarcophilus harrisii]
Unigene80166_All	9.716819461	Up	hypothetical protein AND_07334 [Anopheles darlingi]

CL7179.Contig1_All	9.601027897	Up	PREDICTED: peroxisomal acyl-coenzyme A oxidase 2 [Taeniopygia guttata]
CL6512.Contig3_All	9.519636253	Up	PREDICTED: phosphatidate phosphatase PPAPDC1B-like [Loxodonta africana]
CL7782.Contig2_All	9.494455614	Up	squalene monooxygenase [Capsaspora owczarzaki ATCC 30864] >gi|320165555|gb|EFW42454.1| squalene monooxygenase [Capsaspora owczarzaki ATCC 30864]
CL2544.Contig3_All	9.412781525	Up	CS domain-containing protein [Capsaspora owczarzaki ATCC 30864] >gi|320163723|gb|EFW40622.1| CS domain-containing protein [Capsaspora owczarzaki ATCC 30864]
CL6512.Contig1_All	9.354910512	Up	PREDICTED: phosphatidate phosphatase PPAPDC1B-like [Loxodonta africana]
CL6956.Contig2_All	9.286999211	Up	unknown [Dendroctonus ponderosae]
CL2544.Contig4_All	9.147713722	Up	CS domain-containing protein [Capsaspora owczarzaki ATCC 30864] >gi|320163723|gb|EFW40622.1| CS domain-containing protein [Capsaspora owczarzaki ATCC 30864]
CL7231.Contig1_All	9.071998581	Up	Alkaline ceramidase [Harpegnathos saltator]
CL119.Contig2_All	9.068778278	Up	PREDICTED: similar to CG10320 CG10320-PB [Tribolium castaneum] >gi|270010991|gb|EFA07439.1| hypothetical protein TcasGA2_TC008873 [Tribolium castaneum]
CL5387.Contig2_All	9.043027284	Up	unnamed protein product [Oikopleura dioica]
CL2812.Contig1_All	9.03039126	Up	PREDICTED: piggyBac transposable element-derived protein 3-like [Megachile rotundata]
CL692.Contig1_All	9.019312726	Up	PREDICTED: vanin-like protein 1-like isoform 2 [Acyrthosiphon pisum] >gi|328701752|ref|XP_001947951.2| PREDICTED: vanin-like protein 1-like isoform 1 [Acyrthosiphon pisum]
CL6466.Contig2_All	8.948498481	Up	fructose-1-6-bisphosphatase [Capsaspora owczarzaki ATCC 30864] >gi|320168762|gb|EFW45661.1| fructose-1-6-bisphosphatase [Capsaspora owczarzaki ATCC 30864]
CL4475.Contig4_All	8.879276912	Up	PREDICTED: similar to juvenile hormone esterase [Tribolium castaneum] >gi|270008042|gb|EFA04490.1| hypothetical protein TcasGA2_TC014795 [Tribolium castaneum]
CL1459.Contig1_All	8.872520985	Up	PREDICTED: V-type proton ATPase subunit e 2-like [Nasonia vitripennis]
CL2544.Contig1_All	8.7370783	Up	CS domain-containing protein [Capsaspora owczarzaki ATCC 30864] >gi|320163723|gb|EFW40622.1| CS domain-containing protein [Capsaspora owczarzaki ATCC 30864]
CL2268.Contig3_All	8.718410557	Up	phosphoesterase [Dictyostelium fasciculatum] >gi|328867956|gb|EGG16337.1| phosphoesterase [Dictyostelium fasciculatum]

CL6454.Contig1_All	8.692441147	Up	PREDICTED: choline/ethanolaminephosphotransferase 1-like isoform 2 [Bombus impatiens]
CL5340.Contig1_All	8.625387397	Up	GK10285 [Drosophila willistoni] >gi|194157263|gb|EDW72164.1| GK10285 [Drosophila willistoni]
Unigene43423_All	8.448260595	Up	uricase [Acanthamoeba castellanii str. Neff] >gi|440794103|gb|ELR15274.1| uricase [Acanthamoeba castellanii str. Neff]
CL1204.Contig1_All	8.446669787	Up	PREDICTED: ethanolamine-phosphate cytidylyltransferase-like [Nasonia vitripennis]
CL2263.Contig2_All	8.446417072	Up	PREDICTED: predicted protein-like [Saccoglossus kowalevskii]
CL1739.Contig1_All	8.381808554	Up	UDPglucose pyrophosphorylase 2 isoform, putative [Acanthamoeba castellanii str. Neff] >gi|440797781|gb|ELR18856.1| UDPglucose pyrophosphorylase 2 isoform, putative [Acanthamoeba castellanii str. Neff]
CL5669.Contig1_All	8.268257416	Up	succinate-CoA ligase [Capsaspora owczarzaki ATCC 30864] >gi|320166382|gb|EFW43281.1| succinate-CoA ligase [Capsaspora owczarzaki ATCC 30864]
CL3059.Contig2_All	8.266725443	Up	HAD-superfamily hydrolase [Capsaspora owczarzaki ATCC 30864] >gi|320168056|gb|EFW44955.1| HAD-superfamily hydrolase [Capsaspora owczarzaki ATCC 30864]
CL1556.Contig3_All	8.227296907	Up	porphobilinogen deaminase [Capsaspora owczarzaki ATCC 30864] >gi|320168193|gb|EFW45092.1| porphobilinogen deaminase [Capsaspora owczarzaki ATCC 30864]
CL9510.Contig2_All	8.22468689	Up	ornithine-oxo-acid transaminase [Dictyostelium purpureum] >gi|325074089|gb|EGC28248.1| ornithine-oxo-acid transaminase [Dictyostelium purpureum]
Unigene41338_All	8.217939251	Up	3-hydroxyacyl-CoA dehydrogenase [Capsaspora owczarzaki ATCC 30864] >gi|320162945|gb|EFW39844.1| 3-hydroxyacyl-CoA dehydrogenase [Capsaspora owczarzaki ATCC 30864]
Unigene42052_All	8.188588846	Up	PREDICTED: ornithine decarboxylase [Monodelphis domestica]
CL2979.Contig1_All	8.169791412	Up	glutamine synthetase [Paracentrotus lividus]
CL476.Contig2_All	8.166581558	Up	C-4 methyl sterol oxidase [Dictyostelium discoideum AX4] >gi|60474307|gb|EAL72244.1| C-4 methyl sterol oxidase [Dictyostelium discoideum AX4]
CL8364.Contig1_All	8.149057374	Up	GH13345 [Drosophila grimshawi] >gi|193900042|gb|EDV98908.1| GH13345 [Drosophila grimshawi]
CL7537.Contig1_All	8.139727753	Up	PREDICTED: NADH dehydrogenase [ubiquinone] iron-sulfur protein 3, mitochondrial-like [Acyrthosiphon pisum]
CL8253.Contig1_All	8.077355831	Up	PREDICTED: NADH-ubiquinone oxidoreductase 75 kDa subunit, mitochondrial-like isoform 1

			[Strongylocentrotus purpuratus]
CL7532.Contig2_All	8.066972315	Up	predicted protein [Naegleria gruberi] >gi|284092276|gb|EFC45919.1| predicted protein [Naegleria gruberi]
CL1819.Contig1_All	7.927257588	Up	isocitrate dehydrogenase [Aedes aegypti] >gi|108884328|gb|EAT48553.1| AAEL000454-PA [Aedes aegypti]
Unigene55712_All	7.911714371	Up	GJ18479 [Drosophila virilis] >gi|194141032|gb|EDW57458.1| GJ18479 [Drosophila virilis]
Unigene57888_All	7.911023055	Up	acetyl-CoA C-acetyltransferase [Capsaspora owczarzaki ATCC 30864] >gi|320163922|gb|EFW40821.1| acetyl-CoA C-acetyltransferase [Capsaspora owczarzaki ATCC 30864]
CL2290.Contig2_All	7.880276603	Up	glyceraldehyde-3-phosphate dehydrogenase [Eimeria tenella]
CL2695.Contig1_All	7.880233359	Up	PREDICTED: NADH dehydrogenase [ubiquinone] 1 beta subcomplex subunit 8, mitochondrial-like [Hydra magnipapillata]
Unigene42154_All	7.859312919	Up	sulfate adenylyltransferase [Acanthamoeba castellanii str. Neff] >gi|440795708|gb|ELR16825.1| sulfate adenylyltransferase [Acanthamoeba castellanii str. Neff]
Unigene56937_All	7.846685436	Up	GF17416 [Drosophila ananassae] >gi|190619892|gb|EDV35416.1| GF17416 [Drosophila ananassae]
Unigene49309_All	7.845692378	Up	hypothetical protein DAPPUDRAFT_230594 [Daphnia pulex]
CL8293.Contig1_All	7.729788795	Up	asparagine synthase (glutaminehydrolyzing), putative [Acanthamoeba castellanii str. Neff] >gi|440792442|gb|ELR13664.1| asparagine synthase (glutaminehydrolyzing), putative [Acanthamoeba castellanii str. Neff]
Unigene56930_All	7.720039834	Up	predicted protein [Nematostella vectensis] >gi|156220587|gb|EDO41453.1| predicted protein [Nematostella vectensis]
Unigene56337_All	7.719086781	Up	hypothetical protein [Monosiga brevicollis MX1] >gi|163778558|gb|EDQ92173.1| predicted protein [Monosiga brevicollis MX1]
CL3103.Contig1_All	7.709864268	Up	hypothetical protein TRIADDRAFT_29716 [Trichoplax adhaerens] >gi|190581814|gb|EDV21889.1| hypothetical protein TRIADDRAFT_29716 [Trichoplax adhaerens]
CL3411.Contig2_All	7.696314046	Up	Chain J, Orientation Of Rna Polymerase Ii Within The Human Vp16-Mediator-Pol Ii-Tfiif Assembly
Unigene42143_All	7.679155254	Up	PREDICTED: 6-phosphofructokinase, liver type [Ovis aries]
Unigene56399_All	7.636691498	Up	hypothetical protein BRAFLDRAFT_126490 [Branchiostoma floridae] >gi|229291503|gb|EEN62174.1| hypothetical protein BRAFLDRAFT_126490 [Branchiostoma floridae]
CL3755.Contig2_All	7.627205262	Up	surface protein with EGF domain, putative [Tetrahymena thermophila] >gi|89288293|gb|EAR86281.1|

			surface protein with EGF domain, putative [Tetrahymena thermophila SB210]
CL5861.Contig1_All	7.626439137	Up	PREDICTED: sphingomyelin synthase-related 1-like [Nasonia vitripennis]
CL3867.Contig1_All	7.60051654	Up	PREDICTED: cytochrome c oxidase subunit 6A1, mitochondrial [Apis mellifera]
CL2751.Contig1_All	7.584036806	Up	enolase 3 [Salpingoeca sp. ATCC 50818]
CL9789.Contig2_All	7.570583533	Up	glycerol kinase [Acanthamoeba castellanii str. Neff] >gi|440793310|gb|ELR14497.1| glycerol kinase [Acanthamoeba castellanii str. Neff]
CL3938.Contig1_All	7.561779725	Up	mannitol-1-phosphate dehydrogenase-like protein [Physarum polycephalum]
Unigene57976_All	7.547136794	Up	PREDICTED: glucokinase-like [Cricetulus griseus]
CL3357.Contig1_All	7.53117441	Up	GF22866 [Drosophila ananassae] >gi|190615050|gb|EDV30574.1| GF22866 [Drosophila ananassae]
Unigene55989_All	7.51910728	Up	serine-repeat antigen [Plasmodium cynomolgi strain B] >gi|390371137|dbj|GAB65018.1| serine-repeat antigen [Plasmodium cynomolgi strain B]
CL1052.Contig2_All	7.508587112	Up	NAD-specific glutamate dehydrogenase [Capsaspora owczarzaki ATCC 30864] >gi|320170749|gb|EFW47648.1| NAD-specific glutamate dehydrogenase [Capsaspora owczarzaki ATCC 30864]
CL3824.Contig2_All	7.497766316	Up	hypothetical protein [Monosiga brevicollis MX1] >gi|163778508|gb|EDQ92123.1| predicted protein [Monosiga brevicollis MX1]
CL9268.Contig1_All	7.490131341	Up	Glutamate 5-kinase, putative [Perkinsus marinus ATCC 50983] >gi|239885762|gb|EER09595.1| Glutamate 5-kinase, putative [Perkinsus marinus ATCC 50983]
Unigene57078_All	7.481645031	Up	PREDICTED: acyl-CoA synthetase long-chain family member 5-like [Saccoglossus kowalevskii]
CL3202.Contig4_All	7.455902979	Up	transketolase [Salpingoeca sp. ATCC 50818]
CL5743.Contig1_All	7.439127783	Up	PREDICTED: peroxisomal multifunctional enzyme type 2 [Strongylocentrotus purpuratus]
CL548.Contig1_All	7.429309094	Up	hypothetical protein [Monosiga brevicollis MX1] >gi|163775779|gb|EDQ89402.1| predicted protein [Monosiga brevicollis MX1]
Unigene51206_All	7.428795979	Up	PREDICTED: similar to retinol dehydrogenase 11 [Tribolium castaneum] >gi|270013154|gb|EFA09602.1| hypothetical protein TcasGA2_TC011722 [Tribolium castaneum]
CL2729.Contig2_All	7.427824966	Up	predicted protein [Nematostella vectensis] >gi|156213633|gb|EDO34645.1| predicted protein [Nematostella vectensis]
Unigene42156_All	7.420022121	Up	hypothetical protein BRAFLDRAFT_278137 [Branchiostoma floridae] >gi|229289578|gb|EEN60264.1|

			hypothetical protein BRAFLDRAFT_278137 [Branchiostoma floridae]
Unigene56014_All	7.401692222	Up	Man1b1 protein [Rattus norvegicus]
Unigene53073_All	7.401270375	Up	Cytochrome c oxidase assembly protein COX11, mitochondrial [Heterocephalus glaber]
CL6396.Contig2_All	7.396868783	Up	PREDICTED: cytochrome b-c1 complex subunit 2, mitochondrial-like [Strongylocentrotus purpuratus]
CL844.Contig1_All	7.372463279	Up	NADH dehydrogenase [ubiquinone] iron-sulfur protein 7, mitochondrial [Rattus norvegicus] >gi|55824717|gb|AAH86574.1| NADH dehydrogenase (ubiquinone) Fe-S protein 7 [Rattus norvegicus] >gi|149034571|gb|EDL89308.1| NADH dehydrogenase (ubiquinone) Fe-S protein 7, isoform CRA_d [Rattus norvegicus]
Unigene54766_All	7.371322895	Up	palmitoyl-protein thioesterase 1 [Polysphondylium pallidum PN500]
Unigene41052_All	7.367640092	Up	succinate dehydrogenase flavoprotein subunit [Capsaspora owczarzaki ATCC 30864] >gi|320166305|gb|EFW43204.1| succinate dehydrogenase flavoprotein subunit [Capsaspora owczarzaki ATCC 30864]
Unigene41431_All	7.351497304	Up	hypothetical protein EGM_05033 [Macaca fascicularis]
CL1289.Contig2_All	7.350148765	Up	PREDICTED: LOW QUALITY PROTEIN: 6-phosphogluconolactonase-like [Apis florea]
Unigene57446_All	7.349863792	Up	PREDICTED: cytochrome P450 2U1-like [Strongylocentrotus purpuratus]
CL541.Contig2_All	7.327515631	Up	vacuolar ATPase subunit D [Capsaspora owczarzaki ATCC 30864] >gi|320167077|gb|EFW43976.1| vacuolar ATPase subunit D [Capsaspora owczarzaki ATCC 30864]
CL3938.Contig2_All	7.323006689	Up	CBN-SODH-2 protein [Caenorhabditis brenneri]
CL3755.Contig1_All	7.307595434	Up	surface protein with EGF domain, putative [Tetrahymena thermophila] >gi|89288293|gb|EAR86281.1| surface protein with EGF domain, putative [Tetrahymena thermophila SB210]
Unigene40833_All	7.299993719	Up	hypothetical protein DAPPUDRAFT_319968 [Daphnia pulex]
CL6064.Contig2_All	7.287898898	Up	mannose-1-phosphate guanyltransferase beta [Capsaspora owczarzaki ATCC 30864] >gi|320169204|gb|EFW46103.1| mannose-1-phosphate guanyltransferase beta [Capsaspora owczarzaki ATCC 30864]
Unigene42015_All	7.277042637	Up	glucose-6-phosphate 1-dehydrogenase [Capsaspora owczarzaki ATCC 30864] >gi|320168424|gb|EFW45323.1| glucose-6-phosphate 1-dehydrogenase [Capsaspora owczarzaki ATCC 30864]
CL5961.Contig1_All	7.266696891	Up	enoyl-CoA hydratase [Dictyostelium purpureum] >gi|325083300|gb|EGC36756.1| enoyl-CoA hydratase

			[Dictyostelium purpureum]
CL6812.Contig1_All	7.260500744	Up	Peroxisomal multifunctional enzyme type 2 [Crassostrea gigas]
Unigene40787_All	7.232760739	Up	PREDICTED: guanylate kinase-like isoform 1 [Nasonia vitripennis]
Unigene57464_All	7.226375365	Up	gamma-glutamyl phosphate reductase, putative [Toxoplasma gondii ME49] >gi|211963385|gb|EEA98580.1| gamma-glutamyl phosphate reductase, putative [Toxoplasma gondii ME49] >gi|221488178|gb|EEE26392.1| gamma-glutamyl phosphate reductase, putative [Toxoplasma gondii GT1] >gi|221508691|gb|EEE34260.1| gamma-glutamyl phosphate reductase, putative [Toxoplasma gondii VEG]
CL84.Contig2_All	7.216658216	Up	AGAP011050-PA [Anopheles gambiae str. PEST] >gi|116132724|gb|EAA45511.3| AGAP011050-PA [Anopheles gambiae str. PEST]
CL5990.Contig1_All	7.215922958	Up	PREDICTED: gluconate 5-dehydrogenase-like [Xenopus (Silurana) tropicalis]
CL5366.Contig2_All	7.215487072	Up	predicted protein [Nematostella vectensis] >gi|156228565|gb|EDO49363.1| predicted protein [Nematostella vectensis]
CL2998.Contig2_All	7.203065592	Up	predicted protein [Naegleria gruberi] >gi|284091150|gb|EFC44798.1| predicted protein [Naegleria gruberi]
CL1763.Contig1_All	7.198114761	Up	PREDICTED: ATP-citrate synthase-like [Amphimedon queenslandica]
Unigene57498_All	7.197604706	Up	PREDICTED: cytochrome b-c1 complex subunit 2, mitochondrial-like [Oryzias latipes]
CL5377.Contig1_All	7.194835365	Up	ATP synthase b [Spodoptera exigua]
Unigene56733_All	7.171104616	Up	PREDICTED: hypothetical protein LOC100147904 [Danio rerio]
CL2399.Contig1_All	7.161960291	Up	tryptophan synthase alpha chain, putative [Acanthamoeba castellanii str. Neff] >gi|440800106|gb|ELR21149.1| tryptophan synthase alpha chain, putative [Acanthamoeba castellanii str. Neff]
CL4029.Contig2_All	7.150746912	Up	4-hydroxyphenylpyruvate dioxygenase [Papilio xuthus]
Unigene41574_All	7.149777089	Up	PREDICTED: probable fumarate hydratase, mitochondrial-like isoform 1 [Ciona intestinalis] >gi|459176367|ref|XP_004226066.1| PREDICTED: probable fumarate hydratase, mitochondrial-like [Ciona intestinalis]
CL2472.Contig1_All	7.118954112	Up	hypothetical protein [Monosiga brevicollis MX1] >gi|163771876|gb|EDQ85537.1| predicted protein [Monosiga brevicollis MX1]
Unigene58263_All	7.112824923	Up	pyrroline carboxylate reductase, putative [Perkinsus marinus ATCC

			50983] >gi|239868611|gb|EEQ99879.1| pyrroline carboxylate reductase, putative [Perkinsus marinus ATCC 50983]
Unigene42145_All	7.092672099	Up	PREDICTED: glucosamine-6-phosphate isomerase 1 isoform 1 [Ovis aries]
CL1310.Contig2_All	7.078974539	Up	PREDICTED: ATP synthase subunit beta, mitochondrial-like [Oryzias latipes]
CL3503.Contig1_All	7.074676686	Up	PREDICTED: uridine-cytidine kinase-like 1-like isoform 1 [Bombus terrestris] >gi|340720303|ref|XP_003398580.1| PREDICTED: uridine-cytidine kinase-like 1-like isoform 2 [Bombus terrestris]
CL5650.Contig1_All	7.063421519	Up	PREDICTED: UDP-N-acetylhexosamine pyrophosphorylase-like [Meleagris gallopavo]
Unigene57920_All	7.063176419	Up	acylCoA dehydrogenase, middle domain containing protein [Acanthamoeba castellanii str. Neff] >gi|440801832|gb|ELR22836.1| acylCoA dehydrogenase, middle domain containing protein [Acanthamoeba castellanii str. Neff]
Unigene56501_All	7.055867826	Up	PREDICTED: membrane primary amine oxidase-like [Anolis carolinensis]
Unigene57524_All	7.053004975	Up	transketolase [Capsaspora owczarzaki ATCC 30864] >gi|320170582|gb|EFW47481.1| transketolase [Capsaspora owczarzaki ATCC 30864]
Unigene42565_All	7.050648549	Up	hypothetical protein CRE_07734 [Caenorhabditis remanei] >gi|308269155|gb|EFP13108.1| hypothetical protein CRE_07734 [Caenorhabditis remanei]
CL5438.Contig1_All	7.041820176	Up	PREDICTED: dihydrolipoyl dehydrogenase, mitochondrial-like [Amphimedon queenslandica]
CL983.Contig1_All	7.031329458	Up	Rieske iron-sulfur protein 1 [Capsaspora owczarzaki ATCC 30864] >gi|320168907|gb|EFW45806.1| Rieske iron-sulfur protein 1 [Capsaspora owczarzaki ATCC 30864]
CL9458.Contig1_All	7.025646014	Up	4-aminobutyrate transaminase [Dictyostelium discoideum AX4] >gi|74897469|sp|Q55FI1.1|GABT_DICDI RecName: Full=4-aminobutyrate aminotransferase; AltName: Full=GABA aminotransferase; Short=GABA-AT; AltName: Full=Gamma-amino-N-butyrate transaminase; Short=GABA transaminase >gi|60475570|gb|EAL73505.1| 4-aminobutyrate transaminase [Dictyostelium discoideum AX4]
CL1028.Contig1_All	7.020411026	Up	putative ATPase H+ transporting lysosomal 31 kDa V1 subunit E [Taeniopygia guttata] >gi|197127345|gb|ACH43843.1| putative ATPase H+ transporting lysosomal 31 kDa V1 subunit E [Taeniopygia guttata] >gi|197127346|gb|ACH43844.1| putative ATPase H+ transporting lysosomal 31 kDa V1 subunit E [Taeniopygia guttata] >gi|197127347|gb|ACH43845.1| putative ATPase H+

			transporting lysosomal 31 kDa V1 subunit E [Taeniopygia guttata] >gi|197127348|gb|ACH43846.1| putative ATPase H+ transporting lysosomal 31 kDa V1 subunit E [Taeniopygia guttata]
Unigene56080_All	7.014470483	Up	NADH dehydrogenase [ubiquinone] flavoprotein 1, mitochondrial [Danio rerio] >gi|50925098|gb|AAH78648.1| NADH dehydrogenase (ubiquinone) flavoprotein 1 [Danio rerio]
CL1274.Contig2_All	7.011915184	Up	ATP synthase subunit gamma [Capsaspora owczarzaki ATCC 30864] >gi|320169247|gb|EFW46146.1| ATP synthase subunit gamma [Capsaspora owczarzaki ATCC 30864]
CL7514.Contig1_All	7.011328233	Up	HAD-superfamily hydrolase [Capsaspora owczarzaki ATCC 30864] >gi|320168056|gb|EFW44955.1| HAD-superfamily hydrolase [Capsaspora owczarzaki ATCC 30864]
Unigene58239_All	7.002839757	Up	DNA-directed RNA polymerase I subunit D [Tetrahymena thermophila] >gi|89290022|gb|EAR88010.1| DNA-directed RNA polymerase I subunit D [Tetrahymena thermophila SB210]
CL6260.Contig1_All	6.984824077	Up	C30D10.14 [Salmo salar] >gi|221221092|gb|ACM09207.1| C30D10.14 [Salmo salar]
Unigene57980_All	6.983139781	Up	D-aspartate oxidase [Dictyostelium discoideum AX4] >gi|66822775|ref|XP_644742.1| D-aspartate oxidase [Dictyostelium discoideum AX4] >gi|74857668|sp|Q556W1.1|OXDD_DICDI RecName: Full=D-aspartate oxidase; Short=DASOX; AltName: Full=DDO >gi|60472631|gb|EAL70582.1| D-aspartate oxidase [Dictyostelium discoideum AX4] >gi|60472914|gb|EAL70863.1| D-aspartate oxidase [Dictyostelium discoideum AX4]
CL3946.Contig2_All	6.975656546	Up	glucose-6-phosphate isomerase [Nilaparvata lugens]
CL5390.Contig1_All	6.9726233	Up	PREDICTED: uncharacterized short-chain type dehydrogenase/reductase y4vI-like [Xenopus (Silurana) tropicalis]
CL7418.Contig1_All	6.970974979	Up	adenosylmethionine decarboxylase [Acanthamoeba castellanii str. Neff] >gi|440794734|gb|ELR15889.1| adenosylmethionine decarboxylase [Acanthamoeba castellanii str. Neff]
CL9510.Contig1_All	6.940708879	Up	ornithine-oxo-acid transaminase [Dictyostelium purpureum] >gi|325074089|gb|EGC28248.1| ornithine-oxo-acid transaminase [Dictyostelium purpureum]
Unigene57668_All	6.940054857	Up	RecName: Full=2-oxoglutarate dehydrogenase, mitochondrial; AltName: Full=2-oxoglutarate dehydrogenase complex component E1; Short=OGDC-E1; AltName: Full=Alpha-ketoglutarate dehydrogenase; Flags: Precursor
CL1906.Contig1_All	6.936265363	Up	hypothetical protein F09E5.2 - Caenorhabditis elegans
CL2614.Contig1_All	6.936200437	Up	NADH dehydrogenase, putative [Acanthamoeba castellanii str. Neff] >gi|440796531|gb|ELR17640.1|

			NADH dehydrogenase, putative [Acanthamoeba castellanii str. Neff]
CL2358.Contig1_All	6.935430848	Up	predicted protein [Naegleria gruberi] >gi|284093417|gb|EFC47055.1| predicted protein [Naegleria gruberi]
CL9806.Contig1_All	6.930559692	Up	hypothetical protein [Monosiga brevicollis MX1] >gi|163770700|gb|EDQ84382.1| predicted protein [Monosiga brevicollis MX1]
Unigene42917_All	6.930447412	Up	vacuolar ATPase G subunit [Antheraea yamamai]
CL6089.Contig1_All	6.918423384	Up	GE24304 [Drosophila yakuba] >gi|194183848|gb|EDW97459.1| GE24304 [Drosophila yakuba]
CL8829.Contig2_All	6.907950251	Up	GF17072 [Drosophila ananassae] >gi|190626792|gb|EDV42316.1| GF17072 [Drosophila ananassae]
CL5787.Contig2_All	6.90742615	Up	predicted protein [Naegleria gruberi] >gi|284097080|gb|EFC50707.1| predicted protein [Naegleria gruberi]
Unigene58223_All	6.895847892	Up	PREDICTED: acetyl-CoA acetyltransferase A, mitochondrial-like [Meleagris gallopavo]
CL3206.Contig1_All	6.879473303	Up	triosephosphate isomerase, putative [Pediculus humanus corporis] >gi|212510370|gb|EEB13564.1| triosephosphate isomerase, putative [Pediculus humanus corporis]
CL2488.Contig1_All	6.874032562	Up	neuron-specific enolase [Capsaspora owczarzaki ATCC 30864] >gi|320170655|gb|EFW47554.1| neuron-specific enolase [Capsaspora owczarzaki ATCC 30864]
CL6816.Contig1_All	6.869553259	Up	PREDICTED: dihydrolipoyllysine-residue acetyltransferase component of pyruvate dehydrogenase complex, mitochondrial [Taeniopygia guttata]
CL2779.Contig1_All	6.869349388	Up	PREDICTED: ATP synthase subunit delta, mitochondrial-like [Megachile rotundata]
Unigene10295_All	6.867178193	Up	PREDICTED: probable NADP-dependent mannitol dehydrogenase-like, partial [Acyrthosiphon pisum]
CL5772.Contig1_All	6.861944809	Up	pro-phenoloxidase 1 [Locusta migratoria]
CL1319.Contig1_All	6.85774545	Up	chloride channel protein k, putative [Toxoplasma gondii VEG]
CL9290.Contig1_All	6.85488823	Up	unnamed protein product, partial [Leishmania mexicana MHOM/GT/2001/U1103] >gi|356491280|emb|CBZ41020.1| unnamed protein product, partial [Leishmania mexicana MHOM/GT/2001/U1103]
Unigene50515_All	6.849556411	Up	pyrroline carboxylate reductase, putative [Perkinsus marinus ATCC 50983] >gi|239868611|gb|EEQ99879.1| pyrroline carboxylate reductase, putative [Perkinsus marinus ATCC 50983]
Unigene41967_All	6.842415824	Up	Protein NIT-1 [Caenorhabditis elegans] >gi|3881500|emb|CAA84681.1| Protein NIT-1 [Caenorhabditis elegans]
CL2350.Contig2_All	6.841190479	Up	nucleoside diphosphate kinase B [Haliotis discus discus]

CL8798.Contig1_All	6.829775095	Up	PREDICTED: ATP synthase subunit O, mitochondrial-like [Takifugu rubripes]
CL5688.Contig2_All	6.807726894	Up	Protein T25B9.9 [Caenorhabditis elegans] >gi|74962414|sp|Q17761.2|6PGD_CAEEL RecName: Full=6-phosphogluconate dehydrogenase, decarboxylating >gi|3880188|emb|CAA94380.1| Protein T25B9.9 [Caenorhabditis elegans]
Unigene51030_All	6.806561296	Up	predicted protein [Nematostella vectensis] >gi|156219968|gb|EDO40842.1| predicted protein [Nematostella vectensis]
Unigene41498_All	6.806392805	Up	arginase [Capsaspora owczarzaki ATCC 30864] >gi|320170473|gb|EFW47372.1| arginase [Capsaspora owczarzaki ATCC 30864]
Unigene40461_All	6.800850324	Up	succinate dehydrogenase flavoprotein subunit [Capsaspora owczarzaki ATCC 30864] >gi|320166305|gb|EFW43204.1| succinate dehydrogenase flavoprotein subunit [Capsaspora owczarzaki ATCC 30864]
CL6686.Contig1_All	6.784140962	Up	DNA-directed RNA polymerase II largest subunit [Loa loa]
Unigene57259_All	6.782014343	Up	cytochrome c oxidase subunit VIa/COX13 [Aedes aegypti] >gi|108881257|gb|EAT45482.1| AAEL003234-PA [Aedes aegypti]
Unigene55713_All	6.781869951	Up	GJ18479 [Drosophila virilis] >gi|194141032|gb|EDW57458.1| GJ18479 [Drosophila virilis]
CL7026.Contig2_All	6.775735757	Up	PREDICTED: ATP synthase subunit d, mitochondrial-like [Amphimedon queenslandica]
Unigene43698_All	6.770637562	Up	PREDICTED: 3-isopropylmalate dehydrogenase-like [Xenopus (Silurana) tropicalis]
CL9630.Contig1_All	6.770096342	Up	PREDICTED: mannose phosphate isomerase-like [Saccoglossus kowalevskii]
Unigene56988_All	6.756693026	Up	cytochrome P450 family protein [Dictyostelium fasciculatum] >gi|328867195|gb|EGG15578.1| cytochrome P450 family protein [Dictyostelium fasciculatum]
CL2161.Contig1_All	6.756443456	Up	IgA-specific serine endopeptidase, putative [Acanthamoeba castellanii str. Neff] >gi|440796236|gb|ELR17345.1| IgA-specific serine endopeptidase, putative [Acanthamoeba castellanii str. Neff]
Unigene43124_All	6.741835812	Up	hypothetical protein DICPUDRAFT_48962 [Dictyostelium purpureum] >gi|325079768|gb|EGC33352.1| hypothetical protein DICPUDRAFT_48962 [Dictyostelium purpureum]
CL520.Contig2_All	6.735593252	Up	Aldehyde dehydrogenase, mitochondrial [Crassostrea gigas]
Unigene56938_All	6.735289812	Up	MGC81784 protein, related [Neospora caninum Liverpool] >gi|325118289|emb|CBZ53840.1| MGC81784 protein, related [Neospora caninum Liverpool]

CL7794.Contig1_All	6.728449866	Up	seed maturation protein PM34, putative [Acanthamoeba castellanii str. Neff] >gi|440804090|gb|ELR24970.1| seed maturation protein PM34, putative [Acanthamoeba castellanii str. Neff]
CL3178.Contig1_All	6.72368104	Up	hypothetical protein BRAFLDRAFT_115230 [Branchiostoma floridae] >gi|229280343|gb|EEN51112.1| hypothetical protein BRAFLDRAFT_115230 [Branchiostoma floridae]
CL7813.Contig1_All	6.721067824	Up	hypothetical protein [Monosiga brevicollis MX1] >gi|163778141|gb|EDQ91756.1| predicted protein [Monosiga brevicollis MX1]
CL8555.Contig1_All	6.707765125	Up	oligomycin sensitivity-conferring protein [Tribolium castaneum]
CL6388.Contig2_All	6.705719843	Up	PREDICTED: aspartate aminotransferase, cytoplasmic-like, partial [Mus musculus]
Unigene53721_All	6.702228553	Up	hypothetical protein PTSG_03958 [Salpingoeca sp. ATCC 50818]
Unigene47273_All	6.694796427	Up	PREDICTED: o-acetylhomoserine (thiol)-lyase-like [Xenopus (Silurana) tropicalis]
Unigene59730_All	6.693661186	Up	Chain E, Orientation Of Rna Polymerase Ii Within The Human Vp16-Mediator-Pol Ii-Tfiif Assembly
CL1167.Contig2_All	6.690762948	Up	PREDICTED: 1,4-alpha-glucan-branching enzyme [Canis lupus familiaris]
CL8440.Contig1_All	6.686743096	Up	PREDICTED: transaldolase-like [Strongylocentrotus purpuratus]
Unigene57594_All	6.682754736	Up	PREDICTED: probable delta-1-pyrroline-5-carboxylate synthase-like [Metaseiulus occidentalis]
Unigene42902_All	6.675567488	Up	pyruvate kinase [Salpingoeca sp. ATCC 50818]
CL8440.Contig3_All	6.663642305	Up	PREDICTED: transaldolase-like [Strongylocentrotus purpuratus]
CL143.Contig1_All	6.659188029	Up	PREDICTED: succinyl-CoA ligase [GDP-forming] subunit alpha, mitochondrial-like isoform 1 [Bombus terrestris]
CL9004.Contig1_All	6.655382679	Up	PREDICTED: predicted protein-like [Saccoglossus kowalevskii]
Unigene47512_All	6.654596251	Up	cytosolic phospholipase A2 [Gallus gallus] >gi|1352706|sp|P49147.1|PA24A_CHICK RecName: Full=Cytosolic phospholipase A2; Short=cPLA2; AltName: Full=Phospholipase A2 group IVA; Includes: RecName: Full=Phospholipase A2; AltName: Full=Phosphatidylcholine 2-acylhydrolase; Includes: RecName: Full=Lysophospholipase >gi|508625|gb|AAA53228.1| cytosolic phospholipase A2 [Gallus gallus]
Unigene42583_All	6.64244662	Up	CRE-DUT-1 protein [Caenorhabditis remanei] >gi|308246560|gb|EFO90512.1| CRE-DUT-1 protein [Caenorhabditis remanei]
Unigene58976_All	6.641628089	Up	glycerol kinase, putative [Pediculus humanus corporis] >gi|212515272|gb|EEB17443.1| glycerol kinase,

			putative [Pediculus humanus corporis]
CL1847.Contig1_All	6.628986976	Up	hypothetical protein DICPUDRAFT_48737 [Dictyostelium purpureum] >gi|325080264|gb|EGC33827.1| hypothetical protein DICPUDRAFT_48737 [Dictyostelium purpureum]
CL2488.Contig2_All	6.626592905	Up	neuron-specific enolase [Capsaspora owczarzaki ATCC 30864] >gi|320170655|gb|EFW47554.1| neuron-specific enolase [Capsaspora owczarzaki ATCC 30864]
CL1759.Contig1_All	6.624432707	Up	isocitrate dehydrogenase [Mytilus trossulus]
CL641.Contig1_All	6.623619164	Up	NADH-ubiquinone oxidoreductase 75 kDa subunit [Salpingoeca sp. ATCC 50818]
Unigene53515_All	6.62301129	Up	hypothetical protein LOAG_10438 [Loa loa] >gi|307758825|gb|EFO18059.1| hypothetical protein LOAG_10438 [Loa loa]
CL9363.Contig2_All	6.606753185	Up	glyceraldehyde-3-phosphate dehydrogenase [Danaus plexippus]
Unigene55099_All	6.604314627	Up	hypothetical protein CAOG_07884 [Capsaspora owczarzaki ATCC 30864] >gi|320165853|gb|EFW42752.1| hypothetical protein CAOG_07884 [Capsaspora owczarzaki ATCC 30864]
CL7232.Contig1_All	6.602169916	Up	Osmotic growth protein, putative [Perkinsus marinus ATCC 50983] >gi|239878344|gb|EER05108.1| Osmotic growth protein, putative [Perkinsus marinus ATCC 50983]
CL9839.Contig2_All	6.601399391	Up	unknown [Dendroctonus ponderosae]
CL430.Contig3_All	6.597131664	Up	PREDICTED: NADH dehydrogenase [ubiquinone] flavoprotein 2, mitochondrial-like isoform 2 [Oreochromis niloticus]
Unigene41952_All	6.575560178	Up	hypothetical protein AND_17546 [Anopheles darlingi]
CL6132.Contig1_All	6.574959078	Up	AGAP009609-PA [Anopheles gambiae str. PEST] >gi|157013894|gb|EAA13786.5| AGAP009609-PA [Anopheles gambiae str. PEST]
CL6023.Contig2_All	6.569264582	Up	PREDICTED: probable gluconokinase-like isoform 1 [Macaca mulatta] >gi|355567860|gb|EHH24201.1| hypothetical protein EGK_07818 [Macaca mulatta]
Unigene46356_All	6.552542082	Up	hypothetical protein TRIADDRAFT_63196 [Trichoplax adhaerens] >gi|190589004|gb|EDV29026.1| hypothetical protein TRIADDRAFT_63196 [Trichoplax adhaerens]
CL2359.Contig2_All	6.550596286	Up	NAD+ dependent glutamate dehydrogenase [Dictyostelium fasciculatum] >gi|328871248|gb|EGG19619.1| NAD+ dependent glutamate dehydrogenase [Dictyostelium fasciculatum]
Unigene42633_All	6.548112095	Up	cytochrome oxidase subunit 3, partial (mitochondrion) [Bibio xanthopus]

Unigene41687_All	6.540202568	Up	PREDICTED: inosine-5'-monophosphate dehydrogenase 1 [Sarcophilus harrisii]
CL2711.Contig1_All	6.523615445	Up	oxidase heme a,cytochrome [Salpingoeca sp. ATCC 50818]
CL2414.Contig3_All	6.516183945	Up	peroxisomal 3-ketoacyl-CoA thiolase B [Capsaspora owczarzaki ATCC 30864] >gi|320167663|gb|EFW44562.1| peroxisomal 3-ketoacyl-CoA thiolase B [Capsaspora owczarzaki ATCC 30864]
CL5624.Contig1_All	6.505190644	Up	hypothetical protein TRIADDRAFT_51268 [Trichoplax adhaerens] >gi|190589188|gb|EDV29210.1| hypothetical protein TRIADDRAFT_51268 [Trichoplax adhaerens]
Unigene40512_All	6.504090671	Up	isocitrate dehydrogenase [Polysphondylium pallidum PN500]
CL3085.Contig2_All	6.503444148	Up	unnamed protein product [Tetraodon nigroviridis]
Unigene42462_All	6.497554799	Up	ubiquinol cytochrome c reductase subunit QCR8 [Argas monolakensis]
Unigene57441_All	6.490593651	Up	PREDICTED: NADH dehydrogenase [ubiquinone] iron-sulfur protein 2, mitochondrial-like, partial [Hydra magnipapillata]
Unigene40602_All	6.489722276	Up	PREDICTED: triosephosphate isomerase [Sarcophilus harrisii]
CL1567.Contig2_All	6.488431143	Up	isocitrate lyase [Capsaspora owczarzaki ATCC 30864] >gi|320165109|gb|EFW42008.1| isocitrate lyase [Capsaspora owczarzaki ATCC 30864]
Unigene54916_All	6.481883717	Up	conserved domain protein [Trichinella spiralis] >gi|316975086|gb|EFV58545.1| conserved domain protein [Trichinella spiralis]
CL4181.Contig2_All	6.476290689	Up	hypothetical protein TRIADDRAFT_27878 [Trichoplax adhaerens] >gi|190582943|gb|EDV23014.1| hypothetical protein TRIADDRAFT_27878 [Trichoplax adhaerens]
Unigene6932_All	6.474244979	Up	hypothetical protein DFA_11663 [Dictyostelium fasciculatum] >gi|328865516|gb|EGG13902.1| hypothetical protein DFA_11663 [Dictyostelium fasciculatum]
Unigene54292_All	6.473852264	Up	GH20939 [Drosophila grimshawi] >gi|193901697|gb|EDW00564.1| GH20939 [Drosophila grimshawi]
CL6812.Contig2_All	6.462288253	Up	PREDICTED: peroxisomal multifunctional enzyme type 2-like isoform 1 [Ciona intestinalis]
Unigene40824_All	6.461404927	Up	predicted protein [Naegleria gruberi] >gi|284097080|gb|EFC50707.1| predicted protein [Naegleria gruberi]
Unigene58600_All	6.461164224	Up	hypothetical protein CAPTEDRAFT_98567 [Capitella teleta]
CL7195.Contig2_All	6.446761244	Up	PREDICTED: 3-isopropylmalate dehydratase-like [Amphimedon queenslandica]
CL1809.Contig2_All	6.445553953	Up	2-oxoglutarate dehydrogenase [Capsaspora owczarzaki ATCC 30864] >gi|320166651|gb|EFW43550.1| 2-oxoglutarate dehydrogenase [Capsaspora owczarzaki ATCC 30864]

CL9812.Contig1_All	6.444710422	Up	Glutaryl-CoA dehydrogenase, mitochondrial [Crassostrea gigas]
CL7462.Contig1_All	6.444362145	Up	C. briggsae CBR-MEL-32 protein [Caenorhabditis briggsae]
Unigene57119_All	6.441794706	Up	PREDICTED: coproporphyrinogen-III oxidase, mitochondrial [Ovis aries]
Unigene40985_All	6.437818464	Up	NADH dehydrogenase [ubiquinone] iron-sulfur protein 7, mitochondrial [Camponotus floridanus]
CL8757.Contig1_All	6.436936756	Up	NADH:ubiquinone oxidoreductase, putative [Ixodes scapularis] >gi|215492875|gb|EEC02516.1| NADH:ubiquinone oxidoreductase, putative [Ixodes scapularis]
Unigene41013_All	6.429625288	Up	Isocitrate dehydrogenase, mitochondrial [Lepeophtheirus salmonis]
Unigene57494_All	6.414542201	Up	hypothetical protein [Monosiga brevicollis MX1] >gi|163770943|gb|EDQ84618.1| predicted protein [Monosiga brevicollis MX1]
Unigene56966_All	6.403888944	Up	C2 domain containing protein [Trichomonas vaginalis G3] >gi|121891685|gb|EAX97007.1| C2 domain containing protein [Trichomonas vaginalis G3]
CL5772.Contig2_All	6.39800309	Up	pro-phenoloxidase 1 [Locusta migratoria]
Unigene42437_All	6.397728844	Up	Lathosterol oxidase [Crassostrea gigas]
CL6148.Contig1_All	6.39706213	Up	Chain B, Orientation Of Rna Polymerase Ii Within The Human Vp16-Mediator-Pol Ii-Tfiif Assembly
Unigene57367_All	6.387600462	Up	Peroxisomalcoenzyme A synthetase [Acanthamoeba castellanii str. Neff] >gi|440792564|gb|ELR13773.1| Peroxisomalcoenzyme A synthetase [Acanthamoeba castellanii str. Neff]
Unigene40633_All	6.385008535	Up	sphingosine hydroxylase [Acanthamoeba castellanii str. Neff] >gi|440790695|gb|ELR11975.1| sphingosine hydroxylase [Acanthamoeba castellanii str. Neff]
CL7794.Contig2_All	6.383589443	Up	seed maturation protein PM34, putative [Acanthamoeba castellanii str. Neff] >gi|440804090|gb|ELR24970.1| seed maturation protein PM34, putative [Acanthamoeba castellanii str. Neff]
CL2729.Contig1_All	6.382945437	Up	predicted protein [Nematostella vectensis] >gi|156213633|gb|EDO34645.1| predicted protein [Nematostella vectensis]
CL3178.Contig2_All	6.382501982	Up	hypothetical protein TRIADDRAFT_54073 [Trichoplax adhaerens] >gi|190586747|gb|EDV26800.1| hypothetical protein TRIADDRAFT_54073 [Trichoplax adhaerens]
Unigene58592_All	6.381748933	Up	hypothetical protein TcasGA2_TC000207 [Tribolium castaneum]
Unigene56253_All	6.375850122	Up	CDP-alcohol phosphatidyltransferase [Polysphondylium pallidum PN500]
CL9474.Contig1_All	6.375792449	Up	adenylsulfate kinase [Acanthamoeba castellanii str. Neff] >gi|440802140|gb|ELR23079.1| adenylsulfate

			kinase [Acanthamoeba castellanii str. Neff]
Unigene42237_All	6.351137165	Up	spermidine synthase [Dictyostelium fasciculatum] >gi|328867727|gb|EGG16109.1| spermidine synthase [Dictyostelium fasciculatum]
Unigene41212_All	6.34973933	Up	hypothetical protein DICPUDRAFT_44750 [Dictyostelium purpureum] >gi|325086786|gb|EGC40171.1| hypothetical protein DICPUDRAFT_44750 [Dictyostelium purpureum]
CL6386.Contig1_All	6.347984278	Up	hypothetical protein TRIADDRAFT_22398 [Trichoplax adhaerens] >gi|190587195|gb|EDV27248.1| hypothetical protein TRIADDRAFT_22398 [Trichoplax adhaerens]
Unigene6452_All	6.346600725	Up	PREDICTED: similar to steroid dehydrogenase [Tribolium castaneum] >gi|270014405|gb|EFA10853.1| hypothetical protein TcasGA2_TC001630 [Tribolium castaneum]
Unigene41572_All	6.334283245	Up	PREDICTED: NADH dehydrogenase [ubiquinone] iron-sulfur protein 2, mitochondrial-like, partial [Hydra magnipapillata]
CL4567.Contig2_All	6.334076066	Up	chitin synthase, putative [Perkinsus marinus ATCC 50983] >gi|239868941|gb|EER00096.1| chitin synthase, putative [Perkinsus marinus ATCC 50983]
CL623.Contig2_All	6.333023772	Up	Chain I, Orientation Of Rna Polymerase Ii Within The Human Vp16-Mediator-Pol Ii-Tfiif Assembly
CL5978.Contig1_All	6.327492352	Up	5-methyltetrahydropteroyltriglutamate-homocysteine-S-methyltransferase [Polysphondylium pallidum PN500]
CL1567.Contig1_All	6.318282366	Up	isocitrate lyase [Capsaspora owczarzaki ATCC 30864] >gi|320165109|gb|EFW42008.1| isocitrate lyase [Capsaspora owczarzaki ATCC 30864]
Unigene55177_All	6.318116899	Up	PREDICTED: hydroxysteroid dehydrogenase-like protein 2-like [Strongylocentrotus purpuratus]
Unigene42435_All	6.316964767	Up	enolase [Echinococcus granulosus]
Unigene43666_All	6.306343781	Up	sigma-class glutathione S-transferase [Laternula elliptica]
CL8835.Contig1_All	6.300697776	Up	PREDICTED: NADH dehydrogenase [ubiquinone] iron-sulfur protein 8, mitochondrial-like [Metaseiulus occidentalis]
CL8419.Contig2_All	6.300575417	Up	PREDICTED: fumarylacetoacetase-like [Anolis carolinensis]
CL2979.Contig2_All	6.300258503	Up	glutamine synthetase [Paracentrotus lividus]
CL4793.Contig1_All	6.297065023	Up	malate dehydrogenase [Capsaspora owczarzaki ATCC 30864] >gi|320165329|gb|EFW42228.1| malate dehydrogenase [Capsaspora owczarzaki ATCC 30864]
Unigene58421_All	6.285551977	Up	aldehyde dehydrogenase [Steinernema feltiae]

<table>
<tr><td>CL548.Contig3_All</td><td>6.285026715</td><td>Up</td><td>hypothetical protein [Monosiga brevicollis MX1] >gi|163775779|gb|EDQ89402.1| predicted protein [Monosiga brevicollis MX1]</td></tr>
<tr><td>Unigene60754_All</td><td>6.284484007</td><td>Up</td><td>cystathionine gamma-lyase [Dictyostelium purpureum] >gi|325086027|gb|EGC39424.1| cystathionine gamma-lyase [Dictyostelium purpureum]</td></tr>
<tr><td>CL5366.Contig1_All</td><td>6.284019431</td><td>Up</td><td>predicted protein [Nematostella vectensis] >gi|156228565|gb|EDO49363.1| predicted protein [Nematostella vectensis]</td></tr>
<tr><td>CL513.Contig1_All</td><td>6.267513339</td><td>Up</td><td>Isocitrate dehydrogenase [NADP], mitochondrial [Crassostrea gigas]</td></tr>
<tr><td>CL2829.Contig2_All</td><td>6.266596234</td><td>Up</td><td>PREDICTED: ATP synthase lipid-binding protein, mitochondrial-like [Strongylocentrotus purpuratus]</td></tr>
<tr><td>CL8602.Contig1_All</td><td>6.264833522</td><td>Up</td><td>unnamed protein product [Tetraodon nigroviridis]</td></tr>
<tr><td>CL2784.Contig2_All</td><td>6.250116179</td><td>Up</td><td>Choline/ethanolamine kinase [Harpegnathos saltator]</td></tr>
<tr><td>CL520.Contig1_All</td><td>6.243942359</td><td>Up</td><td>Aldehyde dehydrogenase, mitochondrial [Crassostrea gigas]</td></tr>
<tr><td>Unigene58769_All</td><td>6.233033746</td><td>Up</td><td>prolyl-tRNA synthetase, putative [Pediculus humanus corporis] >gi|212510156|gb|EEB13371.1| prolyl-tRNA synthetase, putative [Pediculus humanus corporis]</td></tr>
<tr><td>Unigene41375_All</td><td>6.232956546</td><td>Up</td><td>Atp6v1a-prov protein [Salpingoeca sp. ATCC 50818]</td></tr>
<tr><td>CL561.Contig1_All</td><td>6.231073115</td><td>Up</td><td>malate synthase [Capsaspora owczarzaki ATCC 30864] >gi|320165110|gb|EFW42009.1| malate synthase [Capsaspora owczarzaki ATCC 30864]</td></tr>
<tr><td>CL5125.Contig1_All</td><td>6.227580933</td><td>Up</td><td>pyruvate carboxylase [Capsaspora owczarzaki ATCC 30864] >gi|320168344|gb|EFW45243.1| pyruvate carboxylase [Capsaspora owczarzaki ATCC 30864]</td></tr>
<tr><td>CL3296.Contig2_All</td><td>6.226444023</td><td>Up</td><td>phosphoglycerete kinase 1 [Capsaspora owczarzaki ATCC 30864] >gi|320168420|gb|EFW45319.1| phosphoglycerete kinase 1 [Capsaspora owczarzaki ATCC 30864]</td></tr>
<tr><td>Unigene62207_All</td><td>6.213649702</td><td>Up</td><td>unnamed protein product [Trypanosoma congolense IL3000]</td></tr>
<tr><td>Unigene55115_All</td><td>6.213409875</td><td>Up</td><td>ACYPI008879 [Acyrthosiphon pisum]</td></tr>
<tr><td>CL5912.Contig1_All</td><td>6.202250511</td><td>Up</td><td>4-hydroxyphenylpyruvate dioxygenase [Oxytricha trifallax]</td></tr>
<tr><td>CL9967.Contig1_All</td><td>6.197937335</td><td>Up</td><td>1,4-alpha-glucan branching enzyme [Polysphondylium pallidum PN500]</td></tr>
<tr><td>Unigene42213_All</td><td>6.196522116</td><td>Up</td><td>inositol-3-phosphate synthase [Dictyostelium purpureum] >gi|325078585|gb|EGC32229.1| inositol-3-phosphate synthase [Dictyostelium purpureum]</td></tr>
<tr><td>CL6846.Contig1_All</td><td>6.19603207</td><td>Up</td><td>PREDICTED: fumarylacetoacetase-like [Hydra magnipapillata]</td></tr>
<tr><td>Unigene50251_All</td><td>6.195697555</td><td>Up</td><td>adenylosuccinate synthetase [Polysphondylium pallidum PN500]</td></tr>
</table>

CL3396.Contig1_All	6.188300763	Up	C2 domain containing protein [Oxytricha trifallax]
Unigene57022_All	6.18486768	Up	hypothetical protein [Monosiga brevicollis MX1] >gi|163770700|gb|EDQ84382.1| predicted protein [Monosiga brevicollis MX1]
CL392.Contig2_All	6.183543044	Up	PREDICTED: similar to Glutamine:fructose-6-phosphate aminotransferase 1 CG12449-PH [Tribolium castaneum] >gi|270003358|gb|EEZ99805.1| hypothetical protein TcasGA2_TC002585 [Tribolium castaneum]
CL5834.Contig1_All	6.179666396	Up	vacuolar ATP synthase subunit, putative [Ichthyophthirius multifiliis] >gi|340502176|gb|EGR28889.1| vacuolar ATP synthase subunit, putative [Ichthyophthirius multifiliis]
Unigene61801_All	6.178079351	Up	4-aminobutyrate transaminase [Dictyostelium discoideum AX4] >gi|74897469|sp|Q55FI1.1|GABT_DICDI RecName: Full=4-aminobutyrate aminotransferase; AltName: Full=GABA aminotransferase; Short=GABA-AT; AltName: Full=Gamma-amino-N-butyrate transaminase; Short=GABA transaminase >gi|60475570|gb|EAL73505.1| 4-aminobutyrate transaminase [Dictyostelium discoideum AX4]
Unigene56534_All	6.176720563	Up	predicted protein [Naegleria gruberi] >gi|284097080|gb|EFC50707.1| predicted protein [Naegleria gruberi]
Unigene42909_All	6.169333806	Up	ATP synthase gamma subunit, putative [Ixodes scapularis] >gi|215509716|gb|EEC19169.1| ATP synthase gamma subunit, putative [Ixodes scapularis]
Unigene43312_All	6.164140303	Up	NADH-Ubiquinone oxidoreductase B14 subunit [Pediculus humanus corporis] >gi|212511485|gb|EEB14460.1| NADH-Ubiquinone oxidoreductase B14 subunit [Pediculus humanus corporis]
Unigene60308_All	6.161093701	Up	2-amino-3-carboxymuconate-6-semialdehyde decarboxylase [Salpingoeca sp. ATCC 50818]
CL7627.Contig1_All	6.159538383	Up	aldehyde dehydrogenase [Capsaspora owczarzaki ATCC 30864] >gi|320166834|gb|EFW43733.1| aldehyde dehydrogenase [Capsaspora owczarzaki ATCC 30864]
CL9431.Contig1_All	6.157178485	Up	PREDICTED: adenosylhomocysteinase-like [Bombus impatiens]
CL1442.Contig1_All	6.155979516	Up	PREDICTED: v-type proton ATPase 16 kDa proteolipid subunit-like [Amphimedon queenslandica]
CL5589.Contig2_All	6.153250814	Up	hypothetical protein [Monosiga brevicollis MX1] >gi|163772346|gb|EDQ85999.1| predicted protein [Monosiga brevicollis MX1]
CL7157.Contig1_All	6.149996201	Up	RING zinc finger-containing protein [Dictyostelium fasciculatum] >gi|328875329|gb|EGG23694.1| RING zinc finger-containing protein [Dictyostelium fasciculatum]

CL8608.Contig1_All	6.142617655	Up	sulfate adenylyltransferase [Acanthamoeba castellanii str. Neff] >gi|440795708|gb|ELR16825.1| sulfate adenylyltransferase [Acanthamoeba castellanii str. Neff]
Unigene41466_All	6.136182886	Up	PREDICTED: uncharacterized protein LOC100882671 [Megachile rotundata]
CL8030.Contig1_All	6.135994235	Up	Succinate dehydrogenase cytochrome b560 subunit, mitochondrial precursor [Caligus clemensi]
CL1066.Contig1_All	6.131305419	Up	predicted protein [Nematostella vectensis] >gi|156221628|gb|EDO42481.1| predicted protein [Nematostella vectensis]
Unigene57065_All	6.126174064	Up	Dihydrolipoamide succinyltransferase [Oxytricha trifallax]
CL84.Contig1_All	6.115997889	Up	PREDICTED: similar to AGAP011050-PA [Tribolium castaneum] >gi|270002564|gb|EEZ99011.1| hypothetical protein TcasGA2_TC004879 [Tribolium castaneum]
Unigene47427_All	6.112317546	Up	PREDICTED: putative glutamate synthase [NADPH]-like [Apis florea]
CL385.Contig2_All	6.1109947	Up	1,2-dihydroxy-3-keto-5-methylthiopentene dioxygenase 3 [Salpingoeca sp. ATCC 50818]
CL3738.Contig1_All	6.106983167	Up	ATP synthase, H+ transporting, mitochondrial F1 complex, alpha subunit 1, cardiac muscle [Homo sapiens]
Unigene49261_All	6.103746949	Up	PREDICTED: cytochrome P450 2U1-like [Ornithorhynchus anatinus]
CL2490.Contig2_All	6.100737419	Up	PREDICTED: cytochrome b-c1 complex subunit 6, mitochondrial-like [Strongylocentrotus purpuratus]
Unigene56953_All	6.097208531	Up	glutamate decarboxylase [Acanthamoeba castellanii str. Neff] >gi|440801136|gb|ELR22160.1| glutamate decarboxylase [Acanthamoeba castellanii str. Neff]
Unigene41257_All	6.085667328	Up	WSC domain-containing protein [Dictyostelium fasciculatum] >gi|328871330|gb|EGG19701.1| WSC domain-containing protein [Dictyostelium fasciculatum]
Unigene51871_All	6.085379758	Up	CDP-diacylglycerol synthase [Capsaspora owczarzaki ATCC 30864] >gi|320166752|gb|EFW43651.1| CDP-diacylglycerol synthase [Capsaspora owczarzaki ATCC 30864]
Unigene60159_All	6.084297356	Up	PREDICTED: hypothetical protein LOC100168206 [Acyrthosiphon pisum]
CL6694.Contig2_All	6.069931726	Up	vacuolar ATP synthase proteolipid subunit [Trichinella spiralis] >gi|316979348|gb|EFV62153.1| vacuolar ATP synthase proteolipid subunit [Trichinella spiralis]
CL5589.Contig1_All	6.066228105	Up	hypothetical protein [Monosiga brevicollis MX1] >gi|163772346|gb|EDQ85999.1| predicted protein [Monosiga brevicollis MX1]
Unigene42479_All	6.065784449	Up	Enoyl-CoA hydratase [Oxytricha trifallax]
Unigene48360_All	6.065602187	Up	glycine dehydrogenase [Capsaspora owczarzaki ATCC 30864] >gi|320165212|gb|EFW42111.1| glycine

			dehydrogenase [Capsaspora owczarzaki ATCC 30864]
CL9605.Contig2_All	6.065182997	Up	PREDICTED: ribose-phosphate pyrophosphokinase 1-like [Oreochromis niloticus] >gi|432878820|ref|XP_004073401.1| PREDICTED: ribose-phosphate pyrophosphokinase 1-like isoform 1 [Oryzias latipes]
Unigene41776_All	6.064913226	Up	PREDICTED: 2-dehydro-3-deoxy-D-gluconate 5-dehydrogenase-like, partial [Acyrthosiphon pisum]
CL4009.Contig2_All	6.064785209	Up	hypothetical protein BRAFLDRAFT_98927 [Branchiostoma floridae] >gi|229287278|gb|EEN57986.1| hypothetical protein BRAFLDRAFT_98927 [Branchiostoma floridae]
Unigene47060_All	6.064703145	Up	sphingosine hydroxylase [Acanthamoeba castellanii str. Neff] >gi|440790695|gb|ELR11975.1| sphingosine hydroxylase [Acanthamoeba castellanii str. Neff]
Unigene54746_All	6.061476866	Up	hypothetical protein PPL_08064 [Polysphondylium pallidum PN500]
Unigene40615_All	6.054760277	Up	uncharacterized protein LOC641566 [Danio rerio]
CL8632.Contig2_All	6.054521995	Up	isopropylmalate dehydrogenase [Capsaspora owczarzaki ATCC 30864] >gi|320169661|gb|EFW46560.1| isopropylmalate dehydrogenase [Capsaspora owczarzaki ATCC 30864]
CL7772.Contig1_All	6.045710327	Up	PREDICTED: glutaryl-CoA dehydrogenase, mitochondrial-like [Bombus impatiens]
Unigene42373_All	6.036903456	Up	PREDICTED: cytochrome c1, heme protein, mitochondrial-like [Amphimedon queenslandica]
CL9217.Contig1_All	6.025414826	Up	PREDICTED: dolichol-phosphate mannosyltransferase subunit 3-like [Oreochromis niloticus]
Unigene41703_All	6.024544693	Up	glutamate decarboxylase [Acanthamoeba castellanii str. Neff] >gi|440801136|gb|ELR22160.1| glutamate decarboxylase [Acanthamoeba castellanii str. Neff]
Unigene56979_All	6.021773002	Up	acyl-coa dehydrogenase, putative [Perkinsus marinus ATCC 50983] >gi|239890237|gb|EER12110.1| acyl-coa dehydrogenase, putative [Perkinsus marinus ATCC 50983]
CL6575.Contig2_All	6.01766169	Up	phosphoribosylglycinamide formyltransferase [Capsaspora owczarzaki ATCC 30864] >gi|320167463|gb|EFW44362.1| phosphoribosylglycinamide formyltransferase [Capsaspora owczarzaki ATCC 30864]
Unigene12573_All	6.017077045	Up	AGAP008062-PA [Anopheles gambiae str. PEST] >gi|116123205|gb|EAA12601.3| AGAP008062-PA [Anopheles gambiae str. PEST]
CL9237.Contig1_All	6.010576534	Up	short-chain dehydrogenase/reductase SDR [Capsaspora owczarzaki ATCC 30864] >gi|320162883|gb|EFW39782.1| short-chain dehydrogenase/reductase SDR [Capsaspora owczarzaki ATCC 30864]

CL6023.Contig1_All	5.989884167	Up	shikimate-kinase [Schistosoma mansoni]
Unigene49928_All	5.983996418	Up	pyrroline-5-carboxylate synthetase-like protein [Leishmania infantum JPCM5] >gi|398021164|ref|XP_003863745.1| pyrroline-5-carboxylate synthetase-like protein [Leishmania donovani] >gi|134072374|emb|CAM71082.1| pyrroline-5-carboxylate synthetase-like protein [Leishmania infantum JPCM5] >gi|322501978|emb|CBZ37062.1| pyrroline-5-carboxylate synthetase-like protein [Leishmania donovani]
Unigene47617_All	5.983905262	Up	glutamine synthetase [Acanthamoeba castellanii str. Neff] >gi|440804153|gb|ELR25031.1| glutamine synthetase [Acanthamoeba castellanii str. Neff]
Unigene57031_All	5.976676215	Up	PREDICTED: probable isocitrate dehydrogenase [NAD] subunit alpha, mitochondrial-like [Hydra magnipapillata]
CL748.Contig1_All	5.974055234	Up	Protein MDH-2 [Caenorhabditis elegans] >gi|3183074|sp|O02640.1|MDHM_CAEEL RecName: Full=Probable malate dehydrogenase, mitochondrial; Flags: Precursor >gi|351061902|emb|CCD69776.1| Protein MDH-2 [Caenorhabditis elegans]
Unigene55025_All	5.96299885	Up	PREDICTED: cytochrome c oxidase subunit 5B, mitochondrial-like [Callithrix jacchus]
CL8835.Contig2_All	5.962457137	Up	PREDICTED: NADH dehydrogenase [ubiquinone] iron-sulfur protein 8, mitochondrial-like [Metaseiulus occidentalis]
Unigene50901_All	5.961981732	Up	predicted protein [Nematostella vectensis] >gi|156229239|gb|EDO50035.1| predicted protein [Nematostella vectensis]
Unigene57802_All	5.961864871	Up	hypothetical protein CAPTEDRAFT_191669 [Capitella teleta]
CL2103.Contig2_All	5.958678014	Up	isocitrate dehydrogenase subunit 1 [Capsaspora owczarzaki ATCC 30864] >gi|320169014|gb|EFW45913.1| isocitrate dehydrogenase subunit 1 [Capsaspora owczarzaki ATCC 30864]
Unigene22317_All	5.952982944	Up	pro-phenoloxidase 1 [Locusta migratoria]
Unigene41601_All	5.95130492	Up	hypothetical protein DAPPUDRAFT_230594 [Daphnia pulex]
CL2824.Contig2_All	5.942006028	Up	hypothetical protein DICPUDRAFT_48509 [Dictyostelium purpureum] >gi|325080636|gb|EGC34183.1| hypothetical protein DICPUDRAFT_48509 [Dictyostelium purpureum]
Unigene55163_All	5.940628497	Up	PREDICTED: maleylacetoacetate isomerase-like, partial [Hydra magnipapillata]
CL6855.Contig1_All	5.930385254	Up	--

CL4931.Contig1_All	5.925172036	Up	PREDICTED: cytochrome b-c1 complex subunit 7-like [Hydra magnipapillata]
CL5623.Contig2_All	5.92011512	Up	conserved hypothetical protein [Capsaspora owczarzaki ATCC 30864] >gi|320168370|gb|EFW45269.1| conserved hypothetical protein [Capsaspora owczarzaki ATCC 30864]
CL7943.Contig2_All	5.9092409	Up	PREDICTED: CNDP dipeptidase 2 isoform 2 [Oryctolagus cuniculus]
Unigene13043_All	5.905169717	Up	hypothetical protein TcasGA2_TC012121 [Tribolium castaneum]
Unigene58156_All	5.898567548	Up	hypothetical protein DICPUDRAFT_56740 [Dictyostelium purpureum] >gi|325079375|gb|EGC32978.1| hypothetical protein DICPUDRAFT_56740 [Dictyostelium purpureum]
Unigene56448_All	5.896676285	Up	malate synthase [Capsaspora owczarzaki ATCC 30864] >gi|320165110|gb|EFW42009.1| malate synthase [Capsaspora owczarzaki ATCC 30864]
CL7514.Contig2_All	5.895904287	Up	HAD-superfamily hydrolase [Capsaspora owczarzaki ATCC 30864] >gi|320168056|gb|EFW44955.1| HAD-superfamily hydrolase [Capsaspora owczarzaki ATCC 30864]
Unigene54546_All	5.895900732	Up	GK17336 [Drosophila willistoni] >gi|194158152|gb|EDW73053.1| GK17336 [Drosophila willistoni]
Unigene42424_All	5.880342126	Up	isocitrate dehydrogenase subunit 1 [Capsaspora owczarzaki ATCC 30864] >gi|320169014|gb|EFW45913.1| isocitrate dehydrogenase subunit 1 [Capsaspora owczarzaki ATCC 30864]
CL1214.Contig2_All	5.875822791	Up	hypothetical protein BRAFLDRAFT_126490 [Branchiostoma floridae] >gi|229291503|gb|EEN62174.1| hypothetical protein BRAFLDRAFT_126490 [Branchiostoma floridae]
Unigene47589_All	5.875167721	Up	NADH dehydrogenase iron-sulfur protein 8, mitochondrial precursor [Osmerus mordax]
Unigene59231_All	5.874625458	Up	aspartate transcarbamylase [Capsaspora owczarzaki ATCC 30864] >gi|320170371|gb|EFW47270.1| aspartate transcarbamylase [Capsaspora owczarzaki ATCC 30864]
CL1226.Contig2_All	5.861030405	Up	predicted protein [Nematostella vectensis] >gi|156228174|gb|EDO48974.1| predicted protein [Nematostella vectensis]
Unigene51092_All	5.853781998	Up	prostaglandin F synthase [Penaeus monodon]
Unigene42727_All	5.853441725	Up	PREDICTED: cyclin-K-like [Meleagris gallopavo]
CL3742.Contig1_All	5.85206751	Up	phosphoribosylaminoimidazole-succinocarboxamide synthase [Capsaspora owczarzaki ATCC 30864] >gi|320169740|gb|EFW46639.1| phosphoribosylaminoimidazole-succinocarboxamide synthase [Capsaspora owczarzaki ATCC 30864]
Unigene42179_All	5.851394647	Up	Alpha-enolase [Anoplopoma fimbria]

Unigene56901_All	5.845185688	Up	hypothetical protein PTSG_02255 [Salpingoeca sp. ATCC 50818]
CL4195.Contig2_All	5.835054791	Up	Alcohol dehydrogenase class-3 [Salmo salar] >gi|223672821|gb|ACN12592.1| Alcohol dehydrogenase class-3 [Salmo salar] >gi|224747157|gb|ACN62250.1| alcohol dehydrogenase class 3 [Salmo salar]
Unigene42500_All	5.831651051	Up	PREDICTED: s-adenosylmethionine synthase isoform type-1-like [Amphimedon queenslandica]
Unigene46748_All	5.829652272	Up	PREDICTED: peroxidase-like [Apis florea]
Unigene41035_All	5.829500563	Up	predicted protein [Nematostella vectensis] >gi|156228688|gb|EDO49486.1| predicted protein [Nematostella vectensis]
Unigene59344_All	5.828605839	Up	unnamed protein product, partial [Leishmania mexicana MHOM/GT/2001/U1103] >gi|356491245|emb|CBZ41040.1| unnamed protein product, partial [Leishmania mexicana MHOM/GT/2001/U1103]
Unigene62061_All	5.827900627	Up	Aldehyde dehydrogenase, mitochondrial [Crassostrea gigas]
Unigene49051_All	5.827792839	Up	hypothetical protein TcasGA2_TC000207 [Tribolium castaneum]
CL7330.Contig1_All	5.821419844	Up	hypothetical protein [Monosiga brevicollis MX1] >gi|163776257|gb|EDQ89877.1| predicted protein [Monosiga brevicollis MX1]
CL5155.Contig1_All	5.812972349	Up	acylCoA synthetase, putative [Acanthamoeba castellanii str. Neff] >gi|440794657|gb|ELR15814.1| acylCoA synthetase, putative [Acanthamoeba castellanii str. Neff]
CL1296.Contig2_All	5.811325645	Up	pyruvate dehydrogenase E1 alpha 1 [Capsaspora owczarzaki ATCC 30864] >gi|320167542|gb|EFW44441.1| pyruvate dehydrogenase E1 alpha 1 [Capsaspora owczarzaki ATCC 30864]
CL6650.Contig2_All	5.809892645	Up	catalase [Acanthamoeba castellanii str. Neff] >gi|372290526|gb|AEX91750.1| catalase 2 [Acanthamoeba castellanii] >gi|440792545|gb|ELR13756.1| catalase [Acanthamoeba castellanii str. Neff]
Unigene54382_All	5.807026701	Up	hypothetical protein TRIADDRAFT_33726 [Trichoplax adhaerens] >gi|190579220|gb|EDV19320.1| hypothetical protein TRIADDRAFT_33726, partial [Trichoplax adhaerens]
CL6596.Contig1_All	5.806471103	Up	Hexokinase type 2 [Acromyrmex echinatior]
CL3124.Contig1_All	5.805950203	Up	PREDICTED: mitochondrial malate dehydrogenase-like [Saccoglossus kowalevskii]
CL3518.Contig2_All	5.805906112	Up	PREDICTED: arginase-1-like isoform 1 [Otolemur garnettii]
Unigene56249_All	5.805757494	Up	hypothetical protein TcasGA2_TC012061 [Tribolium castaneum]
CL3525.Contig1_All	5.805597324	Up	NADH dehydrogenase 1 beta subcomplex subunit 9 [Salmo salar]

<table>
<tr><td>CL3683.Contig1_All</td><td>5.805326892</td><td>Up</td><td>glyceraldehyde-3-phosphate dehydrogenase [Cricetulus griseus] >gi|120689|sp|P17244.2|G3P_CRIGR RecName: Full=Glyceraldehyde-3-phosphate dehydrogenase; Short=GAPDH; AltName: Full=Peptidyl-cysteine S-nitrosylase GAPDH</td></tr>
<tr><td>Unigene50579_All</td><td>5.805182444</td><td>Up</td><td>PREDICTED: isocitrate dehydrogenase [NAD] subunit 1, mitochondrial-like [Amphimedon queenslandica]</td></tr>
<tr><td>Unigene56434_All</td><td>5.799574856</td><td>Up</td><td>mitochondrial ATP synthase gamma-subunit [Cyprinus carpio]</td></tr>
<tr><td>CL5139.Contig2_All</td><td>5.778987199</td><td>Up</td><td>Hypothetical protein CBG05011 [Caenorhabditis briggsae]</td></tr>
<tr><td>CL7279.Contig1_All</td><td>5.773103591</td><td>Up</td><td>allantoicase [Polysphondylium pallidum PN500]</td></tr>
<tr><td>CL5346.Contig1_All</td><td>5.76340357</td><td>Up</td><td>glucosamine-6-phosphate deaminase 1 [Salpingoeca sp. ATCC 50818]</td></tr>
<tr><td>Unigene53951_All</td><td>5.758574532</td><td>Up</td><td>PREDICTED: UDP-glucuronosyltransferase 2B10-like [Acyrthosiphon pisum]</td></tr>
<tr><td>CL5896.Contig1_All</td><td>5.758011911</td><td>Up</td><td>isocitrate lyase [Capsaspora owczarzaki ATCC 30864] >gi|320165109|gb|EFW42008.1| isocitrate lyase [Capsaspora owczarzaki ATCC 30864]</td></tr>
<tr><td>CL6396.Contig1_All</td><td>5.756367832</td><td>Up</td><td>unknown [Dendroctonus ponderosae]</td></tr>
<tr><td>CL778.Contig2_All</td><td>5.733478887</td><td>Up</td><td>hypothetical protein BRAFLDRAFT_86500 [Branchiostoma floridae] >gi|229294759|gb|EEN65413.1| hypothetical protein BRAFLDRAFT_86500 [Branchiostoma floridae]</td></tr>
<tr><td>CL3104.Contig2_All</td><td>5.732705865</td><td>Up</td><td>GG13904 [Drosophila erecta] >gi|190654193|gb|EDV51436.1| GG13904 [Drosophila erecta]</td></tr>
<tr><td>Unigene43088_All</td><td>5.732153235</td><td>Up</td><td>inositol-3-phosphate synthase A [Capsaspora owczarzaki ATCC 30864] >gi|320166496|gb|EFW43395.1| inositol-3-phosphate synthase A [Capsaspora owczarzaki ATCC 30864]</td></tr>
<tr><td>CL6368.Contig1_All</td><td>5.720836187</td><td>Up</td><td>glucose-6-phosphate 1-dehydrogenase [Cephaloscyllium umbratile]</td></tr>
<tr><td>CL4805.Contig2_All</td><td>5.718554401</td><td>Up</td><td>hypothetical protein TRIADDRAFT_58627 [Trichoplax adhaerens] >gi|190582851|gb|EDV22923.1| hypothetical protein TRIADDRAFT_58627 [Trichoplax adhaerens]</td></tr>
<tr><td>Unigene65489_All</td><td>5.708792529</td><td>Up</td><td>predicted protein [Naegleria gruberi] >gi|284097080|gb|EFC50707.1| predicted protein [Naegleria gruberi]</td></tr>
<tr><td>Unigene42263_All</td><td>5.708330317</td><td>Up</td><td>vacuolar ATPase subunit DVA41 [Dictyostelium discoideum AX4] >gi|66822901|ref|XP_644805.1| vacuolar ATPase subunit DVA41 [Dictyostelium discoideum AX4] >gi|166214669|sp|P54641.2|VA0D_DICDI RecName: Full=V-type proton ATPase subunit d; Short=V-ATPase subunit d; AltName: Full=DVA41; AltName: Full=V-ATPase 41 kDa accessory protein; AltName: Full=Vacuolar proton pump subunit d >gi|60472568|gb|EAL70519.1| vacuolar ATPase subunit DVA41 [Dictyostelium discoideum AX4] >gi|60472804|gb|EAL70753.1| vacuolar ATPase subunit</td></tr>
</table>

			DVA41 [Dictyostelium discoideum AX4]
Unigene41941_All	5.70733589	Up	GMP synthase, putative [Toxoplasma gondii GT1]
CL46.Contig1_All	5.707075676	Up	putative delta-1-pyrroline-5-carboxylate dehydrogenase [Polysphondylium pallidum PN500]
Unigene41138_All	5.701509058	Up	hypothetical protein [Monosiga brevicollis MX1] >gi|163771649|gb|EDQ85313.1| predicted protein [Monosiga brevicollis MX1]
CL385.Contig1_All	5.682762485	Up	1,2-dihydroxy-3-keto-5-methylthiopentene dioxygenase 3 [Salpingoeca sp. ATCC 50818]
Unigene47460_All	5.68156321	Up	hypothetical protein CRE_19553 [Caenorhabditis remanei] >gi|308260741|gb|EFP04694.1| hypothetical protein CRE_19553 [Caenorhabditis remanei]
CL803.Contig1_All	5.662056598	Up	Argininosuccinate synthase [Callorhinchus milii]
Unigene57036_All	5.66071227	Up	PREDICTED: probable NADP-dependent mannitol dehydrogenase-like, partial [Acyrthosiphon pisum]
Unigene52281_All	5.657282136	Up	vacuolar H+ ATPase B subunit [Dictyostelium fasciculatum] >gi|328871769|gb|EGG20139.1| vacuolar H+ ATPase B subunit [Dictyostelium fasciculatum]
Unigene59016_All	5.657238938	Up	PREDICTED: peroxisomal multifunctional enzyme type 2 [Oreochromis niloticus]
Unigene48832_All	5.657086639	Up	PREDICTED: 3-isopropylmalate dehydrogenase-like [Xenopus (Silurana) tropicalis]
Unigene61624_All	5.656779661	Up	Aldehyde dehydrogenase family 3 member B1 [Crassostrea gigas]
Unigene57701_All	5.656214527	Up	hypothetical protein TRIADDRAFT_20946 [Trichoplax adhaerens] >gi|190588269|gb|EDV28311.1| hypothetical protein TRIADDRAFT_20946 [Trichoplax adhaerens]
Unigene57251_All	5.656051988	Up	hypothetical protein DICPUDRAFT_56740 [Dictyostelium purpureum] >gi|325079375|gb|EGC32978.1| hypothetical protein DICPUDRAFT_56740 [Dictyostelium purpureum]
Unigene55609_All	5.640585637	Up	expressed hypothetical protein [Trichoplax adhaerens] >gi|190586853|gb|EDV26906.1| expressed hypothetical protein [Trichoplax adhaerens]
Unigene60243_All	5.631095593	Up	enolase 3 [Salpingoeca sp. ATCC 50818]
Unigene51976_All	5.630486553	Up	RecName: Full=Hydroxymethylglutaryl-CoA synthase 1; Short=HMG-CoA synthase 1; AltName: Full=3-hydroxy-3-methylglutaryl coenzyme A synthase 1 >gi|1772495|emb|CAA52032.1| hydroxymethylglutaryl-CoA synthase [Blattella germanica]
Unigene61768_All	5.630287769	Up	PREDICTED: glycerol kinase-like isoform 2 [Anolis carolinensis]
Unigene62929_All	5.629599478	Up	hypothetical protein BRAFLDRAFT_211091 [Branchiostoma floridae] >gi|229289567|gb|EEN60253.1| hypothetical protein BRAFLDRAFT_211091 [Branchiostoma floridae]

Unigene58426_All	5.629296318	Up	oxoglutarate dehydrogenase (succinyltransferring), E1 component, putative [Acanthamoeba castellanii str. Neff] >gi|440798433|gb|ELR19501.1| oxoglutarate dehydrogenase (succinyltransferring), E1 component, putative [Acanthamoeba castellanii str. Neff]
Unigene42612_All	5.624130811	Up	3-phosphoglycerate dehydrogenase [Dictyostelium fasciculatum] >gi|328867831|gb|EGG16212.1| 3-phosphoglycerate dehydrogenase [Dictyostelium fasciculatum]
CL7847.Contig1_All	5.620947001	Up	PREDICTED: 3-ketoacyl-CoA thiolase B, peroxisomal-like [Taeniopygia guttata]
CL4013.Contig1_All	5.615088344	Up	GK16804 [Drosophila willistoni] >gi|194158242|gb|EDW73143.1| GK16804 [Drosophila willistoni]
CL3796.Contig1_All	5.609044503	Up	hypothetical protein TcasGA2_TC005493 [Tribolium castaneum]
Unigene48409_All	5.603669469	Up	aconitate hydratase, mitochondrial, putative [Acanthamoeba castellanii str. Neff] >gi|440799601|gb|ELR20645.1| aconitate hydratase, mitochondrial, putative [Acanthamoeba castellanii str. Neff]
Unigene59004_All	5.602930077	Up	hypothetical protein DICPUDRAFT_97663 [Dictyostelium purpureum] >gi|325082685|gb|EGC36159.1| hypothetical protein DICPUDRAFT_97663 [Dictyostelium purpureum]
Unigene61024_All	5.601438254	Up	PREDICTED: CAD protein-like [Oryzias latipes]
Unigene56180_All	5.599058398	Up	GG15230 [Drosophila erecta] >gi|190653684|gb|EDV50927.1| GG15230 [Drosophila erecta]
Unigene46355_All	5.593966611	Up	PREDICTED: aspartate aminotransferase, mitochondrial isoform 2 [Ovis aries]
Unigene58427_All	5.576202833	Up	Glycerol kinase 2 [Dictyostelium fasciculatum] >gi|328871818|gb|EGG20188.1| Glycerol kinase 2 [Dictyostelium fasciculatum]
Unigene50448_All	5.573568219	Up	alanine transaminase [Dictyostelium purpureum] >gi|325078081|gb|EGC31753.1| alanine transaminase [Dictyostelium purpureum]
Unigene58807_All	5.547088534	Up	enolase 3 [Salpingoeca sp. ATCC 50818]
CL7847.Contig3_All	5.536503574	Up	Acetyl-CoA acyltransferases family protein [Oxytricha trifallax]
CL9164.Contig1_All	5.531002757	Up	Peroxisomal multifunctional enzyme type 2 [Crassostrea gigas]
CL4218.Contig1_All	5.528636573	Up	PREDICTED: RNA-directed DNA polymerase from mobile element jockey-like [Strongylocentrotus purpuratus]
Unigene65309_All	5.518654921	Up	hypothetical protein TcasGA2_TC012288 [Tribolium castaneum]
Unigene48068_All	5.518473776	Up	S-adenosylmethionine synthetase, putative [Toxoplasma gondii ME49] >gi|211964352|gb|EEA99547.1| S-adenosylmethionine synthetase, putative [Toxoplasma gondii ME49] >gi|221486026|gb|EEE24296.1|

			S-adenosylmethionine synthetase, putative [Toxoplasma gondii GT1] >gi|221503522|gb|EEE29213.1| S-adenosylmethionine synthetase, putative [Toxoplasma gondii VEG]
Unigene66673_All	5.490860573	Up	NADP-dependent malic enzyme-like protein [Locusta migratoria]
CL1949.Contig2_All	5.489339092	Up	aconitase 2 [Capsaspora owczarzaki ATCC 30864] >gi|320169225|gb|EFW46124.1| aconitase 2 [Capsaspora owczarzaki ATCC 30864]
Unigene40554_All	5.489273111	Up	PREDICTED: probable citrate synthase 1, mitochondrial-like [Amphimedon queenslandica]
CL1918.Contig2_All	5.489017836	Up	NADH dehydrogenase iron-sulfur protein 4, mitochondrial precursor [Caligus clemensi]
Unigene50740_All	5.488744096	Up	hypothetical protein CAPTEDRAFT_220007 [Capitella teleta]
CL3630.Contig2_All	5.47489412	Up	putative porphobilinogen deaminase [Danaus plexippus]
CL4796.Contig1_All	5.474560529	Up	GDP-mannose 4,6 dehydratase [Crassostrea gigas]
Unigene42649_All	5.464375896	Up	3-ketoacyl-CoA thiolase B, peroxisomal [Danio rerio] >gi|49257449|gb|AAH72706.1| Acetyl-Coenzyme A acyltransferase 1 [Danio rerio] >gi|182892130|gb|AAI65883.1| Acaa1 protein [Danio rerio]
CL5452.Contig2_All	5.460599709	Up	NADH dehydrogenase [ubiquinone] 1 alpha subcomplex subunit 2 [Bombyx mori] >gi|87248185|gb|ABD36145.1| NADH-ubiquinone oxidoreductase B8 subunit [Bombyx mori]
Unigene59770_All	5.460420782	Up	hypothetical protein CAPTEDRAFT_152363 [Capitella teleta]
Unigene40993_All	5.449723278	Up	6phosphofructokinase [Acanthamoeba castellanii str. Neff] >gi|440803962|gb|ELR24845.1| 6phosphofructokinase [Acanthamoeba castellanii str. Neff]
Unigene57918_All	5.42925347	Up	glucose-6-phosphate 1-dehydrogenase [Callorhinchus callorynchus]
Unigene41200_All	5.428646196	Up	--
CL5064.Contig1_All	5.419076424	Up	PREDICTED: formyltetrahydrofolate deformylase-like [Xenopus (Silurana) tropicalis]
CL3392.Contig2_All	5.414138166	Up	hypothetical protein CAPTEDRAFT_221879 [Capitella teleta]
Unigene54820_All	5.406365197	Up	myosin-VIIa [Capsaspora owczarzaki ATCC 30864] >gi|320163463|gb|EFW40362.1| myosin-VIIa [Capsaspora owczarzaki ATCC 30864]
Unigene67540_All	5.399335841	Up	hypothetical protein PTSG_11901 [Salpingoeca sp. ATCC 50818]
CL8189.Contig1_All	5.399329246	Up	hypothetical protein PTSG_12079 [Salpingoeca sp. ATCC 50818]
Unigene60671_All	5.39842744	Up	predicted protein [Capsaspora owczarzaki ATCC 30864] >gi|320168729|gb|EFW45628.1| predicted protein [Capsaspora owczarzaki ATCC 30864]
Unigene50449_All	5.393614367	Up	alanine aminotransferase [Capsaspora owczarzaki ATCC 30864] >gi|320163805|gb|EFW40704.1| alanine

			aminotransferase [Capsaspora owczarzaki ATCC 30864]
CL5044.Contig1_All	5.392277619	Up	similarity to endo-1-like protein [Leishmania braziliensis MHOM/BR/75/M2904] >gi|134066644|emb|CAM44443.1| similarity to endo-1-like protein [Leishmania braziliensis MHOM/BR/75/M2904]
Unigene43041_All	5.387763677	Up	PREDICTED: glucose dehydrogenase [acceptor] [Apis mellifera]
Unigene56251_All	5.3818835	Up	PREDICTED: phospholipase B1, membrane-associated-like [Megachile rotundata]
CL5697.Contig1_All	5.381696962	Up	carbamoyl-phosphate synthase large subunit [Capsaspora owczarzaki ATCC 30864] >gi|320164656|gb|EFW41555.1| carbamoyl-phosphate synthase large subunit [Capsaspora owczarzaki ATCC 30864]
CL1536.Contig1_All	5.367548865	Up	isocitrate dehydrogenase [Clonorchis sinensis]
Unigene41224_All	5.366742968	Up	PREDICTED: 3-isopropylmalate dehydratase-like [Amphimedon queenslandica]
CL8133.Contig2_All	5.365787382	Up	hypothetical protein BRAFLDRAFT_233353 [Branchiostoma floridae] >gi|229275371|gb|EEN46192.1| hypothetical protein BRAFLDRAFT_233353 [Branchiostoma floridae]
CL8352.Contig2_All	5.356521753	Up	malate dehydrogenase [Leishmania sp.] >gi|163963025|gb|ABY50464.1| malate dehydrogenase [Leishmania sp.] >gi|163963031|gb|ABY50467.1| malate dehydrogenase [Leishmania sp.] >gi|163963033|gb|ABY50468.1| malate dehydrogenase [Leishmania sp.] >gi|163963035|gb|ABY50469.1| malate dehydrogenase [Leishmania sp.] >gi|163963037|gb|ABY50470.1| malate dehydrogenase [Leishmania sp.] >gi|163963039|gb|ABY50471.1| malate dehydrogenase [Leishmania sp.] >gi|163963059|gb|ABY50481.1| malate dehydrogenase [Leishmania sp.] >gi|163963061|gb|ABY50482.1| malate dehydrogenase [Leishmania sp.]
Unigene42456_All	5.354123293	Up	RecName: Full=ATP synthase subunit beta, mitochondrial; Flags: Precursor >gi|391913|dbj|BAA04178.1| H(+)-transporting ATPase beta subunit [Hemicentrotus pulcherrimus] >gi|1094359|prf||2105433A H ATPase:SUBUNIT=beta
Unigene40930_All	5.347540501	Up	Transaldolase [Heterocephalus glaber]
CL1218.Contig2_All	5.334785904	Up	PREDICTED: uncharacterized short-chain type dehydrogenase/reductase y4vI-like [Xenopus (Silurana) tropicalis]
CL623.Contig1_All	5.334115275	Up	Chain I, Orientation Of Rna Polymerase Ii Within The Human Vp16-Mediator-Pol Ii-Tfiif Assembly

Unigene40716_All	5.333977658	Up	F1-ATP synthase beta subunit [Litopenaeus vannamei]
Unigene61377_All	5.333351425	Up	PREDICTED: levodione reductase-like [Amphimedon queenslandica]
Unigene57044_All	5.326338993	Up	ornithine--oxo-acid transaminase [Clonorchis sinensis]
CL8440.Contig2_All	5.323367588	Up	transaldolase [Danaus plexippus]
Unigene57135_All	5.323184971	Up	PREDICTED: fructose-1,6-bisphosphatase 1-like isoform 1 [Bombus terrestris] >gi|340727201|ref|XP_003401937.1| PREDICTED: fructose-1,6-bisphosphatase 1-like isoform 2 [Bombus terrestris]
Unigene63414_All	5.317059747	Up	PREDICTED: phosphoglucomutase-2-like [Hydra magnipapillata]
Unigene57481_All	5.312604354	Up	6-phosphofructokinase type C [Pteropus alecto]
CL2046.Contig1_All	5.307134098	Up	PREDICTED: v-type proton ATPase subunit d 1-like [Monodelphis domestica]
Unigene48523_All	5.301858995	Up	Chain A, Orientation Of Rna Polymerase Ii Within The Human Vp16-Mediator-Pol Ii-Tfiif Assembly
Unigene54908_All	5.30170788	Up	unknown [Dendroctonus ponderosae]
Unigene54542_All	5.300995591	Up	PREDICTED: similar to glyceraldehyde 3-phosphate dehydrogenase [Tribolium castaneum] >gi|270012069|gb|EFA08517.1| hypothetical protein TcasGA2_TC006170 [Tribolium castaneum]
Unigene40794_All	5.300157733	Up	Chain D, Structure Of The Apo Uracil Phosphoribosyltransferase, 2 Mutant C128v >gi|5107476|pdb|1BD3|C Chain C, Structure Of The Apo Uracil Phosphoribosyltransferase, 2 Mutant C128v >gi|5107477|pdb|1BD3|B Chain B, Structure Of The Apo Uracil Phosphoribosyltransferase, 2 Mutant C128v >gi|5107478|pdb|1BD3|A Chain A, Structure Of The Apo Uracil Phosphoribosyltransferase, 2 Mutant C128v >gi|5107479|pdb|1BD4|D Chain D, Uprt-Uracil Complex >gi|5107480|pdb|1BD4|C Chain C, Uprt-Uracil Complex >gi|5107481|pdb|1BD4|B Chain B, Uprt-Uracil Complex >gi|5107482|pdb|1BD4|A Chain A, Uprt-Uracil Complex >gi|18655843|pdb|1JLS|B Chain B, Structure Of The Uracil Phosphoribosyltransferase UracilCPR 2 MUTANT C128V >gi|18655844|pdb|1JLS|A Chain A, Structure Of The Uracil Phosphoribosyltransferase UracilCPR 2 MUTANT C128V >gi|18655845|pdb|1JLS|D Chain D, Structure Of The Uracil Phosphoribosyltransferase UracilCPR 2 MUTANT C128V >gi|18655846|pdb|1JLS|C Chain C, Structure Of The Uracil Phosphoribosyltransferase UracilCPR 2 MUTANT C128V >gi|18655950|pdb|1JLR|A Chain A, Structure Of The Uracil Phosphoribosyltransferase Gtp Complex 2 Mutant

			C128v >gi|18655951|pdb|1JLR|B Chain B, Structure Of The Uracil Phosphoribosyltransferase Gtp Complex 2 Mutant C128v >gi|18655952|pdb|1JLR|D Chain D, Structure Of The Uracil Phosphoribosyltransferase Gtp Complex 2 Mutant C128v >gi|18655953|pdb|1JLR|C Chain C, Structure Of The Uracil Phosphoribosyltransferase Gtp Complex 2 Mutant C128v
CL9794.Contig1_All	5.284206223	Up	4-hydroxyphenylpyruvate dioxygenase protein [Platynereis dumerilii]
CL8509.Contig1_All	5.278097923	Up	uncharacterized protein LOC100216301 [Xenopus (Silurana) tropicalis]
CL8439.Contig1_All	5.268231347	Up	predicted protein [Naegleria gruberi] >gi|284091150|gb|EFC44798.1| predicted protein [Naegleria gruberi]
CL2703.Contig2_All	5.267724472	Up	citrate synthase [Clonorchis sinensis]
Unigene53371_All	5.267187261	Up	transaldolase [Aedes aegypti] >gi|108874520|gb|EAT38745.1| AAEL009389-PA [Aedes aegypti]
CL6064.Contig1_All	5.267058056	Up	hypothetical protein BRAFLDRAFT_93657 [Branchiostoma floridae] >gi|229299000|gb|EEN69623.1| hypothetical protein BRAFLDRAFT_93657 [Branchiostoma floridae]
Unigene63496_All	5.266427334	Up	methylmalonate-semialdehyde dehydrogenase [Capsaspora owczarzaki ATCC 30864] >gi|320167930|gb|EFW44829.1| methylmalonate-semialdehyde dehydrogenase [Capsaspora owczarzaki ATCC 30864]
Unigene54869_All	5.255864186	Up	hypothetical protein SINV_04203 [Solenopsis invicta]
CL7279.Contig2_All	5.255822354	Up	Allantoicase [Crassostrea gigas]
Unigene41938_All	5.250475789	Up	PREDICTED: 6-phosphogluconolactonase-like [Cricetulus griseus]
Unigene58416_All	5.250215085	Up	testis-specific phosphoglycerate kinase 2, variant [Salpingoeca sp. ATCC 50818] >gi|326426700|gb|EGD72270.1| testis-specific phosphoglycerate kinase 2 [Salpingoeca sp. ATCC 50818]
CL5391.Contig1_All	5.233260348	Up	hypothetical protein CAPTEDRAFT_91181, partial [Capitella teleta]
Unigene41062_All	5.232812707	Up	hypothetical protein IMG5_100430 [Ichthyophthirius multifiliis] >gi|340505576|gb|EGR31893.1| hypothetical protein IMG5_100430 [Ichthyophthirius multifiliis]
CL3518.Contig1_All	5.232744622	Up	hypothetical protein DDB_G0275155 [Dictyostelium discoideum AX4] >gi|60471904|gb|EAL69858.1| hypothetical protein DDB_G0275155 [Dictyostelium discoideum AX4]
Unigene64124_All	5.232446086	Up	aconitate hydratase, mitochondrial, putative [Acanthamoeba castellanii str. Neff] >gi|440799601|gb|ELR20645.1| aconitate hydratase, mitochondrial, putative [Acanthamoeba castellanii str. Neff]

Unigene54576_All	5.231756971	Up	PREDICTED: o-acetylhomoserine (thiol)-lyase-like [Xenopus (Silurana) tropicalis]
CL6071.Contig1_All	5.231455356	Up	GMP synthetase, putative [Perkinsus marinus ATCC 50983] >gi|239868875|gb|EER00030.1| GMP synthetase, putative [Perkinsus marinus ATCC 50983]
Unigene70247_All	5.231072177	Up	glycoside hydrolase family 63 protein [Polysphondylium pallidum PN500]
Unigene42139_All	5.219900223	Up	hypothetical protein ACA1_369890 [Acanthamoeba castellanii str. Neff] >gi|440797151|gb|ELR18246.1| hypothetical protein ACA1_369890 [Acanthamoeba castellanii str. Neff]
CL3385.Contig2_All	5.215308284	Up	Homogentisate 1,2-dioxygenase [Camponotus floridanus]
Unigene56819_All	5.214705466	Up	PREDICTED: aconitate hydratase, mitochondrial-like [Oreochromis niloticus]
Unigene18479_All	5.212057958	Up	hypothetical protein DAPPUDRAFT_230594 [Daphnia pulex]
CL4009.Contig1_All	5.197105296	Up	Protein ACLY-2 [Caenorhabditis elegans] >gi|3873757|emb|CAB02690.1| Protein ACLY-2 [Caenorhabditis elegans]
Unigene68865_All	5.161265047	Up	hypothetical protein AND_00461 [Anopheles darlingi]
Unigene41385_All	5.161029718	Up	dihydrolipoyllysine-residue succinyltransferase component of 2-oxoglutarate dehydrogenase complex, mitochondrial [Xenopus laevis] >gi|28280000|gb|AAH45016.1| Dlst-prov protein [Xenopus laevis]
CL1204.Contig3_All	5.161000098	Up	PREDICTED: similar to ethanolamine-phosphate cytidylyltransferase [Tribolium castaneum] >gi|270003752|gb|EFA00200.1| hypothetical protein TcasGA2_TC003025 [Tribolium castaneum]
CL1226.Contig1_All	5.160289574	Up	PREDICTED: probable citrate synthase 1, mitochondrial-like [Acyrthosiphon pisum]
Unigene67002_All	5.123899556	Up	PREDICTED: phospholipase D1-like [Saccoglossus kowalevskii]
CL9429.Contig1_All	5.122353756	Up	carbamoyl-phosphate synthase large subunit [Capsaspora owczarzaki ATCC 30864] >gi|320164656|gb|EFW41555.1| carbamoyl-phosphate synthase large subunit [Capsaspora owczarzaki ATCC 30864]
Unigene43302_All	5.107793201	Up	hypothetical protein PTSG_08108 [Salpingoeca sp. ATCC 50818]
Unigene76032_All	5.086037555	Up	alpha-amylase I [Tribolium castaneum]
Unigene61689_All	5.085203334	Up	glucose/ribitol dehydrogenase family protein [Polysphondylium pallidum PN500]
Unigene44235_All	5.084695876	Up	alcohol dehydrogenase GroESlike domain containing protein [Acanthamoeba castellanii str. Neff] >gi|440792946|gb|ELR14151.1| alcohol dehydrogenase GroESlike domain containing protein [Acanthamoeba castellanii str. Neff]

CL3345.Contig1_All	5.08411894	Up	PREDICTED: similar to malate dehydrogenase [Tribolium castaneum] >gi|270008524|gb|EFA04972.1| hypothetical protein TcasGA2_TC015050 [Tribolium castaneum]
CL1310.Contig3_All	5.077187991	Up	ATP synthase subunit beta [Capsaspora owczarzaki ATCC 30864] >gi|320166885|gb|EFW43784.1| ATP synthase subunit beta [Capsaspora owczarzaki ATCC 30864]
CL5102.Contig1_All	5.046205129	Up	protein SEQ ID from patent family protein [Acanthamoeba castellanii str. Neff] >gi|440804729|gb|ELR25602.1| protein SEQ ID from patent family protein [Acanthamoeba castellanii str. Neff]
Unigene58848_All	5.045627569	Up	PREDICTED: acetyl-CoA acetyltransferase, mitochondrial-like [Hydra magnipapillata]
Unigene42085_All	5.045588491	Up	unnamed protein product [Oikopleura dioica]
Unigene46764_All	5.045520938	Up	PREDICTED: RNA-directed DNA polymerase from mobile element jockey-like [Hydra magnipapillata]
Unigene40715_All	5.045365304	Up	F1-ATP synthase beta subunit [Litopenaeus vannamei]
Unigene65119_All	5.045365304	Up	Heat shock protein 67B2 [Camponotus floridanus]
Unigene42515_All	5.044430826	Up	Ribose-phosphate pyrophosphokinase 1 [Pteropus alecto]
Unigene52166_All	5.044126706	Up	predicted protein [Nematostella vectensis] >gi|156227107|gb|EDO47912.1| predicted protein [Nematostella vectensis]
Unigene42230_All	5.043815914	Up	hypothetical protein CAPTEDRAFT_149787 [Capitella teleta]
CL2687.Contig1_All	5.034058224	Up	Formate dehydrogenase, putative [Perkinsus marinus ATCC 50983] >gi|239894020|gb|EER14209.1| Formate dehydrogenase, putative [Perkinsus marinus ATCC 50983]
Unigene58939_All	5.01205134	Up	1-phosphatidylinositol-4,5-bisphosphate phosphodiesterase epsilon-1 [Acromyrmex echinatior]
CL6836.Contig1_All	5.010283599	Up	2-oxoglutarate dehydrogenase [Salpingoeca sp. ATCC 50818]
Unigene44695_All	5.005044858	Up	hypothetical protein DAPPUDRAFT_301525 [Daphnia pulex]
Unigene69311_All	5.005041349	Up	GTP:AMP phosphotransferase mitochondrial [Harpegnathos saltator]
Unigene49952_All	5.005011168	Up	Ham1 family [Acanthamoeba castellanii str. Neff] >gi|440795226|gb|ELR16362.1| Ham1 family [Acanthamoeba castellanii str. Neff]
Unigene55201_All	5.00428538	Up	branched chain amino-acid transaminase 1, cytosolic [Xenopus (Silurana) tropicalis] >gi|112418510|gb|AAI21909.1| LOC100124778 protein [Xenopus (Silurana) tropicalis]
Unigene69490_All	5.004099777	Up	Succinate-semialdehyde dehydrogenase, mitochondrial [Crassostrea gigas]
Unigene48791_All	5.003898668	Up	hypothetical protein TTHERM_00497570 [Tetrahymena thermophila] >gi|89309715|gb|EAS07703.1|

			hypothetical protein TTHERM_00497570 [Tetrahymena thermophila SB210]
Unigene64350_All	5.003623595	Up	hypothetical protein [Monosiga brevicollis MX1] >gi|163773572|gb|EDQ87210.1| predicted protein [Monosiga brevicollis MX1]
CL478.Contig3_All	4.985396829	Up	hypothetical protein DICPUDRAFT_34436 [Dictyostelium purpureum] >gi|325081249|gb|EGC34771.1| hypothetical protein DICPUDRAFT_34436 [Dictyostelium purpureum]
CL6016.Contig1_All	4.979030226	Up	aminotransferase [Capsaspora owczarzaki ATCC 30864] >gi|320166795|gb|EFW43694.1| aminotransferase [Capsaspora owczarzaki ATCC 30864]
Unigene58796_All	4.963049839	Up	aconitate hydratase [Dictyostelium purpureum] >gi|325081369|gb|EGC34887.1| aconitate hydratase [Dictyostelium purpureum]
CL3178.Contig3_All	4.963029066	Up	PREDICTED: probable citrate synthase 1, mitochondrial-like [Amphimedon queenslandica]
Unigene60333_All	4.962173762	Up	predicted protein [Naegleria gruberi] >gi|284084467|gb|EFC38159.1| predicted protein [Naegleria gruberi]
Unigene42824_All	4.961262878	Up	homologous to 40kD subunit of RNA-polymerase I and III [Cricetulus griseus]
CL2364.Contig1_All	4.953808877	Up	hypothetical protein [Monosiga brevicollis MX1] >gi|163778618|gb|EDQ92233.1| predicted protein [Monosiga brevicollis MX1]
Unigene67507_All	4.920287939	Up	unnamed protein product [Tetraodon nigroviridis]
Unigene65077_All	4.919763111	Up	PREDICTED: ribose-phosphate pyrophosphokinase 1 isoform 2 [Gallus gallus] >gi|224098594|ref|XP_002187809.1| PREDICTED: ribose-phosphate pyrophosphokinase 1 isoform 1 [Taeniopygia guttata]
Unigene52095_All	4.919616079	Up	hypothetical protein CAPTEDRAFT_158431 [Capitella teleta]
Unigene65557_All	4.919595582	Up	Chain A, Orientation Of Rna Polymerase Ii Within The Human Vp16-Mediator-Pol Ii-Tfiif Assembly
CL3856.Contig1_All	4.919384128	Up	PREDICTED: ribulose-phosphate 3-epimerase-like [Amphimedon queenslandica]
Unigene67632_All	4.919116898	Up	dTDPD-glucose 4,6-dehydratase [Acanthamoeba castellanii str. Neff] >gi|440793013|gb|ELR14214.1| dTDPD-glucose 4,6-dehydratase [Acanthamoeba castellanii str. Neff]
Unigene48595_All	4.919040222	Up	Ribose-phosphate pyrophosphokinase 1 [Lepeophtheirus salmonis] >gi|290560958|gb|ADD37881.1| Ribose-phosphate pyrophosphokinase 1 [Lepeophtheirus salmonis]
Unigene56316_All	4.919025668	Up	1-pyrroline-5-carboxylate dehydrogenase [Clonorchis sinensis]
CL7732.Contig2_All	4.91899645	Up	4-aminobutyrate transaminase [Dictyostelium discoideum AX4] >gi|74897469|sp|Q55FI1.1|GABT_DICDI RecName: Full=4-aminobutyrate aminotransferase;

			AltName: Full=GABA aminotransferase; Short=GABA-AT; AltName: Full=Gamma-amino-N-butyrate transaminase; Short=GABA transaminase >gi|60475570|gb|EAL73505.1| 4-aminobutyrate transaminase [Dictyostelium discoideum AX4]
Unigene62479_All	4.918834227	Up	PREDICTED: AMP deaminase 2-like [Amphimedon queenslandica]
Unigene64118_All	4.918802713	Up	fumarate hydratase [Dictyostelium discoideum AX4] >gi|74897192|sp|Q54VA2.1|FUMH_DICDI RecName: Full=Fumarate hydratase, mitochondrial; Short=Fumarase; Flags: Precursor >gi|60469231|gb|EAL67226.1| fumarate hydratase [Dictyostelium discoideum AX4]
Unigene62445_All	4.918789179	Up	hypothetical protein BRAFLDRAFT_68404 [Branchiostoma floridae] >gi|229298781|gb|EEN69405.1| hypothetical protein BRAFLDRAFT_68404 [Branchiostoma floridae]
Unigene40643_All	4.902605959	Up	PREDICTED: glutamine synthetase-like [Saccoglossus kowalevskii]
CL5594.Contig2_All	4.897449563	Up	aminolevulinate dehydratase [Xenopus (Silurana) tropicalis] >gi|111598456|gb|AAH80497.1| delta-aminolevulinic acid dehydratase [Xenopus (Silurana) tropicalis]
Unigene50169_All	4.896678868	Up	4aminobutyrate aminotransferase [Acanthamoeba castellanii str. Neff] >gi|440794796|gb|ELR15946.1| 4aminobutyrate aminotransferase [Acanthamoeba castellanii str. Neff]
Unigene41268_All	4.87874707	Up	4-hydroxyphenylpyruvate dioxygenase family protein [Tetrahymena thermophila] >gi|3334226|sp|Q27203.1|HPPD_TETTH RecName: Full=4-hydroxyphenylpyruvate dioxygenase; AltName: Full=4-hydroxyphenylpyruvic acid oxidase; Short=4HPPD; Short=HPD; Short=HPPDase; AltName: Full=F-antigen homolog; AltName: Full=TF-AG >gi|161863|gb|AAA96492.1| F-antigen [Tetrahymena thermophila] >gi|89309572|gb|EAS07560.1| 4-hydroxyphenylpyruvate dioxygenase family protein [Tetrahymena thermophila SB210]
Unigene43096_All	4.875011239	Up	PREDICTED: aconitate hydratase, mitochondrial-like [Amphimedon queenslandica]
Unigene67308_All	4.874877062	Up	phosphoribosylformylglycinamidine synthase [Capsaspora owczarzaki ATCC 30864] >gi|320166620|gb|EFW43519.1| phosphoribosylformylglycinamidine synthase [Capsaspora owczarzaki ATCC 30864]
Unigene56987_All	4.874745465	Up	succinate dehydrogenase [Aedes aegypti] >gi|94468558|gb|ABF18128.1| mitochondrial succinate dehydrogenase cytochrome b subunit [Aedes aegypti] >gi|108875100|gb|EAT39325.1| AAEL008871-PA [Aedes aegypti]
Unigene57754_All	4.874697171	Up	pyruvate kinase [Capsaspora owczarzaki ATCC 30864] >gi|320167794|gb|EFW44693.1| pyruvate kinase

			[Capsaspora owczarzaki ATCC 30864]
Unigene41592_All	4.874387653	Up	PREDICTED: V-type proton ATPase catalytic subunit A-like [Hydra magnipapillata]
CL5233.Contig1_All	4.830650891	Up	Protein UBC-26 [Caenorhabditis elegans] >gi|351051497|emb|CCD73698.1| Protein UBC-26 [Caenorhabditis elegans]
Unigene42934_All	4.829621691	Up	Aminopeptidase N [Camponotus floridanus]
Unigene63427_All	4.829621691	Up	RNA polymerase II largest subunit [Ctenocephalides felis]
Unigene50323_All	4.829533737	Up	PREDICTED: long-chain-fatty-acid--CoA ligase 5-like isoform 2 [Takifugu rubripes]
Unigene64362_All	4.829269698	Up	CRE-ACO-2 protein [Caenorhabditis remanei] >gi|308262304|gb|EFP06257.1| CRE-ACO-2 protein [Caenorhabditis remanei]
Unigene59487_All	4.828804985	Up	aldehyde dehydrogenase [Salpingoeca sp. ATCC 50818]
CL5233.Contig2_All	4.827903453	Up	ubiquitin-conjugating enzyme e2, putative [Leishmania donovani] >gi|322502493|emb|CBZ37576.1| ubiquitin-conjugating enzyme e2, putative [Leishmania donovani]
Unigene57202_All	4.821668373	Up	Medium-chain specific acyl-CoA dehydrogenase, putative [Perkinsus marinus ATCC 50983] >gi|239902533|gb|EER19323.1| Medium-chain specific acyl-CoA dehydrogenase, putative [Perkinsus marinus ATCC 50983]
Unigene53372_All	4.817558141	Up	Transaldolase [Sycon ciliatum]
Unigene64332_All	4.782780738	Up	enolase [Heterorhabditis indica]
Unigene62120_All	4.782587285	Up	Protein F09F7.4, isoform a [Caenorhabditis elegans] >gi|351060667|emb|CCD68384.1| Protein F09F7.4, isoform a [Caenorhabditis elegans]
Unigene54256_All	4.781572015	Up	GM25688 [Drosophila sechellia] >gi|194119808|gb|EDW41851.1| GM25688 [Drosophila sechellia]
Unigene58691_All	4.78148602	Up	hypothetical protein CAPTEDRAFT_134102 [Capitella teleta]
Unigene61099_All	4.781359714	Up	catalase [Capsaspora owczarzaki ATCC 30864] >gi|320166530|gb|EFW43429.1| catalase [Capsaspora owczarzaki ATCC 30864]
Unigene68531_All	4.78107501	Up	hypothetical protein DICPUDRAFT_90278 [Dictyostelium purpureum] >gi|325076130|gb|EGC29943.1| hypothetical protein DICPUDRAFT_90278 [Dictyostelium purpureum]
Unigene45956_All	4.780799801	Up	nitric oxide synthase [Gryllus bimaculatus]
Unigene16813_All	4.769734731	Up	cytochrome c oxidase subunit 1 (mitochondrion) [Jakoba bahamiensis]
Unigene56931_All	4.768698621	Up	putative ubiquinol-cytochrome c reductase complex ubiquinone-binding protein QP-C [Graphocephala

			atropunctata]
Unigene61757_All	4.757982601	Up	catalase [Acanthamoeba castellanii str. Neff] >gi|372290526|gb|AEX91750.1| catalase 2 [Acanthamoeba castellanii] >gi|440792545|gb|ELR13756.1| catalase [Acanthamoeba castellanii str. Neff]
Unigene59883_All	4.745464747	Up	Probable cytochrome P450 304a1 [Camponotus floridanus]
Unigene65861_All	4.733473374	Up	putative prolyl-tRNA synthetase [Trypanosoma vivax Y486]
Unigene72524_All	4.732861196	Up	DNA polymerase epsilon catalytic subunit A [Myotis davidii]
Unigene72667_All	4.732186442	Up	PREDICTED: cysteine dioxygenase type 1-like [Metaseiulus occidentalis]
Unigene56956_All	4.727292267	Up	fructose-1-6-bisphosphatase [Capsaspora owczarzaki ATCC 30864] >gi|320168762|gb|EFW45661.1| fructose-1-6-bisphosphatase [Capsaspora owczarzaki ATCC 30864]
CL5084.Contig1_All	4.723111038	Up	expressed hypothetical protein [Trichoplax adhaerens] >gi|190587654|gb|EDV27696.1| expressed hypothetical protein [Trichoplax adhaerens]
CL3288.Contig2_All	4.713069029	Up	zinc-binding dehydrogenase [Capsaspora owczarzaki ATCC 30864] >gi|320165597|gb|EFW42496.1| zinc-binding dehydrogenase [Capsaspora owczarzaki ATCC 30864]
CL2887.Contig3_All	4.697649167	Up	cytochrome c oxidase subunit 1 [Monosiga brevicollis ATCC 50154] >gi|23344079|gb|AAN28355.1| cytochrome c oxidase subunit 1 (mitochondrion) [Monosiga brevicollis]
Unigene44487_All	4.683055009	Up	GF18942 [Drosophila ananassae] >gi|190628844|gb|EDV44368.1| GF18942 [Drosophila ananassae]
Unigene60205_All	4.682806783	Up	NADH-ubiquinone oxidoreductase 75 kDa subunit, mitochondrial [Danio rerio] >gi|55716039|gb|AAH85651.1| NADH dehydrogenase (ubiquinone) Fe-S protein 1 [Danio rerio]
Unigene70959_All	4.68277865	Up	HMGL-like family protein [Tetrahymena thermophila] >gi|89306979|gb|EAS04967.1| HMGL-like family protein [Tetrahymena thermophila SB210]
Unigene47625_All	4.682080339	Up	succinate dehydrogenase [Dictyostelium purpureum] >gi|325080618|gb|EGC34166.1| succinate dehydrogenase [Dictyostelium purpureum]
Unigene60068_All	4.68197719	Up	PREDICTED: thymidylate kinase-like isoform 2 [Oryzias latipes]
Unigene65260_All	4.681877495	Up	conserved domain protein [Trichinella spiralis] >gi|316975086|gb|EFV58545.1| conserved domain protein [Trichinella spiralis]
Unigene58762_All	4.681799801	Up	hypothetical protein BRAFLDRAFT_285351 [Branchiostoma floridae] >gi|229277578|gb|EEN48371.1| hypothetical protein BRAFLDRAFT_285351 [Branchiostoma floridae]
Unigene51623_All	4.681651003	Up	PREDICTED: serine dehydratase-like [Cricetulus griseus]

Unigene60562_All	4.681484574	Up	adenylate kinase, putative [Ixodes scapularis] >gi|215496559|gb|EEC06199.1| adenylate kinase, putative [Ixodes scapularis]
Unigene55425_All	4.676075861	Up	AGAP003168-PA [Anopheles gambiae str. PEST] >gi|333468507|gb|EAA08466.6| AGAP003168-PA [Anopheles gambiae str. PEST]
Unigene58834_All	4.656619168	Up	PREDICTED: short/branched chain specific acyl-CoA dehydrogenase, mitochondrial [Papio anubis]
Unigene60807_All	4.65650212	Up	PREDICTED: 1-phosphatidylinositol-4,5-bisphosphate phosphodiesterase gamma-1-like [Apis florea]
Unigene53890_All	4.656224089	Up	DNA-directed RNA polymerase II largest chain [Cryptosporidium hominis TU502] >gi|54657094|gb|EAL35927.1| DNA-directed RNA polymerase (EC 2.7.7.6) II largest chain [Cryptosporidium hominis]
Unigene57919_All	4.647998562	Up	hypothetical protein TcasGA2_TC000206 [Tribolium castaneum]
Unigene66677_All	4.630151274	Up	long-chain-fatty-acid--CoA ligase [Entamoeba dispar SAW760]
CL7900.Contig1_All	4.629820963	Up	adenosylhomocysteinase, putative [Pediculus humanus corporis] >gi|212508033|gb|EEB11852.1| adenosylhomocysteinase, putative [Pediculus humanus corporis]
CL6064.Contig3_All	4.629731603	Up	Mannose-1-phosphate guanyltransferase, putative [Pediculus humanus corporis] >gi|212514815|gb|EEB17065.1| Mannose-1-phosphate guanyltransferase, putative [Pediculus humanus corporis]
Unigene64154_All	4.629406812	Up	aldehyde dehydrogenase [Capsaspora owczarzaki ATCC 30864] >gi|320166834|gb|EFW43733.1| aldehyde dehydrogenase [Capsaspora owczarzaki ATCC 30864]
CL5139.Contig1_All	4.629387959	Up	PREDICTED: aspartate aminotransferase, mitochondrial-like [Hydra magnipapillata]
Unigene41031_All	4.629331962	Up	unnamed protein product [Tetraodon nigroviridis]
Unigene64933_All	4.629319539	Up	hypothetical protein DICPUDRAFT_48007 [Dictyostelium purpureum] >gi|325081384|gb|EGC34902.1| hypothetical protein DICPUDRAFT_48007 [Dictyostelium purpureum]
Unigene61111_All	4.629274595	Up	hypothetical protein CAPTEDRAFT_44911, partial [Capitella teleta]
Unigene45058_All	4.629174657	Up	PREDICTED: acetolactate synthase isozyme 2 large subunit-like [Bombus impatiens]
Unigene64291_All	4.629129411	Up	PREDICTED: glucosamine--fructose-6-phosphate aminotransferase [isomerizing] 2-like [Takifugu rubripes]
Unigene53826_All	4.619027139	Up	PREDICTED: similar to AGAP003501-PA [Tribolium castaneum] >gi|270005165|gb|EFA01613.1| hypothetical protein TcasGA2_TC007182 [Tribolium castaneum]

Unigene56971_All	4.605887922	Up	PREDICTED: cytochrome b-c1 complex subunit 7-like [Strongylocentrotus purpuratus] >gi|390346186|ref|XP_790701.3| PREDICTED: cytochrome b-c1 complex subunit 7-like [Strongylocentrotus purpuratus]
Unigene53843_All	4.602624927	Up	hypothetical protein KGM_00715 [Danaus plexippus]
Unigene55419_All	4.59353495	Up	PREDICTED: uncharacterized protein LOC100889850 [Strongylocentrotus purpuratus]
Unigene50701_All	4.576158232	Up	PREDICTED: probable C-5 sterol desaturase-like [Metaseiulus occidentalis]
CL7534.Contig1_All	4.57586637	Up	hypothetical protein BRAFLDRAFT_120901 [Branchiostoma floridae] >gi|229274258|gb|EEN45095.1| hypothetical protein BRAFLDRAFT_120901 [Branchiostoma floridae]
CL5650.Contig2_All	4.575754581	Up	PREDICTED: UDP-N-acetylhexosamine pyrophosphorylase-like [Meleagris gallopavo]
Unigene62304_All	4.575699129	Up	GE14984 [Drosophila yakuba] >gi|194187097|gb|EDX00681.1| GE14984 [Drosophila yakuba]
Unigene60792_All	4.575609409	Up	Rieske iron-sulfur protein 1 [Capsaspora owczarzaki ATCC 30864] >gi|320168907|gb|EFW45806.1| Rieske iron-sulfur protein 1 [Capsaspora owczarzaki ATCC 30864]
Unigene64084_All	4.575603306	Up	saccharopine dehydrogenase [Salpingoeca sp. ATCC 50818]
Unigene76268_All	4.574857623	Up	hypothetical protein CAPTEDRAFT_154512 [Capitella teleta]
CL2577.Contig1_All	4.574791891	Up	Peroxisomal multifunctional enzyme type 2 [Heterocephalus glaber]
Unigene69485_All	4.574759959	Up	PREDICTED: amidophosphoribosyltransferase-like [Xenopus (Silurana) tropicalis]
Unigene42590_All	4.574725392	Up	carbamoylphosphate synthase, large subunit, partial [Acanthamoeba castellanii str. Neff] >gi|440800294|gb|ELR21333.1| carbamoylphosphate synthase, large subunit, partial [Acanthamoeba castellanii str. Neff]
Unigene58516_All	4.553176867	Up	dihydrolipoyl dehydrogenase [Loa loa]
Unigene53886_All	4.547127538	Up	hypothetical protein TcasGA2_TC012309 [Tribolium castaneum]
Unigene49596_All	4.533675816	Up	Ceramide glucosyltransferase, putative [Pediculus humanus corporis] >gi|212516619|gb|EEB18613.1| Ceramide glucosyltransferase, putative [Pediculus humanus corporis]
Unigene57310_All	4.530276181	Up	RecName: Full=1,2-dihydroxy-3-keto-5-methylthiopentene dioxygenase; AltName: Full=Acireductone dioxygenase (Fe(2+)-requiring); Short=ARD; Short=Fe-ARD
CL3290.Contig1_All	4.529049477	Up	hCG1793893 [Homo sapiens]
Unigene44636_All	4.519533102	Up	PREDICTED: uncharacterized protein LOC100208015 [Hydra magnipapillata]
Unigene58072_All	4.519330472	Up	PREDICTED: choline/ethanolamine kinase-like [Bombus impatiens]

CL1819.Contig2_All	4.519256224	Up	hypothetical protein TcasGA2_TC004962 [Tribolium castaneum]
Unigene68397_All	4.519165297	Up	predicted protein [Naegleria gruberi] >gi|284088153|gb|EFC41818.1| predicted protein [Naegleria gruberi]
Unigene46304_All	4.519031196	Up	PREDICTED: cytochrome b-c1 complex subunit 2, mitochondrial [Monodelphis domestica]
Unigene61269_All	4.518821129	Up	PREDICTED: o-acetylhomoserine (thiol)-lyase-like [Xenopus (Silurana) tropicalis]
CL561.Contig2_All	4.518790083	Up	hypothetical protein DICPUDRAFT_48509 [Dictyostelium purpureum] >gi|325080636|gb|EGC34183.1| hypothetical protein DICPUDRAFT_48509 [Dictyostelium purpureum]
Unigene63504_All	4.518728802	Up	succinate-CoA ligase [Capsaspora owczarzaki ATCC 30864] >gi|320166382|gb|EFW43281.1| succinate-CoA ligase [Capsaspora owczarzaki ATCC 30864]
Unigene41982_All	4.51789523	Up	PREDICTED: similar to argininosuccinate synthetase [Tribolium castaneum] >gi|270001069|gb|EEZ97516.1| hypothetical protein TcasGA2_TC011361 [Tribolium castaneum]
Unigene75254_All	4.517744211	Up	RecName: Full=Serine palmitoyltransferase 1; AltName: Full=Long chain base biosynthesis protein 1; Short=LCB 1; AltName: Full=Serine-palmitoyl-CoA transferase 1; Short=SPT 1; Short=SPT1
Unigene49113_All	4.499093236	Up	PREDICTED: 1-phosphatidylinositol-4,5-bisphosphate phosphodiesterase epsilon-1-like [Nasonia vitripennis]
Unigene58438_All	4.48972611	Up	hypothetical protein CAPTEDRAFT_183731 [Capitella teleta]
Unigene57972_All	4.489577825	Up	hypothetical protein DDB_G0293470 [Dictyostelium discoideum AX4] >gi|60462491|gb|EAL60706.1| hypothetical protein DDB_G0293470 [Dictyostelium discoideum AX4]
CL727.Contig1_All	4.489562446	Up	phosphogluconate dehydrogenase [Eumesocampa frigilis]
CL7314.Contig1_All	4.48632127	Up	DNA-directed RNA polymerases I, II, and III subunit RPABC2 [Lepeophtheirus salmonis]
Unigene42395_All	4.47879585	Up	unknown [Dendroctonus ponderosae]
Unigene57053_All	4.477174661	Up	migration-inducing protein 4 [Capsaspora owczarzaki ATCC 30864] >gi|320169564|gb|EFW46463.1| migration-inducing protein 4 [Capsaspora owczarzaki ATCC 30864]
Unigene45619_All	4.460681652	Up	hypothetical protein BRAFLDRAFT_285351 [Branchiostoma floridae] >gi|229277578|gb|EEN48371.1| hypothetical protein BRAFLDRAFT_285351 [Branchiostoma floridae]
Unigene72126_All	4.46062599	Up	predicted protein [Nematostella vectensis] >gi|156224744|gb|EDO45567.1| predicted protein [Nematostella vectensis]
Unigene45972_All	4.46014813	Up	unnamed protein product [Tetraodon nigroviridis]

CL2414.Contig1_All	4.459757511	Up	Acetyl-CoA acetyltransferase,related [Neospora caninum Liverpool] >gi|325118059|emb|CBZ53610.1| Acetyl-CoA acetyltransferase,related [Neospora caninum Liverpool]
CL5231.Contig1_All	4.459532892	Up	shortchain dehydrogenase/reductase SDR, putative [Acanthamoeba castellanii str. Neff] >gi|440794861|gb|ELR16006.1| shortchain dehydrogenase/reductase SDR, putative [Acanthamoeba castellanii str. Neff]
CL4760.Contig1_All	4.43782851	Up	PREDICTED: ATP synthase subunit d, mitochondrial-like [Hydra magnipapillata]
Unigene49640_All	4.429930942	Up	mannose-1-phosphate guanyltransferase alpha-a [Ictalurus punctatus]
Unigene58893_All	4.414529839	Up	PREDICTED: putative glutamate synthase [NADPH]-like [Strongylocentrotus purpuratus]
CL2784.Contig3_All	4.405192166	Up	Choline/ethanolamine kinase [Harpegnathos saltator]
Unigene72836_All	4.399310626	Up	hypothetical protein BRAFLDRAFT_290854 [Branchiostoma floridae] >gi|229297260|gb|EEN67897.1| hypothetical protein BRAFLDRAFT_290854 [Branchiostoma floridae]
Unigene69346_All	4.399000858	Up	leukotriene a-4 hydrolase [Papilio polytes]
Unigene57942_All	4.398949111	Up	PREDICTED: similar to Sb:cb283 protein [Tribolium castaneum] >gi|270014120|gb|EFA10568.1| hypothetical protein TcasGA2_TC012824 [Tribolium castaneum]
Unigene50741_All	4.398515641	Up	predicted protein [Nematostella vectensis] >gi|156227682|gb|EDO48484.1| predicted protein [Nematostella vectensis]
CL4274.Contig3_All	4.398205982	Up	peroxisomal 3-ketoacyl-CoA thiolase B [Capsaspora owczarzaki ATCC 30864] >gi|320167663|gb|EFW44562.1| peroxisomal 3-ketoacyl-CoA thiolase B [Capsaspora owczarzaki ATCC 30864]
Unigene40812_All	4.390834213	Up	PREDICTED: succinate dehydrogenase [ubiquinone] iron-sulfur subunit, mitochondrial-like [Oreochromis niloticus]
Unigene12194_All	4.388626938	Up	aldose reductase, putative [Pediculus humanus corporis] >gi|212512105|gb|EEB14937.1| aldose reductase, putative [Pediculus humanus corporis]
Unigene49853_All	4.367184944	Up	Histone-lysine N-methyltransferase MLL4, partial [Heterocephalus glaber]
Unigene55010_All	4.361451944	Up	PREDICTED: UDP-GlcNAc:betaGal beta-1,3-N-acetylglucosaminyltransferase 5-like [Bombus impatiens]
CL6264.Contig1_All	4.360201907	Up	Long-chain-fatty-acid--CoA ligase, putative [Perkinsus marinus ATCC 50983] >gi|239896246|gb|EER15489.1| Long-chain-fatty-acid--CoA ligase, putative [Perkinsus marinus

			ATCC 50983]
CL7688.Contig1_All	4.356132926	Up	P11915 Nonspecific lipid-transfer protein [Polysphondylium pallidum PN500]
Unigene56818_All	4.355977495	Up	hypothetical protein AND_05986 [Anopheles darlingi]
Unigene76210_All	4.334710502	Up	4-aminobutyrate transaminase [Dictyostelium discoideum AX4] >gi|74897469|sp|Q55FI1.1|GABT_DICDI RecName: Full=4-aminobutyrate aminotransferase; AltName: Full=GABA aminotransferase; Short=GABA-AT; AltName: Full=Gamma-amino-N-butyrate transaminase; Short=GABA transaminase >gi|60475570|gb|EAL73505.1| 4-aminobutyrate transaminase [Dictyostelium discoideum AX4]
Unigene44128_All	4.334458346	Up	vacuolar proton ATPase [Danaus plexippus]
Unigene75708_All	4.33437627	Up	PREDICTED: 2-oxoglutarate dehydrogenase, mitochondrial-like [Metaseiulus occidentalis]
Unigene70915_All	4.334046818	Up	tyrosine transaminase [Dictyostelium fasciculatum] >gi|328872629|gb|EGG20996.1| tyrosine transaminase [Dictyostelium fasciculatum]
Unigene70900_All	4.333816769	Up	Aldehyde dehydrogenase, putative [Pediculus humanus corporis] >gi|212511600|gb|EEB14529.1| Aldehyde dehydrogenase, putative [Pediculus humanus corporis]
Unigene63139_All	4.333788883	Up	unnamed protein product [Oikopleura dioica]
Unigene45371_All	4.333751859	Up	PREDICTED: allantoinase, mitochondrial-like [Takifugu rubripes]
Unigene42136_All	4.317807405	Up	PREDICTED: isocitrate dehydrogenase [NAD] subunit 1, mitochondrial-like [Amphimedon queenslandica]
Unigene10413_All	4.316051014	Up	UDP-n-acteylglucosamine pyrophosphorylase [Culex quinquefasciatus] >gi|167874835|gb|EDS38218.1| UDP-n-acteylglucosamine pyrophosphorylase [Culex quinquefasciatus]
Unigene64868_All	4.300711609	Up	PREDICTED: fatty aldehyde dehydrogenase-like [Sus scrofa]
CL1310.Contig6_All	4.297874078	Up	F1-ATP synthase beta subunit [Litopenaeus vannamei]
Unigene54850_All	4.290251264	Up	phospholipase b, plb1 [Culex quinquefasciatus] >gi|167880884|gb|EDS44267.1| phospholipase b, plb1 [Culex quinquefasciatus]
Unigene65096_All	4.289669367	Up	lipoic acid synthetase precursor [Polysphondylium pallidum PN500]
CL3645.Contig2_All	4.274163999	Up	aminomethyltransferase,putative [Pediculus humanus corporis] >gi|212505419|gb|EEB09880.1| aminomethyltransferase,putative [Pediculus humanus corporis]
Unigene57153_All	4.273987957	Up	pyruvate carboxylase [Capsaspora owczarzaki ATCC 30864] >gi|320168344|gb|EFW45243.1| pyruvate

<table>
<tr><td></td><td></td><td></td><td>carboxylase [Capsaspora owczarzaki ATCC 30864]</td></tr>
<tr><td>Unigene40960_All</td><td>4.267491013</td><td>Up</td><td>unnamed protein product [Oikopleura dioica]</td></tr>
<tr><td>Unigene69206_All</td><td>4.267281688</td><td>Up</td><td>PREDICTED: acetyl-coenzyme A synthetase-like [Bombus impatiens]</td></tr>
<tr><td>Unigene42809_All</td><td>4.267199782</td><td>Up</td><td>PREDICTED: similar to reverse transcriptase-like protein, partial [Tribolium castaneum]</td></tr>
<tr><td>Unigene59031_All</td><td>4.267198164</td><td>Up</td><td>hypothetical protein TRIADDRAFT_27680 [Trichoplax adhaerens] >gi|190583544|gb|EDV23615.1| hypothetical protein TRIADDRAFT_27680 [Trichoplax adhaerens]</td></tr>
<tr><td>Unigene64062_All</td><td>4.267059452</td><td>Up</td><td>aminolevulinate synthase [Papilio xuthus]</td></tr>
<tr><td>Unigene46798_All</td><td>4.266895258</td><td>Up</td><td>AcylCoA oxidase [Acanthamoeba castellanii str. Neff] >gi|440789742|gb|ELR11041.1| AcylCoA oxidase [Acanthamoeba castellanii str. Neff]</td></tr>
<tr><td>CL5386.Contig2_All</td><td>4.266826833</td><td>Up</td><td>GI22624 [Drosophila mojavensis] >gi|193916847|gb|EDW15714.1| GI22624 [Drosophila mojavensis]</td></tr>
<tr><td>CL1182.Contig1_All</td><td>4.220726684</td><td>Up</td><td>acetylCoA acyltransferase, putative [Acanthamoeba castellanii str. Neff] >gi|440796676|gb|ELR17785.1| acetylCoA acyltransferase, putative [Acanthamoeba castellanii str. Neff]</td></tr>
<tr><td>Unigene58947_All</td><td>4.220614075</td><td>Up</td><td>PREDICTED: uncharacterized protein LOC100685098 [Canis lupus familiaris]</td></tr>
<tr><td>CL3145.Contig2_All</td><td>4.211595663</td><td>Up</td><td>ATP synthase subunit alpha, mitochondrial [Danio rerio] >gi|115528192|gb|AAI24812.1| ATP synthase, H+ transporting, mitochondrial F1 complex, alpha subunit 1, cardiac muscle [Danio rerio]</td></tr>
<tr><td>Unigene42933_All</td><td>4.208450047</td><td>Up</td><td>NADH-ubiquinone oxidoreductase flavoprotein 1 (ndufv1) [Aedes aegypti] >gi|108876370|gb|EAT40595.1| AAEL007681-PA [Aedes aegypti]</td></tr>
<tr><td>Unigene42701_All</td><td>4.197628337</td><td>Up</td><td>Succinate-semialdehyde dehydrogenase, mitochondrial [Crassostrea gigas]</td></tr>
<tr><td>Unigene76092_All</td><td>4.197265522</td><td>Up</td><td>CG10932 [Drosophila melanogaster] >gi|7290840|gb|AAF46282.1| CG10932 [Drosophila melanogaster] >gi|19528343|gb|AAL90286.1| LD24105p [Drosophila melanogaster]</td></tr>
<tr><td>Unigene60344_All</td><td>4.197030414</td><td>Up</td><td>hypothetical protein AND_07551 [Anopheles darlingi]</td></tr>
<tr><td>Unigene65593_All</td><td>4.19696342</td><td>Up</td><td>DNAdirected RNA polymerase, beta subunit [Acanthamoeba castellanii str. Neff] >gi|440793499|gb|ELR14681.1| DNAdirected RNA polymerase, beta subunit [Acanthamoeba castellanii str. Neff]</td></tr>
<tr><td>Unigene41749_All</td><td>4.196881942</td><td>Up</td><td>malate dehydrogenase precursor [Haliotis discus discus]</td></tr>
<tr><td>Unigene69941_All</td><td>4.196847762</td><td>Up</td><td>alcohol dehydrogenase class-3 [Danio rerio] >gi|45595752|gb|AAH67170.1| Alcohol dehydrogenase 5 [Danio rerio]</td></tr>
<tr><td>Unigene56947_All</td><td>4.190774548</td><td>Up</td><td>hypothetical protein AND_17060 [Anopheles darlingi]</td></tr>
</table>

Unigene56499_All	4.172650205	Up	PREDICTED: dihydropyrimidine dehydrogenase [NADP(+)] [Ciona intestinalis]
Unigene66282_All	4.159814551	Up	NADH:ubiquinone oxidoreductase, putative [Ixodes scapularis] >gi|215492875|gb|EEC02516.1| NADH:ubiquinone oxidoreductase, putative [Ixodes scapularis]
CL8264.Contig1_All	4.124932155	Up	aconitase 2 [Capsaspora owczarzaki ATCC 30864] >gi|320169225|gb|EFW46124.1| aconitase 2 [Capsaspora owczarzaki ATCC 30864]
CL962.Contig2_All	4.12282937	Up	seminal fluid protein HACP047 [Heliconius erato]
Unigene65415_All	4.122788785	Up	voltage gated chloride channel domain-containing protein [Toxoplasma gondii ME49] >gi|211966343|gb|EEB01539.1| voltage gated chloride channel domain-containing protein [Toxoplasma gondii ME49]
Unigene41044_All	4.122568294	Up	unnamed protein product [Oikopleura dioica]
Unigene42093_All	4.097184764	Up	PREDICTED: uncharacterized protein LOC594361, partial [Strongylocentrotus purpuratus]
Unigene42165_All	4.092026522	Up	V-type H+ ATPase subunit E [Capsaspora owczarzaki ATCC 30864] >gi|320169729|gb|EFW46628.1| V-type H+ ATPase subunit E [Capsaspora owczarzaki ATCC 30864]
Unigene42707_All	4.084311589	Up	NADH dehydrogenase 1 alpha subcomplex subunit 4 [Dicentrarchus labrax]
Unigene42354_All	4.045614158	Up	sphingosine-1-phosphate lyase 1 [Capsaspora owczarzaki ATCC 30864] >gi|320166604|gb|EFW43503.1| sphingosine-1-phosphate lyase 1 [Capsaspora owczarzaki ATCC 30864]
CL7813.Contig2_All	4.045365304	Up	vacuolar ATP synthase proteolipid subunit [Trichinella spiralis] >gi|316979348|gb|EFV62153.1| vacuolar ATP synthase proteolipid subunit [Trichinella spiralis]
Unigene42025_All	4.045365304	Up	PREDICTED: aldehyde dehydrogenase, mitochondrial-like [Monodelphis domestica]
Unigene52007_All	4.044671316	Up	uricase [Danio rerio] >gi|49900519|gb|AAH76466.1| Urate oxidase [Danio rerio] >gi|182888674|gb|AAI64061.1| Uox protein [Danio rerio]
Unigene62549_All	4.044572792	Up	voltage gated chloride channel domain-containing protein [Toxoplasma gondii ME49] >gi|211966343|gb|EEB01539.1| voltage gated chloride channel domain-containing protein [Toxoplasma gondii ME49]
CL2392.Contig2_All	4.044517728	Up	2-oxoglutarate dehydrogenase, putative [Pediculus humanus corporis] >gi|212511538|gb|EEB14492.1| 2-oxoglutarate dehydrogenase, putative [Pediculus humanus corporis]
Unigene52835_All	4.04431434	Up	--
Unigene58113_All	4.018020141	Up	PREDICTED: 6-phosphogluconate dehydrogenase, decarboxylating-like [Nasonia vitripennis]

Unigene63053_All	4.004650205	Up	putative NADH-dependent fumarate reductase [Leishmania major strain Friedlin] >gi|323363740|emb|CBZ12746.1| putative NADH-dependent fumarate reductase [Leishmania major strain Friedlin]
CL8392.Contig1_All	4.004103218	Up	phenylalanine hydroxylase [Polyrhachis vicina]
Unigene52333_All	4.004083298	Up	cytochrome c oxidase polypeptide VIb [Salpingoeca sp. ATCC 50818]
Unigene7595_All	3.999629522	Up	phenylalanine-tryptophan hydroxylase [Gryllus bimaculatus]
Unigene60713_All	3.990349168	Up	PREDICTED: long-chain-fatty-acid--CoA ligase 3 [Strongylocentrotus purpuratus]
Unigene53359_All	3.983352706	Up	Atp6v0c protein [Capsaspora owczarzaki ATCC 30864] >gi|320168466|gb|EFW45365.1| Atp6v0c protein [Capsaspora owczarzaki ATCC 30864]
CL8442.Contig1_All	3.979199954	Up	Chain B, Orientation Of Rna Polymerase Ii Within The Human Vp16-Mediator-Pol Ii-Tfiif Assembly
Unigene68328_All	3.962962621	Up	Glucose-6-phosphate 1-dehydrogenase [Pteropus alecto]
Unigene65749_All	3.96274142	Up	dihydrolipoyl dehydrogenase [Loa loa]
Unigene52171_All	3.962732837	Up	hypothetical protein DICPUDRAFT_58919 [Dictyostelium purpureum] >gi|325075333|gb|EGC29234.1| hypothetical protein DICPUDRAFT_58919 [Dictyostelium purpureum]
Unigene58741_All	3.96266411	Up	Ethanolamine-phosphate cytidylyltransferase [Crassostrea gigas]
Unigene57867_All	3.962613111	Up	AcylCoA oxidase [Acanthamoeba castellanii str. Neff] >gi|440789742|gb|ELR11041.1| AcylCoA oxidase [Acanthamoeba castellanii str. Neff]
CL4597.Contig1_All	3.96204826	Up	hydroxymethyltransferase [Naegleria gruberi] >gi|284096811|gb|EFC50438.1| hydroxymethyltransferase [Naegleria gruberi]
Unigene66727_All	3.962026484	Up	phosphoglucomutase [Drosophila melanogaster]
Unigene71331_All	3.961877131	Up	hypothetical protein CAPTEDRAFT_220999 [Capitella teleta]
Unigene73581_All	3.961720988	Up	serine palmitoyltransferase subunit 1 [Capsaspora owczarzaki ATCC 30864] >gi|320164847|gb|EFW41746.1| serine palmitoyltransferase subunit 1 [Capsaspora owczarzaki ATCC 30864]
Unigene45649_All	3.961719606	Up	unnamed protein product [Mus musculus]
Unigene72271_All	3.961679154	Up	PREDICTED: RNA-directed DNA polymerase from mobile element jockey-like [Strongylocentrotus purpuratus]
Unigene71350_All	3.961576388	Up	PREDICTED: v-type proton ATPase subunit C-like [Amphimedon queenslandica]

<table>
<tr><td>Unigene74480_All</td><td>3.961548897</td><td>Up</td><td>2,3-diphosphoglycerate-independent phosphoglycerate mutase [Dirofilaria immitis]</td></tr>
<tr><td>Unigene55517_All</td><td>3.941240655</td><td>Up</td><td>PREDICTED: uncharacterized protein LOC100874903 [Megachile rotundata]</td></tr>
<tr><td>Unigene57436_All</td><td>3.933628916</td><td>Up</td><td>ribonucleotide reductase, beta subunit, putative [Ixodes scapularis] >gi|215494664|gb|EEC04305.1| ribonucleotide reductase, beta subunit, putative [Ixodes scapularis]</td></tr>
<tr><td>Unigene57224_All</td><td>3.925497972</td><td>Up</td><td>ACYPI000747 [Acyrthosiphon pisum]</td></tr>
<tr><td>CL3756.Contig2_All</td><td>3.919411653</td><td>Up</td><td>putative histone acetyltransferase [Danaus plexippus]</td></tr>
<tr><td>CL9605.Contig1_All</td><td>3.905056518</td><td>Up</td><td>ribose-phosphate pyrophosphokinase 1 isoform 1 [Danio rerio] >gi|42542750|gb|AAH66485.1| Phosphoribosyl pyrophosphate synthetase 1A [Danio rerio]</td></tr>
<tr><td>Unigene57226_All</td><td>3.90166552</td><td>Up</td><td>PREDICTED: putative glutamate synthase [NADPH]-like [Nasonia vitripennis]</td></tr>
<tr><td>Unigene53887_All</td><td>3.889817082</td><td>Up</td><td>PREDICTED: glycogen debranching enzyme-like [Nasonia vitripennis]</td></tr>
<tr><td>Unigene57002_All</td><td>3.882374112</td><td>Up</td><td>oxidase heme a,cytochrome [Salpingoeca sp. ATCC 50818]</td></tr>
<tr><td>CL585.Contig2_All</td><td>3.875728152</td><td>Up</td><td>putative CAD trifunctional protein, partial [Campichoeta punctum]</td></tr>
<tr><td>Unigene73180_All</td><td>3.875429905</td><td>Up</td><td>PREDICTED: acetyl-CoA carboxylase 2 [Trichechus manatus latirostris]</td></tr>
<tr><td>Unigene75849_All</td><td>3.875196163</td><td>Up</td><td>hypothetical protein DICPUDRAFT_59190 [Dictyostelium purpureum] >gi|325074815|gb|EGC28796.1| hypothetical protein DICPUDRAFT_59190 [Dictyostelium purpureum]</td></tr>
<tr><td>Unigene77445_All</td><td>3.875097114</td><td>Up</td><td>glucose phosphate isomerase [Apatelodes torrefacta]</td></tr>
<tr><td>Unigene47232_All</td><td>3.875016026</td><td>Up</td><td>--</td></tr>
<tr><td>Unigene67953_All</td><td>3.874964328</td><td>Up</td><td>PREDICTED: phosphatidate phosphatase PPAPDC1B-like [Oryzias latipes]</td></tr>
<tr><td>Unigene59690_All</td><td>3.874953925</td><td>Up</td><td>hypothetical protein TcasGA2_TC014935 [Tribolium castaneum]</td></tr>
<tr><td>Unigene69945_All</td><td>3.874573205</td><td>Up</td><td>hypothetical protein DICPUDRAFT_51014 [Dictyostelium purpureum] >gi|325076136|gb|EGC29949.1| hypothetical protein DICPUDRAFT_51014 [Dictyostelium purpureum]</td></tr>
<tr><td>CL8133.Contig1_All</td><td>3.874268563</td><td>Up</td><td>--</td></tr>
<tr><td>Unigene53825_All</td><td>3.866612905</td><td>Up</td><td>PREDICTED: lipase 3-like [Nasonia vitripennis]</td></tr>
<tr><td>Unigene57449_All</td><td>3.856903016</td><td>Up</td><td>PREDICTED: polypeptide N-acetylgalactosaminyltransferase 3-like isoform 1 [Acyrthosiphon pisum] >gi|328723400|ref|XP_003247833.1| PREDICTED: polypeptide N-acetylgalactosaminyltransferase 3-like isoform 2 [Acyrthosiphon pisum]</td></tr>
<tr><td>Unigene67823_All</td><td>3.829084679</td><td>Up</td><td>FAD synthetase [Crassostrea gigas]</td></tr>
<tr><td>Unigene58681_All</td><td>3.80892168</td><td>Up</td><td>juvenile hormone esterase-like protein Est1 [Reticulitermes flavipes]</td></tr>
</table>

CL7688.Contig2_All	3.800870565	Up	PREDICTED: peroxisomal multifunctional enzyme type 2-like [Anolis carolinensis]
Unigene43234_All	3.788193508	Up	PREDICTED: RNA-directed DNA polymerase from mobile element jockey-like [Strongylocentrotus purpuratus]
CL8312.Contig1_All	3.784800059	Up	PREDICTED: N-acetylglucosaminyl-phosphatidylinositol de-N-acetylase-like [Bombus impatiens]
Unigene47126_All	3.782154368	Up	CG1746, isoform A [Drosophila melanogaster] >gi|24651601|ref|NP_733422.1| CG1746, isoform B [Drosophila melanogaster] >gi|24651603|ref|NP_733423.1| CG1746, isoform C [Drosophila melanogaster] >gi|386766809|ref|NP_001247382.1| CG1746, isoform E [Drosophila melanogaster] >gi|386766813|ref|NP_001247384.1| CG1746, isoform G [Drosophila melanogaster] >gi|194905019|ref|XP_001981105.1| GG11879 [Drosophila erecta] >gi|7302028|gb|AAF57131.1| CG1746, isoform B [Drosophila melanogaster] >gi|23172756|gb|AAN14267.1| CG1746, isoform A [Drosophila melanogaster] >gi|23172757|gb|AAN14268.1| CG1746, isoform C [Drosophila melanogaster] >gi|41058227|gb|AAR99150.1| GM13193p [Drosophila melanogaster] >gi|190655743|gb|EDV52975.1| GG11879 [Drosophila erecta] >gi|383293039|gb|AFH06699.1| CG1746, isoform E [Drosophila melanogaster] >gi|383293041|gb|AFH06701.1| CG1746, isoform G [Drosophila melanogaster]
Unigene77193_All	3.781914337	Up	uncharacterized protein LOC100216301 [Xenopus (Silurana) tropicalis]
Unigene57067_All	3.781867834	Up	--
Unigene48678_All	3.781861525	Up	Glycine dehydrogenase [decarboxylating], mitochondrial [Camponotus floridanus]
Unigene63590_All	3.781813463	Up	hypothetical protein TRIADDRAFT_20946 [Trichoplax adhaerens] >gi|190588269|gb|EDV28311.1| hypothetical protein TRIADDRAFT_20946 [Trichoplax adhaerens]
Unigene49135_All	3.781753685	Up	GH17900 [Drosophila grimshawi] >gi|193896488|gb|EDV95354.1| GH17900 [Drosophila grimshawi]
Unigene64254_All	3.781748842	Up	epoxide hydrolase 2 [Danio rerio] >gi|56269293|gb|AAH86714.1| Epoxide hydrolase 2, cytoplasmic [Danio rerio] >gi|182890020|gb|AAI65172.1| Ephx2 protein [Danio rerio]
Unigene59588_All	3.781739006	Up	PREDICTED: succinate-CoA ligase, ADP-forming, beta subunit-like [Saccoglossus kowalevskii]
Unigene46008_All	3.781496247	Up	uncharacterized protein LOC100216301 [Xenopus (Silurana) tropicalis]
Unigene71413_All	3.781310499	Up	cysteine dioxygenase type 1 [Salmo salar] >gi|209737796|gb|ACI69767.1| Cysteine dioxygenase type 1 [Salmo salar]

Unigene59719_All	3.765767021	Up	AAEL001273-PA [Aedes aegypti]
Unigene47426_All	3.765665654	Up	predicted protein [Nematostella vectensis] >gi|156217801|gb|EDO38711.1| predicted protein [Nematostella vectensis]
Unigene56436_All	3.760894071	Up	PREDICTED: DNA-directed RNA polymerase III subunit RPC1-like [Bombus terrestris]
Unigene58349_All	3.749436819	Up	proteophosphoglycan ppg1 [Leishmania mexicana MHOM/GT/2001/U1103] >gi|322495303|emb|CBZ30607.1| proteophosphoglycan ppg1 [Leishmania mexicana MHOM/GT/2001/U1103]
Unigene60932_All	3.749246355	Up	PREDICTED: acyl-Coenzyme A dehydrogenase, C-4 to C-12 straight chain-like [Saccoglossus kowalevskii]
Unigene56602_All	3.743761857	Up	hypothetical protein TcasGA2_TC010001 [Tribolium castaneum]
Unigene43640_All	3.743227554	Up	PREDICTED: exosome component 10-like [Megachile rotundata]
Unigene57212_All	3.741305861	Up	PREDICTED: glutamine synthetase-like [Oryzias latipes]
Unigene55795_All	3.732987231	Up	PREDICTED: similar to pyridoxal kinase [Tribolium castaneum] >gi|270003960|gb|EFA00408.1| hypothetical protein TcasGA2_TC003259 [Tribolium castaneum]
Unigene51717_All	3.732967721	Up	hypothetical protein NEMVEDRAFT_v1g152068 [Nematostella vectensis] >gi|156201946|gb|EDO27109.1| predicted protein [Nematostella vectensis]
Unigene74468_All	3.732694241	Up	seed maturation protein PM34, putative [Acanthamoeba castellanii str. Neff] >gi|440804090|gb|ELR24970.1| seed maturation protein PM34, putative [Acanthamoeba castellanii str. Neff]
Unigene57794_All	3.722872623	Up	PREDICTED: ribonucleoside-diphosphate reductase subunit M2-like isoform 1 [Strongylocentrotus purpuratus]
Unigene49390_All	3.721729259	Up	PREDICTED: pancreatic triacylglycerol lipase-like [Acyrthosiphon pisum]
Unigene57773_All	3.716303418	Up	1-acyl-sn-glycerol-3-phosphate acyltransferase alpha [Tupaia chinensis]
Unigene58431_All	3.716195252	Up	hypothetical protein [Monosiga brevicollis MX1] >gi|163774028|gb|EDQ87662.1| predicted protein [Monosiga brevicollis MX1]
Unigene52136_All	3.714520693	Up	PREDICTED: DNA-directed RNA polymerase I subunit RPA2-like [Bombus impatiens]
CL3296.Contig1_All	3.695095142	Up	testis-specific phosphoglycerate kinase 2, variant [Salpingoeca sp. ATCC 50818] >gi|326426700|gb|EGD72270.1| testis-specific phosphoglycerate kinase 2 [Salpingoeca sp. ATCC

<table>
<tr><td></td><td></td><td></td><td>50818]</td></tr>
<tr><td>Unigene40640_All</td><td>3.682497983</td><td>Up</td><td>uncharacterized protein LOC100216301 [Xenopus (Silurana) tropicalis]</td></tr>
<tr><td>Unigene60466_All</td><td>3.682342841</td><td>Up</td><td>PREDICTED: 72 kDa inositol polyphosphate 5-phosphatase-like [Megachile rotundata]</td></tr>
<tr><td>Unigene67737_All</td><td>3.682184308</td><td>Up</td><td>retinal dehydrogenase 2 [Trichinella spiralis] >gi|316975779|gb|EFV59178.1| retinal dehydrogenase 2 [Trichinella spiralis]</td></tr>
<tr><td>Unigene58393_All</td><td>3.682133995</td><td>Up</td><td>ribose-phosphate pyrophosphokinase [Trichinella spiralis] >gi|316974759|gb|EFV58235.1| ribose-phosphate pyrophosphokinase [Trichinella spiralis]</td></tr>
<tr><td>Unigene41041_All</td><td>3.657434265</td><td>Up</td><td>methylglutaconyl-CoA hydratase [Capsaspora owczarzaki ATCC 30864] >gi|320165650|gb|EFW42549.1| methylglutaconyl-CoA hydratase [Capsaspora owczarzaki ATCC 30864]</td></tr>
<tr><td>Unigene51270_All</td><td>3.629785165</td><td>Up</td><td>glutamine synthetase 1 [Procambarus clarkii]</td></tr>
<tr><td>Unigene69441_All</td><td>3.62961883</td><td>Up</td><td>3'-phosphoadenosine 5'-phosphosulfate synthase 1 [Xenopus (Silurana) tropicalis] >gi|49523166|gb|AAH75507.1| 3'-phosphoadenosine 5'-phosphosulfate synthase 1 [Xenopus (Silurana) tropicalis]</td></tr>
<tr><td>Unigene61999_All</td><td>3.611855466</td><td>Up</td><td>AGAP002711-PF [Anopheles gambiae str. PEST] >gi|333468019|gb|EGK96796.1| AGAP002711-PF [Anopheles gambiae str. PEST]</td></tr>
<tr><td>Unigene57263_All</td><td>3.606879976</td><td>Up</td><td>5-methyltetrahydropteroyltriglutamate-homocysteine methyltransferase [Capsaspora owczarzaki ATCC 30864] >gi|320169714|gb|EFW46613.1| 5-methyltetrahydropteroyltriglutamate-homocysteine methyltransferase [Capsaspora owczarzaki ATCC 30864]</td></tr>
<tr><td>Unigene52340_All</td><td>3.593710972</td><td>Up</td><td>glucosyl glucuronosyl transferases [Locusta migratoria]</td></tr>
<tr><td>Unigene47332_All</td><td>3.591249708</td><td>Up</td><td>ATP synthase, H+ transporting, mitochondrial F1 complex, O subunit [Acyrthosiphon pisum]</td></tr>
<tr><td>Unigene48358_All</td><td>3.575636975</td><td>Up</td><td>cystathionine gammasynthase [Acanthamoeba castellanii str. Neff] >gi|440793285|gb|ELR14472.1| cystathionine gammasynthase [Acanthamoeba castellanii str. Neff]</td></tr>
<tr><td>Unigene57860_All</td><td>3.57557658</td><td>Up</td><td>Ceramide glucosyltransferase [Harpegnathos saltator]</td></tr>
<tr><td>Unigene57107_All</td><td>3.575453484</td><td>Up</td><td>PREDICTED: NADH dehydrogenase [ubiquinone] 1 alpha subcomplex subunit 9, mitochondrial-like [Takifugu rubripes]</td></tr>
<tr><td>Unigene54321_All</td><td>3.575213792</td><td>Up</td><td>hypothetical protein DICPUDRAFT_30850 [Dictyostelium purpureum] >gi|325083650|gb|EGC37097.1| hypothetical protein DICPUDRAFT_30850 [Dictyostelium purpureum]</td></tr>
<tr><td>Unigene68764_All</td><td>3.575054598</td><td>Up</td><td>NADH dehydrogenase 1 alpha subcomplex [Ornithodoros coriaceus]</td></tr>
</table>

Unigene51086_All	3.575051729	Up	NAD+ dependent glutamate dehydrogenase [Acanthamoeba castellanii str. Neff] >gi|440799441|gb|ELR20488.1| NAD+ dependent glutamate dehydrogenase [Acanthamoeba castellanii str. Neff]
CL3503.Contig3_All	3.564266059	Up	Uridine/cytidine kinase-like 1 [Camponotus floridanus]
CL5965.Contig1_All	3.563263407	Up	PREDICTED: similar to CG11255 CG11255-PB [Tribolium castaneum] >gi|270011602|gb|EFA08050.1| hypothetical protein TcasGA2_TC005644 [Tribolium castaneum]
Unigene56402_All	3.560773173	Up	L-lactate dehydrogenase [Daphnia magna]
CL3683.Contig2_All	3.543327931	Up	glyceraldehyde-3-phosphate dehydrogenase [Dasyatis sabina]
Unigene64164_All	3.519030357	Up	PREDICTED: similar to heparanase-like protein [Tribolium castaneum]
Unigene67205_All	3.518754526	Up	Chain B, Orientation Of Rna Polymerase Ii Within The Human Vp16-Mediator-Pol Ii-Tfiif Assembly
Unigene52713_All	3.51070105	Up	PREDICTED: aminopeptidase N-like [Megachile rotundata]
Unigene62498_All	3.499481123	Up	PREDICTED: gamma-glutamyltransferase 7-like [Takifugu rubripes]
Unigene55862_All	3.493209599	Up	PREDICTED: uncharacterized protein LOC100893421 [Strongylocentrotus purpuratus]
Unigene42196_All	3.489688318	Up	--
Unigene59035_All	3.479879147	Up	medium-chain specific acyl-CoA dehydrogenase [Salpingoeca sp. ATCC 50818]
CL1180.Contig2_All	3.478280992	Up	AGAP004159-PB [Anopheles gambiae str. PEST] >gi|333468634|gb|EGK97005.1| AGAP004159-PB [Anopheles gambiae str. PEST]
Unigene41984_All	3.470336374	Up	hypothetical protein TRIADDRAFT_60142 [Trichoplax adhaerens] >gi|190581109|gb|EDV21187.1| hypothetical protein TRIADDRAFT_60142 [Trichoplax adhaerens]
Unigene68145_All	3.460067763	Up	putative vacuolar ATP synthase subunit d 1 [Triops longicaudatus]
Unigene57393_All	3.460023668	Up	NADH:ubiquinone oxidoreductase 24 kDa subunit [Acanthamoeba castellanii str. Neff] >gi|440804510|gb|ELR25387.1| NADH:ubiquinone oxidoreductase 24 kDa subunit [Acanthamoeba castellanii str. Neff]
Unigene62754_All	3.459620408	Up	PREDICTED: uncharacterized short-chain type dehydrogenase/reductase y4vI-like [Xenopus (Silurana) tropicalis]
Unigene21237_All	3.45923556	Up	PREDICTED: similar to juvenile hormone esterase [Tribolium castaneum] >gi|270008042|gb|EFA04490.1| hypothetical protein TcasGA2_TC014795 [Tribolium castaneum]

CL8680.Contig2_All	3.419554533	Up	PREDICTED: carbonyl reductase [NADPH] 1-like [Bombus impatiens]
CL1082.Contig1_All	3.41949087	Up	Acyl-CoA dehydrogenase, putative [Perkinsus marinus ATCC 50983] >gi|239886243|gb|EER09917.1| Acyl-CoA dehydrogenase, putative [Perkinsus marinus ATCC 50983]
CL1036.Contig2_All	3.398509459	Up	PREDICTED: cytochrome b-c1 complex subunit 6, mitochondrial-like [Strongylocentrotus purpuratus]
CL9266.Contig2_All	3.397944409	Up	isoprenyl diphosphate synthase [Rhopalosiphum padi]
Unigene57207_All	3.395295815	Up	PREDICTED: aspartate aminotransferase, cytoplasmic-like [Takifugu rubripes]
CL7900.Contig2_All	3.385972628	Up	adenosylhomocysteinase B [Salpingoeca sp. ATCC 50818]
Unigene58442_All	3.385777863	Up	--
CL3163.Contig2_All	3.383537078	Up	PREDICTED: L-lactate dehydrogenase-like isoform 2 [Bombus impatiens]
Unigene58273_All	3.371343483	Up	glutathione S-transferase S4 [Apis cerana cerana] >gi|378406122|gb|AFB82729.1| glutathione S-transferase S4 [Apis cerana cerana]
Unigene43365_All	3.351625421	Up	PREDICTED: inosine-5'-monophosphate dehydrogenase-like isoform 1 [Megachile rotundata]
CL2716.Contig1_All	3.341133519	Up	PREDICTED: putative adenosylhomocysteinase 3-like isoform 1 [Bombus terrestris] >gi|350425225|ref|XP_003494052.1| PREDICTED: putative adenosylhomocysteinase 3-like [Bombus impatiens]
Unigene78848_All	3.334424395	Up	GDPmannose pyrophosphorylase [Acanthamoeba castellanii str. Neff] >gi|440804606|gb|ELR25483.1| GDPmannose pyrophosphorylase [Acanthamoeba castellanii str. Neff]
CL585.Contig1_All	3.334135097	Up	CAD [Polypogon strigilatus]
Unigene74498_All	3.334115275	Up	1,4-alpha-glucan branching enzyme [Homo sapiens]
Unigene55129_All	3.316829258	Up	PREDICTED: RNA-directed DNA polymerase from mobile element jockey-like [Strongylocentrotus purpuratus]
Unigene60310_All	3.312521587	Up	hypothetical protein BRAFLDRAFT_275316 [Branchiostoma floridae] >gi|229280821|gb|EEN51586.1| hypothetical protein BRAFLDRAFT_275316 [Branchiostoma floridae]
Unigene47590_All	3.29645134	Up	NADH dehydrogenase [ubiquinone] iron-sulfur protein 8, mitochondrial [Crassostrea gigas]
Unigene56381_All	3.280987007	Up	PREDICTED: 4-hydroxyphenylpyruvate dioxygenase-like [Strongylocentrotus purpuratus]
Unigene2604_All	3.257225141	Up	hypothetical protein TcasGA2_TC015613 [Tribolium castaneum]
Unigene42668_All	3.253477916	Up	branched-chain amino acid aminotransferase [Dictyostelium discoideum AX4] >gi|74853814|sp|Q54N47.1|BCAT_DICDI RecName: Full=Branched-chain-amino-acid

			aminotransferase >gi|60466540|gb|EAL64592.1| branched-chain amino acid aminotransferase [Dictyostelium discoideum AX4]
CL772.Contig1_All	3.246121687	Up	expressed hypothetical protein [Trichoplax adhaerens] >gi|190586853|gb|EDV26906.1| expressed hypothetical protein [Trichoplax adhaerens]
Unigene52371_All	3.237605626	Up	PREDICTED: polypeptide N-acetylgalactosaminyltransferase 3-like isoform 1 [Acyrthosiphon pisum] >gi|328723400|ref|XP_003247833.1| PREDICTED: polypeptide N-acetylgalactosaminyltransferase 3-like isoform 2 [Acyrthosiphon pisum]
Unigene64059_All	3.232446086	Up	PREDICTED: GPI-anchored wall transfer protein 1-like [Megachile rotundata]
Unigene52753_All	3.232302651	Up	Proline-rich protein 12 [Dicentrarchus labrax]
Unigene7280_All	3.232054154	Up	Beta-1,4-N-acetylgalactosaminyltransferase bre-4 [Camponotus floridanus]
Unigene59088_All	3.214924249	Up	Exostosin-3 [Harpegnathos saltator]
Unigene41068_All	3.21233047	Up	protein Y94A7B.2 [imported] - Caenorhabditis elegans
Unigene25735_All	3.202320068	Up	PREDICTED: similar to glucosyl/glucuronosyl transferases [Tribolium castaneum] >gi|270013465|gb|EFA09913.1| hypothetical protein TcasGA2_TC012064 [Tribolium castaneum]
CL5476.Contig1_All	3.200827036	Up	fatty acid beta-oxidation complex subunit beta [Heliothis virescens]
CL2657.Contig1_All	3.19701701	Up	GF18258 [Drosophila ananassae] >gi|190627466|gb|EDV42990.1| GF18258 [Drosophila ananassae]
Unigene71159_All	3.196759805	Up	Phospholipid methyltransferase [Acanthamoeba castellanii str. Neff] >gi|440791405|gb|ELR12643.1| Phospholipid methyltransferase [Acanthamoeba castellanii str. Neff]
Unigene59809_All	3.196735479	Up	PREDICTED: fatty aldehyde dehydrogenase-like [Ciona intestinalis]
Unigene20889_All	3.177492252	Up	AAEL010345-PA [Aedes aegypti]
CL3683.Contig3_All	3.167747748	Up	PREDICTED: glyceraldehyde-3-phosphate dehydrogenase-like isoform 1 [Strongylocentrotus purpuratus]
Unigene67695_All	3.167447771	Up	pyrroline-5-carboxylate reductase [Capsaspora owczarzaki ATCC 30864] >gi|320169301|gb|EFW46200.1| pyrroline-5-carboxylate reductase [Capsaspora owczarzaki ATCC 30864]
Unigene69447_All	3.160153169	Up	UDP-glucuronosyltransferase 2A3 [Camponotus floridanus]
CL1473.Contig3_All	3.152022716	Up	PREDICTED: pyruvate carboxylase, mitochondrial-like isoform 1 [Nasonia vitripennis] >gi|345495988|ref|XP_003427614.1| PREDICTED: pyruvate carboxylase, mitochondrial-like isoform 2 [Nasonia vitripennis]

Unigene71341_All	3.148034513	Up	NADP-dependent malic enzyme [Crassostrea gigas]
Unigene48916_All	3.147780643	Up	hypothetical protein [Monosiga brevicollis MX1] >gi|163776889|gb|EDQ90507.1| predicted protein [Monosiga brevicollis MX1]
Unigene59428_All	3.147708491	Up	hypothetical protein TcasGA2_TC001939 [Tribolium castaneum]
Unigene19144_All	3.144571605	Up	PREDICTED: beta-glucuronidase-like [Acyrthosiphon pisum]
CL3836.Contig2_All	3.133815361	Up	Long-chain-fatty-acid--CoA ligase, putative [Pediculus humanus corporis] >gi|212516281|gb|EEB18314.1| Long-chain-fatty-acid--CoA ligase, putative [Pediculus humanus corporis]
Unigene57161_All	3.133744123	Up	Cytochrome b-c1 complex subunit 9 [Acromyrmex echinatior]
CL290.Contig2_All	3.123224063	Up	Phosphatidylinositol transfer protein alpha isoform [Camponotus floridanus]
CL3470.Contig1_All	3.119771801	Up	PREDICTED: similar to AGAP004450-PA [Tribolium castaneum] >gi|270005322|gb|EFA01770.1| hypothetical protein TcasGA2_TC007369 [Tribolium castaneum]
Unigene57256_All	3.115918808	Up	PREDICTED: cytochrome c1, heme protein, mitochondrial-like [Anolis carolinensis]
Unigene51968_All	3.107475677	Up	putative Tc1-like transporase [Danaus plexippus]
Unigene55438_All	3.092901115	Up	PREDICTED: phosphatidylinositide phosphatase SAC1-like [Nasonia vitripennis]
Unigene54953_All	3.091703638	Up	PREDICTED: similar to reverse transcriptase homolog [Tribolium castaneum] >gi|270017183|gb|EFA13629.1| hypothetical protein TcasGA2_TC005274 [Tribolium castaneum]
Unigene46765_All	3.084521295	Up	pol-like protein [Biomphalaria glabrata]
CL3645.Contig3_All	3.081404002	Up	aminomethyltransferase,putative [Pediculus humanus corporis] >gi|212505419|gb|EEB09880.1| aminomethyltransferase,putative [Pediculus humanus corporis]
CL4029.Contig3_All	3.07485719	Up	PREDICTED: 4-hydroxyphenylpyruvate dioxygenase-like [Nasonia vitripennis]
Unigene53709_All	3.071224051	Up	PREDICTED: similar to glucosyl/glucuronosyl transferases [Tribolium castaneum] >gi|270013463|gb|EFA09911.1| hypothetical protein TcasGA2_TC012062 [Tribolium castaneum]
CL7198.Contig1_All	3.066878388	Up	PREDICTED: iron/zinc purple acid phosphatase-like protein-like [Acyrthosiphon pisum]
Unigene46172_All	3.044763105	Up	PREDICTED: stomatin-like protein 2-like [Anolis carolinensis]
Unigene46171_All	3.044564366	Up	Aminopeptidase N [Camponotus floridanus]

Unigene3293_All	3.035238846	Up	Pyrroline-5-carboxylate reductase, putative [Pediculus humanus corporis] >gi|212514649|gb|EEB16926.1| Pyrroline-5-carboxylate reductase, putative [Pediculus humanus corporis]
Unigene758_All	3.008820671	Up	PREDICTED: similar to sphingosine phosphate lyase isoform 1 [Tribolium castaneum] >gi|270001960|gb|EEZ98407.1| hypothetical protein TcasGA2_TC000875 [Tribolium castaneum]
CL1949.Contig3_All	3.0076399	Up	aconitase 2 [Capsaspora owczarzaki ATCC 30864] >gi|320169225|gb|EFW46124.1| aconitase 2 [Capsaspora owczarzaki ATCC 30864]
Unigene19518_All	3.00424219	Up	PREDICTED: similar to CG1673 CG1673-PA [Tribolium castaneum] >gi|270001557|gb|EEZ98004.1| hypothetical protein TcasGA2_TC000403 [Tribolium castaneum]
Unigene42487_All	3.004055725	Up	choline dehydrogenase [Clonorchis sinensis]
Unigene54972_All	3.002906186	Up	PREDICTED: uncharacterized protein LOC100882671 [Megachile rotundata]
CL8322.Contig2_All	2.996183488	Up	Dolichyl-diphosphooligosaccharide--protein glycosyltransferase subunit STT3A [Acromyrmex echinatior]
Unigene60374_All	2.990572054	Up	Succinyl-CoA ligase subunit alpha, mitochondrial precursor [Lepeophtheirus salmonis] >gi|290561819|gb|ADD38307.1| Succinyl-CoA ligase subunit alpha, mitochondrial [Lepeophtheirus salmonis]
CL1045.Contig1_All	2.990478622	Up	uncharacterized protein LOC100216301 [Xenopus (Silurana) tropicalis]
Unigene49049_All	2.990385843	Up	acetyl-CoA synthetase [Plasmodium falciparum 3D7] >gi|46361230|emb|CAG25091.1| acetyl-CoA synthetase [Plasmodium falciparum 3D7]
CL1485.Contig2_All	2.990339491	Up	uridine 5'-monophosphate synthase [Culex quinquefasciatus] >gi|167867356|gb|EDS30739.1| uridine 5'-monophosphate synthase [Culex quinquefasciatus]
Unigene68971_All	2.990317199	Up	hypothetical protein BRAFLDRAFT_123269 [Branchiostoma floridae] >gi|229293031|gb|EEN63692.1| hypothetical protein BRAFLDRAFT_123269 [Branchiostoma floridae]
CL5417.Contig2_All	2.987997367	Up	ATP synthase subunit delta [Capsaspora owczarzaki ATCC 30864] >gi|320169944|gb|EFW46843.1| ATP synthase subunit delta [Capsaspora owczarzaki ATCC 30864]
CL995.Contig1_All	2.985294144	Up	malate synthase [Polysphondylium pallidum PN500]
CL9135.Contig2_All	2.979327451	Up	predicted protein [Nematostella vectensis] >gi|156212302|gb|EDO33367.1| predicted protein [Nematostella vectensis]
Unigene49052_All	2.979225707	Up	PREDICTED: pyruvate dehydrogenase phosphatase regulatory subunit, mitochondrial-like [Megachile

<table>
<tr><td></td><td></td><td></td><td>rotundata]</td></tr>
<tr><td>Unigene59465_All</td><td>2.962475642</td><td>Up</td><td>NADH dehydrogenase, putative [Acanthamoeba castellanii str. Neff] >gi|470446485|ref|XP_004339965.1| NADH dehydrogenase, putative [Acanthamoeba castellanii str. Neff] >gi|440795825|gb|ELR16940.1| NADH dehydrogenase, putative [Acanthamoeba castellanii str. Neff] >gi|440796848|gb|ELR17949.1| NADH dehydrogenase, putative [Acanthamoeba castellanii str. Neff]</td></tr>
<tr><td>Unigene57090_All</td><td>2.962460067</td><td>Up</td><td>Ethanolaminephosphotransferase 1 [Camponotus floridanus]</td></tr>
<tr><td>Unigene53430_All</td><td>2.958738035</td><td>Up</td><td>conserved hypothetical protein [Pediculus humanus corporis] >gi|212516889|gb|EEB18843.1| conserved hypothetical protein [Pediculus humanus corporis]</td></tr>
<tr><td>CL1473.Contig2_All</td><td>2.95735219</td><td>Up</td><td>PREDICTED: pyruvate carboxylase, mitochondrial-like isoform 4 [Nasonia vitripennis]</td></tr>
<tr><td>Unigene50470_All</td><td>2.953574245</td><td>Up</td><td>Ethanolaminephosphotransferase 1 [Acromyrmex echinatior]</td></tr>
<tr><td>CL5387.Contig1_All</td><td>2.950050632</td><td>Up</td><td>unnamed protein product [Oikopleura dioica]</td></tr>
<tr><td>Unigene51969_All</td><td>2.933829498</td><td>Up</td><td>PREDICTED: similar to AGAP009609-PA [Tribolium castaneum] >gi|270011358|gb|EFA07806.1| hypothetical protein TcasGA2_TC005367 [Tribolium castaneum]</td></tr>
<tr><td>CL1259.Contig1_All</td><td>2.918545003</td><td>Up</td><td>Rieske iron-sulfur protein 1 [Capsaspora owczarzaki ATCC 30864] >gi|320168907|gb|EFW45806.1| Rieske iron-sulfur protein 1 [Capsaspora owczarzaki ATCC 30864]</td></tr>
<tr><td>Unigene54955_All</td><td>2.915333201</td><td>Up</td><td>PREDICTED: similar to reverse transcriptase homolog [Tribolium castaneum] >gi|270017183|gb|EFA13629.1| hypothetical protein TcasGA2_TC005274 [Tribolium castaneum]</td></tr>
<tr><td>Unigene40796_All</td><td>2.913184625</td><td>Up</td><td>catalase [Capsaspora owczarzaki ATCC 30864] >gi|320166530|gb|EFW43429.1| catalase [Capsaspora owczarzaki ATCC 30864]</td></tr>
<tr><td>Unigene55334_All</td><td>2.913132228</td><td>Up</td><td>UDP-glucose pyrophosphorylase [Locusta migratoria]</td></tr>
<tr><td>Unigene58490_All</td><td>2.910660502</td><td>Up</td><td>Medium-chain specific acyl-CoA dehydrogenase, putative [Perkinsus marinus ATCC 50983] >gi|239902533|gb|EER19323.1| Medium-chain specific acyl-CoA dehydrogenase, putative [Perkinsus marinus ATCC 50983]</td></tr>
<tr><td>Unigene57181_All</td><td>2.906364672</td><td>Up</td><td>PREDICTED: dol-P-Glc:Glc(2)Man(9)GlcNAc(2)-PP-Dol alpha-1,2-glucosyltransferase-like [Hydra magnipapillata]</td></tr>
<tr><td>Unigene60118_All</td><td>2.90464022</td><td>Up</td><td>vacuolar ATP synthase subunit G [Capsaspora owczarzaki ATCC 30864] >gi|320169461|gb|EFW46360.1| vacuolar ATP synthase subunit G [Capsaspora owczarzaki ATCC 30864]</td></tr>
</table>

Unigene13382_All	2.896864225	Up	hypothetical protein DAPPUDRAFT_40255 [Daphnia pulex]
Unigene6815_All	2.895739009	Up	PREDICTED: cytoplasmic aconitate hydratase [Megachile rotundata]
Unigene41979_All	2.886847762	Up	PREDICTED: NADH-ubiquinone oxidoreductase 75 kDa subunit, mitochondrial isoform 2 [Monodelphis domestica]
Unigene54318_All	2.88617363	Up	PREDICTED: pecanex-like protein 1-like [Nasonia vitripennis]
Unigene65613_All	2.875198613	Up	biotin synthase [Capsaspora owczarzaki ATCC 30864] >gi|320166303|gb|EFW43202.1| biotin synthase [Capsaspora owczarzaki ATCC 30864]
Unigene55307_All	2.875009635	Up	PREDICTED: LOW QUALITY PROTEIN: aminopeptidase N-like [Apis florea]
CL3206.Contig2_All	2.874957422	Up	triose-phosphate isomerase [Schistosoma turkestanicum]
Unigene48752_All	2.87492323	Up	malate dehydrogenase [Capsaspora owczarzaki ATCC 30864] >gi|320165329|gb|EFW42228.1| malate dehydrogenase [Capsaspora owczarzaki ATCC 30864]
Unigene41945_All	2.873942298	Up	PREDICTED: ethanolamine-phosphate cytidylyltransferase-like [Nasonia vitripennis]
Unigene52998_All	2.855760562	Up	AAEL017443-PA [Aedes aegypti]
CL1949.Contig1_All	2.840737569	Up	hypothetical protein [Monosiga brevicollis MX1] >gi|163774116|gb|EDQ87748.1| predicted protein [Monosiga brevicollis MX1]
Unigene43500_All	2.817877729	Up	PREDICTED: isocitrate dehydrogenase 3 (NAD+) gamma-like [Saccoglossus kowalevskii]
Unigene55765_All	2.8035297	Up	hypothetical protein TcasGA2_TC014935 [Tribolium castaneum]
Unigene62169_All	2.797867544	Up	PREDICTED: similar to reverse transcriptase homolog [Tribolium castaneum] >gi|270017183|gb|EFA13629.1| hypothetical protein TcasGA2_TC005274 [Tribolium castaneum]
CL1036.Contig1_All	2.796950096	Up	hypothetical protein [Monosiga brevicollis MX1] >gi|163775514|gb|EDQ89138.1| predicted protein [Monosiga brevicollis MX1]
Unigene53474_All	2.784501711	Up	PREDICTED: polypeptide N-acetylgalactosaminyltransferase 2-like [Megachile rotundata]
Unigene58566_All	2.781960294	Up	PREDICTED: allantoicase-like [Takifugu rubripes]
CL1659.Contig1_All	2.78186596	Up	Aldose reductase [Osmerus mordax]
Unigene62636_All	2.781783621	Up	PREDICTED: exostosin-3-like isoform 2 [Nasonia vitripennis] >gi|345494727|ref|XP_001604616.2| PREDICTED: exostosin-3-like isoform 1 [Nasonia vitripennis]
CL9795.Contig2_All	2.778435524	Up	PREDICTED: putative glutamate synthase [NADPH]-like [Acyrthosiphon pisum]

Unigene8988_All	2.77522735	Up	Protein diaphanous [Harpegnathos saltator]
Unigene45659_All	2.762428712	Up	Synaptojanin-1 [Camponotus floridanus]
Unigene64194_All	2.75779343	Up	PREDICTED: similar to CG3194 CG3194-PA [Tribolium castaneum] >gi|270013933|gb|EFA10381.1| hypothetical protein TcasGA2_TC012612 [Tribolium castaneum]
Unigene13304_All	2.757770314	Up	beta-N-acetylglucosaminidase NAG2 precursor [Tribolium castaneum] >gi|148611478|gb|ABQ95983.1| beta-N-acetylglucosaminidase NAG2 [Tribolium castaneum]
Unigene51970_All	2.749304809	Up	GF14763 [Drosophila ananassae] >gi|190615466|gb|EDV30990.1| GF14763 [Drosophila ananassae]
Unigene46254_All	2.740249613	Up	NAD-dependent malic enzyme, putative [Acanthamoeba castellanii str. Neff] >gi|440804624|gb|ELR25501.1| NAD-dependent malic enzyme, putative [Acanthamoeba castellanii str. Neff]
CL3796.Contig2_All	2.735435278	Up	hypothetical protein TcasGA2_TC005493 [Tribolium castaneum]
Unigene54440_All	2.733120857	Up	Transposable element Tcb1 transposase [Salmo salar]
Unigene49520_All	2.730837649	Up	carmil, putative [Pediculus humanus corporis] >gi|212516589|gb|EEB18583.1| carmil, putative [Pediculus humanus corporis]
Unigene48400_All	2.725809181	Up	predicted protein [Nematostella vectensis] >gi|156225910|gb|EDO46724.1| predicted protein [Nematostella vectensis]
Unigene56772_All	2.711259391	Up	malate dehydrogenase, putative [Ixodes scapularis] >gi|215496276|gb|EEC05916.1| malate dehydrogenase, putative [Ixodes scapularis]
Unigene2807_All	2.70719838	Up	PREDICTED: glucose dehydrogenase [acceptor]-like [Nasonia vitripennis]
Unigene10455_All	2.683004201	Up	PREDICTED: similar to conserved hypothetical protein [Tribolium castaneum] >gi|270003721|gb|EFA00169.1| hypothetical protein TcasGA2_TC002991 [Tribolium castaneum]
Unigene11579_All	2.682274536	Up	PREDICTED: dolichyl pyrophosphate Man9GlcNAc2 alpha-1,3-glucosyltransferase [Taeniopygia guttata]
Unigene58399_All	2.682233346	Up	TPA_inf: vacuolar H+-ATPase V0 sector subunits c/c' [Amblyomma variegatum]
Unigene54074_All	2.682232466	Up	Lysocardiolipin acyltransferase 1 [Acromyrmex echinatior]
Unigene57176_All	2.661611054	Up	predicted protein [Nematostella vectensis] >gi|156226759|gb|EDO47567.1| predicted protein [Nematostella vectensis]

Unigene51224_All	2.649456948	Up	PREDICTED: glycogen debranching enzyme [Megachile rotundata]
Unigene54598_All	2.647630472	Up	PREDICTED: similar to alpha-(1,6)-fucosyltransferase [Tribolium castaneum] >gi|270006336|gb|EFA02784.1| hypothetical protein TcasGA2_TC008521 [Tribolium castaneum]
Unigene55508_All	2.647625663	Up	GI23350 [Drosophila mojavensis] >gi|193915529|gb|EDW14396.1| GI23350 [Drosophila mojavensis]
Unigene13135_All	2.642505338	Up	PREDICTED: inositol-tetrakisphosphate 1-kinase-like [Acyrthosiphon pisum]
Unigene15527_All	2.63573105	Up	3-hydroxy-3-methylglutaryl coenzyme A reductase [Nasutitermes takasagoensis]
CL1310.Contig7_All	2.630078274	Up	mitochondrial ATP synthase subunit beta precursor [Litopenaeus vannamei]
CL432.Contig1_All	2.617190391	Up	PREDICTED: V-type proton ATPase 116 kDa subunit a isoform 1-like isoform 2 [Bombus impatiens]
CL8910.Contig2_All	2.616044892	Up	PREDICTED: glucosamine--fructose-6-phosphate aminotransferase [isomerizing] 2-like [Apis florea]
Unigene78998_All	2.612242085	Up	2-oxoglutarate dehydrogenase, E1 subunit [Dictyostelium discoideum AX4] >gi|74852748|sp|Q54JE4.1|ODO1_DICDI RecName: Full=2-oxoglutarate dehydrogenase, mitochondrial; AltName: Full=2-oxoglutarate dehydrogenase complex component E1; Short=OGDC-E1; AltName: Full=Alpha-ketoglutarate dehydrogenase; Flags: Precursor >gi|60465316|gb|EAL63408.1| 2-oxoglutarate dehydrogenase, E1 subunit [Dictyostelium discoideum AX4]
Unigene61551_All	2.612001141	Up	PREDICTED: rhomboid-related protein 3-like [Strongylocentrotus purpuratus]
Unigene74278_All	2.611686619	Up	predicted protein [Nematostella vectensis] >gi|156227695|gb|EDO48497.1| predicted protein [Nematostella vectensis]
Unigene20843_All	2.611642998	Up	PREDICTED: probable beta-hexosaminidase fdl-like [Nasonia vitripennis]
Unigene19669_All	2.597485894	Up	hypothetical protein AND_16858 [Anopheles darlingi]
Unigene57751_All	2.587677075	Up	RecName: Full=5-methyltetrahydropteroyltriglutamate--homocysteine methyltransferase; AltName: Full=Cobalamin-independent methionine synthase; AltName: Full=Methionine synthase, vitamin-B12 independent isozyme
Unigene66783_All	2.585679843	Up	PREDICTED: protein retinal degeneration B-like [Bombus impatiens]
Unigene59599_All	2.575408713	Up	DNA polymerase epsilon catalytic subunit A [Heterocephalus glaber]
Unigene58509_All	2.575386589	Up	ethanolamine-phosphate cytidylyltransferase [Rattus norvegicus] >gi|30580471|sp|O88637.1|PCY2_RAT RecName: Full=Ethanolamine-phosphate cytidylyltransferase; AltName: Full=CTP:phosphoethanolamine cytidylyltransferase; AltName: Full=Phosphorylethanolamine transferase >gi|3396102|gb|AAC28864.1|

			CTP:phosphoethanolamine cytidylyltransferase [Rattus norvegicus] >gi|149055051|gb|EDM06868.1| phosphate cytidylyltransferase 2, ethanolamine, isoform CRA_a [Rattus norvegicus]
Unigene51928_All	2.559268764	Up	hypothetical protein TcasGA2_TC001824 [Tribolium castaneum]
Unigene48611_All	2.556750323	Up	PREDICTED: ATP synthase subunit beta, mitochondrial-like [Apis florea]
Unigene67560_All	2.553096476	Up	nitric oxide synthase [Gryllus bimaculatus]
Unigene47832_All	2.553046484	Up	hypothetical protein TcasGA2_TC009600 [Tribolium castaneum]
CL3900.Contig1_All	2.538022199	Up	Spermidine synthase [Crassostrea gigas]
Unigene11648_All	2.53267069	Up	PREDICTED: alpha-1,3-mannosyl-glycoprotein 2-beta-N-acetylglucosaminyltransferase-like [Megachile rotundata]
CL416.Contig1_All	2.513918595	Up	PREDICTED: similar to acid alpha-glucosidase [Tribolium castaneum]
Unigene57782_All	2.512484717	Up	Spermidine synthase [Crassostrea gigas]
Unigene17403_All	2.507786092	Up	PREDICTED: inositol-3-phosphate synthase 1-B-like [Megachile rotundata]
CL6316.Contig1_All	2.507363899	Up	mannose-1-phosphate guanyltransferase beta [Capsaspora owczarzaki ATCC 30864] >gi|320169204|gb|EFW46103.1| mannose-1-phosphate guanyltransferase beta [Capsaspora owczarzaki ATCC 30864]
Unigene63350_All	2.507110589	Up	PREDICTED: ribose-phosphate pyrophosphokinase 1 isoform 2 [Gallus gallus] >gi|224098594|ref|XP_002187809.1| PREDICTED: ribose-phosphate pyrophosphokinase 1 isoform 1 [Taeniopygia guttata]
Unigene3692_All	2.506376882	Up	PREDICTED: similar to protein-O-xylosyltransferase [Tribolium castaneum] >gi|270016288|gb|EFA12734.1| hypothetical protein TcasGA2_TC002371 [Tribolium castaneum]
CL4227.Contig1_All	2.503236681	Up	PREDICTED: similar to Mgat2 CG7921-PB [Tribolium castaneum]
Unigene49050_All	2.503000929	Up	acetyl-CoA synthetase [Polysphondylium pallidum PN500]
Unigene47410_All	2.495923953	Up	PREDICTED: type I inositol-3,4-bisphosphate 4-phosphatase-like [Apis florea]
Unigene57277_All	2.492328476	Up	juvenile hormone esterase-like protein Est1 [Reticulitermes flavipes]
Unigene5865_All	2.490601704	Up	senescence marker protein-30, putative [Pediculus humanus corporis] >gi|212517874|gb|EEB19703.1| senescence marker protein-30, putative [Pediculus humanus corporis]
Unigene12177_All	2.483590432	Up	4-aminobutyrate aminotransferase [Aedes aegypti] >gi|108870984|gb|EAT35209.1| AAEL012609-PA

			[Aedes aegypti]
CL3685.Contig1_All	2.482538548	Up	PREDICTED: putative polypeptide N-acetylgalactosaminyltransferase 9-like isoform 1 [Bombus impatiens]
Unigene20009_All	2.473151521	Up	NADP-dependent malic enzyme-like protein [Locusta migratoria]
Unigene17890_All	2.470948721	Up	glutathione S-transferase sigma 6 [Locusta migratoria]
CL10030.Contig3_All	2.468314481	Up	pol-like protein [Biomphalaria glabrata]
CL8797.Contig1_All	2.467708721	Up	transposase [Salmo salar]
Unigene43230_All	2.464730253	Up	PREDICTED: RNA-directed DNA polymerase from mobile element jockey-like [Strongylocentrotus purpuratus]
Unigene55886_All	2.459925656	Up	Tetratricopeptide repeat protein 27 [Camponotus floridanus]
Unigene65992_All	2.459837032	Up	PREDICTED: NADH dehydrogenase [ubiquinone] 1 beta subcomplex subunit 8, mitochondrial-like [Hydra magnipapillata]
Unigene42319_All	2.459737103	Up	Aldose reductase [Giardia lamblia P15]
Unigene20913_All	2.452010977	Up	PREDICTED: beta-1,3-galactosyltransferase 1-like isoform 1 [Bombus terrestris]
CL4259.Contig1_All	2.446482324	Up	PREDICTED: nucleoside diphosphate kinase-like [Anolis carolinensis]
CL6466.Contig1_All	2.445347203	Up	fructose-1,6-bisphosphatase family protein [Tetrahymena thermophila] >gi|89289359|gb|EAR87347.1| fructose-1,6-bisphosphatase family protein [Tetrahymena thermophila SB210]
Unigene58687_All	2.442503412	Up	predicted protein [Nematostella vectensis] >gi|156228774|gb|EDO49572.1| predicted protein [Nematostella vectensis]
Unigene50870_All	2.4397898	Up	carboxyl/choline esterase CCE014a [Helicoverpa armigera]
CL8559.Contig1_All	2.431810576	Up	PREDICTED: hypothetical protein LOC100679013 isoform 1 [Nasonia vitripennis]
Unigene57208_All	2.417443431	Up	Cytochrome c oxidase subunit 5B, mitochondrial [Myotis davidii]
Unigene58434_All	2.412997585	Up	PREDICTED: beta-chimaerin-like [Acyrthosiphon pisum]
Unigene44450_All	2.411211954	Up	aspartate aminotransferase, cytoplasmic [Rattus norvegicus] >gi|122065118|sp|P13221.3|AATC_RAT RecName: Full=Aspartate aminotransferase, cytoplasmic; AltName: Full=Glutamate oxaloacetate transaminase 1; AltName: Full=Transaminase A >gi|345752|pir||S29028 aspartate transaminase (EC 2.6.1.1) (clone 8C7) - human >gi|38197390|gb|AAH61877.1| Glutamic-oxaloacetic transaminase 1, soluble (aspartate aminotransferase 1) [Rattus norvegicus] >gi|149040215|gb|EDL94253.1| glutamate

<table>
<tr><td></td><td></td><td></td><td>oxaloacetate transaminase 1, isoform CRA_a [Rattus norvegicus] >gi|361035|prf||1406303A cytosolic Asp aminotransferase</td></tr>
<tr><td>Unigene55254_All</td><td>2.405968696</td><td>Up</td><td>PREDICTED: type I inositol-3,4-bisphosphate 4-phosphatase-like [Megachile rotundata]</td></tr>
<tr><td>Unigene47114_All</td><td>2.401535953</td><td>Up</td><td>PREDICTED: glycerate kinase-like [Oryzias latipes]</td></tr>
<tr><td>CL9135.Contig1_All</td><td>2.389488608</td><td>Up</td><td>carbamylphosphate synthetase 1 [Salpingoeca sp. ATCC 50818]</td></tr>
<tr><td>CL5482.Contig2_All</td><td>2.364410044</td><td>Up</td><td>GMP synthase, putative [Pediculus humanus corporis] >gi|212512030|gb|EEB14877.1| GMP synthase, putative [Pediculus humanus corporis]</td></tr>
<tr><td>Unigene46591_All</td><td>2.364312566</td><td>Up</td><td>synaptojanin-1, putative [Pediculus humanus corporis] >gi|212514961|gb|EEB17177.1| synaptojanin-1, putative [Pediculus humanus corporis]</td></tr>
<tr><td>Unigene14050_All</td><td>2.344002895</td><td>Up</td><td>Galactoside 2-alpha-L-fucosyltransferase 3 [Camponotus floridanus]</td></tr>
<tr><td>Unigene59309_All</td><td>2.334382985</td><td>Up</td><td>triacylglycerol lipase, pancreatic, putative [Pediculus humanus corporis] >gi|212509146|gb|EEB12623.1| triacylglycerol lipase, pancreatic, putative [Pediculus humanus corporis]</td></tr>
<tr><td>CL6538.Contig1_All</td><td>2.334344142</td><td>Up</td><td>putative 5'-nucleotidase [Chorthippus parallelus]</td></tr>
<tr><td>Unigene16848_All</td><td>2.328464543</td><td>Up</td><td>CBN-SODH-1 protein [Caenorhabditis brenneri]</td></tr>
<tr><td>Unigene62049_All</td><td>2.317862015</td><td>Up</td><td>PREDICTED: similar to reverse transcriptase homolog [Tribolium castaneum] >gi|270017183|gb|EFA13629.1| hypothetical protein TcasGA2_TC005274 [Tribolium castaneum]</td></tr>
<tr><td>Unigene15520_All</td><td>2.31456285</td><td>Up</td><td>3-hydroxy-3-methylglutaryl coenzyme A synthase [Nasutitermes takasagoensis]</td></tr>
<tr><td>Unigene43713_All</td><td>2.308415768</td><td>Up</td><td>hypothetical protein TcasGA2_TC009196 [Tribolium castaneum]</td></tr>
<tr><td>Unigene15638_All</td><td>2.305572465</td><td>Up</td><td>mevalonate kinase [Dendroctonus ponderosae]</td></tr>
<tr><td>CL1529.Contig2_All</td><td>2.300994715</td><td>Up</td><td>Annexin-B11 [Harpegnathos saltator]</td></tr>
<tr><td>Unigene46624_All</td><td>2.290091394</td><td>Up</td><td>PREDICTED: tripeptidyl-peptidase 2-like [Bombus impatiens]</td></tr>
<tr><td>Unigene58087_All</td><td>2.289957558</td><td>Up</td><td>PREDICTED: putative alpha-1,2-glucosyltransferase ALG10-B-like [Bombus terrestris]</td></tr>
<tr><td>Unigene62002_All</td><td>2.289891288</td><td>Up</td><td>Pyridoxal phosphate phosphatase, putative [Pediculus humanus corporis] >gi|212506991|gb|EEB11037.1| Pyridoxal phosphate phosphatase, putative [Pediculus humanus corporis]</td></tr>
<tr><td>Unigene63086_All</td><td>2.289891288</td><td>Up</td><td>PREDICTED: S-methyl-5'-thioadenosine phosphorylase [Felis catus]</td></tr>
<tr><td>Unigene56796_All</td><td>2.273418424</td><td>Up</td><td>PREDICTED: hypothetical protein LOC100162931 [Acyrthosiphon pisum]</td></tr>
<tr><td>Unigene13221_All</td><td>2.27302263</td><td>Up</td><td>GI23457 [Drosophila mojavensis] >gi|193915344|gb|EDW14211.1| GI23457 [Drosophila mojavensis]</td></tr>
</table>

Unigene64232_All	2.253493977	Up	juvenile hormone esterase-like protein Est1 [Reticulitermes flavipes]
Unigene47246_All	2.253447034	Up	PREDICTED: LOW QUALITY PROTEIN: glycogen debranching enzyme-like [Apis florea]
Unigene47571_All	2.24593728	Up	PREDICTED: phosphatidylinositol-4-phosphate 3-kinase C2 domain-containing subunit beta-like [Megachile rotundata]
Unigene20881_All	2.244623362	Up	PREDICTED: luciferin 4-monooxygenase-like [Megachile rotundata]
Unigene59731_All	2.244284471	Up	unnamed protein product [Oikopleura dioica] >gi|313241230|emb|CBY33512.1| unnamed protein product [Oikopleura dioica]
Unigene64330_All	2.244226316	Up	GA10135 [Drosophila pseudoobscura pseudoobscura] >gi|198138284|gb|EAL33646.2| GA10135 [Drosophila pseudoobscura pseudoobscura]
CL1542.Contig1_All	2.240027841	Up	unnamed protein product, partial [Leishmania mexicana MHOM/GT/2001/U1103] >gi|356491245|emb|CBZ41040.1| unnamed protein product, partial [Leishmania mexicana MHOM/GT/2001/U1103]
Unigene53629_All	2.239594943	Up	PREDICTED: similar to AGAP005012-PA [Tribolium castaneum] >gi|270004305|gb|EFA00753.1| hypothetical protein TcasGA2_TC003637 [Tribolium castaneum]
Unigene14275_All	2.239496675	Up	PREDICTED: glycoprotein-N-acetylgalactosamine 3-beta-galactosyltransferase 1-like isoform 1 [Bombus terrestris] >gi|340717982|ref|XP_003397452.1| PREDICTED: glycoprotein-N-acetylgalactosamine 3-beta-galactosyltransferase 1-like isoform 2 [Bombus terrestris]
CL9789.Contig1_All	2.237887408	Up	hypothetical protein DICPUDRAFT_34436 [Dictyostelium purpureum] >gi|325081249|gb|EGC34771.1| hypothetical protein DICPUDRAFT_34436 [Dictyostelium purpureum]
CL5218.Contig1_All	2.237537858	Up	hypothetical protein TcasGA2_TC002488 [Tribolium castaneum]
Unigene5204_All	2.230369561	Up	Platelet-activating factor acetylhydrolase precursor, putative [Pediculus humanus corporis] >gi|212512001|gb|EEB14848.1| Platelet-activating factor acetylhydrolase precursor, putative [Pediculus humanus corporis]
Unigene20094_All	2.217025172	Up	hypothetical protein DAPPUDRAFT_302347 [Daphnia pulex]
Unigene60327_All	2.196963642	Up	hypothetical protein TcasGA2_TC009600 [Tribolium castaneum]
Unigene52176_All	2.196922364	Up	NAD+ dependent glutamate dehydrogenase [Dictyostelium discoideum AX4] >gi|263432342|sp|Q54VI3.2|GLUD2_DICDI RecName: Full=Glutamate dehydrogenase 2; AltName: Full=NAD-specific glutamate dehydrogenase;

			Short=NAD-GDH >gi|165988616|gb|EAL67386.2| NAD+ dependent glutamate dehydrogenase [Dictyostelium discoideum AX4]
Unigene51894_All	2.196919889	Up	Protein stoned-B [Camponotus floridanus]
Unigene64185_All	2.196790518	Up	dihydrolipoyl dehydrogenase [Capsaspora owczarzaki ATCC 30864] >gi|320165064|gb|EFW41963.1| dihydrolipoyl dehydrogenase [Capsaspora owczarzaki ATCC 30864]
Unigene50355_All	2.196782383	Up	nitric oxide synthase [Gryllus bimaculatus]
Unigene56452_All	2.191938722	Up	hypothetical protein SINV_06469 [Solenopsis invicta]
Unigene11000_All	2.186528471	Up	hypothetical protein AND_07861 [Anopheles darlingi]
Unigene22105_All	2.17612041	Up	hypothetical protein TcasGA2_TC007649 [Tribolium castaneum]
CL9576.Contig2_All	2.170940902	Up	2-oxo-4-hydroxy-4-carboxy-5-ureidoimidazoline decarboxylase [Crassostrea gigas]
Unigene60220_All	2.162527642	Up	AAEL010345-PA [Aedes aegypti]
Unigene6342_All	2.161797381	Up	hypothetical protein AND_05215 [Anopheles darlingi]
CL9405.Contig3_All	2.161027719	Up	PREDICTED: RNA-directed DNA polymerase from mobile element jockey-like [Strongylocentrotus purpuratus]
Unigene22611_All	2.160838354	Up	isocitrate dehydrogenase [Papilio xuthus]
Unigene48053_All	2.155120719	Up	hypothetical protein IMG5_127390 [Ichthyophthirius multifiliis] >gi|340504160|gb|EGR30635.1| hypothetical protein IMG5_127390 [Ichthyophthirius multifiliis]
Unigene12928_All	2.15126951	Up	PREDICTED: hypothetical protein LOC100119058 [Nasonia vitripennis]
CL2564.Contig1_All	2.146718752	Up	PREDICTED: similar to 5-aminoimidazole-4-carboxamide ribonucleotide formyltransferase/IMP cyclohydrolase [Tribolium castaneum] >gi|270002383|gb|EEZ98830.1| hypothetical protein TcasGA2_TC004437 [Tribolium castaneum]
CL6507.Contig1_All	2.132328606	Up	PREDICTED: hydroxysteroid dehydrogenase-like protein 2-like [Nasonia vitripennis]
CL532.Contig1_All	2.131281693	Up	peroxiredoxin, partial [Schistocerca gregaria]
Unigene59669_All	2.131210771	Up	Ubiquinone biosynthesis protein COQ7 homolog [Caligus rogercresseyi]
Unigene5314_All	2.130563268	Up	PREDICTED: guanine deaminase-like [Bombus impatiens]
CL5150.Contig1_All	2.127270868	Up	Probable peroxisomal acyl-coenzyme A oxidase 1 [Camponotus floridanus]
Unigene9577_All	2.124508077	Up	cytochrome c oxidase subunit 2 [Monosiga brevicollis ATCC 50154] >gi|23344078|gb|AAN28354.1| cytochrome c oxidase subunit 2 (mitochondrion) [Monosiga brevicollis]

Unigene57761_All	2.112095772	Up	PREDICTED: NADH dehydrogenase [ubiquinone] flavoprotein 2, mitochondrial isoform 2 [Gallus gallus]
Unigene47638_All	2.101237851	Up	PREDICTED: putative neutral sphingomyelinase-like [Bombus impatiens]
CL7016.Contig1_All	2.093411153	Up	--
Unigene207_All	2.092856168	Up	PREDICTED: aromatic-L-amino-acid decarboxylase-like isoform 1 [Bombus impatiens]
Unigene22022_All	2.084082004	Up	GK22419 [Drosophila willistoni] >gi|313471317|sp|B4NG41.1|MTNA_DROWI RecName: Full=Methylthioribose-1-phosphate isomerase; Short=M1Pi; Short=MTR-1-P isomerase; AltName: Full=S-methyl-5-thioribose-1-phosphate isomerase; AltName: Full=Translation initiation factor eIF-2B subunit alpha/beta/delta-like protein >gi|194168357|gb|EDW83258.1| GK22419 [Drosophila willistoni]
Unigene18107_All	2.074534104	Up	PREDICTED: similar to CG11190 CG11190-PA [Tribolium castaneum] >gi|270006777|gb|EFA03225.1| hypothetical protein TcasGA2_TC013150 [Tribolium castaneum]
CL4454.Contig1_All	2.070686895	Up	Fumarylacetoacetate hydrolase domain-containing protein, putative [Pediculus humanus corporis] >gi|212515086|gb|EEB17286.1| Fumarylacetoacetate hydrolase domain-containing protein, putative [Pediculus humanus corporis]
CL2488.Contig3_All	2.067610346	Up	Chain A, Structure Of Trypanosoma Brucei Enolase Reveals The Inhibitory Divalent Metal Site
CL2564.Contig2_All	2.066131897	Up	PREDICTED: similar to 5-aminoimidazole-4-carboxamide ribonucleotide formyltransferase/IMP cyclohydrolase [Tribolium castaneum] >gi|270002383|gb|EEZ98830.1| hypothetical protein TcasGA2_TC004437 [Tribolium castaneum]
Unigene5355_All	2.062207082	Up	juvenile hormone esterase-like protein Est1 [Reticulitermes flavipes]
Unigene59131_All	2.059310881	Up	PREDICTED: xylulose kinase-like [Megachile rotundata]
CL3443.Contig2_All	2.058833866	Up	PREDICTED: CAD protein-like [Megachile rotundata]
Unigene23308_All	2.056255039	Up	ATP-citrate synthase [Aedes aegypti] >gi|108880117|gb|EAT44342.1| AAEL004297-PA [Aedes aegypti]
Unigene55112_All	2.05565667	Up	PREDICTED: RNA-directed DNA polymerase from mobile element jockey-like [Strongylocentrotus purpuratus]
Unigene23476_All	2.053793108	Up	PREDICTED: probable aspartate aminotransferase, cytoplasmic-like [Megachile rotundata]
Unigene16753_All	2.051877245	Up	PREDICTED: 5'-nucleotidase domain-containing protein 1-like [Megachile rotundata]
Unigene12957_All	2.050731866	Up	tdrd7 [Gryllus bimaculatus]
CL5508.Contig3_All	2.048196788	Up	putative DNA-mediated transposase [Helicoverpa zea]

CL3645.Contig4_All	2.04660664	Up	aminomethyltransferase,putative [Pediculus humanus corporis] >gi|212505419|gb|EEB09880.1| aminomethyltransferase,putative [Pediculus humanus corporis]
CL2784.Contig6_All	2.046028173	Up	Choline/ethanolamine kinase [Harpegnathos saltator]
Unigene49483_All	2.044920097	Up	CG33791, isoform E [Drosophila melanogaster] >gi|281365454|ref|NP_001163322.1| CG33791, isoform F [Drosophila melanogaster] >gi|272455003|gb|ACZ94593.1| CG33791, isoform E [Drosophila melanogaster] >gi|272455004|gb|ACZ94594.1| CG33791, isoform F [Drosophila melanogaster]
Unigene69299_All	2.044822664	Up	C4methyl sterol oxidase [Acanthamoeba castellanii str. Neff] >gi|440796037|gb|ELR17146.1| C4methyl sterol oxidase [Acanthamoeba castellanii str. Neff]
CL1922.Contig4_All	2.034797232	Up	AGAP000849-PA [Anopheles gambiae str. PEST] >gi|116126160|gb|EAL39756.2| AGAP000849-PA [Anopheles gambiae str. PEST]
Unigene60101_All	2.024724414	Up	arginase [Leishmania braziliensis MHOM/BR/75/M2904] >gi|134065587|emb|CAM43357.1| arginase [Leishmania braziliensis MHOM/BR/75/M2904]
Unigene13279_All	2.023452285	Up	purine nucleoside phosphorylase [Papilio xuthus]
CL5673.Contig2_All	2.021764945	Up	predicted protein [Nematostella vectensis] >gi|156222143|gb|EDO42990.1| predicted protein [Nematostella vectensis]
CL3685.Contig2_All	2.018821888	Up	PREDICTED: putative polypeptide N-acetylgalactosaminyltransferase 9-like isoform 1 [Bombus impatiens]
CL1522.Contig3_All	2.001604693	Up	PREDICTED: short/branched chain specific acyl-CoA dehydrogenase, mitochondrial-like [Megachile rotundata]
Unigene2335_All	2.00148882	Up	Probable glutamine-dependent NAD(+) synthetase [Harpegnathos saltator]
Unigene61185_All	1.99055332	Up	PREDICTED: ornithine decarboxylase 2-like [Hydra magnipapillata]
Unigene46619_All	1.985448814	Up	uncharacterized protein LOC100166388 [Acyrthosiphon pisum] >gi|239791407|dbj|BAH72175.1| ACYPI007265 [Acyrthosiphon pisum]
Unigene21247_All	1.982224486	Up	Nucleoside diphosphate kinase 6 [Acromyrmex echinatior]
CL1076.Contig1_All	1.98164142	Up	hypothetical protein AND_03396 [Anopheles darlingi]
Unigene63980_All	1.977200131	Up	serine palmitoyltransferase 2 [Mus musculus] >gi|6685600|sp|P97363.2|SPTC2_MOUSE RecName: Full=Serine palmitoyltransferase 2; AltName: Full=Long chain base biosynthesis protein 2; Short=LCB 2; AltName: Full=Long chain base biosynthesis protein 2a; Short=LCB2a; AltName:

			Full=Serine-palmitoyl-CoA transferase 2; Short=SPT 2 >gi|1838984|emb|CAA64898.1| serine C-palmitoyltransferase [Mus musculus] >gi|2267217|gb|AAC53310.1| serine palmitoyltransferase LCB2 subunit [Mus musculus] >gi|13096856|gb|AAH03227.1| Serine palmitoyltransferase, long chain base subunit 2 [Mus musculus] >gi|148670998|gb|EDL02945.1| serine palmitoyltransferase, long chain base subunit 2, isoform CRA_a [Mus musculus]
CL3645.Contig1_All	1.970495526	Up	aminomethyltransferase,putative [Pediculus humanus corporis] >gi|212505419|gb|EEB09880.1| aminomethyltransferase,putative [Pediculus humanus corporis]
Unigene41216_All	1.970161019	Up	pol-like protein [Biomphalaria glabrata]
CL1814.Contig2_All	1.966826659	Up	PREDICTED: UDP-glucuronosyltransferase 2B10-like [Acyrthosiphon pisum]
Unigene24032_All	1.965875689	Up	PREDICTED: similar to AGAP004744-PA [Tribolium castaneum]
Unigene49222_All	1.962371089	Up	NAD-specific glutamate dehydrogenase [Capsaspora owczarzaki ATCC 30864] >gi|320170749|gb|EFW47648.1| NAD-specific glutamate dehydrogenase [Capsaspora owczarzaki ATCC 30864]
CL2577.Contig2_All	1.956701088	Up	PREDICTED: peroxisomal multifunctional enzyme type 2 isoform 4 [Pan paniscus]
CL1082.Contig2_All	1.955912053	Up	acyl-coa dehydrogenase, putative [Perkinsus marinus ATCC 50983] >gi|239881088|gb|EER06951.1| acyl-coa dehydrogenase, putative [Perkinsus marinus ATCC 50983]
Unigene11554_All	1.955023964	Up	PREDICTED: lactosylceramide 4-alpha-galactosyltransferase-like [Megachile rotundata]
Unigene46599_All	1.942058619	Up	PREDICTED: inositol polyphosphate 5-phosphatase K-like [Bombus terrestris]
CL4882.Contig1_All	1.940190306	Up	Heat shock protein 67B2, putative [Pediculus humanus corporis] >gi|212513684|gb|EEB16164.1| Heat shock protein 67B2, putative [Pediculus humanus corporis]
CL5397.Contig4_All	1.934095104	Up	PREDICTED: uroporphyrinogen decarboxylase-like [Acyrthosiphon pisum]
Unigene19788_All	1.933321049	Up	PREDICTED: golgin subfamily A member 7B-like isoform 1 [Apis mellifera] >gi|380024401|ref|XP_003695987.1| PREDICTED: golgin subfamily A member 7B-like isoform 1 [Apis florea]
Unigene42127_All	1.932477738	Up	glutathione S-transferase sigma 5 [Locusta migratoria]
CL2785.Contig1_All	1.925620974	Up	PREDICTED: dihydropyrimidinase-like [Apis florea]
Unigene53802_All	1.925517267	Up	PREDICTED: uncharacterized protein LOC100889615 [Strongylocentrotus purpuratus]
Unigene73610_All	1.919369468	Up	NADP-dependent alcohol dehydrogenase, putative [Entamoeba nuttalli P19]

Unigene47124_All	1.91927138	Up	NADH dehydrogenase 1 alpha subcomplex subunit 2 [Esox lucius]
Unigene41555_All	1.918052614	Up	glucosyl glucuronosyl transferases [Locusta migratoria]
Unigene57979_All	1.915532018	Up	1-phosphatidylinositol-4,5-bisphosphate phosphodiesterase, putative [Pediculus humanus corporis] >gi|212505739|gb|EEB10119.1| 1-phosphatidylinositol-4,5-bisphosphate phosphodiesterase, putative [Pediculus humanus corporis]
CL3150.Contig2_All	1.914259508	Up	PREDICTED: similar to cytochrome c oxidase assembly protein cox11 [Tribolium castaneum] >gi|270004002|gb|EFA00450.1| hypothetical protein TcasGA2_TC003306 [Tribolium castaneum]
Unigene20005_All	1.914121951	Up	PREDICTED: inositol monophosphatase 1-like [Nasonia vitripennis]
Unigene14928_All	1.913620263	Up	hypothetical protein TcasGA2_TC014887 [Tribolium castaneum]
Unigene18162_All	1.911617887	Up	putative DNA-mediated transposase [Helicoverpa zea]
Unigene54400_All	1.910589614	Up	conserved hypothetical protein [Toxoplasma gondii GT1]
CL2055.Contig2_All	1.905925259	Up	receptor expression enhancing protein isoform 1 [Bombyx mori] >gi|87248615|gb|ABD36360.1| receptor expression enhancing protein isoform 1 [Bombyx mori]
CL4829.Contig2_All	1.904850847	Up	hypothetical protein DAPPUDRAFT_307444 [Daphnia pulex]
Unigene46241_All	1.900444581	Up	UDP-glucuronosyltransferase 1-7C [Camponotus floridanus]
Unigene11026_All	1.899547852	Up	GK15915 [Drosophila willistoni] >gi|194159975|gb|EDW74876.1| GK15915 [Drosophila willistoni]
CL85.Contig1_All	1.896051109	Up	hypothetical protein - bloodfluke planorb (fragment)
Unigene20901_All	1.890907705	Up	AAEL002349-PA [Aedes aegypti]
Unigene2715_All	1.887679351	Up	PREDICTED: phosphatidate cytidylyltransferase, photoreceptor-specific-like [Bombus impatiens]
Unigene47430_All	1.885536433	Up	PREDICTED: FAD synthase-like [Gallus gallus]
Unigene56364_All	1.884236362	Up	PREDICTED: probable aldehyde oxidase 2-like [Megachile rotundata]
Unigene23264_All	1.883511747	Up	PREDICTED: dihydropyrimidine dehydrogenase [NADP(+)]-like [Megachile rotundata]
Unigene23273_All	1.875991999	Up	PREDICTED: similar to fatty acid synthase [Tribolium castaneum]
Unigene66889_All	1.874995095	Up	PREDICTED: uncharacterized protein LOC100442257 [Pongo abelii]
CL5743.Contig2_All	1.874985126	Up	peroxisomal multifunctional enzyme [Dictyostelium fasciculatum] >gi|328873745|gb|EGG22112.1| peroxisomal multifunctional enzyme [Dictyostelium fasciculatum]
Unigene40779_All	1.874947146	Up	PREDICTED: glutamine synthetase-like [Oryzias latipes]

Unigene59152_All	1.874906962	Up	PREDICTED: glutamyl-tRNA(Gln) amidotransferase subunit A, mitochondrial-like [Takifugu rubripes]
CL4884.Contig1_All	1.865917721	Up	GH22329 [Drosophila grimshawi] >gi|195107698|ref|XP_001998445.1| GI23969 [Drosophila mojavensis] >gi|193899709|gb|EDV98575.1| GH22329 [Drosophila grimshawi] >gi|193915039|gb|EDW13906.1| GI23969 [Drosophila mojavensis]
CL4274.Contig1_All	1.865077614	Up	peroxisomal 3-ketoacyl-CoA thiolase B [Capsaspora owczarzaki ATCC 30864] >gi|320167663|gb|EFW44562.1| peroxisomal 3-ketoacyl-CoA thiolase B [Capsaspora owczarzaki ATCC 30864]
Unigene5342_All	1.861328892	Up	PREDICTED: DNA-directed RNA polymerase I subunit RPA2-like [Megachile rotundata]
CL996.Contig2_All	1.847070195	Up	juvenile hormone esterase [Gryllus assimilis]
Unigene60589_All	1.841781475	Up	PREDICTED: NADH dehydrogenase (ubiquinone) Fe-S protein 4-like [Saccoglossus kowalevskii]
Unigene16893_All	1.839578659	Up	Glycerate kinase, partial [Columba livia]
CL7185.Contig1_All	1.835489162	Up	ATPase [Homo sapiens]
CL4602.Contig1_All	1.834277394	Up	PREDICTED: piggyBac transposable element-derived protein 3-like, partial [Oryzias latipes]
Unigene15761_All	1.826672672	Up	PREDICTED: similar to presqualene diphosphate phosphatase [Tribolium castaneum] >gi|270003950|gb|EFA00398.1| hypothetical protein TcasGA2_TC003248 [Tribolium castaneum]
CL2553.Contig1_All	1.826075719	Up	pol-like protein [Biomphalaria glabrata]
Unigene58641_All	1.825018471	Up	1-phosphatidylinositol-4,5-bisphosphate phosphodiesterase classes I and II [Acromyrmex echinatior]
Unigene58164_All	1.818432747	Up	ATP synthase subunit O, mitochondrial [Acromyrmex echinatior]
Unigene5530_All	1.818399638	Up	hypothetical protein DAPPUDRAFT_205741 [Daphnia pulex]
Unigene46397_All	1.814561307	Up	hypothetical protein TcasGA2_TC016048 [Tribolium castaneum]
Unigene24428_All	1.812129957	Up	hypothetical protein SINV_06379 [Solenopsis invicta]
Unigene41310_All	1.809905456	Up	PREDICTED: uncharacterized protein K02A2.6-like [Oreochromis niloticus]
Unigene18299_All	1.806611958	Up	hypothetical protein BRAFLDRAFT_266349 [Branchiostoma floridae] >gi|229288520|gb|EEN59214.1| hypothetical protein BRAFLDRAFT_266349 [Branchiostoma floridae]
Unigene60249_All	1.801054722	Up	acyl carrier protein [Naegleria gruberi] >gi|284089602|gb|EFC43258.1| acyl carrier protein [Naegleria gruberi]
Unigene58159_All	1.800943299	Up	PREDICTED: hypothetical protein LOC100118182 [Nasonia vitripennis]

Unigene63206_All	1.800892157	Up	NADH-ubiquinone oxidoreductase subunit B17.2, putative [Pediculus humanus corporis] >gi|212507854|gb|EEB11716.1| NADH-ubiquinone oxidoreductase subunit B17.2, putative [Pediculus humanus corporis]
CL4801.Contig1_All	1.799815729	Up	Arginase-1 [Camponotus floridanus]
Unigene18827_All	1.793610476	Up	PREDICTED: similar to AGAP007531-PA [Tribolium castaneum] >gi|270001821|gb|EEZ98268.1| hypothetical protein TcasGA2_TC000711 [Tribolium castaneum]
CL9642.Contig2_All	1.782533922	Up	hypothetical protein KGM_20919 [Danaus plexippus]
CL5606.Contig1_All	1.779442006	Up	conserved hypothetical protein [Pediculus humanus corporis] >gi|212516741|gb|EEB18715.1| conserved hypothetical protein [Pediculus humanus corporis]
Unigene22470_All	1.777644817	Up	Alpha-1,2-mannosyltransferase ALG9 [Camponotus floridanus]
Unigene21445_All	1.767508231	Up	PREDICTED: acetyl-CoA acetyltransferase, cytosolic-like [Nasonia vitripennis]
CL294.Contig1_All	1.765151159	Up	PREDICTED: alanine aminotransferase 2-like [Acyrthosiphon pisum]
Unigene20732_All	1.760670461	Up	PREDICTED: methylcrotonoyl-CoA carboxylase subunit alpha, mitochondrial-like [Nasonia vitripennis]
CL9690.Contig1_All	1.759529672	Up	GK19782 [Drosophila willistoni] >gi|194160377|gb|EDW75278.1| GK19782 [Drosophila willistoni]
Unigene2171_All	1.752500062	Up	PREDICTED: similar to MGC82032 protein [Tribolium castaneum] >gi|270010868|gb|EFA07316.1| hypothetical protein TcasGA2_TC015909 [Tribolium castaneum]
Unigene2625_All	1.751509644	Up	PREDICTED: nicotinate phosphoribosyltransferase-like isoform 1 [Bombus terrestris] >gi|350410409|ref|XP_003489036.1| PREDICTED: nicotinate phosphoribosyltransferase-like isoform 1 [Bombus impatiens]
Unigene10555_All	1.740884806	Up	Transmembrane protein 15 [Harpegnathos saltator]
Unigene5286_All	1.739946518	Up	PREDICTED: UDP-N-acetylhexosamine pyrophosphorylase-like [Acyrthosiphon pisum]
Unigene46631_All	1.735626949	Up	kynurenine 3-monooxygenase [Anopheles stephensi]
CL2784.Contig4_All	1.734158804	Up	PREDICTED: choline/ethanolamine kinase-like [Bombus impatiens]
CL6811.Contig2_All	1.72347589	Up	PREDICTED: isovaleryl-CoA dehydrogenase, mitochondrial-like [Megachile rotundata]
CL5999.Contig1_All	1.722897388	Up	PREDICTED: phosphoribosylformylglycinamidine synthase-like [Megachile rotundata]
Unigene5475_All	1.721540269	Up	N-acetyllactosaminide beta-1,3-N-acetylglucosaminyltransferase, putative [Pediculus humanus corporis] >gi|212518216|gb|EEB19993.1| N-acetyllactosaminide beta-1,3-N-acetylglucosaminyltransferase, putative [Pediculus humanus corporis]

Unigene47463_All	1.720719921	Up	GK20524 [Drosophila willistoni] >gi|194164627|gb|EDW79528.1| GK20524 [Drosophila willistoni]
Unigene24112_All	1.719914436	Up	PREDICTED: similar to vacuolar ATP synthase subunit S1 [Tribolium castaneum] >gi|270014778|gb|EFA11226.1| hypothetical protein TcasGA2_TC010758 [Tribolium castaneum]
Unigene15751_All	1.719235616	Up	hypothetical protein TcasGA2_TC006332 [Tribolium castaneum]
CL8667.Contig2_All	1.718150282	Up	Beta-1,4-galactosyltransferase 7 [Harpegnathos saltator]
Unigene11201_All	1.713698448	Up	hypothetical protein BRAFLDRAFT_219230 [Branchiostoma floridae] >gi|229281893|gb|EEN52646.1| hypothetical protein BRAFLDRAFT_219230 [Branchiostoma floridae]
Unigene52966_All	1.706873966	Up	Phosphatidic acid phosphatase type 2 domain-containing protein 1A [Acromyrmex echinatior]
Unigene18600_All	1.703416738	Up	Phosphatidylinositol 4-kinase alpha, putative [Pediculus humanus corporis] >gi|212518501|gb|EEB20254.1| Phosphatidylinositol 4-kinase alpha, putative [Pediculus humanus corporis]
Unigene54548_All	1.702747974	Up	PREDICTED: similar to conserved hypothetical protein [Tribolium castaneum] >gi|270008773|gb|EFA05221.1| hypothetical protein TcasGA2_TC015362 [Tribolium castaneum]
Unigene19111_All	1.700256078	Up	Phosphatidylinositol N-acetylglucosaminyltransferase subunit C, putative [Pediculus humanus corporis] >gi|212517284|gb|EEB19195.1| Phosphatidylinositol N-acetylglucosaminyltransferase subunit C, putative [Pediculus humanus corporis]
Unigene45962_All	1.697993395	Up	PREDICTED: methylenetetrahydrofolate reductase [Cricetulus griseus]
CL351.Contig1_All	1.693719126	Up	molybdopterin cofactor synthesis protein A, putative [Pediculus humanus corporis] >gi|212512166|gb|EEB14987.1| molybdopterin cofactor synthesis protein A, putative [Pediculus humanus corporis]
Unigene18124_All	1.692081855	Up	PREDICTED: putative glycerol kinase 3-like [Bombus impatiens]
Unigene41430_All	1.690536613	Up	RecName: Full=Cytochrome c oxidase subunit 5B, mitochondrial; AltName: Full=Cytochrome c oxidase polypeptide Vb; Flags: Precursor >gi|192924|gb|AAA37515.1| cytochrome c oxidase subunit Vb [Mus musculus]
Unigene13143_All	1.690284296	Up	PREDICTED: similar to acyl-coa dehydrogenase isoform 1 [Tribolium castaneum] >gi|270014595|gb|EFA11043.1| hypothetical protein TcasGA2_TC004634 [Tribolium

			castaneum]
Unigene13004_All	1.685027395	Up	Heparan-alpha-glucosaminide N-acetyltransferase [Harpegnathos saltator]
Unigene48762_All	1.682349158	Up	uncharacterized protein LOC100166388 [Acyrthosiphon pisum] >gi|239791407|dbj|BAH72175.1| ACYPI007265 [Acyrthosiphon pisum]
Unigene13533_All	1.682309619	Up	PREDICTED: similar to glycerol-3-phosphate acyltransferase [Tribolium castaneum] >gi|270013527|gb|EFA09975.1| hypothetical protein TcasGA2_TC012134 [Tribolium castaneum]
Unigene53037_All	1.682275831	Up	--
Unigene61179_All	1.682247947	Up	PREDICTED: adenosylhomocysteinase-like [Ovis aries] >gi|426241354|ref|XP_004014556.1| PREDICTED: adenosylhomocysteinase-like [Ovis aries]
Unigene20716_All	1.675053443	Up	PREDICTED: CTP synthase-like [Apis mellifera]
Unigene55308_All	1.668452502	Up	Aminopeptidase N [Harpegnathos saltator]
Unigene60191_All	1.668404459	Up	DNA polymerase subunit gamma 1, mitochondrial [Culex quinquefasciatus] >gi|167873869|gb|EDS37252.1| DNA polymerase subunit gamma 1, mitochondrial [Culex quinquefasciatus]
Unigene5898_All	1.667003752	Up	hypothetical protein SINV_16184 [Solenopsis invicta]
Unigene24080_All	1.666363928	Up	PREDICTED: delta-aminolevulinic acid dehydratase-like isoform 2 [Bombus impatiens]
Unigene387_All	1.660829995	Up	PREDICTED: cytoplasmic phosphatidylinositol transfer protein 1-like [Bombus terrestris]
Unigene18298_All	1.656742691	Up	Amidophosphoribosyltransferase [Acromyrmex echinatior]
CL3361.Contig1_All	1.65411815	Up	PREDICTED: sphingomyelin phosphodiesterase-like isoform 3 [Acyrthosiphon pisum]
Unigene67760_All	1.647489208	Up	PREDICTED: aminopeptidase N-like [Xenopus (Silurana) tropicalis]
Unigene23259_All	1.645760077	Up	PREDICTED: similar to mannosidase alpha class 2a [Tribolium castaneum] >gi|270009868|gb|EFA06316.1| hypothetical protein TcasGA2_TC009186 [Tribolium castaneum]
Unigene53346_All	1.640555923	Up	PREDICTED: THAP domain-containing protein 9-like [Acyrthosiphon pisum]
Unigene3702_All	1.638567444	Up	Phosphatidylserine synthase, putative [Pediculus humanus corporis] >gi|212508225|gb|EEB11994.1| Phosphatidylserine synthase, putative [Pediculus humanus corporis]
CL4721.Contig1_All	1.633927357	Up	PREDICTED: lactosylceramide 4-alpha-galactosyltransferase-like [Megachile rotundata]

Unigene13133_All	1.631630507	Up	PREDICTED: similar to polypeptide GalNAc transferase 5 CG31651-PA [Tribolium castaneum] >gi|270011823|gb|EFA08271.1| hypothetical protein TcasGA2_TC005902 [Tribolium castaneum]
Unigene597_All	1.629804863	Up	Serine/threonine kinase 11-interacting protein [Harpegnathos saltator]
Unigene51540_All	1.629804622	Up	uncharacterized protein LOC100216301 [Xenopus (Silurana) tropicalis]
CL5397.Contig1_All	1.625886246	Up	PREDICTED: uroporphyrinogen decarboxylase-like [Acyrthosiphon pisum]
CL1722.Contig2_All	1.622578073	Up	PREDICTED: mannose-6-phosphate isomerase-like [Apis mellifera]
Unigene9222_All	1.616924265	Up	PREDICTED: beta-1,3-galactosyltransferase 1-like isoform 2 [Nasonia vitripennis]
CL2294.Contig2_All	1.616187481	Up	homogentisate 1,2-dioxygenase [Culex quinquefasciatus] >gi|167874202|gb|EDS37585.1| homogentisate 1,2-dioxygenase [Culex quinquefasciatus]
Unigene24695_All	1.614895191	Up	PREDICTED: hypothetical protein LOC409658 [Apis mellifera]
CL5757.Contig2_All	1.614649728	Up	PREDICTED: c-1-tetrahydrofolate synthase, cytoplasmic-like [Megachile rotundata]
Unigene24851_All	1.613462435	Up	Hydroxysteroid dehydrogenase-like protein 1 [Columba livia]
Unigene46316_All	1.611934738	Up	PREDICTED: galactosylgalactosylxylosylprotein 3-beta-glucuronosyltransferase P-like [Acyrthosiphon pisum]
CL8014.Contig1_All	1.59408999	Up	PREDICTED: similar to hydroxyacyl-coenzyme A dehydrogenase [Tribolium castaneum]
CL2824.Contig1_All	1.592804936	Up	malate synthase [Capsaspora owczarzaki ATCC 30864] >gi|320165110|gb|EFW42009.1| malate synthase [Capsaspora owczarzaki ATCC 30864]
Unigene11335_All	1.589808126	Up	PREDICTED: phosphatidate phosphatase PPAPDC1A-like [Bombus terrestris]
Unigene24426_All	1.580186813	Up	C-4 methyl sterol oxidase [Dictyostelium discoideum AX4] >gi|60474307|gb|EAL72244.1| C-4 methyl sterol oxidase [Dictyostelium discoideum AX4]
CL7603.Contig2_All	1.579607343	Up	hypothetical protein TcasGA2_TC008338 [Tribolium castaneum]
Unigene58712_All	1.575383293	Up	PREDICTED: type I inositol-3,4-bisphosphate 4-phosphatase-like [Bombus impatiens]
Unigene3861_All	1.569538317	Up	DNA primase large subunit [Danaus plexippus]
Unigene22698_All	1.56672118	Up	PREDICTED: adenosylhomocysteinase-like isoform 1 [Bombus terrestris]
CL10030.Contig2_All	1.559479501	Up	pol-like protein [Biomphalaria glabrata]
Unigene7136_All	1.558468197	Up	pro-phenoloxidase 1 [Diabolocatantops pinguis]
CL6762.Contig2_All	1.55683345	Up	hypothetical protein TcasGA2_TC012513 [Tribolium castaneum]

<table>
<tr><td>CL5393.Contig1_All</td><td>1.550445814</td><td>Up</td><td>PREDICTED: hypothetical protein LOC100570266 [Acyrthosiphon pisum]</td></tr>
<tr><td>Unigene22623_All</td><td>1.545746323</td><td>Up</td><td>RecName: Full=V-type proton ATPase subunit B; Short=V-ATPase subunit B; AltName: Full=Vacuolar proton pump subunit B >gi|9714|emb|CAA45706.1| H(+)-transporting ATPase [Manduca sexta]</td></tr>
<tr><td>CL5476.Contig2_All</td><td>1.540415303</td><td>Up</td><td>fatty acid beta-oxidation complex subunit beta [Heliothis virescens]</td></tr>
<tr><td>Unigene5833_All</td><td>1.536356223</td><td>Up</td><td>PREDICTED: uncharacterized protein LOC100207270 [Hydra magnipapillata]</td></tr>
<tr><td>CL10015.Contig1_All</td><td>1.532097487</td><td>Up</td><td>hypothetical protein - bloodfluke planorb (fragment)</td></tr>
<tr><td>Unigene16495_All</td><td>1.527747334</td><td>Up</td><td>PREDICTED: probable uridine-cytidine kinase-like isoform 2 [Nasonia vitripennis]</td></tr>
<tr><td>Unigene7848_All</td><td>1.526369872</td><td>Up</td><td>glutaryl-CoA dehydrogenase, mitochondrial precursor [Bos taurus] >gi|122142797|sp|Q2KHZ9.1|GCDH_BOVIN RecName: Full=Glutaryl-CoA dehydrogenase, mitochondrial; Short=GCD; Flags: Precursor >gi|86438384|gb|AAI12823.1| Glutaryl-Coenzyme A dehydrogenase [Bos taurus] >gi|296485913|tpg|DAA28028.1| TPA: glutaryl-CoA dehydrogenase, mitochondrial precursor [Bos taurus]</td></tr>
<tr><td>CL6158.Contig2_All</td><td>1.524377548</td><td>Up</td><td>Protein GLN-3, isoform b [Caenorhabditis elegans] >gi|5824686|emb|CAB54376.1| Protein GLN-3, isoform b [Caenorhabditis elegans]</td></tr>
<tr><td>CL5752.Contig1_All</td><td>1.523806987</td><td>Up</td><td>hypothetical protein TcasGA2_TC007759 [Tribolium castaneum]</td></tr>
<tr><td>CL4923.Contig1_All</td><td>1.52167088</td><td>Up</td><td>PREDICTED: TBC1 domain family member 24-like isoform 1 [Bombus impatiens]</td></tr>
<tr><td>CL5219.Contig1_All</td><td>1.518446112</td><td>Up</td><td>uridine phosphorylase, putative [Pediculus humanus corporis] >gi|212513787|gb|EEB16220.1| uridine phosphorylase, putative [Pediculus humanus corporis]</td></tr>
<tr><td>CL2352.Contig2_All</td><td>1.518070887</td><td>Up</td><td>PREDICTED: similar to AGAP005170-PA [Tribolium castaneum] >gi|270009811|gb|EFA06259.1| hypothetical protein TcasGA2_TC009118 [Tribolium castaneum]</td></tr>
<tr><td>CL9526.Contig1_All</td><td>1.517685204</td><td>Up</td><td>pol-like protein [Biomphalaria glabrata]</td></tr>
<tr><td>Unigene7045_All</td><td>1.514303603</td><td>Up</td><td>PREDICTED: similar to dihydrolipoamide succinyltransferase component of 2-oxoglutarate dehydrogenase [Tribolium castaneum]</td></tr>
<tr><td>Unigene13080_All</td><td>1.51360268</td><td>Up</td><td>UDP-GalNAc:polypeptide N-acetylgalactosaminyltransferase, putative [Pediculus humanus corporis] >gi|212517982|gb|EEB19792.1| UDP-GalNAc:polypeptide N-acetylgalactosaminyltransferase, putative [Pediculus humanus corporis]</td></tr>
<tr><td>CL2784.Contig5_All</td><td>1.512110282</td><td>Up</td><td>PREDICTED: choline/ethanolamine kinase-like [Bombus impatiens]</td></tr>
<tr><td>Unigene53796_All</td><td>1.511060881</td><td>Up</td><td>PREDICTED: aspartate aminotransferase, cytoplasmic-like, partial [Mus musculus]</td></tr>
</table>

<table>
<tr><td>CL8227.Contig1_All</td><td>1.496403235</td><td>Up</td><td>PREDICTED: transaldolase-like [Strongylocentrotus purpuratus]</td></tr>
<tr><td>CL5623.Contig1_All</td><td>1.491994252</td><td>Up</td><td>conserved hypothetical protein [Capsaspora owczarzaki ATCC 30864] >gi|320168370|gb|EFW45269.1| conserved hypothetical protein [Capsaspora owczarzaki ATCC 30864]</td></tr>
<tr><td>CL3718.Contig3_All</td><td>1.490966411</td><td>Up</td><td>PREDICTED: similar to GA19017-PA [Tribolium castaneum] >gi|270001651|gb|EEZ98098.1| hypothetical protein TcasGA2_TC000511 [Tribolium castaneum]</td></tr>
<tr><td>CL7695.Contig2_All</td><td>1.488760764</td><td>Up</td><td>DNA polymerase alpha subunit B [Crassostrea gigas]</td></tr>
<tr><td>CL4381.Contig1_All</td><td>1.483350833</td><td>Up</td><td>PREDICTED: similar to AGAP002711-PA [Tribolium castaneum]</td></tr>
<tr><td>Unigene308_All</td><td>1.482088908</td><td>Up</td><td>hypothetical protein AND_15863 [Anopheles darlingi]</td></tr>
<tr><td>CL1143.Contig2_All</td><td>1.474689086</td><td>Up</td><td>NADH kinase, putative [Pediculus humanus corporis] >gi|212507761|gb|EEB11623.1| NADH kinase, putative [Pediculus humanus corporis]</td></tr>
<tr><td>Unigene18223_All</td><td>1.474501771</td><td>Up</td><td>PREDICTED: bifunctional heparan sulfate N-deacetylase/N-sulfotransferase-like [Nasonia vitripennis]</td></tr>
<tr><td>Unigene16690_All</td><td>1.473598651</td><td>Up</td><td>PREDICTED: similar to n-acetylgalactosaminyltransferase [Tribolium castaneum] >gi|270005204|gb|EFA01652.1| hypothetical protein TcasGA2_TC007223 [Tribolium castaneum]</td></tr>
<tr><td>Unigene2710_All</td><td>1.466690051</td><td>Up</td><td>PREDICTED: DNA-directed RNA polymerase III subunit RPC2 [Megachile rotundata]</td></tr>
<tr><td>Unigene8377_All</td><td>1.463059646</td><td>Up</td><td>PREDICTED: 1-phosphatidylinositol-4,5-bisphosphate phosphodiesterase-like isoform 1 [Acyrthosiphon pisum] >gi|328724636|ref|XP_003248206.1| PREDICTED: 1-phosphatidylinositol-4,5-bisphosphate phosphodiesterase-like isoform 2 [Acyrthosiphon pisum]</td></tr>
<tr><td>CL1485.Contig1_All</td><td>1.459939223</td><td>Up</td><td>uridine 5'-monophosphate synthase [Culex quinquefasciatus] >gi|167867356|gb|EDS30739.1| uridine 5'-monophosphate synthase [Culex quinquefasciatus]</td></tr>
<tr><td>CL1459.Contig2_All</td><td>1.459922598</td><td>Up</td><td>PREDICTED: V-type proton ATPase subunit e 2-like [Nasonia vitripennis]</td></tr>
<tr><td>Unigene4617_All</td><td>1.459883155</td><td>Up</td><td>FAD synthase [Chelonia mydas]</td></tr>
<tr><td>CL6454.Contig2_All</td><td>1.459812277</td><td>Up</td><td>PREDICTED: choline/ethanolaminephosphotransferase 1-like [Apis florea]</td></tr>
<tr><td>Unigene11154_All</td><td>1.455761127</td><td>Up</td><td>PREDICTED: uncharacterized protein C05D11.1-like [Nasonia vitripennis]</td></tr>
<tr><td>Unigene5417_All</td><td>1.454471419</td><td>Up</td><td>FAD oxidoreductase [Culex quinquefasciatus] >gi|167880398|gb|EDS43781.1| FAD oxidoreductase [Culex quinquefasciatus]</td></tr>
<tr><td>Unigene11216_All</td><td>1.44843075</td><td>Up</td><td>hypothetical protein TcasGA2_TC010363 [Tribolium castaneum]</td></tr>
<tr><td>CL6699.Contig1_All</td><td>1.442805482</td><td>Up</td><td>PREDICTED: similar to zinc binding dehydrogenase [Tribolium</td></tr>
</table>

			castaneum] >gi|270012629|gb|EFA09077.1| hypothetical protein TcasGA2_TC006794 [Tribolium castaneum]
CL65.Contig2_All	1.435692539	Up	PREDICTED: calcium-independent phospholipase A2-gamma-like [Nasonia vitripennis]
Unigene49924_All	1.432971263	Up	PREDICTED: DNA polymerase epsilon catalytic subunit A-like isoform 2 [Acyrthosiphon pisum]
Unigene25098_All	1.430529061	Up	PREDICTED: 3-ketoacyl-CoA thiolase, mitochondrial-like [Bombus impatiens]
Unigene18279_All	1.428748791	Up	PREDICTED: similar to n-acetylgalactosaminyltransferase [Tribolium castaneum] >gi|270006291|gb|EFA02739.1| hypothetical protein TcasGA2_TC008465 [Tribolium castaneum]
Unigene21557_All	1.422295192	Up	GPI ethanolamine phosphate transferase, putative [Pediculus humanus corporis] >gi|212511249|gb|EEB14273.1| GPI ethanolamine phosphate transferase, putative [Pediculus humanus corporis]
Unigene18817_All	1.417762983	Up	PREDICTED: 6-phosphogluconolactonase-like [Nasonia vitripennis]
CL4824.Contig2_All	1.414158777	Up	ORF2-encoded protein [Danio rerio]
Unigene18609_All	1.409559692	Up	beta-hexosaminidase beta chain precursor, putative [Pediculus humanus corporis] >gi|212513146|gb|EEB15774.1| beta-hexosaminidase beta chain precursor, putative [Pediculus humanus corporis]
Unigene10364_All	1.40639578	Up	four wheel drive [Tribolium castaneum]
Unigene9821_All	1.392386466	Up	Phosphatidylinositol-4-phosphate 3-kinase C2 domain-containing beta polypeptide [Acromyrmex echinatior]
Unigene23917_All	1.39206296	Up	Dihydrolipoyllysine-residue succinyltransferase component of 2-oxoglutarate dehydrogenase, putative [Pediculus humanus corporis] >gi|212516425|gb|EEB18438.1| Dihydrolipoyllysine-residue succinyltransferase component of 2-oxoglutarate dehydrogenase, putative [Pediculus humanus corporis]
CL1143.Contig1_All	1.391917174	Up	NADH kinase, putative [Pediculus humanus corporis] >gi|212507761|gb|EEB11623.1| NADH kinase, putative [Pediculus humanus corporis]
Unigene23353_All	1.390421087	Up	serine palmitoyltransferase, putative [Pediculus humanus corporis] >gi|212510255|gb|EEB13466.1| serine palmitoyltransferase, putative [Pediculus humanus corporis]
Unigene9298_All	1.386876061	Up	PREDICTED: ATP synthase subunit alpha, mitochondrial isoform 2 [Ovis aries]
CL2209.Contig1_All	1.383975301	Up	polyprotein [Bombyx mori]

Unigene20787_All	1.383238463	Up	glucosyl glucuronosyl transferases [Locusta migratoria]
CL3944.Contig1_All	1.381865089	Up	PREDICTED: probable GDP-L-fucose synthase-like [Megachile rotundata]
Unigene2762_All	1.380960568	Up	PREDICTED: similar to phosphatidylinositol 3-kinase catalytic subunit type 3 [Tribolium castaneum] >gi|270001744|gb|EEZ98191.1| hypothetical protein TcasGA2_TC000620 [Tribolium castaneum]
Unigene4540_All	1.380115513	Up	PREDICTED: 5-formyltetrahydrofolate cyclo-ligase-like [Danio rerio]
Unigene15601_All	1.378322012	Up	Striatin, putative [Pediculus humanus corporis] >gi|212510257|gb|EEB13468.1| Striatin, putative [Pediculus humanus corporis]
Unigene21091_All	1.377713214	Up	PREDICTED: ceramide synthase 6-like [Bombus impatiens]
Unigene50385_All	1.377505635	Up	IgA-specific serine endopeptidase, putative [Acanthamoeba castellanii str. Neff] >gi|440796236|gb|ELR17345.1| IgA-specific serine endopeptidase, putative [Acanthamoeba castellanii str. Neff]
CL6289.Contig1_All	1.377445871	Up	phosphoserine phosphatase [Locusta migratoria]
Unigene15512_All	1.376544637	Up	pol-like protein [Biomphalaria glabrata]
CL9778.Contig1_All	1.375724231	Up	PREDICTED: required for meiotic nuclear division protein 1 homolog [Nasonia vitripennis]
CL1274.Contig1_All	1.373811383	Up	ATP synthase subunit gamma [Capsaspora owczarzaki ATCC 30864] >gi|320169247|gb|EFW46146.1| ATP synthase subunit gamma [Capsaspora owczarzaki ATCC 30864]
Unigene5146_All	1.367227039	Up	PREDICTED: uncharacterized protein LOC100882671 [Megachile rotundata]
Unigene18422_All	1.363103189	Up	PREDICTED: geranylgeranyl pyrophosphate synthase-like [Nasonia vitripennis]
Unigene54657_All	1.362212412	Up	PREDICTED: RNA-directed DNA polymerase from mobile element jockey-like [Strongylocentrotus purpuratus]
CL7298.Contig2_All	1.356243988	Up	Bifunctional coenzyme A synthase [Camponotus floridanus]
Unigene47812_All	1.354991089	Up	PREDICTED: hypothetical protein LOC100747839 [Bombus impatiens]
Unigene7144_All	1.354108332	Up	PREDICTED: phosphatidylinositol transfer protein alpha isoform-like [Nasonia vitripennis]
Unigene2385_All	1.352025016	Up	protein FAM11A, putative [Pediculus humanus corporis] >gi|212512678|gb|EEB15397.1| protein FAM11A, putative [Pediculus humanus corporis]
Unigene54067_All	1.350041653	Up	Aldehyde dehydrogenase family 16 member A1 [Crassostrea gigas]
Unigene3371_All	1.349599217	Up	vacuolar ATPase subunit H [Locusta migratoria manilensis]

Unigene13305_All	1.349284757	Up	PREDICTED: similar to glutamate cysteine ligase isoform 1 [Tribolium castaneum] >gi|270008080|gb|EFA04528.1| hypothetical protein TcasGA2_TC016323 [Tribolium castaneum]
Unigene453_All	1.34890723	Up	PREDICTED: 2-aminoethanethiol dioxygenase-like [Acyrthosiphon pisum]
Unigene15042_All	1.347548983	Up	beta-alanyl conjugating enzyme [Periplaneta americana]
Unigene7756_All	1.346867819	Up	vacuolar ATP synthase 21 kDa proteolipid subunit [Culex quinquefasciatus] >gi|167880111|gb|EDS43494.1| vacuolar ATP synthase 21 kDa proteolipid subunit [Culex quinquefasciatus]
CL6432.Contig2_All	1.344883033	Up	PREDICTED: phosphatidylinositol 4-kinase type 2-beta-like [Acyrthosiphon pisum]
CL2625.Contig2_All	1.341763146	Up	PREDICTED: peroxisomal acyl-coenzyme A oxidase 3-like isoform 3 [Acyrthosiphon pisum] >gi|328721041|ref|XP_003247198.1| PREDICTED: peroxisomal acyl-coenzyme A oxidase 3-like isoform 4 [Acyrthosiphon pisum]
Unigene25563_All	1.335751388	Up	AGAP002933-PA [Anopheles gambiae str. PEST] >gi|333467792|gb|EAA07616.5| AGAP002933-PA [Anopheles gambiae str. PEST]
CL9795.Contig1_All	1.335171806	Up	PREDICTED: putative glutamate synthase [NADPH]-like [Acyrthosiphon pisum]
CL9928.Contig1_All	1.332257254	Up	PREDICTED: RNA-directed DNA polymerase from mobile element jockey-like [Strongylocentrotus purpuratus]
Unigene24177_All	1.325618283	Up	PREDICTED: uncharacterized protein LOC755078 [Strongylocentrotus purpuratus]
Unigene18870_All	1.32123503	Up	isopentenyl pyrophosphate:dimethylallyl pyrophosphate isomerase [Culex quinquefasciatus] >gi|167863496|gb|EDS26879.1| isopentenyl pyrophosphate:dimethylallyl pyrophosphate isomerase [Culex quinquefasciatus]
Unigene14342_All	1.319818592	Up	conserved hypothetical protein [Culex quinquefasciatus] >gi|167865828|gb|EDS29211.1| conserved hypothetical protein [Culex quinquefasciatus]
Unigene154_All	1.316167974	Up	pol-like protein [Biomphalaria glabrata]
Unigene3077_All	1.31290414	Up	PREDICTED: LOW QUALITY PROTEIN: polyphosphoinositide phosphatase-like [Apis florea]
Unigene6445_All	1.312336879	Up	succinic semialdehyde dehydrogenase [Ctenocephalides felis]
Unigene3342_All	1.307711502	Up	hypothetical protein BRAFLDRAFT_270381 [Branchiostoma floridae] >gi|229286768|gb|EEN57484.1| hypothetical protein BRAFLDRAFT_270381 [Branchiostoma floridae]

CL3503.Contig2_All	1.305562802	Up	PREDICTED: uridine-cytidine kinase-like 1-like isoform 1 [Bombus terrestris] >gi|340720303|ref|XP_003398580.1| PREDICTED: uridine-cytidine kinase-like 1-like isoform 2 [Bombus terrestris]
Unigene53479_All	1.30464596	Up	hypothetical protein TcasGA2_TC030617 [Tribolium castaneum]
Unigene60642_All	1.297177005	Up	PREDICTED: 1-phosphatidylinositol-4,5-bisphosphate phosphodiesterase gamma-1-like [Apis florea]
Unigene4304_All	1.294680708	Up	acetyl-CoA acetyltransferase [Bombus terrestris]
Unigene7803_All	1.290397842	Up	hypothetical protein DAPPUDRAFT_302871 [Daphnia pulex]
Unigene3658_All	1.284832709	Up	PREDICTED: cytochrome b-c1 complex subunit 7-like [Hydra magnipapillata]
CL5999.Contig2_All	1.281145897	Up	PREDICTED: phosphoribosylformylglycinamidine synthase-like [Megachile rotundata]
Unigene1221_All	1.280920355	Up	Cytochrome b-c1 complex subunit 9 [Acromyrmex echinatior]
Unigene374_All	1.273688347	Up	PREDICTED: similar to 5-aminolevulinic acid synthase [Tribolium castaneum]
Unigene54796_All	1.271891716	Up	GG11199 [Drosophila erecta] >gi|190656790|gb|EDV54022.1| GG11199 [Drosophila erecta]
Unigene42819_All	1.27134648	Up	hypothetical protein TcasGA2_TC010280 [Tribolium castaneum]
Unigene1882_All	1.26823924	Up	putative V-ATPase C-subunit [Lutzomyia longipalpis]
Unigene53006_All	1.26724566	Up	PREDICTED: glucosylceramidase-like isoform 1 [Apis mellifera]
Unigene13781_All	1.26490448	Up	PREDICTED: type I inositol-3,4-bisphosphate 4-phosphatase-like isoform 3 [Nasonia vitripennis]
CL5567.Contig2_All	1.254658389	Up	enzymatic polyprotein; Endonuclease; Reverse transcriptase, putative [Pediculus humanus corporis] >gi|212516797|gb|EEB18765.1| enzymatic polyprotein; Endonuclease; Reverse transcriptase, putative [Pediculus humanus corporis]
Unigene60222_All	1.253443398	Up	PREDICTED: similar to heparanase-like protein [Tribolium castaneum]
CL8213.Contig1_All	1.252054845	Up	PREDICTED: uncharacterized protein LOC100889850 [Strongylocentrotus purpuratus]
Unigene7792_All	1.249189905	Up	reverse transcriptase [Bombyx mori]
CL4186.Contig3_All	1.248051023	Up	PREDICTED: regucalcin-like isoform 1 [Nasonia vitripennis] >gi|345478834|ref|XP_003423819.1| PREDICTED: regucalcin-like isoform 2 [Nasonia vitripennis]
Unigene23459_All	1.245360014	Up	PREDICTED: diphosphomevalonate decarboxylase-like [Apis mellifera]
Unigene10554_All	1.237689592	Up	gdp mannose-4,6-dehydratase [Aedes aegypti] >gi|108868579|gb|EAT32804.1| AAEL014961-PA [Aedes aegypti]
Unigene5823_All	1.237506448	Up	PREDICTED: 1-phosphatidylinositol-4,5-bisphosphate phosphodiesterase gamma-1-like [Bombus

			impatiens]
Unigene12927_All	1.232034945	Up	putative DNA polymerase alpha catalytic subunit [Danaus plexippus]
Unigene15871_All	1.230751318	Up	PREDICTED: similar to alpha 1,2-mannosidase [Tribolium castaneum]
CL8412.Contig1_All	1.224576063	Up	PREDICTED: lambda-crystallin homolog [Nasonia vitripennis]
CL10030.Contig1_All	1.221156956	Up	pol-like protein [Biomphalaria glabrata]
Unigene47477_All	1.220104731	Up	PREDICTED: alpha-1,2-glucosyltransferase ALG10-A-like [Anolis carolinensis]
Unigene13098_All	1.215776031	Up	PREDICTED: lethal(2)neighbour of Tid protein-like [Bombus impatiens]
Unigene14921_All	1.215529763	Up	PREDICTED: similar to GA11371-PA [Tribolium castaneum]
Unigene22568_All	1.211186448	Up	PREDICTED: similar to CG12262 CG12262-PA [Tribolium castaneum] >gi|270003324|gb|EEZ99771.1| hypothetical protein TcasGA2_TC002547 [Tribolium castaneum]
Unigene15930_All	1.205590279	Up	hypothetical protein TcasGA2_TC001867 [Tribolium castaneum]
Unigene1935_All	1.202525158	Up	PREDICTED: ribonucleoside-diphosphate reductase subunit M2-like [Rattus norvegicus] >gi|392354916|ref|XP_003751887.1| PREDICTED: ribonucleoside-diphosphate reductase subunit M2-like [Rattus norvegicus]
CL9081.Contig1_All	1.196882638	Up	PREDICTED: cysteine dioxygenase type 1-like [Megachile rotundata]
Unigene5330_All	1.193549266	Up	PREDICTED: similar to AGAP010738-PA [Tribolium castaneum] >gi|270009591|gb|EFA06039.1| hypothetical protein TcasGA2_TC008869 [Tribolium castaneum]
CL3950.Contig1_All	1.189000797	Up	AGAP007780-PA [Anopheles gambiae str. PEST] >gi|116123450|gb|EAA12342.3| AGAP007780-PA [Anopheles gambiae str. PEST]
Unigene23375_All	1.186798033	Up	PREDICTED: 6-phosphofructokinase-like isoform 2 [Acyrthosiphon pisum]
CL9848.Contig2_All	1.175175109	Up	hypothetical protein TcasGA2_TC008439 [Tribolium castaneum]
Unigene66795_All	1.174558502	Up	branched chain amino-acid transaminase 1, cytosolic [Xenopus laevis] >gi|52139149|gb|AAH82673.1| LOC494683 protein [Xenopus laevis]
Unigene50196_All	1.172665788	Up	ubiquinol cytochrome c reductase subunit QCR8 [Argas monolakensis]
Unigene8085_All	1.165606352	Up	PREDICTED: similar to iPLA-2 [Tribolium castaneum] >gi|270005558|gb|EFA02006.1| hypothetical protein TcasGA2_TC007628 [Tribolium castaneum]
Unigene24018_All	1.16491045	Up	hypothetical protein DAPPUDRAFT_301077 [Daphnia pulex]
CL9536.Contig1_All	1.163393615	Up	PREDICTED: hypothetical protein LOC100570299 [Acyrthosiphon pisum]

Unigene13003_All	1.160135933	Up	hypothetical protein DAPPUDRAFT_302612 [Daphnia pulex]
CL8846.Contig2_All	1.158755518	Up	PREDICTED: similar to Rpb8 CG11246-PA [Tribolium castaneum] >gi|270009448|gb|EFA05896.1| hypothetical protein TcasGA2_TC008708 [Tribolium castaneum]
Unigene3352_All	1.154117803	Up	DNA primase [Aedes aegypti] >gi|108869184|gb|EAT33409.1| AAEL014314-PA [Aedes aegypti]
Unigene10464_All	1.147438292	Up	RNA polymerase II second largest subunit [Nomadacris japonica]
CL9969.Contig1_All	1.146837058	Up	pol-like protein [Biomphalaria glabrata]
Unigene16304_All	1.145480777	Up	malate dehydrogenase, putative [Pediculus humanus corporis] >gi|212508346|gb|EEB12070.1| malate dehydrogenase, putative [Pediculus humanus corporis]
Unigene10769_All	1.141234098	Up	PREDICTED: probable glutamyl-tRNA(Gln) amidotransferase subunit B, mitochondrial-like isoform 1 [Nasonia vitripennis] >gi|345484767|ref|XP_003425119.1| PREDICTED: probable glutamyl-tRNA(Gln) amidotransferase subunit B, mitochondrial-like isoform 2 [Nasonia vitripennis]
Unigene7490_All	1.141184349	Up	hypothetical protein DAPPUDRAFT_231350 [Daphnia pulex]
Unigene194_All	1.140568461	Up	PREDICTED: diacylglycerol O-acyltransferase 1-like [Megachile rotundata]
Unigene22548_All	1.129174055	Up	V-ATPase subunit D [Locusta migratoria]
Unigene2721_All	1.128035236	Up	PREDICTED: aminoacylase-1-like [Megachile rotundata]
CL5772.Contig3_All	1.126665656	Up	pro-phenoloxidase 1 [Locusta migratoria]
Unigene20141_All	1.1254937	Up	PREDICTED: similar to phospholipase C [Tribolium castaneum]
Unigene54219_All	1.118873959	Up	predicted protein [Nematostella vectensis] >gi|156227778|gb|EDO48580.1| predicted protein [Nematostella vectensis]
CL741.Contig2_All	1.117333371	Up	serine dehydratase-like [Xenopus (Silurana) tropicalis] >gi|56789083|gb|AAH88025.1| serine dehydratase-like [Xenopus (Silurana) tropicalis]
Unigene18774_All	1.103219303	Up	hypothetical protein TcasGA2_TC006611 [Tribolium castaneum]
Unigene23539_All	1.098346584	Up	hypothetical protein DAPPUDRAFT_59365 [Daphnia pulex]
Unigene59097_All	1.097384549	Up	beta-1,3-glucuronyltransferase s, p [Aedes aegypti] >gi|108879377|gb|EAT43602.1| AAEL004974-PB [Aedes aegypti]
CL5042.Contig1_All	1.0918814	Up	hexokinase [Locusta migratoria]
Unigene17372_All	1.089984042	Up	hypothetical protein TcasGA2_TC004842 [Tribolium castaneum]
Unigene18118_All	1.088377503	Up	glucose-6-phosphate 1-dehydrogenase [Culex quinquefasciatus] >gi|167864363|gb|EDS27746.1|

			glucose-6-phosphate 1-dehydrogenase [Culex quinquefasciatus]
CL290.Contig1_All	1.084414119	Up	hypothetical protein TcasGA2_TC006047 [Tribolium castaneum]
Unigene2157_All	1.07745218	Up	putative hemomucin [Schistocerca gregaria]
CL827.Contig1_All	1.072835995	Up	hypothetical protein BRAFLDRAFT_73226 [Branchiostoma floridae] >gi|229296749|gb|EEN67388.1| hypothetical protein BRAFLDRAFT_73226 [Branchiostoma floridae]
CL5638.Contig3_All	1.064804295	Up	xanthine dehydrogenase/oxidase [Culex quinquefasciatus] >gi|167876658|gb|EDS40041.1| xanthine dehydrogenase/oxidase [Culex quinquefasciatus]
Unigene21014_All	1.060824072	Up	hypothetical protein DAPPUDRAFT_313305 [Daphnia pulex]
CL3734.Contig1_All	1.060354264	Up	Conserved oligomeric Golgi complex subunit 5 [Harpegnathos saltator]
Unigene5793_All	1.057443555	Up	PREDICTED: similar to conserved hypothetical protein [Tribolium castaneum] >gi|270010834|gb|EFA07282.1| hypothetical protein TcasGA2_TC014517 [Tribolium castaneum]
CL5461.Contig2_All	1.054793563	Up	Pyruvate dehydrogenase E1 component subunit beta, mitochondrial [Camponotus floridanus]
Unigene22264_All	1.054674449	Up	PREDICTED: dolichyl pyrophosphate Man9GlcNAc2 alpha-1,3-glucosyltransferase-like [Nasonia vitripennis]
CL8150.Contig2_All	1.053524129	Up	Luciferin 4-monooxygenase [Acromyrmex echinatior]
Unigene23812_All	1.048142335	Up	PREDICTED: uracil phosphoribosyltransferase homolog [Nasonia vitripennis]
Unigene10666_All	1.046038774	Up	AGAP005124-PB [Anopheles gambiae str. PEST] >gi|157017071|gb|EDO64087.1| AGAP005124-PB [Anopheles gambiae str. PEST]
Unigene4083_All	1.044894435	Up	PREDICTED: GPI mannosyltransferase 2-like [Bombus impatiens]
Unigene15609_All	1.039068148	Up	PREDICTED: similar to oligosaccharyl transferase [Tribolium castaneum] >gi|270009866|gb|EFA06314.1| hypothetical protein TcasGA2_TC009183 [Tribolium castaneum]
Unigene20734_All	1.038920728	Up	PREDICTED: glycogen debranching enzyme [Megachile rotundata]
Unigene21059_All	1.038653026	Up	PREDICTED: porphobilinogen deaminase-like [Apis mellifera]
Unigene22607_All	1.034066872	Up	GF11142 [Drosophila ananassae] >gi|190622454|gb|EDV37978.1| GF11142 [Drosophila ananassae]
Unigene15872_All	1.030055395	Up	nadp transhydrogenase [Aedes aegypti] >gi|108870974|gb|EAT35199.1| AAEL012616-PA [Aedes aegypti]

<table>
<tr><td>Unigene8645_All</td><td>1.029293102</td><td>Up</td><td>PREDICTED: 2-oxoglutarate dehydrogenase, mitochondrial-like isoform 1 [Apis mellifera]</td></tr>
<tr><td>CL432.Contig2_All</td><td>1.028397336</td><td>Up</td><td>PREDICTED: V-type proton ATPase 116 kDa subunit a isoform 1-like isoform 1 [Bombus impatiens]</td></tr>
<tr><td>CL6259.Contig2_All</td><td>1.026268869</td><td>Up</td><td>ATP synthase lipid-binding protein, mitochondrial [Harpegnathos saltator]</td></tr>
<tr><td>Unigene312_All</td><td>1.026135607</td><td>Up</td><td>phospholipase C delta, putative [Ixodes scapularis] >gi|215503614|gb|EEC13108.1| phospholipase C delta, putative [Ixodes scapularis]</td></tr>
<tr><td>CL4882.Contig2_All</td><td>1.022843877</td><td>Up</td><td>Heat shock protein 67B2, putative [Pediculus humanus corporis] >gi|212513684|gb|EEB16164.1| Heat shock protein 67B2, putative [Pediculus humanus corporis]</td></tr>
<tr><td>CL294.Contig2_All</td><td>1.018193886</td><td>Up</td><td>PREDICTED: alanine aminotransferase 2-like [Acyrthosiphon pisum]</td></tr>
<tr><td>Unigene8664_All</td><td>1.015100716</td><td>Up</td><td>PREDICTED: similar to CG1814 CG1814-PA [Tribolium castaneum]</td></tr>
<tr><td>CL4641.Contig2_All</td><td>1.005308259</td><td>Up</td><td>hypothetical protein DAPPUDRAFT_308242 [Daphnia pulex]</td></tr>
<tr><td>Unigene15342_All</td><td>1.004337534</td><td>Up</td><td>NADH dehydrogenase (ubiquinone) Fe-S protein 4 [Tribolium castaneum]</td></tr>
<tr><td>Unigene7920_All</td><td>1.001116572</td><td>Up</td><td>PREDICTED: similar to AGAP005845-PA [Tribolium castaneum]</td></tr>
<tr><td>CL3734.Contig3_All</td><td>1.00047694</td><td>Up</td><td>Conserved oligomeric Golgi complex subunit 5 [Harpegnathos saltator]</td></tr>
<tr><td>CL9670.Contig2_All</td><td>-1.0130188</td><td>Down</td><td>hypothetical protein DAPPUDRAFT_50786 [Daphnia pulex]</td></tr>
<tr><td>CL3950.Contig2_All</td><td>-1.01347142</td><td>Down</td><td>AGAP007780-PA [Anopheles gambiae str. PEST] >gi|116123450|gb|EAA12342.3| AGAP007780-PA [Anopheles gambiae str. PEST]</td></tr>
<tr><td>CL333.Contig1_All</td><td>-1.02044996</td><td>Down</td><td>PREDICTED: RNA-directed DNA polymerase from mobile element jockey-like [Metaseiulus occidentalis]</td></tr>
<tr><td>Unigene22689_All</td><td>-1.02259647</td><td>Down</td><td>NADH:ubiquinone dehydrogenase [Culex quinquefasciatus] >gi|167877521|gb|EDS40904.1| NADH:ubiquinone dehydrogenase [Culex quinquefasciatus]</td></tr>
<tr><td>Unigene5299_All</td><td>-1.02984664</td><td>Down</td><td>pol polyprotein - Tribolium freemani retrotransposon Woot (fragment) >gi|1117950|gb|AAB38069.1| polyprotein [Tribolium freemani]</td></tr>
<tr><td>Unigene20708_All</td><td>-1.03076225</td><td>Down</td><td>PREDICTED: similar to reverse transcriptase homolog [Tribolium castaneum] >gi|270017183|gb|EFA13629.1| hypothetical protein TcasGA2_TC005274 [Tribolium castaneum]</td></tr>
<tr><td>CL426.Contig1_All</td><td>-1.03468841</td><td>Down</td><td>Cytosolic purine 5'-nucleotidase [Harpegnathos saltator]</td></tr>
<tr><td>CL932.Contig8_All</td><td>-1.04735607</td><td>Down</td><td>DNA primase large subunit [Camponotus floridanus]</td></tr>
<tr><td>Unigene2151_All</td><td>-1.05514836</td><td>Down</td><td>NADH dehydrogenase subunit 3 [Homo sapiens] >gi|60257568|gb|AAX15479.1| NADH dehydrogenase</td></tr>
</table>

			subunit 3 [Homo sapiens]
CL3060.Contig1_All	-1.05671615	Down	PREDICTED: similar to CG13760 CG13760-PB [Tribolium castaneum] >gi|270008923|gb|EFA05371.1| hypothetical protein TcasGA2_TC015537 [Tribolium castaneum]
Unigene9752_All	-1.06417614	Down	aldo-keto reductase, partial [Schistocerca gregaria]
Unigene18157_All	-1.07761124	Down	hypothetical protein BRAFLDRAFT_104160 [Branchiostoma floridae] >gi|229299104|gb|EEN69724.1| hypothetical protein BRAFLDRAFT_104160 [Branchiostoma floridae]
Unigene20471_All	-1.07922955	Down	--
CL6572.Contig1_All	-1.08173741	Down	NADH dehydrogenase subunit 1 [Locusta migratoria manilensis]
CL7874.Contig1_All	-1.08651392	Down	hypothetical protein TcasGA2_TC005208 [Tribolium castaneum]
Unigene9649_All	-1.08742729	Down	UDP-glucose pyrophosphorylase [Spodoptera exigua]
Unigene8527_All	-1.08878119	Down	GL16175 [Drosophila persimilis] >gi|194111018|gb|EDW33061.1| GL16175 [Drosophila persimilis]
Unigene4343_All	-1.09011509	Down	hypothetical protein TcasGA2_TC015470 [Tribolium castaneum]
Unigene3277_All	-1.09188382	Down	PREDICTED: adenosine deaminase [Apis mellifera]
Unigene4410_All	-1.10898956	Down	PREDICTED: alcohol dehydrogenase class-3-like [Nasonia vitripennis]
Unigene23240_All	-1.11977595	Down	transposable element Tc3 transposase [Clonorchis sinensis]
Unigene19803_All	-1.1316416	Down	hypothetical protein DAPPUDRAFT_333266 [Daphnia pulex]
CL4655.Contig1_All	-1.13478658	Down	hypothetical protein DAPPUDRAFT_196566 [Daphnia pulex]
Unigene1621_All	-1.14132753	Down	hypothetical protein TcasGA2_TC001995 [Tribolium castaneum]
CL8815.Contig1_All	-1.14766002	Down	cytochrome c oxidase subunit III [Homo sapiens] >gi|356466353|gb|AET08989.1| cytochrome c oxidase subunit III (mitochondrion) [Homo sapiens] >gi|356466395|gb|AET09028.1| cytochrome c oxidase subunit III (mitochondrion) [Homo sapiens] >gi|356466507|gb|AET09132.1| cytochrome c oxidase subunit III (mitochondrion) [Homo sapiens] >gi|356466563|gb|AET09184.1| cytochrome c oxidase subunit III (mitochondrion) [Homo sapiens] >gi|356466703|gb|AET09314.1| cytochrome c oxidase subunit III (mitochondrion) [Homo sapiens] >gi|356466717|gb|AET09327.1| cytochrome c oxidase subunit III (mitochondrion) [Homo sapiens] >gi|356466801|gb|AET09405.1| cytochrome c oxidase subunit III (mitochondrion) [Homo sapiens] >gi|356466815|gb|AET09418.1| cytochrome c oxidase subunit III (mitochondrion) [Homo sapiens] >gi|356466829|gb|AET09431.1| cytochrome c oxidase subunit III (mitochondrion) [Homo sapiens] >gi|356467025|gb|AET09613.1| cytochrome c oxidase

			subunit III (mitochondrion) [Homo sapiens]
CL2685.Contig2_All	-1.15141237	Down	PREDICTED: uncharacterized protein LOC101235092 [Hydra magnipapillata]
CL3110.Contig1_All	-1.16011387	Down	glucosamine-6-phosphate isomerase [Culex quinquefasciatus] >gi|167866713|gb|EDS30096.1| glucosamine-6-phosphate isomerase [Culex quinquefasciatus]
Unigene16201_All	-1.19365832	Down	triosephosphate isomerase [Blattella germanica]
Unigene20768_All	-1.1954169	Down	PREDICTED: lipase member H-like [Acyrthosiphon pisum]
Unigene8316_All	-1.19541896	Down	hypothetical protein TcasGA2_TC015470 [Tribolium castaneum]
Unigene25253_All	-1.19781236	Down	PREDICTED: pyruvate dehydrogenase phosphatase regulatory subunit, mitochondrial-like [Megachile rotundata]
CL426.Contig2_All	-1.2026169	Down	PREDICTED: cytosolic purine 5'-nucleotidase-like isoform 2 [Bombus terrestris]
CL1765.Contig2_All	-1.20304455	Down	aminopeptidase N [Sitophilus oryzae]
Unigene19612_All	-1.20307104	Down	GI12135 [Drosophila mojavensis] >gi|193919481|gb|EDW18348.1| GI12135 [Drosophila mojavensis]
Unigene1046_All	-1.20907195	Down	PREDICTED: similar to fumarylacetoacetate hydrolase [Tribolium castaneum] >gi|270009995|gb|EFA06443.1| hypothetical protein TcasGA2_TC009325 [Tribolium castaneum]
CL1016.Contig1_All	-1.21890576	Down	glucosyl glucuronosyl transferases [Locusta migratoria]
CL4186.Contig2_All	-1.2283426	Down	PREDICTED: regucalcin-like [Megachile rotundata]
Unigene1927_All	-1.23219968	Down	tyrosine hydroxylase long variant, partial [Gryllus bimaculatus]
CL3035.Contig1_All	-1.23638005	Down	endonuclease-reverse transcriptase [Bombyx mori]
Unigene17312_All	-1.23951324	Down	hypothetical protein TcasGA2_TC001491 [Tribolium castaneum]
Unigene9776_All	-1.24127061	Down	cytochrome c oxidase subunit I [Homo sapiens]
CL9405.Contig1_All	-1.24475556	Down	endonuclease/reverse transcriptase [Branchiostoma floridae]
Unigene12285_All	-1.24616789	Down	unknown [Dendroctonus ponderosae]
Unigene25356_All	-1.25509721	Down	Diacylglycerol kinase epsilon, putative [Pediculus humanus corporis] >gi|212512843|gb|EEB15531.1| Diacylglycerol kinase epsilon, putative [Pediculus humanus corporis]
CL1357.Contig11_All	-1.2557048	Down	conserved hypothetical protein [Culex quinquefasciatus] >gi|167871903|gb|EDS35286.1| conserved hypothetical protein [Culex quinquefasciatus]
Unigene4448_All	-1.25744409	Down	pro-phenoloxidase 1 [Locusta migratoria]

Unigene4421_All	-1.26687004	Down	NADH dehydrogenase subunit 5 (mitochondrion) [Homo sapiens]
Unigene6797_All	-1.26704627	Down	aliphatic nitrilase, putative [Papilio xuthus]
CL8494.Contig3_All	-1.26832396	Down	PREDICTED: kynurenine--oxoglutarate transaminase 3-like [Megachile rotundata]
Unigene7097_All	-1.2687516	Down	hypothetical protein BRAFLDRAFT_114917 [Branchiostoma floridae] >gi|229286915|gb|EEN57628.1| hypothetical protein BRAFLDRAFT_114917 [Branchiostoma floridae]
Unigene22816_All	-1.27278835	Down	DNA-directed RNA polymerases III 12.5 kDa polypeptide, putative [Pediculus humanus corporis] >gi|212510537|gb|EEB13694.1| DNA-directed RNA polymerases III 12.5 kDa polypeptide, putative [Pediculus humanus corporis]
Unigene151_All	-1.28324633	Down	PREDICTED: similar to glucosyl/glucuronosyl transferases [Tribolium castaneum]
CL9670.Contig1_All	-1.284887	Down	hypothetical protein DAPPUDRAFT_50786 [Daphnia pulex]
Unigene48130_All	-1.2901579	Down	Putative octanoyltransferase, mitochondrial [Acromyrmex echinatior]
Unigene11944_All	-1.30171509	Down	cytochrome c oxidase subunit II (mitochondrion) [Homo sapiens]
CL4356.Contig3_All	-1.30342863	Down	lipoic acid synthetase, putative [Pediculus humanus corporis] >gi|212510482|gb|EEB13657.1| lipoic acid synthetase, putative [Pediculus humanus corporis]
CL9009.Contig1_All	-1.31119322	Down	PREDICTED: RNA-directed DNA polymerase from mobile element jockey-like [Metaseiulus occidentalis]
Unigene10966_All	-1.31189639	Down	AGAP001257-PB [Anopheles gambiae str. PEST] >gi|333470437|gb|EGK97623.1| AGAP001257-PB [Anopheles gambiae str. PEST]
Unigene26513_All	-1.32183296	Down	sphingomyelin phosphodiesterase, putative [Pediculus humanus corporis] >gi|212514220|gb|EEB16579.1| sphingomyelin phosphodiesterase, putative [Pediculus humanus corporis]
Unigene16154_All	-1.3255549	Down	Bifunctional ATP-dependent dihydroxyacetone kinase/FAD-AMP lyase (cyclizing) [Acromyrmex echinatior]
CL28.Contig8_All	-1.32787123	Down	hypothetical protein TcasGA2_TC002592 [Tribolium castaneum]
CL7324.Contig2_All	-1.33147401	Down	PREDICTED: similar to GA13505-PA [Tribolium castaneum] >gi|270011394|gb|EFA07842.1| hypothetical protein TcasGA2_TC005412 [Tribolium castaneum]
Unigene7109_All	-1.33521026	Down	cytochrome c oxidase subunit I [Homo sapiens]
Unigene7154_All	-1.34150011	Down	pro-phenoloxidase 1 [Locusta migratoria]
Unigene14769_All	-1.35987172	Down	NADH dehydrogenase subunit 4 (mitochondrion) [Homo sapiens]

Unigene12903_All	-1.36275117	Down	PREDICTED: RNA-directed DNA polymerase from mobile element jockey-like [Hydra magnipapillata]
CL367.Contig10_All	-1.36395875	Down	mitochondrial ribosomal protein VAR1, putative [Pediculus humanus corporis] >gi|212514798|gb|EEB17048.1| mitochondrial ribosomal protein VAR1, putative [Pediculus humanus corporis]
Unigene8665_All	-1.37556482	Down	glucosyl/glucuronosyl transferases [Aedes aegypti] >gi|108881419|gb|EAT45644.1| AAEL003099-PA [Aedes aegypti]
Unigene12074_All	-1.37635869	Down	hypothetical protein SINV_10710 [Solenopsis invicta]
CL5587.Contig2_All	-1.37662224	Down	hypothetical protein KGM_07467 [Danaus plexippus]
Unigene5453_All	-1.37944873	Down	PREDICTED: beta,beta-carotene 9',10'-oxygenase-like [Nasonia vitripennis]
CL7907.Contig2_All	-1.38236027	Down	PREDICTED: polypeptide N-acetylgalactosaminyltransferase 3-like isoform 1 [Acyrthosiphon pisum] >gi|328723400|ref|XP_003247833.1| PREDICTED: polypeptide N-acetylgalactosaminyltransferase 3-like isoform 2 [Acyrthosiphon pisum]
Unigene22706_All	-1.38458793	Down	NADH dehydrogenase subunit 2 [Homo sapiens] >gi|151328528|gb|ABR93752.1| NADH dehydrogenase subunit 2 [Homo sapiens] >gi|156455639|gb|ABU64833.1| NADH dehydrogenase subunit 2 [Homo sapiens] >gi|213492506|gb|ACJ47274.1| NADH dehydrogenase subunit 2 [Homo sapiens] >gi|359467485|gb|AEV47176.1| NADH dehydrogenase subunit 2 (mitochondrion) [Homo sapiens] >gi|359470747|gb|AEV50205.1| NADH dehydrogenase subunit 2 (mitochondrion) [Homo sapiens]
Unigene16006_All	-1.38496828	Down	hypothetical protein EAG_05880 [Camponotus floridanus]
CL1827.Contig1_All	-1.3943723	Down	PREDICTED: peroxidase-like [Megachile rotundata]
Unigene17105_All	-1.39862191	Down	hypothetical protein SINV_05070 [Solenopsis invicta]
Unigene2912_All	-1.40210903	Down	PREDICTED: farnesyl pyrophosphate synthase-like [Nasonia vitripennis]
Unigene15594_All	-1.41067959	Down	hypothetical protein TcasGA2_TC002592 [Tribolium castaneum]
Unigene22664_All	-1.41505981	Down	Molybdenum cofactor synthesis protein 2 large subunit [Harpegnathos saltator]
Unigene25534_All	-1.42272988	Down	PREDICTED: putative glycerol kinase 3-like [Nasonia vitripennis]
Unigene24050_All	-1.42385277	Down	PREDICTED: inositol-trisphosphate 3-kinase B-like isoform 2 [Acyrthosiphon pisum]
Unigene21034_All	-1.44106834	Down	Beta-galactosidase [Camponotus floridanus]
Unigene5325_All	-1.44403704	Down	AGAP009284-PA [Anopheles gambiae str. PEST] >gi|157013832|gb|EAA14953.3| AGAP009284-PA

			[Anopheles gambiae str. PEST]
Unigene19725_All	-1.44404884	Down	NADP-dependent malic enzyme-like protein [Locusta migratoria]
Unigene4573_All	-1.44418142	Down	PREDICTED: similar to glucosyl/glucuronosyl transferases [Tribolium castaneum]
CL9597.Contig1_All	-1.44700224	Down	PREDICTED: dolichyl-diphosphooligosaccharide--protein glycosyltransferase 48 kDa subunit-like [Nasonia vitripennis]
CL5757.Contig1_All	-1.45295299	Down	PREDICTED: c-1-tetrahydrofolate synthase, cytoplasmic-like [Megachile rotundata]
Unigene3070_All	-1.45761928	Down	farnesyl diphosphate synthase [Mythimna unipuncta]
Unigene13636_All	-1.4613488	Down	abhydrolase domain-containing protein 7 [Culex quinquefasciatus] >gi|167866540|gb|EDS29923.1| abhydrolase domain-containing protein 7 [Culex quinquefasciatus]
Unigene16647_All	-1.46385795	Down	Beta-galactosidase [Acromyrmex echinatior]
Unigene17924_All	-1.4679158	Down	PREDICTED: uncharacterized protein LOC100889850 [Strongylocentrotus purpuratus]
Unigene12576_All	-1.48257554	Down	PREDICTED: GPI inositol-deacylase-like [Acyrthosiphon pisum]
Unigene20369_All	-1.48760964	Down	--
CL9420.Contig2_All	-1.48827275	Down	PREDICTED: transketolase-like protein 2-like isoform 1 [Bombus impatiens]
Unigene1961_All	-1.48960802	Down	PREDICTED: hypothetical protein LOC100746638 [Bombus impatiens]
Unigene5227_All	-1.50610272	Down	PREDICTED: dihydropyrimidinase-like isoform 2 [Bombus impatiens]
CL2784.Contig1_All	-1.50620006	Down	Choline/ethanolamine kinase [Harpegnathos saltator]
Unigene14309_All	-1.52521324	Down	PREDICTED: hypothetical protein LOC100568495 [Acyrthosiphon pisum]
Unigene21218_All	-1.52800378	Down	PREDICTED: RNA-directed DNA polymerase from mobile element jockey-like [Strongylocentrotus purpuratus]
Unigene22852_All	-1.53032563	Down	GJ11980 [Drosophila virilis] >gi|194154506|gb|EDW69690.1| GJ11980 [Drosophila virilis]
CL3952.Contig2_All	-1.55796378	Down	PREDICTED: LOW QUALITY PROTEIN: guanylate kinase-like [Megachile rotundata]
CL3952.Contig1_All	-1.56377366	Down	PREDICTED: LOW QUALITY PROTEIN: guanylate kinase-like [Megachile rotundata]
Unigene4191_All	-1.57201162	Down	gpi inositol deacylase pgap1, putative [Pediculus humanus corporis] >gi|212509438|gb|EEB12821.1| gpi inositol deacylase pgap1, putative [Pediculus humanus corporis]
Unigene22620_All	-1.57402326	Down	lipoate protein ligase [Phyllotreta striolata]
Unigene24005_All	-1.57737824	Down	hypothetical protein DAPPUDRAFT_107456 [Daphnia pulex] >gi|321464868|gb|EFX75873.1| hypothetical protein DAPPUDRAFT_107527 [Daphnia pulex]

CL6764.Contig1_All	-1.57872977	Down	S-adenosylmethionine synthetase [Acromyrmex echinatior]
Unigene7934_All	-1.58086958	Down	PREDICTED: hypothetical protein LOC100634292 [Amphimedon queenslandica]
CL7907.Contig1_All	-1.58311157	Down	PREDICTED: polypeptide N-acetylgalactosaminyltransferase 3-like isoform 1 [Acyrthosiphon pisum] >gi|328723400|ref|XP_003247833.1| PREDICTED: polypeptide N-acetylgalactosaminyltransferase 3-like isoform 2 [Acyrthosiphon pisum]
Unigene18594_All	-1.59571856	Down	hypothetical protein SINV_01371 [Solenopsis invicta]
CL5053.Contig2_All	-1.60236912	Down	juvenile hormone esterase-like protein Est1 [Reticulitermes flavipes]
CL4155.Contig2_All	-1.62190869	Down	hypothetical protein TcasGA2_TC012513 [Tribolium castaneum]
Unigene173_All	-1.63907007	Down	24-dehydrocholesterol reductase [Daphnia pulex]
CL10.Contig2_All	-1.64176591	Down	endonuclease-reverse transcriptase [Bombyx mori]
CL6848.Contig2_All	-1.64368386	Down	glutamine synthetase [Schistocerca gregaria]
CL199.Contig2_All	-1.65477728	Down	PREDICTED: RNA-directed DNA polymerase from mobile element jockey-like [Strongylocentrotus purpuratus]
Unigene13297_All	-1.66128661	Down	hypothetical protein TcasGA2_TC009115 [Tribolium castaneum]
Unigene21093_All	-1.66561464	Down	hypothetical protein TcasGA2_TC001995 [Tribolium castaneum]
Unigene16018_All	-1.68024557	Down	hypothetical protein TcasGA2_TC000817 [Tribolium castaneum]
Unigene12018_All	-1.68055963	Down	beta-glucosidase [Nasutitermes takasagoensis]
Unigene23097_All	-1.69065327	Down	PREDICTED: prolyl 4-hydroxylase subunit alpha-2-like [Bombus impatiens]
Unigene24439_All	-1.6965804	Down	--
Unigene7066_All	-1.70470281	Down	PREDICTED: DNA-directed RNA polymerases I and III subunit RPAC1-like [Bombus impatiens]
CL6138.Contig2_All	-1.7098903	Down	PREDICTED: similar to AGAP008016-PA [Tribolium castaneum] >gi|270013121|gb|EFA09569.1| hypothetical protein TcasGA2_TC011683 [Tribolium castaneum]
CL1858.Contig2_All	-1.70992702	Down	unknown [Dendroctonus ponderosae]
Unigene8442_All	-1.71148927	Down	Uncharacterized family 31 glucosidase KIAA1161 [Camponotus floridanus]
Unigene11112_All	-1.72915088	Down	PREDICTED: UDP-glucuronosyltransferase 2B14-like [Acyrthosiphon pisum]
Unigene6804_All	-1.73918698	Down	D-3-phosphoglycerate dehydrogenase, putative [Pediculus humanus corporis] >gi|212505884|gb|EEB10230.1| D-3-phosphoglycerate dehydrogenase, putative [Pediculus humanus corporis]

CL4186.Contig5_All	-1.73928563	Down	PREDICTED: similar to luciferin-regenerating enzyme [Tribolium castaneum] >gi|270006690|gb|EFA03138.1| hypothetical protein TcasGA2_TC013050 [Tribolium castaneum]
CL6818.Contig2_All	-1.74483346	Down	unknown [Blattella germanica]
Unigene74439_All	-1.74650486	Down	cytochrome c oxidase subunit II [Locusta migratoria] >gi|117024|sp|P14573.1|COX2_LOCMI RecName: Full=Cytochrome c oxidase subunit 2; AltName: Full=Cytochrome c oxidase polypeptide II >gi|1182026|emb|CAA56539.1| COII [Locusta migratoria]
CL5269.Contig2_All	-1.75837905	Down	PREDICTED: threonine dehydratase catabolic-like isoform 2 [Bombus terrestris]
Unigene26283_All	-1.7606005	Down	GD20337 [Drosophila simulans] >gi|194199220|gb|EDX12796.1| GD20337 [Drosophila simulans]
CL9420.Contig1_All	-1.76451154	Down	PREDICTED: transketolase-like protein 2-like isoform 1 [Bombus impatiens]
Unigene20706_All	-1.77483521	Down	Transposable element Tcb1 transposase [Harpegnathos saltator]
CL9976.Contig1_All	-1.77709077	Down	PREDICTED: uncharacterized protein LOC100889850 [Strongylocentrotus purpuratus]
Unigene16199_All	-1.78110599	Down	PREDICTED: RNA-directed DNA polymerase from mobile element jockey-like [Hydra magnipapillata]
Unigene11180_All	-1.78857155	Down	hypothetical protein Pmar_PMAR012004 [Perkinsus marinus ATCC 50983] >gi|239867036|gb|EEQ98996.1| hypothetical protein Pmar_PMAR012004 [Perkinsus marinus ATCC 50983]
Unigene8233_All	-1.79637689	Down	carboxylesterase ae17 [Bombyx mori]
Unigene38164_All	-1.81299092	Down	unnamed protein product [Mus musculus]
Unigene23123_All	-1.82541691	Down	Cysteine sulfinic acid decarboxylase, putative [Pediculus humanus corporis] >gi|212512559|gb|EEB15302.1| Cysteine sulfinic acid decarboxylase, putative [Pediculus humanus corporis]
CL8915.Contig1_All	-1.82562611	Down	PREDICTED: similar to nicotinamide mononucleotide adenylyltransferase 1 [Tribolium castaneum] >gi|270013679|gb|EFA10127.1| hypothetical protein TcasGA2_TC012307 [Tribolium castaneum]
Unigene17095_All	-1.82978445	Down	cytochrome P450 9E1 [Diploptera punctata]
Unigene20067_All	-1.84711181	Down	Alkaline phosphatase 4 [Camponotus floridanus]
Unigene9542_All	-1.86582491	Down	sulfatase [Papilio xuthus]
CL4534.Contig1_All	-1.87062403	Down	PREDICTED: retinal dehydrogenase 1-like [Megachile rotundata]

CL3826.Contig5_All	-1.87426424	Down	2-oxoisovalerate dehydrogenase subunit beta, mitochondrial [Culex quinquefasciatus] >gi|167877436|gb|EDS40819.1| 2-oxoisovalerate dehydrogenase subunit beta, mitochondrial [Culex quinquefasciatus]
Unigene24115_All	-1.87817193	Down	juvenile hormone esterase-like protein Est1 [Reticulitermes flavipes]
CL721.Contig1_All	-1.88310028	Down	PREDICTED: pancreatic lipase-related protein 2-like [Bombus terrestris]
CL1792.Contig1_All	-1.88445577	Down	PREDICTED: similar to ribitol kinase [Tribolium castaneum] >gi|270013302|gb|EFA09750.1| hypothetical protein TcasGA2_TC011889 [Tribolium castaneum]
Unigene9377_All	-1.91295768	Down	ovarian fibroin-like substance-2 [Cyprinus carpio]
CL2791.Contig3_All	-1.92546013	Down	fructose 1,6-bisphosphate aldolase [Schistocerca gregaria]
CL890.Contig2_All	-1.93514739	Down	endonuclease-reverse transcriptase [Bombyx mori]
Unigene14277_All	-1.94003484	Down	D-3-phosphoglycerate dehydrogenase [Acromyrmex echinatior]
CL6856.Contig1_All	-1.94155511	Down	PREDICTED: lipid phosphate phosphohydrolase 1-like isoform 1 [Bombus terrestris]
Unigene16050_All	-1.94882098	Down	PREDICTED: hypothetical protein LOC100739562, partial [Sus scrofa]
CL8846.Contig4_All	-1.95670432	Down	hypothetical protein KGM_12645 [Danaus plexippus]
CL6373.Contig1_All	-1.95697553	Down	hypothetical protein TcasGA2_TC004732 [Tribolium castaneum]
CL5042.Contig2_All	-1.97916504	Down	hexokinase [Locusta migratoria]
CL2887.Contig2_All	-1.99000697	Down	cytochrome c oxidase subunit I, partial (mitochondrion) [Locusta migratoria]
CL8915.Contig2_All	-1.99466257	Down	PREDICTED: similar to nicotinamide mononucleotide adenylyltransferase 1 [Tribolium castaneum] >gi|270013679|gb|EFA10127.1| hypothetical protein TcasGA2_TC012307 [Tribolium castaneum]
CL3794.Contig2_All	-1.99771115	Down	proline oxidase, putative [Pediculus humanus corporis] >gi|212511759|gb|EEB14647.1| proline oxidase, putative [Pediculus humanus corporis]
CL4563.Contig2_All	-2.0126107	Down	PREDICTED: RNA-directed DNA polymerase from mobile element jockey-like [Strongylocentrotus purpuratus]
Unigene17454_All	-2.01581589	Down	hypothetical protein TcasGA2_TC007912 [Tribolium castaneum]
Unigene21748_All	-2.02324217	Down	PREDICTED: GPI ethanolamine phosphate transferase 1-like [Xenopus (Silurana) tropicalis]
CL10032.Contig1_All	-2.03004538	Down	transposable element tc1 transposase, putative [Ixodes scapularis] >gi|215508650|gb|EEC18104.1| transposable element tc1 transposase, putative [Ixodes scapularis]

Unigene39414_All	-2.03193495	Down	PREDICTED: similar to GA13505-PA [Tribolium castaneum] >gi|270011394|gb|EFA07842.1| hypothetical protein TcasGA2_TC005412 [Tribolium castaneum]
CL179.Contig1_All	-2.03904358	Down	glutathione S-transferase sigma 1 [Locusta migratoria]
Unigene1455_All	-2.04562091	Down	GF22904 [Drosophila ananassae] >gi|190614984|gb|EDV30508.1| GF22904 [Drosophila ananassae]
Unigene7298_All	-2.04861498	Down	glutathione S-transferase sigma 1 [Locusta migratoria]
Unigene9217_All	-2.07720599	Down	aldo-keto reductase, partial [Schistocerca gregaria]
Unigene8582_All	-2.08657453	Down	GJ14680 [Drosophila virilis] >gi|194147651|gb|EDW63349.1| GJ14680 [Drosophila virilis]
CL2626.Contig2_All	-2.11524455	Down	hypothetical protein TcasGA2_TC012513 [Tribolium castaneum]
CL2489.Contig1_All	-2.11605649	Down	TPA: endonuclease-reverse transcriptase [Schistosoma mansoni]
Unigene14762_All	-2.12503132	Down	PREDICTED: similar to fumarylacetoacetate hydrolase [Tribolium castaneum] >gi|270009995|gb|EFA06443.1| hypothetical protein TcasGA2_TC009325 [Tribolium castaneum]
CL9855.Contig1_All	-2.12945048	Down	PREDICTED: neutral ceramidase-like isoform 2 [Nasonia vitripennis]
CL2052.Contig2_All	-2.13281822	Down	NADH dehydrogenase subunit 5 [Locusta migratoria manilensis] >gi|283825405|gb|ADB43159.1| NADH dehydrogenase subunit 5 [Locusta migratoria manilensis]
Unigene4326_All	-2.13593947	Down	hypothetical protein TcasGA2_TC004344 [Tribolium castaneum]
Unigene7872_All	-2.13742977	Down	hypothetical protein TcasGA2_TC005208 [Tribolium castaneum]
Unigene24736_All	-2.15055785	Down	Beta-galactosidase [Camponotus floridanus]
CL5378.Contig1_All	-2.1835296	Down	hypothetical protein TcasGA2_TC006160 [Tribolium castaneum]
Unigene39522_All	-2.1838464	Down	GK11429 [Drosophila willistoni] >gi|194165793|gb|EDW80694.1| GK11429 [Drosophila willistoni]
Unigene20109_All	-2.20551937	Down	PREDICTED: prolyl 4-hydroxylase subunit alpha-2-like [Apis florea]
CL6709.Contig2_All	-2.2246771	Down	7-dehydrocholesterol reductase [Tupaia chinensis]
CL4084.Contig2_All	-2.23197553	Down	--
Unigene10471_All	-2.23851904	Down	methionine-rich hexamerin-like protein 1 [Romalea microptera]
CL2858.Contig2_All	-2.26035383	Down	AAEL007486-PA, partial [Aedes aegypti]
Unigene38205_All	-2.29489228	Down	reverse transcriptase, partial [Drepana sp. GAS-2011]
Unigene31880_All	-2.31761236	Down	RNA polymerase II largest subunit [Inocellia japonica]
CL7481.Contig1_All	-2.32377475	Down	PREDICTED: RNA-directed DNA polymerase from mobile element jockey-like [Strongylocentrotus

			purpuratus]
CL4230.Contig1_All	-2.33017256	Down	RecName: Full=RNA-directed DNA polymerase from mobile element jockey; AltName: Full=Reverse transcriptase
CL2858.Contig4_All	-2.34760204	Down	AAEL007486-PA, partial [Aedes aegypti]
Unigene8262_All	-2.36607647	Down	PREDICTED: RNA-directed DNA polymerase from mobile element jockey-like [Metaseiulus occidentalis]
Unigene34541_All	-2.38137542	Down	feruloyl esterase-like protein Est2 [Reticulitermes flavipes]
Unigene5754_All	-2.38240398	Down	hypothetical protein TcasGA2_TC009065 [Tribolium castaneum]
Unigene1711_All	-2.38466385	Down	glycine dehydrogenase [Aedes aegypti] >gi|108869068|gb|EAT33293.1| AAEL014426-PA [Aedes aegypti]
Unigene14734_All	-2.38732487	Down	PREDICTED: similar to N-acetyllactosaminide beta-1,3-N-acetylglucosaminyltransferase, putative [Tribolium castaneum] >gi|270005935|gb|EFA02383.1| hypothetical protein TcasGA2_TC008060 [Tribolium castaneum]
Unigene13248_All	-2.38956466	Down	fructose 1,6-bisphosphate aldolase [Schistocerca gregaria]
CL962.Contig1_All	-2.39362573	Down	seminal fluid protein HACP047 [Heliconius erato]
CL3622.Contig2_All	-2.39804782	Down	endonuclease/reverse transcriptase [Branchiostoma floridae]
Unigene15526_All	-2.40113054	Down	otopetrin [Danaus plexippus]
CL119.Contig3_All	-2.40474427	Down	PREDICTED: similar to acyloxyacyl hydrolase [Tribolium castaneum] >gi|270002377|gb|EEZ98824.1| hypothetical protein TcasGA2_TC004431 [Tribolium castaneum]
Unigene10303_All	-2.41359985	Down	hypothetical protein DAPPUDRAFT_309035 [Daphnia pulex]
Unigene13680_All	-2.41425036	Down	Probable phosphoserine aminotransferase [Harpegnathos saltator]
CL6572.Contig2_All	-2.44692089	Down	NADH dehydrogenase subunit 1 (mitochondrion) [Locusta migratoria]
CL6444.Contig2_All	-2.44726476	Down	PREDICTED: uncharacterized protein LOC100202983 [Hydra magnipapillata]
CL439.Contig2_All	-2.44995629	Down	hypothetical protein DAPPUDRAFT_306482 [Daphnia pulex]
Unigene15419_All	-2.45620121	Down	5'-nucleotidase [Camponotus floridanus]
CL1583.Contig2_All	-2.4667898	Down	PREDICTED: gamma-interferon-inducible lysosomal thiol reductase-like [Acyrthosiphon pisum]
Unigene17816_All	-2.4786994	Down	AGAP010159-PA [Anopheles gambiae str. PEST] >gi|116118465|gb|EAA13891.3| AGAP010159-PA [Anopheles gambiae str. PEST]

CL6444.Contig1_All	-2.48621286	Down	PREDICTED: uncharacterized protein LOC100202983 [Hydra magnipapillata]
CL4085.Contig2_All	-2.49405194	Down	PREDICTED: similar to alpha methylacyl-coa racemase [Tribolium castaneum] >gi|270007738|gb|EFA04186.1| hypothetical protein TcasGA2_TC014435 [Tribolium castaneum]
Unigene17099_All	-2.49856176	Down	glutathione S-transferase sigma 7 [Locusta migratoria]
CL9313.Contig1_All	-2.50139833	Down	PREDICTED: RNA-directed DNA polymerase from mobile element jockey-like [Hydra magnipapillata]
Unigene5074_All	-2.50747998	Down	conserved hypothetical protein [Neospora caninum Liverpool] >gi|325116081|emb|CBZ51635.1| conserved hypothetical protein [Neospora caninum Liverpool]
Unigene14865_All	-2.50753395	Down	GH10838 [Drosophila grimshawi] >gi|193903927|gb|EDW02794.1| GH10838 [Drosophila grimshawi]
Unigene28394_All	-2.52706003	Down	hypothetical protein DAPPUDRAFT_305140 [Daphnia pulex]
Unigene27354_All	-2.53130437	Down	hypothetical protein TcasGA2_TC013859 [Tribolium castaneum]
Unigene30086_All	-2.54005417	Down	glycerol kinase, putative [Pediculus humanus corporis] >gi|212515272|gb|EEB17443.1| glycerol kinase, putative [Pediculus humanus corporis]
CL1827.Contig3_All	-2.54284583	Down	PREDICTED: peroxidase-like [Megachile rotundata]
Unigene36471_All	-2.5512778	Down	PREDICTED: L-xylulose reductase-like [Oreochromis niloticus]
Unigene8469_All	-2.5557124	Down	PREDICTED: methenyltetrahydrofolate synthase domain-containing protein [Nasonia vitripennis]
Unigene38240_All	-2.58459986	Down	enzymatic polyprotein; Endonuclease; Reverse transcriptase, putative [Pediculus humanus corporis] >gi|212516797|gb|EEB18765.1| enzymatic polyprotein; Endonuclease; Reverse transcriptase, putative [Pediculus humanus corporis]
CL199.Contig5_All	-2.5846169	Down	PREDICTED: uncharacterized protein LOC100889850 [Strongylocentrotus purpuratus]
CL6289.Contig2_All	-2.61951595	Down	phosphoserine phosphatase [Locusta migratoria]
Unigene35266_All	-2.62744376	Down	NADH dehydrogenase subunit 4 [Oedaleus decorus asiaticus] >gi|168806203|gb|ACA28988.1| NADH dehydrogenase subunit 4 [Oedaleus decorus asiaticus]
CL9704.Contig2_All	-2.62762688	Down	PREDICTED: RNA-directed DNA polymerase from mobile element jockey-like [Strongylocentrotus purpuratus]
Unigene12208_All	-2.6351483	Down	hypothetical protein CAPTEDRAFT_177589 [Capitella teleta]
Unigene14918_All	-2.64354195	Down	beta-glucosidase [Coptotermes formosanus]
CL6709.Contig1_All	-2.64864166	Down	7-dehydrocholesterol reductase, putative [Pediculus humanus corporis] >gi|212506525|gb|EEB10709.1|

<table>
<tr><td></td><td></td><td></td><td>7-dehydrocholesterol reductase, putative [Pediculus humanus corporis]</td></tr>
<tr><td>Unigene5534_All</td><td>-2.65691702</td><td>Down</td><td>CRE-COL-50 protein [Caenorhabditis remanei] >gi|308259239|gb|EFP03192.1| CRE-COL-50 protein [Caenorhabditis remanei]</td></tr>
<tr><td>CL8010.Contig1_All</td><td>-2.66443742</td><td>Down</td><td>PREDICTED: hypothetical protein LOC100568495 [Acyrthosiphon pisum]</td></tr>
<tr><td>Unigene16924_All</td><td>-2.67719774</td><td>Down</td><td>hCG1793893 [Homo sapiens]</td></tr>
<tr><td>CL1923.Contig2_All</td><td>-2.67958722</td><td>Down</td><td>cytochrome c oxidase subunit II [Oedaleus decorus asiaticus] >gi|168806197|gb|ACA28982.1| cytochrome c oxidase subunit II [Oedaleus decorus asiaticus]</td></tr>
<tr><td>CL7189.Contig2_All</td><td>-2.7098878</td><td>Down</td><td>1-acyl-sn-glycerol-3-phosphate acyltransferase [Papilio xuthus]</td></tr>
<tr><td>Unigene39065_All</td><td>-2.70997276</td><td>Down</td><td>hypothetical protein KGM_11683 [Danaus plexippus]</td></tr>
<tr><td>CL179.Contig4_All</td><td>-2.71007991</td><td>Down</td><td>glutathione S-transferase sigma 4 [Locusta migratoria]</td></tr>
<tr><td>Unigene34600_All</td><td>-2.73164143</td><td>Down</td><td>5' nucleotidase [Culex quinquefasciatus] >gi|167864121|gb|EDS27504.1| 5' nucleotidase [Culex quinquefasciatus]</td></tr>
<tr><td>CL8606.Contig1_All</td><td>-2.76575677</td><td>Down</td><td>cytochrome b [Locusta migratoria manilensis] >gi|364503733|gb|AEW48562.1| cytochrome b (mitochondrion) [Locusta migratoria] >gi|364503747|gb|AEW48575.1| cytochrome b (mitochondrion) [Locusta migratoria] >gi|364503761|gb|AEW48588.1| cytochrome b (mitochondrion) [Locusta migratoria] >gi|364503775|gb|AEW48601.1| cytochrome b (mitochondrion) [Locusta migratoria] >gi|364503789|gb|AEW48614.1| cytochrome b (mitochondrion) [Locusta migratoria] >gi|364503803|gb|AEW48627.1| cytochrome b (mitochondrion) [Locusta migratoria] >gi|364503817|gb|AEW48640.1| cytochrome b (mitochondrion) [Locusta migratoria] >gi|364503831|gb|AEW48653.1| cytochrome b (mitochondrion) [Locusta migratoria] >gi|364503845|gb|AEW48666.1| cytochrome b (mitochondrion) [Locusta migratoria] >gi|364503859|gb|AEW48679.1| cytochrome b (mitochondrion) [Locusta migratoria] >gi|364503873|gb|AEW48692.1| cytochrome b (mitochondrion) [Locusta migratoria] >gi|364503887|gb|AEW48705.1| cytochrome b (mitochondrion) [Locusta migratoria] >gi|364503901|gb|AEW48718.1| cytochrome b (mitochondrion) [Locusta migratoria] >gi|364503915|gb|AEW48731.1| cytochrome b (mitochondrion) [Locusta migratoria] >gi|393802098|gb|AFN24591.1| cytochrome b (mitochondrion) [Locusta migratoria] >gi|393802106|gb|AFN24595.1| cytochrome b (mitochondrion) [Locusta</td></tr>
</table>

			migratoria] >gi|393802108|gb|AFN24596.1| cytochrome b (mitochondrion) [Locusta<br>migratoria] >gi|393802110|gb|AFN24597.1| cytochrome b (mitochondrion) [Locusta<br>migratoria] >gi|393802112|gb|AFN24598.1| cytochrome b (mitochondrion) [Locusta<br>migratoria] >gi|393802114|gb|AFN24599.1| cytochrome b (mitochondrion) [Locusta<br>migratoria] >gi|393802116|gb|AFN24600.1| cytochrome b (mitochondrion) [Locusta<br>migratoria] >gi|393802118|gb|AFN24601.1| cytochrome b (mitochondrion) [Locusta<br>migratoria] >gi|393802120|gb|AFN24602.1| cytochrome b (mitochondrion) [Locusta<br>migratoria] >gi|393802122|gb|AFN24603.1| cytochrome b (mitochondrion) [Locusta<br>migratoria] >gi|393802124|gb|AFN24604.1| cytochrome b (mitochondrion) [Locusta<br>migratoria] >gi|393802126|gb|AFN24605.1| cytochrome b (mitochondrion) [Locusta<br>migratoria] >gi|393802128|gb|AFN24606.1| cytochrome b (mitochondrion) [Locusta<br>migratoria] >gi|393802130|gb|AFN24607.1| cytochrome b (mitochondrion) [Locusta<br>migratoria] >gi|393802132|gb|AFN24608.1| cytochrome b (mitochondrion) [Locusta<br>migratoria] >gi|393802134|gb|AFN24609.1| cytochrome b (mitochondrion) [Locusta<br>migratoria] >gi|393802136|gb|AFN24610.1| cytochrome b (mitochondrion) [Locusta<br>migratoria] >gi|393802138|gb|AFN24611.1| cytochrome b (mitochondrion) [Locusta<br>migratoria] >gi|393802140|gb|AFN24612.1| cytochrome b (mitochondrion) [Locusta<br>migratoria] >gi|393802142|gb|AFN24613.1| cytochrome b (mitochondrion) [Locusta<br>migratoria] >gi|393802144|gb|AFN24614.1| cytochrome b (mitochondrion) [Locusta<br>migratoria] >gi|393802146|gb|AFN24615.1| cytochrome b (mitochondrion) [Locusta<br>migratoria] >gi|393802148|gb|AFN24616.1| cytochrome b (mitochondrion) [Locusta<br>migratoria] >gi|393802150|gb|AFN24617.1| cytochrome b (mitochondrion) [Locusta<br>migratoria] >gi|393802152|gb|AFN24618.1| cytochrome b (mitochondrion) [Locusta<br>migratoria] >gi|393802154|gb|AFN24619.1| cytochrome b (mitochondrion) [Locusta<br>migratoria] >gi|393802156|gb|AFN24620.1| cytochrome b (mitochondrion) [Locusta<br>migratoria] >gi|393802158|gb|AFN24621.1| cytochrome b (mitochondrion) [Locusta<br>migratoria] >gi|393802160|gb|AFN24622.1| cytochrome b (mitochondrion) [Locusta<br>migratoria] >gi|393802162|gb|AFN24623.1| cytochrome b (mitochondrion) [Locusta

			migratoria] >gi|393802164|gb|AFN24624.1| cytochrome b (mitochondrion) [Locusta migratoria] >gi|393802166|gb|AFN24625.1| cytochrome b (mitochondrion) [Locusta migratoria] >gi|393802168|gb|AFN24626.1| cytochrome b (mitochondrion) [Locusta migratoria] >gi|393802170|gb|AFN24627.1| cytochrome b (mitochondrion) [Locusta migratoria] >gi|393802172|gb|AFN24628.1| cytochrome b (mitochondrion) [Locusta migratoria] >gi|393802174|gb|AFN24629.1| cytochrome b (mitochondrion) [Locusta migratoria] >gi|393802176|gb|AFN24630.1| cytochrome b (mitochondrion) [Locusta migratoria] >gi|393802178|gb|AFN24631.1| cytochrome b (mitochondrion) [Locusta migratoria] >gi|393802180|gb|AFN24632.1| cytochrome b (mitochondrion) [Locusta migratoria] >gi|393802182|gb|AFN24633.1| cytochrome b (mitochondrion) [Locusta migratoria] >gi|393802184|gb|AFN24634.1| cytochrome b (mitochondrion) [Locusta migratoria] >gi|393802186|gb|AFN24635.1| cytochrome b (mitochondrion) [Locusta migratoria] >gi|393802254|gb|AFN24669.1| cytochrome b (mitochondrion) [Locusta migratoria] >gi|393802282|gb|AFN24683.1| cytochrome b (mitochondrion) [Locusta migratoria]
CL2858.Contig1_All	-2.78815586	Down	juvenile hormone esterase-like protein Est1 [Reticulitermes flavipes]
Unigene17477_All	-2.80772761	Down	PREDICTED: similar to putative zinc-containing alcohol dehydrogenase [Tribolium castaneum] >gi|270012005|gb|EFA08453.1| hypothetical protein TcasGA2_TC006100 [Tribolium castaneum]
CL3293.Contig1_All	-2.80954235	Down	hypothetical protein EGM_09670, partial [Macaca fascicularis]
CL1272.Contig1_All	-2.81522823	Down	hypothetical protein CRE_09591 [Caenorhabditis remanei] >gi|308259489|gb|EFP03442.1| hypothetical protein CRE_09591 [Caenorhabditis remanei]
Unigene39557_All	-2.82532252	Down	hypothetical protein DAPPUDRAFT_311578 [Daphnia pulex]
CL5564.Contig2_All	-2.8255493	Down	endonuclease-reverse transcriptase [Bombyx mori]
Unigene444_All	-2.82723575	Down	NADH dehydrogenase subunit 3 [Locusta migratoria migratoria] >gi|334362235|ref|YP_004564568.1| NADH dehydrogenase subunit 3 [Locusta migratoria tibetensis] >gi|161172430|gb|ABX59317.1| NADH dehydrogenase subunit 3 [Locusta migratoria migratoria] >gi|297242109|gb|ADI24720.1| NADH dehydrogenase subunit 3 [Locusta migratoria tibetensis] >gi|308206956|gb|ADO20004.1| NADH dehydrogenase subunit 3 [Locusta migratoria manilensis] >gi|364503686|gb|AEW48518.1| NADH

			dehydrogenase subunit 3 (mitochondrion) [Locusta migratoria] >gi|364503700|gb|AEW48531.1| NADH dehydrogenase subunit 3 (mitochondrion) [Locusta migratoria] >gi|364503714|gb|AEW48544.1| NADH dehydrogenase subunit 3 (mitochondrion) [Locusta migratoria] >gi|364503728|gb|AEW48557.1| NADH dehydrogenase subunit 3 (mitochondrion) [Locusta migratoria] >gi|364503742|gb|AEW48570.1| NADH dehydrogenase subunit 3 (mitochondrion) [Locusta migratoria] >gi|364503756|gb|AEW48583.1| NADH dehydrogenase subunit 3 (mitochondrion) [Locusta migratoria] >gi|364503770|gb|AEW48596.1| NADH dehydrogenase subunit 3 (mitochondrion) [Locusta migratoria] >gi|364503784|gb|AEW48609.1| NADH dehydrogenase subunit 3 (mitochondrion) [Locusta migratoria] >gi|364503798|gb|AEW48622.1| NADH dehydrogenase subunit 3 (mitochondrion) [Locusta migratoria] >gi|364503812|gb|AEW48635.1| NADH dehydrogenase subunit 3 (mitochondrion) [Locusta migratoria] >gi|364503826|gb|AEW48648.1| NADH dehydrogenase subunit 3 (mitochondrion) [Locusta migratoria] >gi|364503840|gb|AEW48661.1| NADH dehydrogenase subunit 3 (mitochondrion) [Locusta migratoria] >gi|364503854|gb|AEW48674.1| NADH dehydrogenase subunit 3 (mitochondrion) [Locusta migratoria] >gi|364503868|gb|AEW48687.1| NADH dehydrogenase subunit 3 (mitochondrion) [Locusta migratoria] >gi|364503882|gb|AEW48700.1| NADH dehydrogenase subunit 3 (mitochondrion) [Locusta migratoria] >gi|364503910|gb|AEW48726.1| NADH dehydrogenase subunit 3 (mitochondrion) [Locusta migratoria] >gi|364503924|gb|AEW48739.1| NADH dehydrogenase subunit 3 (mitochondrion) [Locusta migratoria] >gi|364503938|gb|AEW48752.1| NADH dehydrogenase subunit 3 (mitochondrion) [Locusta migratoria] >gi|364503952|gb|AEW48765.1| NADH dehydrogenase subunit 3 (mitochondrion) [Locusta migratoria] >gi|364503966|gb|AEW48778.1| NADH dehydrogenase subunit 3 (mitochondrion) [Locusta migratoria] >gi|364503994|gb|AEW48804.1| NADH dehydrogenase subunit 3 (mitochondrion) [Locusta migratoria] >gi|364504008|gb|AEW48817.1| NADH dehydrogenase subunit 3 (mitochondrion) [Locusta migratoria] >gi|364504022|gb|AEW48830.1| NADH dehydrogenase subunit 3 (mitochondrion) [Locusta migratoria] >gi|364504036|gb|AEW48843.1| NADH dehydrogenase subunit 3 (mitochondrion) [Locusta migratoria] >gi|364504050|gb|AEW48856.1| NADH dehydrogenase subunit 3 (mitochondrion) [Locusta migratoria] >gi|364504064|gb|AEW48869.1| NADH dehydrogenase subunit 3 (mitochondrion) [Locusta migratoria] >gi|364504078|gb|AEW48882.1| NADH dehydrogenase subunit 3 (mitochondrion) [Locusta migratoria] >gi|364504092|gb|AEW48895.1| NADH dehydrogenase subunit 3 (mitochondrion) [Locusta migratoria]

CL8811.Contig2_All	-2.84711023	Down	PREDICTED: ribose-5-phosphate isomerase-like [Monodelphis domestica]
CL7983.Contig4_All	-2.84743236	Down	PREDICTED: similar to phosphomannomutase [Tribolium castaneum]
CL7324.Contig1_All	-2.84994763	Down	hypothetical protein TcasGA2_TC005413 [Tribolium castaneum]
Unigene28757_All	-2.853045	Down	PREDICTED: aspartate aminotransferase, cytoplasmic [Tursiops truncatus]
CL4783.Contig4_All	-2.86187529	Down	hypothetical protein WUBG_15994 [Wuchereria bancrofti]
CL9859.Contig1_All	-2.86198402	Down	hypothetical protein TcasGA2_TC000782 [Tribolium castaneum]
CL4657.Contig2_All	-2.86248436	Down	AGAP000010-PA [Anopheles gambiae str. PEST] >gi|333467406|gb|EAA06461.5| AGAP000010-PA [Anopheles gambiae str. PEST]
CL179.Contig3_All	-2.87256875	Down	glutathione S-transferase sigma 3 [Locusta migratoria]
Unigene29395_All	-2.90274546	Down	juvenile hormone binding protein [Locusta migratoria]
CL6705.Contig2_All	-2.90655308	Down	PREDICTED: hypothetical protein LOC100574452 [Acyrthosiphon pisum]
Unigene29351_All	-2.9324229	Down	hypothetical protein TcasGA2_TC012513 [Tribolium castaneum]
CL508.Contig1_All	-2.93844341	Down	PREDICTED: alanine--glyoxylate aminotransferase 2, mitochondrial isoform 2 [Callithrix jacchus]
Unigene36192_All	-2.95287912	Down	hypothetical protein CAPTEDRAFT_218289 [Capitella teleta]
Unigene19963_All	-2.95409124	Down	Linear gramicidin synthetase subunit D [Crassostrea gigas]
CL3069.Contig1_All	-2.97041516	Down	PREDICTED: uncharacterized protein LOC100889615 [Strongylocentrotus purpuratus]
Unigene19614_All	-2.97919303	Down	putative homocysteine S-methyltransferase isoform 1 [Danaus plexippus]
CL6048.Contig1_All	-3.02195609	Down	PREDICTED: hypothetical protein LOC100165998 [Acyrthosiphon pisum]
CL2227.Contig2_All	-3.03164116	Down	L-xylulose reductase [Danio rerio] >gi|62202602|gb|AAH93141.1| Dicarbonyl/L-xylulose reductase [Danio rerio]
Unigene29692_All	-3.03184343	Down	Transposable element Tcb1 transposase [Salmo salar]
Unigene10882_All	-3.03191833	Down	PREDICTED: similar to fumarylacetoacetate hydrolase [Tribolium castaneum] >gi|270009995|gb|EFA06443.1| hypothetical protein TcasGA2_TC009325 [Tribolium castaneum]
Unigene35851_All	-3.05100892	Down	hypothetical protein AND_22386 [Anopheles darlingi]
Unigene27687_All	-3.12515513	Down	PREDICTED: pyridoxine/pyridoxamine 5'-phosphate oxidase-like [Nasonia vitripennis]
Unigene29085_All	-3.15736929	Down	alpha-amylase [Blattella germanica]
CL4381.Contig2_All	-3.18390429	Down	PREDICTED: similar to AGAP002711-PA [Tribolium castaneum]

Unigene36258_All	-3.18399024	Down	hexamerin-like protein 2 [Locusta migratoria]
Unigene30723_All	-3.24596758	Down	glucosyl glucuronosyl transferases [Locusta migratoria]
CL7298.Contig1_All	-3.24678203	Down	PREDICTED: bifunctional coenzyme A synthase-like [Megachile rotundata]
Unigene7768_All	-3.24798459	Down	PREDICTED: hypothetical protein LOC100572891 [Acyrthosiphon pisum]
Unigene38403_All	-3.254217	Down	predicted protein [Nematostella vectensis] >gi|156208760|gb|EDO30168.1| predicted protein [Nematostella vectensis]
CL2923.Contig2_All	-3.29482038	Down	glutamate--cysteine ligase regulatory subunit, putative [Pediculus humanus corporis] >gi|212518106|gb|EEB19898.1| glutamate--cysteine ligase regulatory subunit, putative [Pediculus humanus corporis]
Unigene20468_All	-3.30500366	Down	NADH dehydrogenase 1 alpha subcomplex subunit 4 [Dicentrarchus labrax]
Unigene539_All	-3.30593851	Down	GL16995 [Drosophila persimilis] >gi|194113807|gb|EDW35850.1| GL16995 [Drosophila persimilis]
Unigene32721_All	-3.36713398	Down	PREDICTED: putative gamma-glutamyltransferase ywrD-like [Xenopus (Silurana) tropicalis]
CL6572.Contig4_All	-3.37274327	Down	ND1 gene product (mitochondrion) [Sylvicola fenestralis] >gi|356489028|gb|AET13133.1| NADH dehydrogenase subunit 1 (mitochondrion) [Sylvicola fenestralis]
Unigene25915_All	-3.42902419	Down	cytochrome c oxidase subunit II (mitochondrion) [Locusta migratoria]
Unigene20152_All	-3.44701991	Down	fructose 1,6-bisphosphate aldolase [Schistocerca gregaria]
Unigene31118_All	-3.49431949	Down	PREDICTED: d-3-phosphoglycerate dehydrogenase-like [Megachile rotundata]
Unigene10903_All	-3.50885439	Down	beta-glucosidase [Coptotermes formosanus]
Unigene4416_All	-3.57646744	Down	PREDICTED: similar to AGAP003501-PA [Tribolium castaneum] >gi|270005168|gb|EFA01616.1| hypothetical protein TcasGA2_TC007185 [Tribolium castaneum]
Unigene15270_All	-3.59101094	Down	enoyl-CoA hydratase, mitochondrial precursor, putative [Trypanosoma cruzi marinkellei]
Unigene33814_All	-3.64854055	Down	NADH dehydrogenase subunit 1 (mitochondrion) [Ceracris kiangsu] >gi|284098441|gb|ADB78285.1| NADH dehydrogenase subunit 1 (mitochondrion) [Ceracris kiangsu]
Unigene31950_All	-3.64876266	Down	GI24716 [Drosophila mojavensis] >gi|193913586|gb|EDW12453.1| GI24716 [Drosophila mojavensis]
Unigene35832_All	-3.70986259	Down	PREDICTED: L-serine dehydratase/L-threonine deaminase-like [Takifugu rubripes]
CL2178.Contig1_All	-3.71794511	Down	PREDICTED: 2-oxoglutarate dehydrogenase, mitochondrial [Oreochromis niloticus]
CL2052.Contig1_All	-3.73965129	Down	NADH dehydrogenase subunit 4 [Gastrimargus marmoratus] >gi|168806189|gb|ACA28975.1| NADH dehydrogenase subunit 4 [Gastrimargus marmoratus]

CL1356.Contig1_All	-3.81869107	Down	PREDICTED: hypothetical protein LOC100570299 [Acyrthosiphon pisum]
CL367.Contig2_All	-3.82673359	Down	mitochondrial ribosomal protein VAR1, putative [Pediculus humanus corporis] >gi|212514798|gb|EEB17048.1| mitochondrial ribosomal protein VAR1, putative [Pediculus humanus corporis]
Unigene15777_All	-3.8691464	Down	conserved hypothetical protein [Culex quinquefasciatus] >gi|167867478|gb|EDS30861.1| conserved hypothetical protein [Culex quinquefasciatus]
Unigene20081_All	-3.89909599	Down	beta-glucosidase [Coptotermes formosanus]
CL9416.Contig1_All	-3.91967658	Down	Retinal dehydrogenase 1 [Camponotus floridanus]
Unigene31533_All	-3.92583236	Down	cytochrome oxidase subunit II, partial (mitochondrion) [Opeia obscura]
CL2091.Contig2_All	-3.93330688	Down	RecName: Full=Basic proline-rich protein; Contains: RecName: Full=Proline-rich peptide SP-B-like; Short=PRP-SP-B-like; Contains: RecName: Full=Proline-rich peptide SP-A; Short=PRP-SP-A; Contains: RecName: Full=Proline-rich peptide SP-B; Short=PRP-SP-B; Contains: RecName: Full=Parotid hormone; AltName: Full=PH-Ab; Flags: Precursor
CL1765.Contig1_All	-3.99170541	Down	aminopeptidase N [Sitophilus oryzae]
Unigene12060_All	-4.01585268	Down	Nucleoside diphosphate kinase-like protein 5 [Harpegnathos saltator]
CL5053.Contig1_All	-4.03332723	Down	juvenile hormone esterase-like protein Est1 [Reticulitermes flavipes]
Unigene23741_All	-4.0698726	Down	PREDICTED: UDP-glucuronosyltransferase 1-10-like [Nasonia vitripennis]
CL1673.Contig1_All	-4.08590167	Down	glucosyl glucuronosyl transferases [Locusta migratoria]
Unigene28888_All	-4.08861035	Down	hypothetical protein CRE_16092 [Caenorhabditis remanei] >gi|308253824|gb|EFO97776.1| hypothetical protein CRE_16092 [Caenorhabditis remanei]
CL367.Contig4_All	-4.12729515	Down	mitochondrial ribosomal protein VAR1, putative [Pediculus humanus corporis] >gi|212514798|gb|EEB17048.1| mitochondrial ribosomal protein VAR1, putative [Pediculus humanus corporis]
Unigene8013_All	-4.1481182	Down	allantoicase [Branchiostoma belcheri tsingtauense]
Unigene26799_All	-4.17824551	Down	PREDICTED: uncharacterized protein K02A2.6-like [Metaseiulus occidentalis]
Unigene31944_All	-4.19116786	Down	alpha-amylase [Drosophila melanogaster]
CL7189.Contig1_All	-4.19860469	Down	1-acyl-sn-glycerol-3-phosphate acyltransferase [Papilio xuthus]
Unigene27843_All	-4.21275048	Down	PREDICTED: angiomotin [Otolemur garnettii]

Unigene30332_All	-4.22189967	Down	GD10568 [Drosophila simulans] >gi|194192606|gb|EDX06182.1| GD10568 [Drosophila simulans]
Unigene29656_All	-4.29502491	Down	unknown [Dendroctonus ponderosae]
CL3250.Contig1_All	-4.34273219	Down	loricrin [Homo sapiens]
CL2227.Contig1_All	-4.36212754	Down	L-xylulose reductase [Anoplopoma fimbria]
CL1923.Contig1_All	-4.36446867	Down	cytochrome c oxidase subunit II [Locusta migratoria tibetensis] >gi|154795799|gb|ABS86898.1| cytochrome c oxidase subunit II [Locusta migratoria] >gi|169792281|gb|ACA80345.1| cytochrome oxidase subunit II [Locusta migratoria] >gi|169792295|gb|ACA80357.1| cytochrome oxidase subunit II [Locusta migratoria] >gi|169792302|gb|ACA80363.1| cytochrome oxidase subunit II [Locusta migratoria] >gi|297242104|gb|ADI24715.1| cytochrome c oxidase subunit II [Locusta migratoria tibetensis] >gi|308206951|gb|ADO19999.1| cytochrome c oxidase subunit II [Locusta migratoria manilensis] >gi|364503682|gb|AEW48514.1| cytochrome c oxidase subunit II (mitochondrion) [Locusta migratoria] >gi|364503696|gb|AEW48527.1| cytochrome c oxidase subunit II (mitochondrion) [Locusta migratoria] >gi|364503710|gb|AEW48540.1| cytochrome c oxidase subunit II (mitochondrion) [Locusta migratoria] >gi|364503724|gb|AEW48553.1| cytochrome c oxidase subunit II (mitochondrion) [Locusta migratoria] >gi|364503738|gb|AEW48566.1| cytochrome c oxidase subunit II (mitochondrion) [Locusta migratoria] >gi|364503752|gb|AEW48579.1| cytochrome c oxidase subunit II (mitochondrion) [Locusta migratoria] >gi|364503766|gb|AEW48592.1| cytochrome c oxidase subunit II (mitochondrion) [Locusta migratoria] >gi|364503780|gb|AEW48605.1| cytochrome c oxidase subunit II (mitochondrion) [Locusta migratoria] >gi|364503794|gb|AEW48618.1| cytochrome c oxidase subunit II (mitochondrion) [Locusta migratoria] >gi|364503808|gb|AEW48631.1| cytochrome c oxidase subunit II (mitochondrion) [Locusta migratoria] >gi|364503822|gb|AEW48644.1| cytochrome c oxidase subunit II (mitochondrion) [Locusta migratoria] >gi|364503836|gb|AEW48657.1| cytochrome c oxidase subunit II (mitochondrion) [Locusta migratoria] >gi|364503864|gb|AEW48683.1| cytochrome c oxidase subunit II (mitochondrion) [Locusta migratoria] >gi|364503892|gb|AEW48709.1| cytochrome c oxidase subunit II (mitochondrion) [Locusta migratoria] >gi|364503906|gb|AEW48722.1| cytochrome c oxidase subunit II (mitochondrion) [Locusta migratoria] >gi|364503920|gb|AEW48735.1| cytochrome c oxidase subunit II (mitochondrion) [Locusta migratoria] >gi|364503934|gb|AEW48748.1| cytochrome c oxidase subunit II (mitochondrion) [Locusta migratoria] >gi|364503948|gb|AEW48761.1| cytochrome c oxidase subunit II (mitochondrion) [Locusta

<table>
<tr>
<td></td>
<td></td>
<td></td>
<td>migratoria] >gi|364503962|gb|AEW48774.1| cytochrome c oxidase subunit II (mitochondrion) [Locusta migratoria] >gi|364503976|gb|AEW48787.1| cytochrome c oxidase subunit II (mitochondrion) [Locusta migratoria] >gi|364503990|gb|AEW48800.1| cytochrome c oxidase subunit II (mitochondrion) [Locusta migratoria] >gi|364504004|gb|AEW48813.1| cytochrome c oxidase subunit II (mitochondrion) [Locusta migratoria] >gi|364504018|gb|AEW48826.1| cytochrome c oxidase subunit II (mitochondrion) [Locusta migratoria] >gi|364504032|gb|AEW48839.1| cytochrome c oxidase subunit II (mitochondrion) [Locusta migratoria] >gi|364504046|gb|AEW48852.1| cytochrome c oxidase subunit II (mitochondrion) [Locusta migratoria] >gi|364504060|gb|AEW48865.1| cytochrome c oxidase subunit II (mitochondrion) [Locusta migratoria] >gi|364504074|gb|AEW48878.1| cytochrome c oxidase subunit II (mitochondrion) [Locusta migratoria] >gi|364504102|gb|AEW48904.1| cytochrome c oxidase subunit II (mitochondrion) [Locusta migratoria] >gi|364504116|gb|AEW48917.1| cytochrome c oxidase subunit II (mitochondrion) [Locusta migratoria] >gi|364504130|gb|AEW48930.1| cytochrome c oxidase subunit II (mitochondrion) [Locusta migratoria] >gi|364504144|gb|AEW48943.1| cytochrome c oxidase subunit II (mitochondrion) [Locusta migratoria] >gi|364504158|gb|AEW48956.1| cytochrome c oxidase subunit II (mitochondrion) [Locusta migratoria] >gi|364504172|gb|AEW48969.1| cytochrome c oxidase subunit II (mitochondrion) [Locusta migratoria] >gi|364504186|gb|AEW48982.1| cytochrome c oxidase subunit II (mitochondrion) [Locusta migratoria] >gi|364504200|gb|AEW48995.1| cytochrome c oxidase subunit II (mitochondrion) [Locusta migratoria] >gi|364504214|gb|AEW49008.1| cytochrome c oxidase subunit II (mitochondrion) [Locusta migratoria] >gi|364504228|gb|AEW49021.1| cytochrome c oxidase subunit II (mitochondrion) [Locusta migratoria] >gi|364504256|gb|AEW49047.1| cytochrome c oxidase subunit II (mitochondrion) [Locusta migratoria] >gi|364504270|gb|AEW49060.1| cytochrome c oxidase subunit II (mitochondrion) [Locusta migratoria] >gi|364504284|gb|AEW49073.1| cytochrome c oxidase subunit II (mitochondrion) [Locusta migratoria] >gi|364504298|gb|AEW49086.1| cytochrome c oxidase subunit II (mitochondrion) [Locusta migratoria] >gi|364504312|gb|AEW49099.1| cytochrome c oxidase subunit II (mitochondrion) [Locusta migratoria] >gi|364504326|gb|AEW49112.1| cytochrome c oxidase subunit II (mitochondrion) [Locusta migratoria] >gi|364504340|gb|AEW49125.1| cytochrome c oxidase subunit II (mitochondrion) [Locusta migratoria] >gi|364504354|gb|AEW49138.1| cytochrome c oxidase subunit II (mitochondrion) [Locusta migratoria] >gi|364504368|gb|AEW49151.1| cytochrome c oxidase subunit II (mitochondrion) [Locusta</td>
</tr>
</table>

			migratoria] >gi|364504382|gb|AEW49164.1| cytochrome c oxidase subunit II (mitochondrion) [Locusta migratoria] >gi|364504396|gb|AEW49177.1| cytochrome c oxidase subunit II (mitochondrion) [Locusta migratoria] >gi|364504410|gb|AEW49190.1| cytochrome c oxidase subunit II (mitochondrion) [Locusta migratoria] >gi|364504424|gb|AEW49203.1| cytochrome c oxidase subunit II (mitochondrion) [Locusta migratoria] >gi|364504438|gb|AEW49216.1| cytochrome c oxidase subunit II (mitochondrion) [Locusta migratoria] >gi|364504452|gb|AEW49229.1| cytochrome c oxidase subunit II (mitochondrion) [Locusta migratoria] >gi|364504466|gb|AEW49242.1| cytochrome c oxidase subunit II (mitochondrion) [Locusta migratoria] >gi|364504480|gb|AEW49255.1| cytochrome c oxidase subunit II (mitochondrion) [Locusta migratoria] >gi|364504494|gb|AEW49268.1| cytochrome c oxidase subunit II (mitochondrion) [Locusta migratoria] >gi|364504508|gb|AEW49281.1| cytochrome c oxidase subunit II (mitochondrion) [Locusta migratoria] >gi|364504522|gb|AEW49294.1| cytochrome c oxidase subunit II (mitochondrion) [Locusta migratoria] >gi|364504536|gb|AEW49307.1| cytochrome c oxidase subunit II (mitochondrion) [Locusta migratoria] >gi|364504550|gb|AEW49320.1| cytochrome c oxidase subunit II (mitochondrion) [Locusta migratoria] >gi|364504578|gb|AEW49346.1| cytochrome c oxidase subunit II (mitochondrion) [Locusta migratoria]
Unigene36091_All	-4.44624213	Down	senescence marker protein-30, putative [Pediculus humanus corporis] >gi|212517874|gb|EEB19703.1| senescence marker protein-30, putative [Pediculus humanus corporis]
CL8606.Contig2_All	-4.46479842	Down	cytochrome b [Locusta migratoria manilensis] >gi|364503733|gb|AEW48562.1| cytochrome b (mitochondrion) [Locusta migratoria] >gi|364503747|gb|AEW48575.1| cytochrome b (mitochondrion) [Locusta migratoria] >gi|364503761|gb|AEW48588.1| cytochrome b (mitochondrion) [Locusta migratoria] >gi|364503775|gb|AEW48601.1| cytochrome b (mitochondrion) [Locusta migratoria] >gi|364503789|gb|AEW48614.1| cytochrome b (mitochondrion) [Locusta migratoria] >gi|364503803|gb|AEW48627.1| cytochrome b (mitochondrion) [Locusta migratoria] >gi|364503817|gb|AEW48640.1| cytochrome b (mitochondrion) [Locusta migratoria] >gi|364503831|gb|AEW48653.1| cytochrome b (mitochondrion) [Locusta migratoria] >gi|364503845|gb|AEW48666.1| cytochrome b (mitochondrion) [Locusta migratoria] >gi|364503859|gb|AEW48679.1| cytochrome b (mitochondrion) [Locusta migratoria] >gi|364503873|gb|AEW48692.1| cytochrome b (mitochondrion) [Locusta

<table>
<tr><td></td><td></td><td></td><td>migratoria] >gi|364503887|gb|AEW48705.1| cytochrome b (mitochondrion) [Locusta
migratoria] >gi|364503901|gb|AEW48718.1| cytochrome b (mitochondrion) [Locusta
migratoria] >gi|364503915|gb|AEW48731.1| cytochrome b (mitochondrion) [Locusta
migratoria] >gi|393802098|gb|AFN24591.1| cytochrome b (mitochondrion) [Locusta
migratoria] >gi|393802106|gb|AFN24595.1| cytochrome b (mitochondrion) [Locusta
migratoria] >gi|393802108|gb|AFN24596.1| cytochrome b (mitochondrion) [Locusta
migratoria] >gi|393802110|gb|AFN24597.1| cytochrome b (mitochondrion) [Locusta
migratoria] >gi|393802112|gb|AFN24598.1| cytochrome b (mitochondrion) [Locusta
migratoria] >gi|393802114|gb|AFN24599.1| cytochrome b (mitochondrion) [Locusta
migratoria] >gi|393802116|gb|AFN24600.1| cytochrome b (mitochondrion) [Locusta
migratoria] >gi|393802118|gb|AFN24601.1| cytochrome b (mitochondrion) [Locusta
migratoria] >gi|393802120|gb|AFN24602.1| cytochrome b (mitochondrion) [Locusta
migratoria] >gi|393802122|gb|AFN24603.1| cytochrome b (mitochondrion) [Locusta
migratoria] >gi|393802124|gb|AFN24604.1| cytochrome b (mitochondrion) [Locusta
migratoria] >gi|393802126|gb|AFN24605.1| cytochrome b (mitochondrion) [Locusta
migratoria] >gi|393802128|gb|AFN24606.1| cytochrome b (mitochondrion) [Locusta
migratoria] >gi|393802130|gb|AFN24607.1| cytochrome b (mitochondrion) [Locusta
migratoria] >gi|393802132|gb|AFN24608.1| cytochrome b (mitochondrion) [Locusta
migratoria] >gi|393802134|gb|AFN24609.1| cytochrome b (mitochondrion) [Locusta
migratoria] >gi|393802136|gb|AFN24610.1| cytochrome b (mitochondrion) [Locusta
migratoria] >gi|393802138|gb|AFN24611.1| cytochrome b (mitochondrion) [Locusta
migratoria] >gi|393802140|gb|AFN24612.1| cytochrome b (mitochondrion) [Locusta
migratoria] >gi|393802142|gb|AFN24613.1| cytochrome b (mitochondrion) [Locusta
migratoria] >gi|393802144|gb|AFN24614.1| cytochrome b (mitochondrion) [Locusta
migratoria] >gi|393802146|gb|AFN24615.1| cytochrome b (mitochondrion) [Locusta
migratoria] >gi|393802148|gb|AFN24616.1| cytochrome b (mitochondrion) [Locusta
migratoria] >gi|393802150|gb|AFN24617.1| cytochrome b (mitochondrion) [Locusta
migratoria] >gi|393802152|gb|AFN24618.1| cytochrome b (mitochondrion) [Locusta</td></tr>
</table>

			migratoria] >gi|393802154|gb|AFN24619.1| cytochrome b (mitochondrion) [Locusta migratoria] >gi|393802156|gb|AFN24620.1| cytochrome b (mitochondrion) [Locusta migratoria] >gi|393802158|gb|AFN24621.1| cytochrome b (mitochondrion) [Locusta migratoria] >gi|393802160|gb|AFN24622.1| cytochrome b (mitochondrion) [Locusta migratoria] >gi|393802162|gb|AFN24623.1| cytochrome b (mitochondrion) [Locusta migratoria] >gi|393802164|gb|AFN24624.1| cytochrome b (mitochondrion) [Locusta migratoria] >gi|393802166|gb|AFN24625.1| cytochrome b (mitochondrion) [Locusta migratoria] >gi|393802168|gb|AFN24626.1| cytochrome b (mitochondrion) [Locusta migratoria] >gi|393802170|gb|AFN24627.1| cytochrome b (mitochondrion) [Locusta migratoria] >gi|393802172|gb|AFN24628.1| cytochrome b (mitochondrion) [Locusta migratoria] >gi|393802174|gb|AFN24629.1| cytochrome b (mitochondrion) [Locusta migratoria] >gi|393802176|gb|AFN24630.1| cytochrome b (mitochondrion) [Locusta migratoria] >gi|393802178|gb|AFN24631.1| cytochrome b (mitochondrion) [Locusta migratoria] >gi|393802180|gb|AFN24632.1| cytochrome b (mitochondrion) [Locusta migratoria] >gi|393802182|gb|AFN24633.1| cytochrome b (mitochondrion) [Locusta migratoria] >gi|393802184|gb|AFN24634.1| cytochrome b (mitochondrion) [Locusta migratoria] >gi|393802186|gb|AFN24635.1| cytochrome b (mitochondrion) [Locusta migratoria] >gi|393802254|gb|AFN24669.1| cytochrome b (mitochondrion) [Locusta migratoria] >gi|393802282|gb|AFN24683.1| cytochrome b (mitochondrion) [Locusta migratoria]
Unigene4457_All	-4.53177494	Down	PREDICTED: similar to putative esterase [Tribolium castaneum] >gi|270008700|gb|EFA05148.1| hypothetical protein TcasGA2_TC015265 [Tribolium castaneum]
Unigene26197_All	-4.55127148	Down	NADH dehydrogenase subunit 4 (mitochondrion) [Locusta migratoria] >gi|364504332|gb|AEW49118.1| NADH dehydrogenase subunit 4 (mitochondrion) [Locusta migratoria] >gi|364504346|gb|AEW49131.1| NADH dehydrogenase subunit 4 (mitochondrion) [Locusta migratoria] >gi|364504360|gb|AEW49144.1| NADH dehydrogenase subunit 4 (mitochondrion) [Locusta migratoria] >gi|364504374|gb|AEW49157.1| NADH dehydrogenase subunit 4 (mitochondrion) [Locusta migratoria] >gi|364504388|gb|AEW49170.1| NADH dehydrogenase subunit 4 (mitochondrion) [Locusta migratoria] >gi|364504402|gb|AEW49183.1| NADH dehydrogenase subunit 4 (mitochondrion) [Locusta migratoria] >gi|364504416|gb|AEW49196.1|

			NADH dehydrogenase subunit 4 (mitochondrion) [Locusta migratoria] >gi|364504430|gb|AEW49209.1| NADH dehydrogenase subunit 4 (mitochondrion) [Locusta migratoria] >gi|364504444|gb|AEW49222.1| NADH dehydrogenase subunit 4 (mitochondrion) [Locusta migratoria] >gi|364504458|gb|AEW49235.1| NADH dehydrogenase subunit 4 (mitochondrion) [Locusta migratoria] >gi|364504472|gb|AEW49248.1| NADH dehydrogenase subunit 4 (mitochondrion) [Locusta migratoria] >gi|364504486|gb|AEW49261.1| NADH dehydrogenase subunit 4 (mitochondrion) [Locusta migratoria] >gi|364504500|gb|AEW49274.1| NADH dehydrogenase subunit 4 (mitochondrion) [Locusta migratoria] >gi|364504514|gb|AEW49287.1| NADH dehydrogenase subunit 4 (mitochondrion) [Locusta migratoria] >gi|364504528|gb|AEW49300.1| NADH dehydrogenase subunit 4 (mitochondrion) [Locusta migratoria] >gi|364504556|gb|AEW49326.1| NADH dehydrogenase subunit 4 (mitochondrion) [Locusta migratoria] >gi|364504570|gb|AEW49339.1| NADH dehydrogenase subunit 4 (mitochondrion) [Locusta migratoria] >gi|364504584|gb|AEW49352.1| NADH dehydrogenase subunit 4 (mitochondrion) [Locusta migratoria]
Unigene2168_All	-4.6269	Down	PREDICTED: similar to 3-oxoacyl-[acyl-carrier-protein] reductase [Tribolium castaneum] >gi|270008569|gb|EFA05017.1| hypothetical protein TcasGA2_TC015100 [Tribolium castaneum]
Unigene8518_All	-4.67067264	Down	sorbitol dehydrogenase [Pyrrhocoris apterus]
CL1673.Contig4_All	-4.74282402	Down	glucosyl glucuronosyl transferases [Locusta migratoria]
CL6337.Contig1_All	-4.77794202	Down	PREDICTED: putative gamma-glutamyltransferase ywrD-like [Anolis carolinensis]
Unigene27109_All	-4.81456293	Down	PREDICTED: uncharacterized protein LOC100889850 [Strongylocentrotus purpuratus]
Unigene21841_All	-4.85711613	Down	PREDICTED: uncharacterized short-chain type dehydrogenase/reductase y4vI-like [Xenopus (Silurana) tropicalis]
CL2557.Contig1_All	-5.00412346	Down	NADH dehydrogenase subunit 6 [Locusta migratoria manilensis] >gi|364503690|gb|AEW48522.1| NADH dehydrogenase subunit 6 (mitochondrion) [Locusta migratoria] >gi|364503732|gb|AEW48561.1| NADH dehydrogenase subunit 6 (mitochondrion) [Locusta migratoria] >gi|364503746|gb|AEW48574.1| NADH dehydrogenase subunit 6 (mitochondrion) [Locusta migratoria] >gi|364503760|gb|AEW48587.1| NADH dehydrogenase subunit 6 (mitochondrion) [Locusta migratoria] >gi|364503774|gb|AEW48600.1| NADH dehydrogenase subunit 6 (mitochondrion) [Locusta migratoria] >gi|364503788|gb|AEW48613.1| NADH dehydrogenase subunit 6 (mitochondrion) [Locusta migratoria] >gi|364503802|gb|AEW48626.1| NADH

			dehydrogenase subunit 6 (mitochondrion) [Locusta migratoria] >gi|364503816|gb|AEW48639.1| NADH dehydrogenase subunit 6 (mitochondrion) [Locusta migratoria] >gi|364503830|gb|AEW48652.1| NADH dehydrogenase subunit 6 (mitochondrion) [Locusta migratoria] >gi|364503844|gb|AEW48665.1| NADH dehydrogenase subunit 6 (mitochondrion) [Locusta migratoria] >gi|364503858|gb|AEW48678.1| NADH dehydrogenase subunit 6 (mitochondrion) [Locusta migratoria] >gi|364503872|gb|AEW48691.1| NADH dehydrogenase subunit 6 (mitochondrion) [Locusta migratoria] >gi|364503886|gb|AEW48704.1| NADH dehydrogenase subunit 6 (mitochondrion) [Locusta migratoria] >gi|364503900|gb|AEW48717.1| NADH dehydrogenase subunit 6 (mitochondrion) [Locusta migratoria] >gi|364503914|gb|AEW48730.1| NADH dehydrogenase subunit 6 (mitochondrion) [Locusta migratoria]
Unigene27732_All	-5.0798029	Down	PREDICTED: putative gamma-glutamyltransferase ywrD-like [Takifugu rubripes]
CL56.Contig2_All	-5.16035167	Down	transposase [Salmo salar]
Unigene37736_All	-5.21332794	Down	PREDICTED: aldehyde dehydrogenase, mitochondrial [Ciona intestinalis]
CL7215.Contig1_All	-5.26898234	Down	hexamerin-like protein 2 [Locusta migratoria]
CL7356.Contig2_All	-5.37203041	Down	PREDICTED: lipase member H-A-like [Megachile rotundata]
Unigene21373_All	-5.49905292	Down	hypothetical protein DAPPUDRAFT_307177 [Daphnia pulex]
Unigene34312_All	-5.56828007	Down	PREDICTED: diacylglycerol kinase theta-like isoform 2 [Apis mellifera]
CL1673.Contig5_All	-5.61244464	Down	glucosyl glucuronosyl transferases [Locusta migratoria]
Unigene13202_All	-5.66883884	Down	PREDICTED: similar to argininosuccinate synthetase [Tribolium castaneum] >gi|270001069|gb|EEZ97516.1| hypothetical protein TcasGA2_TC011361 [Tribolium castaneum]
CL2858.Contig3_All	-5.73727649	Down	juvenile hormone esterase-like protein Est1 [Reticulitermes flavipes]
Unigene30032_All	-5.85363348	Down	PREDICTED: regucalcin-like [Bombus impatiens]
Unigene15993_All	-5.8957342	Down	hemocyanin subunit type 2 precursor [Periplaneta americana]
Unigene32223_All	-6.22675902	Down	PREDICTED: similar to putative zinc-containing alcohol dehydrogenase [Tribolium castaneum] >gi|270012005|gb|EFA08453.1| hypothetical protein TcasGA2_TC006100 [Tribolium castaneum]
Unigene17161_All	-6.23204383	Down	hexamerin-like protein 4 [Locusta migratoria]
Unigene33691_All	-6.29462075	Down	glutamine synthetase [Acanthamoeba castellanii str. Neff] >gi|440804153|gb|ELR25031.1| glutamine

			synthetase [Acanthamoeba castellanii str. Neff]
CL2093.Contig1_All	-6.38840768	Down	hemocyanin subunit type 1 [Locusta migratoria manilensis]
Unigene28950_All	-6.42949411	Down	PREDICTED: uncharacterized protein K02A2.6-like [Metaseiulus occidentalis]
CL2093.Contig3_All	-6.55503675	Down	hemocyanin subunit type 2 precursor [Blaptica dubia]
CL607.Contig3_All	-6.66068081	Down	PREDICTED: hypothetical protein LOC100568495 [Acyrthosiphon pisum]
Unigene30487_All	-6.92207641	Down	hypothetical protein AND_00398 [Anopheles darlingi]
CL2165.Contig3_All	-6.92913165	Down	hexamerin-like protein 4 [Locusta migratoria]
Unigene32424_All	-7.52583762	Down	PREDICTED: venom carboxylesterase-6-like [Acyrthosiphon pisum]
CL8832.Contig1_All	-7.85826534	Down	hemocyanin subunit type 2 [Hierodula membranacea]
CL2165.Contig2_All	-8.16762485	Down	hexamerin-like protein 4 [Locusta migratoria]
CL2165.Contig1_All	-8.23230019	Down	hexamerin-like protein 4 [Locusta migratoria]
CL2074.Contig2_All	-8.3979448	Down	PREDICTED: uncharacterized protein K02A2.6-like [Amphimedon queenslandica]
CL7215.Contig2_All	-8.54198435	Down	hexamerin-like protein 2 [Locusta migratoria]
CL1583.Contig3_All	-8.81794297	Down	PREDICTED: GILT-like protein C02D5.2-like [Nasonia vitripennis]
CL2165.Contig5_All	-9.79880399	Down	hexamerin-like protein 4 [Locusta migratoria]
Unigene38711_All	-10.5502658	Down	hypothetical protein BRAFLDRAFT_95006 [Branchiostoma floridae] >gi|229286520|gb|EEN57239.1| hypothetical protein BRAFLDRAFT_95006 [Branchiostoma floridae]
Unigene39754_All	-10.6171919	Down	PREDICTED: sarcosine dehydrogenase, mitochondrial-like [Megachile rotundata]
Unigene40260_All	-10.6773673	Down	AGAP007029-PA [Anopheles gambiae str. PEST] >gi|116132462|gb|EAA03993.4| AGAP007029-PA [Anopheles gambiae str. PEST]
Unigene29078_All	-10.768846	Down	NAD-specific glutamate dehydrogenase [Capsaspora owczarzaki ATCC 30864] >gi|320170749|gb|EFW47648.1| NAD-specific glutamate dehydrogenase [Capsaspora owczarzaki ATCC 30864]
Unigene27815_All	-11.2372697	Down	UDP-glucuronosyltransferase 1-7C [Camponotus floridanus]
CL6572.Contig3_All	-11.4577913	Down	NADH dehydrogenase subunit 1 (mitochondrion) [Locusta migratoria]
Unigene37006_All	-11.5399342	Down	TPA: adenylate kinase 7 [Bos taurus]
Unigene30284_All	-11.740877	Down	PREDICTED: adenylate kinase 7-like [Ciona intestinalis]
Unigene35866_All	-11.7836531	Down	hCG1793893 [Homo sapiens]

Unigene36121_All	-12.0718981	Down	arylphorin hexamerin-like protein 2 [Romalea microptera]
Unigene28125_All	-12.3048932	Down	cytochrome P450 [Aedes aegypti] >gi|108869082|gb|EAT33307.1| AAEL014412-PA [Aedes aegypti]
Unigene34032_All	-12.324715	Down	alpha-esterase 19 isoform 1 precursor [Bombyx mori] >gi|170284246|gb|ACB12411.1| carboxylesterase CarE-8 variant 1 [Bombyx mori]

**Table S4 Differentially expressed peptides identified by LC-ESI-MS/MS.**							
GroupID	Accession	Unique Spectrum	Peptide	Unique Peptide	N_115-VS-D_119	Quant Number	Sig
1	Unigene5951_All	1013	110	110	2.686	829	
2	Unigene14820_All	239	23	23	3.712	204	
3	CL2093.Contig1_All	204	25	21	0.285	150	
4	CL6.Contig2_All	55	17	8	0.898	46	
4	CL6.Contig5_All	55	17	8			
4	CL6.Contig3_All	55	17	8			
5	Unigene12223_All	212	26	26	2.033	198	
6	CL172.Contig1_All	11	17	1	1.489	6	*
6	CL172.Contig3_All	11	17	1			
7	CL6.Contig1_All	15	10	1	1.511	13	*
8	Unigene8462_All	97	14	8	1.168	71	*
9	CL9514.Contig3_All	6	14	3	1.17	4	
10	Unigene2048_All	25	11	2	1.182	25	
11	CL8832.Contig1_All	138	15	13	0.14	70	*
12	Unigene15598_All	135	13	12	2.903	119	*
13	Unigene53825_All	165	9	9	2.338	156	
14	Unigene3365_All	75	16	11	1.116	73	*
15	CL2165.Contig2_All	27	17	5	0.6	21	
16	Unigene4448_All	87	20	17	0.303	71	*
17	CL1310.Contig1_All	40	13	9	0.977	38	
18	CL2165.Contig1_All	12	19	5	0.338	9	*
19	Unigene14809_All	77	24	22	1.03	72	
20	CL9866.Contig3_All	20	10	3	0.901	19	
21	CL9866.Contig1_All	18	10	3	0.823	16	
22	Unigene3379_All	57	20	19	0.991	51	
23	Unigene20610_All	17	6	2	1.236	15	*
24	CL3996.Contig2_All	34	20	10	0.6	33	
25	Unigene2007_All	70	8	8	3.58	66	
26	Unigene8456_All	45	14	10	0.832	36	*
27	Unigene41759_All	1	4	1	---	1	
28	Unigene25192_All	51	4	4	2.881	45	*
29	Unigene9691_All	63	11	11	0.569	56	*
30	CL8144.Contig1_All	42	9	7	0.737	40	*
31	CL5772.Contig3_All	52	23	17	0.561	42	*
32	Unigene15993_All	74	11	11	0.17	45	*
33	Unigene19984_All	51	14	14	0.872	44	*
34	Unigene55442_All	61	4	4	3.629	55	
35	CL179.Contig1_All	7	7	2	0.337	6	*

36	Unigene1720_All	47	6	6	0.662	37	*
37	Unigene12226_All	32	12	12	1.031	31	
38	CL9226.Contig1_All	47	10	10	0.351	47	*
39	Unigene4575_All	28	6	3	0.303	21	*
40	Unigene9725_All	51	11	11	0.39	41	
41	Unigene16305_All	42	5	5	3.341	35	*
42	CL2093.Contig3_All	47	5	4	0.423	42	*
43	Unigene17161_All	54	7	7	0.887	46	
44	CL179.Contig2_All	27	7	2	0.212	22	*
45	Unigene3844_All	30	8	8	1.36	27	
46	Unigene31505_All	22	3	1	2.177	19	*
47	Unigene23412_All	40	11	11	0.801	39	*
48	CL5802.Contig1_All	16	6	6	1.296	15	*
48	CL5802.Contig4_All	16	6	6			
48	CL5802.Contig2_All	16	6	6			
48	CL5802.Contig3_All	16	6	6			
49	CL3996.Contig1_All	6	13	3	1.002	4	
50	CL9168.Contig3_All	16	7	2	0.904	13	
51	Unigene14838_All	39	12	12	0.527	34	*
52	Unigene19990_All	23	6	4	0.938	18	
53	CL1845.Contig2_All	30	8	7	1.938	27	*
53	CL1845.Contig3_All	30	8	7			
54	CL9559.Contig1_All	27	4	4	0.991	26	
54	CL9559.Contig2_All	27	4	4			
55	Unigene19987_All	28	10	8	0.897	27	*
56	Unigene21074_All	18	4	3	0.729	14	*
57	Unigene5991_All	22	5	5	0.905	22	
58	Unigene6729_All	9	4	1	0.758	8	*
59	Unigene19952_All	26	4	4	0.57	23	*
60	Unigene25168_All	25	10	10	0.632	23	*
61	Unigene9631_All	16	7	4	0.699	15	
62	Unigene12194_All	40	11	11	0.772	37	*
63	Unigene13687_All	66	1	1	2.643	47	
64	CL246.Contig1_All	19	3	3	0.526	19	*
65	Unigene20098_All	33	5	5	0.45	22	
66	Unigene14826_All	19	8	8	0.916	14	
67	Unigene7174_All	28	8	8	0.927	21	
68	Unigene17486_All	22	7	6	1.195	20	
69	Unigene3375_All	37	12	12	0.574	27	*
70	Unigene15539_All	27	9	9	0.989	25	
71	CL9777.Contig1_All	14	3	2	1.321	12	*

71	CL9777.Contig2_All	14	3	2			
72	CL2555.Contig1_All	18	7	7	1.073	17	
72	CL2555.Contig2_All	18	7	7			
73	Unigene22597_All	37	11	11	1.385	29	*
74	CL1425.Contig1_All	14	4	4	3.233	12	*
75	CL2865.Contig1_All	12	2	2	0.853	11	*
76	Unigene1874_All	24	6	6	0.743	19	*
77	Unigene9663_All	22	9	9	0.924	19	
78	CL3314.Contig2_All	21	3	3	0.205	16	*
79	CL9833.Contig2_All	2	4	1	---	1	
80	Unigene6413_All	10	3	2	1.096	9	
81	CL4648.Contig1_All	14	4	4	1.116	13	
81	CL4648.Contig3_All	14	4	4			
81	CL4648.Contig5_All	14	4	4			
81	CL4648.Contig2_All	14	4	4			
81	CL4648.Contig6_All	14	4	4			
81	CL4648.Contig4_All	14	4	4			
82	Unigene12285_All	29	6	6	0.849	23	*
83	Unigene4463_All	14	6	6	1.58	13	*
84	Unigene19989_All	18	9	8	0.714	15	*
85	Unigene2824_All	1	4	1	---	1	
86	CL5499.Contig1_All	8	4	4	1.114	8	
86	CL5499.Contig2_All	8	4	4			
87	Unigene9682_All	20	7	7	1.129	18	*
88	Unigene12189_All	19	7	6	0.561	16	*
89	Unigene22575_All	21	7	7	0.928	19	
90	CL2165.Contig3_All	14	5	3	0.735	12	*
91	Unigene12191_All	12	5	2	1.018	10	
92	Unigene14785_All	21	7	6	1.199	18	*
93	Unigene19772_All	18	5	5	1.124	18	*
94	CL2180.Contig1_All	21	3	3	0.539	12	*
95	CL5757.Contig1_All	17	10	10	0.612	15	*
96	Unigene56350_All	12	2	1	0.854	12	*
97	CL9361.Contig1_All	16	4	4	1.419	16	*
98	CL2627.Contig1_All	17	5	5	1.13	16	
99	Unigene56003_All	30	4	4	0.769	20	*
99	Unigene24956_All	30	4	4			
100	Unigene2803_All	14	4	4	0.677	11	
101	CL8895.Contig1_All	18	5	5	0.838	16	
101	CL8895.Contig2_All	18	5	5			
102	CL6074.Contig1_All	22	5	5	0.899	21	

103	Unigene14646_All	17	3	3	0.906	12	*
104	Unigene4405_All	30	5	4	3.669	28	
105	CL2398.Contig1_All	22	5	5	0.736	20	*
105	CL2398.Contig3_All	22	5	5			
106	Unigene9675_All	5	3	2	1.17	5	
107	CL717.Contig1_All	23	6	6	1.102	19	
107	CL717.Contig2_All	23	6	6			
108	Unigene20105_All	12	3	3	1.138	12	*
109	CL5100.Contig1_All	6	3	1	0.52	6	*
110	Unigene22701_All	7	5	4	0.846	5	
111	Unigene32273_All	6	3	1	0.751	6	
112	CL9844.Contig1_All	12	7	5	0.958	11	
113	Unigene25149_All	19	6	6	0.943	16	
114	Unigene9775_All	19	4	4	0.873	12	
115	Unigene4478_All	22	4	4	1.957	21	*
116	Unigene14822_All	14	6	6	0.384	14	*
117	CL1728.Contig1_All	7	3	1	0.66	7	*
117	CL1728.Contig2_All	7	3	1			
118	Unigene26321_All	11	3	3	2.917	10	
119	CL414.Contig1_All	20	9	9	0.991	18	
120	CL7215.Contig1_All	21	6	4	5.192	14	
121	Unigene11986_All	13	3	3	0.372	12	*
122	CL5772.Contig1_All	4	8	4	2.163	4	*
122	CL5772.Contig2_All	4	8	4			
123	Unigene20009_All	16	7	7	1.113	15	
124	Unigene54462_All	6	1	1	0.682	6	*
125	CL8014.Contig1_All	20	8	8	1.035	18	
126	Unigene9666_All	16	8	8	1.156	13	*
127	Unigene22611_All	20	6	6	0.781	17	*
128	CL2791.Contig1_All	19	8	8	0.517	16	*
128	CL2791.Contig3_All	19	8	8			
129	Unigene19944_All	18	8	6	0.794	12	
130	CL1385.Contig1_All	15	5	5	0.884	11	*
131	Unigene4679_All	40	3	3	0.799	18	*
132	CL4336.Contig1_All	13	7	7	0.622	11	*
133	Unigene17372_All	15	4	4	0.556	14	*
134	Unigene53826_All	17	3	3	1.795	13	*
135	Unigene7840_All	10	2	2	0.486	8	*
136	Unigene17390_All	9	6	5	0.967	9	
137	Unigene23968_All	18	8	8	1.129	16	*
138	CL2564.Contig1_All	17	7	7	0.692	13	

139	Unigene17385_All	10	6	6	0.881	10	
140	Unigene22583_All	19	5	5	0.59	15	*
141	Unigene12255_All	15	8	6	0.935	9	
142	Unigene4403_All	15	8	8	0.584	6	*
143	Unigene750_All	16	4	4	2.376	16	*
144	Unigene21051_All	17	4	4	0.633	16	*
145	Unigene22602_All	16	9	9	1.25	12	
146	Unigene17432_All	12	4	3	1.121	11	
147	Unigene1992_All	16	3	3	0.632	12	*
148	Unigene8061_All	12	4	4	0.608	12	*
149	Unigene78770_All	14	2	2	0.537	14	*
150	Unigene25141_All	15	3	2	0.939	12	
151	CL2362.Contig1_All	16	5	5	0.835	14	*
152	Unigene14884_All	1	4	1	---	1	
153	Unigene7105_All	12	3	3	1.006	11	
154	CL2140.Contig1_All	6	6	5	1.205	5	
155	Unigene8547_All	13	7	7	0.311	10	*
156	Unigene16236_All	8	4	4	0.887	7	
157	CL6821.Contig1_All	14	5	5	0.429	11	*
157	CL6821.Contig2_All	14	5	5			
158	Unigene21458_All	6	2	2	1.969	6	*
159	Unigene40226_All	14	3	2	0.709	13	*
160	Unigene4534_All	11	5	5	0.799	11	*
161	CL8762.Contig1_All	12	6	6	0.977	11	
161	CL8762.Contig2_All	12	6	6			
162	Unigene22623_All	7	3	3	0.968	6	
163	CL9829.Contig1_All	9	4	4	0.97	9	
164	CL329.Contig1_All	12	4	4	0.471	11	*
165	Unigene18403_All	7	3	3	1.008	7	
166	Unigene32_All	14	11	11	0.875	11	
167	Unigene37883_All	2	3	2	0.759	2	
168	Unigene58393_All	17	2	2	1.021	14	
169	CL9514.Contig1_All	1	4	1	---		
169	CL9514.Contig2_All	1	4	1			
170	Unigene17160_All	15	3	3	0.826	13	*
171	Unigene9649_All	5	1	1	1.038	5	
171	Unigene10966_All	5	1	1			
172	Unigene16201_All	13	5	5	0.552	9	*
173	Unigene19998_All	8	3	3	0.338	8	*
174	Unigene12190_All	7	3	3	0.802	7	
175	CL1897.Contig3_All	4	2	1	1.393	4	*

176	Unigene21515_All	16	10	9	1.001	12	
177	CL7629.Contig1_All	2	3	2	0.879	2	
178	Unigene344_All	7	3	3	0.784	6	*
179	Unigene5485_All	13	7	7	1.237	9	*
180	Unigene12238_All	8	5	5	0.492	6	*
181	Unigene9470_All	8	2	2	0.926	8	
182	CL2162.Contig1_All	4	4	1	0.904	2	
182	CL2162.Contig3_All	4	4	1			
183	Unigene14645_All	10	3	3	0.963	10	
184	CL7121.Contig1_All	9	4	4	0.663	8	*
185	Unigene19923_All	13	5	5	1.488	13	*
186	Unigene20061_All	9	3	3	0.917	8	
187	Unigene14834_All	8	2	2	0.901	7	
188	Unigene131_All	6	3	3	0.598	6	
189	Unigene23351_All	6	3	3	1.172	6	
190	Unigene9650_All	10	5	5	0.869	9	
191	CL9597.Contig1_All	10	6	6	0.684	7	*
192	Unigene16304_All	11	3	3	0.645	8	*
193	Unigene7044_All	10	2	2	0.653	9	*
194	Unigene24971_All	10	3	3	0.79	8	*
195	Unigene25098_All	11	4	4	0.858	10	
196	CL5398.Contig2_All	17	3	3	0.488	8	*
196	Unigene7031_All	17	3	3			
197	CL4299.Contig1_All	27	1	1	0.424	19	*
197	Unigene55648_All	27	1	1			
197	Unigene56574_All	27	1	1			
197	Unigene15418_All	27	1	1			
197	CL4299.Contig2_All	27	1	1			
198	Unigene31798_All	8	6	2	0.354	8	
199	CL8131.Contig1_All	8	3	3	0.983	6	
200	Unigene19995_All	8	3	3	1.124	7	*
201	Unigene23268_All	8	2	2	1.419	8	*
202	Unigene22559_All	13	7	7	1.021	13	
203	Unigene21404_All	15	4	4	0.269	10	*
204	Unigene22652_All	4	1	1	0.836	4	
205	CL3139.Contig6_All	4	3	1	1.657	4	*
206	Unigene4474_All	14	9	9	0.869	14	
207	Unigene39054_All	13	2	2	6.793	10	*
208	Unigene47117_All	3	2	1	---	1	
209	CL9084.Contig1_All	11	3	3	0.77	11	*
209	CL9084.Contig3_All	11	3	3			

209	CL9084.Contig2_All	11	3	3			
210	CL2655.Contig1_All	9	3	3	1.299	9	
210	CL2655.Contig2_All	9	3	3			
211	Unigene25255_All	9	4	4	1.054	7	
212	Unigene25259_All	8	5	4	1.196	7	
213	CL6278.Contig2_All	10	3	3	1.054	7	
213	CL6278.Contig3_All	10	3	3			
214	CL6507.Contig1_All	10	3	3	0.765	9	*
214	CL6507.Contig2_All	10	3	3			
215	Unigene8580_All	8	5	5	0.929	6	
216	Unigene17410_All	5	1	1	1.061	5	
217	CL9821.Contig1_All	14	6	6	1.045	13	
218	Unigene37239_All	10	1	1	0.481	7	*
219	Unigene23991_All	8	4	4	1.022	8	
220	Unigene4410_All	7	3	3	0.825	7	*
221	Unigene17453_All	6	3	3	1.045	6	
222	CL1226.Contig2_All	4	1	1	0.779	4	
222	CL4164.Contig1_All	4	1	1			
223	Unigene23627_All	7	4	4	0.683	7	*
224	Unigene22568_All	4	6	4	0.732	2	
225	Unigene2052_All	10	3	3	0.724	8	*
226	CL6745.Contig1_All	7	3	3	0.819	6	*
226	CL6745.Contig2_All	7	3	3			
227	Unigene4460_All	10	6	6	0.861	9	
228	Unigene19992_All	10	2	2	0.91	8	
229	Unigene16240_All	8	6	6	1.124	6	
230	CL6106.Contig1_All	9	4	4	1.094	8	
231	CL9095.Contig1_All	8	2	2	0.78	7	*
232	CL6381.Contig2_All	6	4	4	0.733	5	
233	Unigene19970_All	6	1	1	0.677	5	
234	Unigene6838_All	14	5	5	1.036	6	
235	CL8322.Contig1_All	6	2	2	0.828	5	
236	CL5246.Contig1_All	8	3	3	1.222	5	*
236	CL5246.Contig2_All	8	3	3			
237	Unigene759_All	5	3	3	1.038	4	
238	Unigene77620_All	2	2	1	1.088	2	
239	Unigene40305_All	4	3	2	0.809	4	
240	Unigene25144_All	7	4	4	1.027	7	
241	Unigene17459_All	9	3	3	0.982	9	
242	CL7654.Contig1_All	7	3	3	1.016	6	
242	CL7654.Contig2_All	7	3	3			

243	Unigene1948_All	1	4	1	---	1	
244	CL1352.Contig1_All	7	4	4	0.942	7	
244	CL1352.Contig2_All	7	4	4			
245	CL1473.Contig2_All	12	6	6	0.737	11	
245	CL1473.Contig3_All	12	6	6			
246	CL5191.Contig1_All	5	2	2	1.443	5	
247	Unigene20110_All	10	4	4	0.899	10	*
248	Unigene4574_All	3	3	3	1.192	3	
249	CL8649.Contig1_All	9	6	6	0.269	9	*
250	CL4168.Contig1_All	9	6	6	0.97	6	
251	CL2947.Contig3_All	6	3	3	0.615	6	*
251	CL2947.Contig5_All	6	3	3			
251	CL2947.Contig4_All	6	3	3			
251	CL2947.Contig6_All	6	3	3			
252	Unigene25176_All	7	4	4	1.385	4	*
253	CL3561.Contig1_All	9	4	4	0.858	6	*
254	CL2040.Contig1_All	3	4	2	0.8	2	
255	Unigene25252_All	7	3	3	0.822	7	
256	Unigene14627_All	1	2	1	---	1	
257	CL5383.Contig2_All	4	2	1	0.903	4	*
258	Unigene61997_All	13	1	1	0.614	11	*
259	Unigene15870_All	10	3	3	2.16	7	*
260	Unigene17399_All	7	4	4	1.072	6	
261	CL2761.Contig1_All	5	2	2	0.966	4	
262	Unigene26269_All	5	2	2	0.659	5	
263	Unigene12052_All	8	3	3	0.949	7	
264	Unigene22594_All	4	1	1	0.889	4	
265	Unigene9617_All	7	5	5	1.025	6	
266	Unigene25112_All	11	4	4	0.469	10	
267	CL2581.Contig1_All	11	4	4	1.373	6	
267	Unigene19937_All	11	4	4			
267	CL2581.Contig2_All	11	4	4			
268	Unigene18890_All	8	2	2	0.94	8	
269	Unigene5336_All	5	1	1	0.775	5	*
270	CL3440.Contig1_All	6	2	2	0.611	6	*
271	Unigene17405_All	7	4	4	1.044	5	
272	Unigene61138_All	6	2	2	2.614	5	*
273	Unigene4491_All	8	4	4	0.875	8	
274	CL7185.Contig1_All	7	6	6	1.105	7	
275	Unigene17493_All	13	5	5	0.913	9	
276	CL4736.Contig3_All	6	2	2	0.434	6	*

277	Unigene7019_All	7	2	2	0.801	6	*
278	CL6262.Contig1_All	4	3	3	0.926	4	
279	Unigene23376_All	5	1	1	1.518	5	*
280	Unigene15836_All	5	3	3	1.132	4	
281	Unigene7075_All	4	1	1	0.962	4	
282	CL2319.Contig1_All	5	3	3	1.046	3	*
283	Unigene19936_All	4	1	1	0.282	4	*
284	CL2947.Contig1_All	5	2	2	2.924	5	*
284	CL2947.Contig2_All	5	2	2			
285	Unigene25985_All	8	3	3	0.485	7	*
286	CL4348.Contig1_All	3	2	2	1.174	3	
287	CL643.Contig1_All	11	4	4	0.697	8	*
287	CL643.Contig2_All	11	4	4			
288	Unigene34602_All	6	2	2	1.012	6	
289	Unigene7154_All	1	4	1	---	1	
290	Unigene780_All	8	5	5	0.938	7	
291	Unigene4452_All	10	5	5	0.725	5	
292	Unigene21516_All	5	1	1	1.096	5	
293	Unigene12208_All	5	2	2	0.672	5	*
294	Unigene57126_All	6	1	1	0.705	5	*
295	CL3905.Contig1_All	9	2	2	1.173	8	
296	Unigene4245_All	3	1	1	0.895	3	
297	Unigene14821_All	5	4	4	1.102	4	
298	Unigene7122_All	9	3	3	1.031	8	
299	Unigene19926_All	4	3	3	1.016	4	
300	Unigene6850_All	6	1	1	1.115	6	*
301	CL6897.Contig1_All	8	3	3	0.99	8	
302	Unigene7085_All	2	3	1	1.184	2	
303	Unigene13396_All	11	1	1	0.878	8	*
304	Unigene12218_All	10	6	6	0.601	4	
305	Unigene4416_All	16	6	6	0.311	11	*
306	CL9216.Contig1_All	5	1	1	2.747	5	
306	CL9216.Contig2_All	5	1	1			
307	CL2903.Contig1_All	4	2	2	1.233	3	*
308	CL6760.Contig1_All	5	4	2	0.767	5	
309	Unigene12176_All	4	4	3	1.115	4	
310	Unigene8099_All	10	4	4	0.82	10	
311	Unigene24099_All	8	2	2	0.892	8	
312	Unigene22928_All	3	1	1	1.589	3	*
313	CL2415.Contig1_All	4	1	1	0.594	4	*
314	CL4537.Contig2_All	7	2	2	0.307	5	*

315	Unigene22610_All	5	3	3	0.997	5	
316	Unigene9668_All	8	6	5	1.306	8	
317	Unigene4402_All	8	3	3	0.393	6	*
318	Unigene7103_All	3	1	1	0.685	3	
319	Unigene3396_All	5	2	2	0.893	5	
320	CL586.Contig1_All	5	2	2	0.742	5	
321	CL3669.Contig2_All	3	3	2	0.644	3	
322	Unigene11158_All	8	4	4	0.496	8	*
323	Unigene9762_All	5	3	3	2.326	5	*
324	Unigene17394_All	7	3	3	0.933	7	
325	Unigene1930_All	5	4	4	0.904	4	
326	CL1161.Contig1_All	4	2	2	0.61	4	*
326	Unigene14778_All	4	2	2			
327	Unigene17371_All	6	3	3	1.128	4	
328	Unigene72_All	4	2	2	0.755	3	*
329	Unigene9629_All	6	4	4	0.804	4	
330	CL1931.Contig1_All	2	5	2	0.799	2	
331	Unigene14913_All	7	1	1	0.549	7	*
332	Unigene22626_All	5	3	3	0.745	3	*
333	CL9420.Contig2_All	7	4	4	0.894	4	
334	Unigene16295_All	5	3	3	0.787	5	
335	Unigene23476_All	3	2	2	0.92	2	
336	CL7191.Contig1_All	6	2	2	0.883	4	*
337	CL1146.Contig1_All	3	2	1	0.586	3	
338	CL3110.Contig1_All	6	3	3	0.755	5	*
339	CL1926.Contig1_All	9	5	5	1.296	9	*
339	CL1926.Contig3_All	9	5	5			
339	CL1926.Contig2_All	9	5	5			
339	CL1926.Contig6_All	9	5	5			
339	CL1926.Contig4_All	9	5	5			
339	CL1926.Contig7_All	9	5	5			
339	CL1926.Contig8_All	9	5	5			
339	CL1926.Contig5_All	9	5	5			
340	Unigene17341_All	5	4	4	0.864	3	
341	Unigene2037_All	4	2	1	0.951	4	
342	Unigene356_All	6	4	4	0.896	4	
343	Unigene7039_All	3	2	2	0.865	3	
344	Unigene4583_All	3	2	2	1.067	3	
345	Unigene24953_All	4	1	1	0.139	3	
346	Unigene2786_All	6	3	3	0.707	4	
347	CL9134.Contig1_All	2	1	1	1.101	2	

348	Unigene25151_All	6	2	2	0.443	5	*
349	CL565.Contig1_All	3	1	1	1.052	3	
349	CL565.Contig2_All	3	1	1			
350	Unigene23383_All	10	4	4	0.485	4	*
351	Unigene9765_All	6	3	3	0.936	4	
352	Unigene24098_All	4	2	2	0.747	3	*
353	CL3871.Contig3_All	4	3	3	0.843	3	
354	CL3877.Contig1_All	4	2	2	0.907	2	
355	CL6613.Contig1_All	5	5	4	0.9	4	
356	Unigene14588_All	6	2	2	1.142	6	
357	CL4268.Contig1_All	5	3	3	0.603	4	*
357	CL4268.Contig2_All	5	3	3			
358	CL3836.Contig1_All	3	3	3	1.435	3	
359	Unigene12177_All	6	3	3	1.019	4	
360	Unigene12254_All	5	2	2	0.621	4	*
361	Unigene23762_All	7	3	3	2.653	4	*
362	Unigene4420_All	5	2	2	0.501	4	*
363	Unigene22548_All	3	2	2	1.001	3	
364	Unigene25237_All	3	2	2	0.78	3	*
365	CL8137.Contig1_All	3	2	2	0.467	3	
365	CL8137.Contig2_All	3	2	2			
366	Unigene20099_All	6	4	4	0.59	5	
367	CL5539.Contig1_All	3	1	1	1.842	2	
368	Unigene2650_All	5	1	1	1.229	5	
369	CL9671.Contig1_All	11	3	3	0.689	6	*
369	Unigene15241_All	11	3	3			
370	Unigene14946_All	1	1	1	---	1	
371	Unigene25234_All	4	2	2	0.511	4	*
372	CL2826.Contig2_All	4	1	1	1.936	4	*
373	CL8639.Contig2_All	6	4	4	1.4	6	
374	Unigene3592_All	11	2	2	0.794	3	
375	Unigene9697_All	3	1	1	1.616	3	*
376	Unigene4556_All	3	1	1	0.701	2	
377	Unigene9440_All	7	3	3	0.81	4	*
378	Unigene6800_All	5	2	2	1.832	5	*
379	Unigene7549_All	5	2	2	0.245	3	*
380	CL6869.Contig1_All	5	3	3	1.22	5	*
381	Unigene12206_All	7	3	3	1.053	6	
382	CL596.Contig1_All	3	3	3	1.04	3	
383	Unigene22601_All	6	4	4	1.212	4	
384	CL1031.Contig2_All	4	2	2	0.744	3	

384	CL1031.Contig3_All	4	2	2			
385	Unigene23265_All	25	1	1	2.961	25	*
386	CL5078.Contig1_All	2	1	1	1.517	2	
386	CL5078.Contig2_All	2	1	1			
387	Unigene70281_All	7	1	1	0.57	3	
388	CL7636.Contig1_All	2	2	2	---	1	
388	CL7636.Contig2_All	2	2	2			
389	Unigene12331_All	4	3	3	1.012	3	
390	Unigene14232_All	12	1	1	5.455	12	
391	CL6181.Contig1_All	2	2	2	1.229	2	
391	CL6181.Contig2_All	2	2	2			
392	Unigene69984_All	2	2	1	0.857	2	
393	CL2426.Contig1_All	5	3	3	0.995	4	
394	CL4744.Contig1_All	4	2	2	0.965	4	
394	CL4744.Contig2_All	4	2	2			
395	Unigene22558_All	3	3	3	1.199	3	
396	CL929.Contig1_All	8	3	3	0.411	4	*
397	CL3316.Contig4_All	5	4	4	0.984	2	
397	CL3316.Contig6_All	5	4	4			
397	CL3316.Contig7_All	5	4	4			
398	Unigene9681_All	4	3	3	1.045	4	
399	CL378.Contig2_All	3	1	1	0.953	3	
399	CL378.Contig3_All	3	1	1			
400	CL8570.Contig1_All	6	3	3	0.937	6	
400	CL8570.Contig2_All	6	3	3			
401	Unigene7063_All	5	2	2	0.821	2	
402	Unigene1880_All	4	3	3	0.932	3	
403	Unigene9772_All	5	2	2	1.092	5	
404	Unigene7069_All	4	1	1	1.475	2	
405	CL3826.Contig1_All	3	2	2	1.03	3	
406	Unigene11049_All	2	2	2	1.038	2	
407	Unigene13552_All	5	1	1	0.879	2	
407	Unigene40491_All	5	1	1			
408	Unigene15800_All	2	3	2	1.157	2	
409	Unigene23965_All	5	3	3	1.578	3	
410	Unigene4461_All	3	1	1	0.74	3	
411	CL3077.Contig1_All	7	3	3	1.129	4	
412	CL9363.Contig1_All	3	3	1	1.106	2	
413	CL5476.Contig1_All	5	3	3	1.237	3	*
414	Unigene76_All	6	3	3	1.248	4	*
414	CL4504.Contig1_All	6	3	3			

414	CL4504.Contig5_All	6	3	3			
414	CL4504.Contig4_All	6	3	3			
415	CL6523.Contig1_All	5	2	2	0.698	5	*
416	CL2373.Contig1_All	7	5	5	0.563	5	
416	CL2373.Contig2_All	7	5	5			
417	CL3251.Contig1_All	3	2	2	1.365	2	
417	CL3251.Contig2_All	3	2	2			
418	Unigene25254_All	3	1	1	1.337	3	*
419	Unigene380_All	6	2	2	1.102	3	
420	Unigene15518_All	3	2	2	3.975	3	
421	Unigene20087_All	2	2	1	0.93	2	
422	Unigene15872_All	5	2	2	0.71	5	
423	Unigene16293_All	4	2	2	1.218	3	
424	CL9117.Contig3_All	7	2	2	0.255	7	*
425	Unigene1766_All	8	4	4	1.204	5	
426	Unigene14815_All	3	3	3	0.518	3	
427	Unigene22700_All	8	4	4	0.88	4	*
428	Unigene8155_All	2	1	1	1.477	2	
429	Unigene6923_All	3	1	1	1.23	2	
430	CL78.Contig2_All	5	2	2	1.148	5	
431	CL502.Contig1_All	3	1	1	1.243	2	
432	Unigene4519_All	4	1	1	0.714	3	
433	Unigene9722_All	3	1	1	0.983	3	
434	Unigene8592_All	3	2	2	0.476	3	*
435	Unigene14775_All	4	2	2	0.634	3	*
436	Unigene12956_All	1	1	1	---	1	
437	Unigene5797_All	4	1	1	0.638	3	*
437	CL9407.Contig1_All	4	1	1			
437	Unigene13754_All	4	1	1			
437	Unigene7014_All	4	1	1			
438	CL2839.Contig1_All	2	2	2	0.789	2	*
439	CL7340.Contig1_All	2	1	1	1.08	2	
439	CL7340.Contig3_All	2	1	1			
439	CL7340.Contig2_All	2	1	1			
440	CL6876.Contig1_All	5	2	2	1.121	5	
441	CL5429.Contig1_All	2	2	2	0.454	2	
442	Unigene12186_All	6	2	2	0.925	5	
443	Unigene13708_All	3	4	3	---	1	
444	Unigene1923_All	8	4	4	0.623	5	*
445	Unigene13724_All	3	2	2	1.323	3	
446	CL64.Contig1_All	4	1	1	0.475	3	*

447	Unigene26811_All	1	2	1	---	1	
448	Unigene22698_All	5	3	3	0.853	3	
449	CL1988.Contig2_All	3	3	2	5.121	2	*
450	Unigene13727_All	2	1	1	1.147	2	
451	Unigene9664_All	3	2	2	0.626	3	
452	Unigene23308_All	6	2	2	0.894	2	
453	CL7327.Contig2_All	8	1	1	0.999	7	
454	CL9416.Contig1_All	3	4	3	0.766	3	
455	Unigene20001_All	4	3	3	0.81	2	
456	Unigene12354_All	2	1	1	0.991	2	
457	Unigene4428_All	4	2	2	0.809	3	
458	Unigene25177_All	2	2	2	---	1	
459	Unigene40368_All	2	1	1	1.535	2	
460	Unigene4456_All	6	2	2	0.919	6	
461	Unigene15848_All	4	3	3	0.846	3	
462	CL9485.Contig1_All	1	2	1	---	1	
462	Unigene10322_All	1	2	1			
463	CL4675.Contig1_All	2	1	1	0.339	2	*
463	CL4675.Contig2_All	2	1	1			
464	Unigene16299_All	1	1	1	---	1	
465	Unigene74730_All	2	1	1	0.772	2	
466	Unigene21405_All	2	2	2	1.531	2	
467	Unigene12273_All	7	2	2	3.18	7	
468	CL7292.Contig1_All	4	3	3	1.207	2	
469	CL7264.Contig2_All	4	2	2	1.785	4	
469	CL7264.Contig3_All	4	2	2			
470	Unigene9643_All	2	2	2	1.008	2	
471	Unigene7060_All	2	2	2	1.617	2	
472	Unigene22569_All	4	3	3	1.155	3	
473	Unigene7159_All	2	2	2	2.025	2	*
474	Unigene10692_All	3	2	2	0.703	3	*
475	Unigene45646_All	5	2	2	7.311	5	*
476	Unigene10471_All	2	2	1	2.439	2	*
477	Unigene5144_All	7	4	4	1.07	4	
478	CL290.Contig1_All	4	3	3	0.891	3	
478	Unigene7144_All	4	3	3			
479	Unigene13143_All	4	3	3	1.108	3	
480	CL426.Contig1_All	4	2	2	0.448	4	*
480	CL426.Contig2_All	4	2	2			
481	CL3687.Contig3_All	2	3	2	---	1	
482	Unigene13173_All	1	1	1	---	1	

483	Unigene5358_All	1	1	1	---	1	
484	Unigene4503_All	6	2	2	0.857	4	
485	Unigene19948_All	2	1	1	0.75	2	
486	Unigene25134_All	3	1	1	1.208	3	
487	Unigene25155_All	3	1	1	0.73	3	
488	Unigene13038_All	2	2	2	---	1	
489	Unigene12022_All	1	1	1	---	1	
490	Unigene17381_All	2	1	1	1.123	2	
491	Unigene18041_All	2	1	1	1.502	2	
492	Unigene7178_All	1	1	1	---	1	
493	Unigene5973_All	4	2	2	0.584	3	*
494	Unigene9635_All	3	1	1	0.634	3	*
495	Unigene19930_All	2	2	2	0.934	2	
496	Unigene21522_All	4	3	3	0.774	2	
497	CL1643.Contig1_All	3	3	3	0.774	2	
498	Unigene2021_All	3	2	1	0.703	2	
499	Unigene25116_All	2	1	1	0.988	2	
500	Unigene5372_All	4	2	2	1.259	3	
501	CL1606.Contig1_All	1	3	1	---		
501	CL1606.Contig4_All	1	3	1			
502	Unigene12989_All	3	1	1	1.364	3	*
503	CL5563.Contig1_All	4	3	3	1.176	4	*
503	CL5563.Contig2_All	4	3	3			
504	Unigene22595_All	4	2	2	0.987	2	
505	Unigene229_All	3	2	2	0.616	3	
506	Unigene9760_All	2	2	2	1.121	2	
507	CL267.Contig1_All	2	1	1	0.913	2	
507	CL9756.Contig3_All	2	1	1			
507	CL267.Contig2_All	2	1	1			
507	CL9756.Contig2_All	2	1	1			
507	Unigene2041_All	2	1	1			
508	Unigene13138_All	2	1	1	0.556	2	*
509	CL1485.Contig1_All	2	1	1	0.668	2	*
509	CL1485.Contig2_All	2	1	1			
510	Unigene15834_All	4	2	2	1.204	4	
511	Unigene23626_All	3	3	3	0.781	3	
512	Unigene2001_All	2	1	1	0.923	2	
513	CL1543.Contig1_All	5	1	1	0.3	5	*
513	CL1543.Contig2_All	5	1	1			
514	Unigene11787_All	4	3	2	0.277	3	*
515	Unigene39880_All	1	2	1	---	1	

516	Unigene7167_All	1	1	1	---	1	
517	Unigene13318_All	2	1	1	1.166	2	
518	Unigene29733_All	3	1	1	0.538	2	
519	CL2165.Contig5_All	7	2	2	0.388	3	*
520	Unigene8089_All	3	3	3	1.305	3	
521	Unigene1929_All	2	2	2	0.966	2	
522	Unigene18850_All	3	2	2	1.092	3	
523	Unigene5292_All	2	1	1	0.923	2	
524	Unigene79765_All	5	1	1	---	1	
525	Unigene18440_All	1	1	1	---	1	
526	Unigene20064_All	3	2	2	1.255	2	
527	CL2059.Contig1_All	2	3	2	0.667	2	
528	CL5448.Contig1_All	3	1	1	3.074	2	
528	CL5448.Contig2_All	3	1	1			
529	Unigene5247_All	2	2	2	---	1	
530	Unigene12987_All	3	3	3	0.682	2	
531	Unigene6884_All	3	2	2	0.803	2	
532	Unigene43565_All	7	1	1	2.801	7	*
533	Unigene29395_All	3	1	1	3.333	3	*
534	CL3800.Contig2_All	2	2	2	0.838	2	*
534	CL3800.Contig3_All	2	2	2			
535	CL9211.Contig1_All	1	1	1	---	1	
536	CL541.Contig2_All	2	1	1	0.921	2	
536	Unigene22607_All	2	1	1			
537	CL4259.Contig2_All	3	1	1	0.715	3	
538	Unigene64614_All	3	2	2	0.489	3	
539	Unigene18823_All	2	2	2	0.627	2	
540	CL1936.Contig1_All	1	2	1	---	1	
541	Unigene18811_All	2	2	2	0.94	2	
542	CL7935.Contig1_All	3	1	1	0.946	3	
542	CL7935.Contig2_All	3	1	1			
543	Unigene7027_All	2	1	1	---	1	
544	Unigene4467_All	4	2	2	1.032	4	
545	Unigene13003_All	1	1	1	---	1	
546	CL8569.Contig1_All	4	2	2	1.049	4	
547	CL5201.Contig1_All	3	1	1	0.266	3	
547	CL5201.Contig2_All	3	1	1			
548	Unigene7722_All	1	1	1	---	1	
549	Unigene14611_All	1	1	1	---	1	
550	Unigene1947_All	5	4	4	0.872	3	
551	Unigene11042_All	4	2	2	0.885	4	

552	Unigene13680_All	2	1	1	0.443	2	
553	Unigene23561_All	2	2	2	1.187	2	
554	Unigene57650_All	6	1	1	---	1	
555	Unigene721_All	2	2	2	---	1	
556	Unigene18817_All	3	2	2	0.555	3	
557	Unigene25281_All	5	2	2	1.122	3	
558	CL2093.Contig2_All	1	1	1	---	1	
559	CL3169.Contig1_All	1	1	1	---	1	
559	CL3169.Contig2_All	1	1	1			
560	Unigene38965_All	4	3	3	2.214	3	*
561	CL2082.Contig3_All	2	1	1	0.422	2	
562	Unigene36765_All	2	1	1	---	1	
563	Unigene5800_All	3	1	1	2.342	3	*
564	CL7510.Contig1_All	5	2	2	0.938	5	
565	Unigene17097_All	2	1	1	0.943	2	
566	CL2716.Contig1_All	2	1	1	0.674	2	
566	CL2716.Contig2_All	2	1	1			
567	Unigene17466_All	2	1	1	0.833	2	
568	Unigene17348_All	2	1	1	0.927	2	
569	CL9694.Contig1_All	2	2	2	1.006	2	
570	Unigene10351_All	4	2	2	1.388	4	*
571	Unigene19942_All	3	2	2	1.344	3	*
572	Unigene13202_All	1	1	1	---	1	
573	CL4068.Contig1_All	4	2	2	1.262	4	
574	CL90.Contig1_All	3	1	1	0.666	3	*
575	Unigene5877_All	4	2	2	1.116	2	
576	Unigene14811_All	3	2	2	1.205	2	
577	Unigene14816_All	4	2	2	0.344	2	
578	Unigene12312_All	2	1	1	0.786	2	
579	Unigene25174_All	1	1	1	---	1	
580	CL1584.Contig1_All	3	3	3	0.585	3	
580	Unigene13674_All	3	3	3			
580	CL1584.Contig2_All	3	3	3			
581	Unigene14277_All	1	1	1	---	1	
582	Unigene78957_All	4	1	1	---		
583	CL5058.Contig1_All	2	1	1	0.379	2	
584	Unigene9770_All	2	3	2	1.174	2	
585	CL505.Contig4_All	3	1	1	0.674	3	
586	Unigene17383_All	2	2	2	---	1	
587	Unigene1891_All	2	1	1	1.585	2	
588	Unigene24933_All	5	4	4	0.744	3	

589	Unigene11137_All	2	2	2	0.853	2	
590	CL6811.Contig2_All	1	1	1	---	1	
591	Unigene2019_All	1	2	1	---	1	
592	Unigene1689_All	3	2	2	0.844	3	
593	Unigene9603_All	1	1	1	---	1	
594	Unigene2255_All	1	1	1	---	1	
595	Unigene7036_All	2	1	1	1.125	2	
596	Unigene873_All	2	2	2	1.223	2	
597	Unigene10412_All	1	1	1	---	1	
598	Unigene17426_All	3	1	1	0.534	3	
599	CL9363.Contig2_All	2	2	1	---	1	
600	CL5674.Contig1_All	2	1	1	---	1	
600	CL5674.Contig2_All	2	1	1			
601	Unigene10672_All	3	1	1	1.826	3	*
602	Unigene26039_All	1	2	1	---	1	
603	Unigene7059_All	2	1	1	0.218	2	
604	CL7183.Contig1_All	3	3	3	---	1	
605	Unigene5198_All	1	1	1	---	1	
606	CL6846.Contig2_All	1	1	1	---	1	
607	Unigene22800_All	4	2	2	0.235	3	*
608	Unigene7920_All	3	2	2	---	1	
609	CL4074.Contig2_All	2	2	2	0.978	2	
610	Unigene5197_All	1	1	1	---	1	
611	Unigene14555_All	2	1	1	---	1	
612	CL9532.Contig1_All	3	1	1	0.484	3	
613	CL3823.Contig1_All	1	1	1	---	1	
613	CL3823.Contig3_All	1	1	1			
613	CL3823.Contig2_All	1	1	1			
614	CL5638.Contig3_All	6	5	5	0.575	3	*
615	Unigene7766_All	2	1	1	1.37	2	*
616	Unigene12181_All	2	2	2	0.6	2	
617	Unigene7029_All	4	1	1	0.871	4	
618	CL1961.Contig1_All	3	1	1	0.754	3	
619	CL50.Contig1_All	3	2	2	---	1	
620	Unigene19928_All	4	1	1	1.371	4	
621	Unigene7084_All	2	1	1	0.77	2	
622	CL6373.Contig1_All	3	2	2	1.356	2	
623	CL9670.Contig1_All	2	2	2	0.853	2	
624	CL639.Contig1_All	2	1	1	1.228	2	
624	CL639.Contig2_All	2	1	1			
625	CL4663.Contig1_All	3	2	2	0.788	3	

626	CL902.Contig2_All	3	1	1	---	1	
627	Unigene17403_All	2	1	1	1.643	2	
628	CL7405.Contig1_All	1	1	1	---	1	
629	Unigene13383_All	1	1	1	---	1	
629	Unigene17346_All	1	1	1			
630	CL6573.Contig1_All	2	1	1	0.602	2	
631	Unigene18233_All	2	1	1	1.349	2	*
632	Unigene24928_All	2	1	1	1.162	2	
633	Unigene12235_All	1	1	1	---	1	
634	Unigene8104_All	2	1	1	1.075	2	
635	Unigene13345_All	4	1	1	0.516	4	
636	CL2055.Contig1_All	1	1	1	---	1	
637	Unigene23475_All	1	1	1	---	1	
638	CL8680.Contig1_All	2	2	2	0.802	2	*
638	CL8680.Contig2_All	2	2	2			
639	Unigene20798_All	2	2	2	0.971	2	
640	Unigene20153_All	2	1	1	0.938	2	
641	Unigene11083_All	1	1	1	---	1	
642	CL5001.Contig1_All	2	1	1	0.991	2	
643	Unigene25160_All	3	2	2	0.818	3	
644	Unigene17332_All	2	1	1	1.121	2	
645	Unigene14828_All	1	1	1	---	1	
646	Unigene12037_All	3	2	2	1.117	3	
647	Unigene11116_All	2	2	2	---	1	
648	CL3076.Contig1_All	3	1	1	0.75	3	*
649	CL4632.Contig1_All	2	2	2	0.696	2	
650	CL4924.Contig1_All	2	1	1	1.103	2	
650	CL4924.Contig2_All	2	1	1			
651	Unigene8440_All	1	1	1	---	1	
652	CL8530.Contig1_All	1	1	1	---	1	
653	Unigene25216_All	2	2	2	0.779	2	*
654	Unigene59804_All	2	1	1	---		
655	Unigene20903_All	2	1	1	2.406	2	
656	Unigene5103_All	2	1	1	11.211	2	*
657	Unigene2035_All	1	1	1	---	1	
658	CL8811.Contig1_All	1	1	1	---	1	
658	CL8811.Contig2_All	1	1	1			
659	Unigene24045_All	1	2	1	---	1	
660	Unigene20065_All	2	1	1	---	1	
661	Unigene56_All	2	1	1	---	1	
662	CL8773.Contig1_All	1	1	1	---	1	

663	Unigene11149_All	1	1	1	---	1	
664	Unigene7139_All	2	2	2	0.964	2	
665	CL9028.Contig1_All	1	1	1	---	1	
665	CL9028.Contig2_All	1	1	1			
666	CL193.Contig1_All	1	1	1	---	1	
666	CL193.Contig3_All	1	1	1			
666	CL193.Contig2_All	1	1	1			
667	CL7615.Contig2_All	1	1	1	---	1	
668	Unigene36258_All	3	3	2	5.807	3	*
669	Unigene25236_All	2	2	2	1.313	2	
670	CL3373.Contig1_All	2	2	2	1.065	2	
670	CL3373.Contig4_All	2	2	2			
670	CL3373.Contig3_All	2	2	2			
670	CL3373.Contig2_All	2	2	2			
671	Unigene22838_All	2	1	1	0.562	2	*
672	CL713.Contig2_All	2	2	2	---	1	
672	CL713.Contig4_All	2	2	2			
673	CL620.Contig1_All	4	1	1	0.65	2	*
674	Unigene4267_All	3	2	2	0.758	2	*
675	CL2543.Contig4_All	2	2	2	0.893	2	
675	Unigene18236_All	2	2	2			
676	Unigene2016_All	2	1	1	---	1	
676	CL6794.Contig2_All	2	1	1			
676	CL6794.Contig1_All	2	1	1			
677	Unigene17350_All	1	1	1	---	1	
678	Unigene2638_All	2	2	2	---	1	
679	CL7674.Contig1_All	3	1	1	---		
679	Unigene41770_All	3	1	1			
679	Unigene79545_All	3	1	1			
680	Unigene43932_All	4	1	1	---		
681	CL4908.Contig1_All	2	2	2	0.994	2	
682	CL8264.Contig1_All	2	1	1	0.977	2	
683	Unigene14812_All	3	2	2	0.526	2	*
684	CL8885.Contig1_All	1	1	1	---	1	
685	Unigene16311_All	3	2	2	0.978	3	
686	Unigene20892_All	1	2	1	---	1	
687	Unigene51240_All	7	1	1	2.738	7	*
688	Unigene14568_All	1	2	1	---		
689	Unigene18931_All	4	2	2	0.205	4	*
690	Unigene4462_All	2	2	2	---	1	
691	Unigene12230_All	2	1	1	1.324	2	

692	CL5557.Contig2_All	1	1	1	---	1	
692	CL9342.Contig2_All	1	1	1			
693	Unigene9020_All	2	1	1	0.401	2	*
694	Unigene12137_All	1	1	1	---	1	
695	CL3944.Contig1_All	1	1	1	---	1	
696	Unigene12239_All	3	2	2	---	1	
697	Unigene4179_All	3	1	1	---	1	
698	CL3139.Contig3_All	3	2	2	---	1	
699	Unigene4514_All	2	2	2	1	2	
700	Unigene25735_All	1	1	1	---	1	
701	Unigene19790_All	2	2	2	0.826	2	
702	CL5462.Contig1_All	1	1	1	---	1	
702	CL5462.Contig2_All	1	1	1			
703	CL1759.Contig2_All	2	1	1	0.559	2	*
704	CL7548.Contig1_All	3	2	2	0.981	2	
704	CL7548.Contig2_All	3	2	2			
705	Unigene2047_All	1	1	1	---	1	
706	Unigene4555_All	3	2	2	---	1	
707	Unigene21415_All	1	1	1	---	1	
708	Unigene7153_All	2	2	2	---	1	
709	Unigene23329_All	2	1	1	0.472	2	*
710	CL3512.Contig1_All	2	2	2	---	1	
711	CL8111.Contig1_All	2	1	1	---	1	
712	CL2810.Contig1_All	1	1	1	---	1	
712	CL2810.Contig2_All	1	1	1			
713	Unigene769_All	1	1	1	---	1	
714	Unigene14839_All	1	1	1	---	1	
715	Unigene8114_All	2	1	1	---	1	
716	Unigene57054_All	2	1	1	---	1	
717	CL8480.Contig1_All	2	2	2	---	1	
717	CL8480.Contig2_All	2	2	2			
718	Unigene25229_All	1	1	1	---	1	
719	CL333.Contig1_All	2	1	1	---	1	
720	CL2997.Contig1_All	1	1	1	---	1	
720	CL2997.Contig3_All	1	1	1			
720	CL2997.Contig2_All	1	1	1			
721	Unigene25099_All	3	2	2	---	1	
722	Unigene18761_All	1	1	1	---	1	
723	Unigene42395_All	1	1	1	---	1	
724	Unigene13756_All	1	1	1	---	1	
725	CL6092.Contig1_All	2	1	1	0.832	2	

725	CL6092.Contig2_All	2	1	1			
726	CL2379.Contig1_All	1	1	1	---	1	
726	CL2379.Contig2_All	1	1	1			
727	Unigene22519_All	1	1	1	---	1	
728	Unigene5889_All	1	1	1	---	1	
729	CL5083.Contig1_All	5	2	2	0.322	4	*
730	Unigene21066_All	1	1	1	---	1	
731	Unigene23253_All	1	1	1	---	1	
732	CL4714.Contig1_All	2	2	2	---	1	
732	CL4714.Contig2_All	2	2	2			
733	CL1081.Contig1_All	3	1	1	---		
733	Unigene32399_All	3	1	1			
734	Unigene14813_All	1	1	1	---	1	
735	Unigene3312_All	1	1	1	---	1	
736	Unigene20056_All	5	1	1	0.392	4	*
737	CL4379.Contig2_All	2	2	2	0.428	2	
738	CL5964.Contig1_All	1	1	1	---	1	
739	Unigene56251_All	8	1	1	---	1	
740	Unigene18152_All	1	1	1	---	1	
741	Unigene1708_All	1	1	1	---	1	
742	CL9537.Contig1_All	1	1	1	---	1	
743	Unigene9619_All	1	1	1	---	1	
744	Unigene11104_All	1	1	1	---	1	
745	CL5029.Contig1_All	1	1	1	---	1	
746	Unigene39_All	1	1	1	---	1	
747	CL4023.Contig3_All	1	1	1	---	1	
748	Unigene13128_All	1	1	1	---	1	
749	Unigene4585_All	1	1	1	---	1	
750	CL7215.Contig2_All	1	3	1	---		
751	Unigene17515_All	1	1	1	---	1	
752	CL7194.Contig1_All	4	1	1	---		
753	Unigene27648_All	2	2	2	---	1	
754	CL5637.Contig1_All	1	1	1	---	1	
755	CL3929.Contig1_All	2	1	1	0.932	2	
755	CL3929.Contig2_All	2	1	1			
756	CL7910.Contig1_All	1	1	1	---	1	
757	CL9795.Contig2_All	2	2	2	---	1	
758	Unigene23637_All	1	1	1	---	1	
759	Unigene7032_All	1	1	1	---	1	
760	Unigene40375_All	3	2	2	---	1	
761	Unigene7079_All	2	2	2	0.94	2	

762	Unigene24692_All	1	1	1	---	1	
763	CL581.Contig1_All	1	1	1	---	1	
763	CL581.Contig2_All	1	1	1			
763	CL581.Contig3_All	1	1	1			
763	CL581.Contig4_All	1	1	1			
764	CL7344.Contig2_All	2	1	1	---	1	
765	Unigene13058_All	2	1	1	---	1	
766	CL2100.Contig1_All	1	1	1	---	1	
767	Unigene8582_All	1	1	1	---	1	
768	CL9390.Contig1_All	3	1	1	---	1	
769	CL1955.Contig1_All	1	1	1	---	1	
769	CL1955.Contig5_All	1	1	1			
769	CL1955.Contig3_All	1	1	1			
770	CL8910.Contig1_All	2	2	2	---		
770	CL8910.Contig2_All	2	2	2			
771	Unigene22582_All	6	2	2	0.156	5	*
772	CL8595.Contig1_All	1	1	1	---	1	
773	CL3483.Contig1_All	2	1	1	---	1	
774	Unigene9647_All	1	1	1	---	1	
775	Unigene10507_All	1	1	1	---	1	
776	CL1950.Contig1_All	2	2	2	---	1	
776	CL1950.Contig3_All	2	2	2			
777	Unigene11960_All	1	1	1	---	1	
778	Unigene6998_All	2	2	2	---	1	
779	Unigene13326_All	2	1	1	---	1	
780	Unigene393_All	2	1	1	---		
781	Unigene5437_All	2	1	1	0.656	2	
782	CL3486.Contig1_All	1	1	1	---	1	
783	Unigene59990_All	1	1	1	---	1	
784	Unigene78165_All	3	1	1	---		
785	CL5675.Contig1_All	1	1	1	---	1	
786	Unigene25080_All	1	1	1	---	1	
787	Unigene781_All	1	1	1	---	1	
788	Unigene37759_All	1	1	1	---	1	
789	CL2350.Contig1_All	1	1	1	---	1	
789	Unigene67921_All	1	1	1			
790	CL8562.Contig1_All	2	1	1	---		
791	CL2677.Contig1_All	1	1	1	---	1	
792	Unigene12995_All	1	1	1	---	1	
793	Unigene21071_All	1	1	1	---	1	
794	Unigene46914_All	1	1	1	---	1	

795	Unigene32721_All	2	2	2	---	1	
796	CL5229.Contig2_All	1	1	1	---	1	
797	CL1172.Contig2_All	2	1	1	---	1	
798	Unigene9669_All	1	1	1	---	1	
799	Unigene23613_All	2	2	2	1.075	2	
800	Unigene9638_All	1	1	1	---	1	
801	Unigene9444_All	1	1	1	---	1	
802	Unigene14914_All	1	1	1	---	1	
803	Unigene17369_All	1	1	1	---	1	
804	CL2488.Contig2_All	2	1	1	0.446	2	*
804	Unigene60243_All	2	1	1			
804	CL2488.Contig4_All	2	1	1			
804	CL2751.Contig1_All	2	1	1			
805	CL8564.Contig1_All	1	1	1	---	1	
805	CL8564.Contig2_All	1	1	1			
806	Unigene70028_All	2	1	1	---		
807	CL3786.Contig1_All	1	1	1	---	1	
808	CL7250.Contig1_All	1	1	1	---	1	
808	CL7250.Contig2_All	1	1	1			
809	CL9158.Contig1_All	1	1	1	---	1	
810	Unigene17373_All	1	1	1	---	1	
811	Unigene23371_All	2	2	2	0.853	2	
812	Unigene10480_All	1	1	1	---	1	
813	CL3163.Contig1_All	2	1	1	1.578	2	
813	CL3163.Contig7_All	2	1	1			
813	CL3163.Contig2_All	2	1	1			
813	CL3163.Contig3_All	2	1	1			
813	CL3163.Contig5_All	2	1	1			
814	Unigene18453_All	3	1	1	---		
815	CL1575.Contig1_All	2	1	1	---	1	
815	CL7338.Contig1_All	2	1	1			
815	Unigene12234_All	2	1	1			
815	Unigene20847_All	2	1	1			
815	CL7338.Contig2_All	2	1	1			
816	Unigene2990_All	1	1	1	---	1	
817	CL6935.Contig1_All	1	1	1	---	1	
817	CL6935.Contig2_All	1	1	1			
818	CL5790.Contig1_All	1	1	1	---	1	
819	CL3443.Contig2_All	1	1	1	---	1	
820	CL294.Contig1_All	1	1	1	---	1	
821	Unigene13144_All	1	1	1	---	1	

822	CL8040.Contig2_All	1	1	1	---	1	
823	Unigene50705_All	3	1	1	---		
824	Unigene7045_All	1	1	1	---	1	
825	Unigene20053_All	2	1	1	---		
826	Unigene32432_All	1	1	1	---	1	
827	Unigene20889_All	1	1	1	---	1	
828	Unigene8492_All	1	1	1	---	1	
829	Unigene19982_All	1	1	1	---	1	
830	Unigene20032_All	1	1	1	---	1	
831	Unigene24002_All	1	1	1	---	1	
832	Unigene35255_All	3	1	1	---		
833	Unigene18137_All	1	1	1	---	1	
834	Unigene18051_All	1	1	1	---	1	
835	Unigene15822_All	1	1	1	---	1	
836	Unigene9701_All	2	2	2	---	1	
837	Unigene77051_All	1	1	1	---	1	
838	Unigene48458_All	1	1	1	---		
839	CL4619.Contig1_All	1	1	1	---	1	
839	CL4619.Contig2_All	1	1	1			
840	CL5183.Contig1_All	1	1	1	---	1	
841	Unigene4522_All	1	1	1	---	1	
842	Unigene4510_All	1	1	1	---	1	
843	Unigene16279_All	2	2	2	---	1	
844	Unigene9435_All	2	2	2	0.833	2	
845	Unigene77499_All	1	1	1	---	1	
846	CL8285.Contig1_All	4	1	1	2.429	3	*
846	CL8285.Contig2_All	4	1	1			
847	Unigene19849_All	1	1	1	---	1	
848	Unigene25173_All	1	1	1	---	1	
849	Unigene2768_All	1	1	1	---	1	
850	CL9741.Contig1_All	1	1	1	---	1	
850	CL9741.Contig2_All	1	1	1			
851	Unigene16015_All	4	1	1	---		
852	Unigene24036_All	1	1	1	---	1	
853	Unigene1932_All	2	2	2	---	1	
854	Unigene13259_All	1	1	1	---	1	
855	CL1200.Contig15_All	1	1	1	---	1	
855	CL1200.Contig16_All	1	1	1			
856	CL4967.Contig1_All	1	1	1	---	1	
856	CL4967.Contig2_All	1	1	1			
857	Unigene25156_All	1	1	1	---	1	

858	Unigene52299_All	1	1	1	---		
859	CL7776.Contig3_All	1	1	1	---	1	
860	Unigene55980_All	1	1	1	---	1	
861	Unigene53783_All	1	1	1	---	1	
862	Unigene7078_All	2	1	1	---	1	
863	Unigene75036_All	3	1	1	4.41	3	*
864	CL1341.Contig3_All	1	1	1	---	1	
865	Unigene59202_All	1	1	1	---	1	
866	Unigene2679_All	1	1	1	---	1	
867	Unigene17330_All	1	1	1	---	1	
868	Unigene14353_All	1	1	1	---	1	
869	Unigene7136_All	1	1	1	---	1	
870	CL1218.Contig1_All	1	1	1	---		
871	Unigene368_All	1	1	1	---		
872	Unigene12173_All	1	1	1	---	1	
873	Unigene10476_All	1	1	1	---	1	
874	Unigene2993_All	1	1	1	---	1	
875	Unigene25172_All	1	1	1	---	1	
876	Unigene19006_All	1	1	1	---	1	
877	CL8622.Contig1_All	1	1	1	---	1	
877	CL8622.Contig2_All	1	1	1			
878	Unigene52191_All	2	1	1	1.404	2	
879	CL5545.Contig2_All	4	1	1	---	1	
879	CL5545.Contig1_All	4	1	1			
880	Unigene10686_All	1	1	1	---		
881	Unigene5919_All	1	1	1	---		
882	Unigene9689_All	1	1	1	---	1	
883	Unigene22941_All	1	1	1	---	1	
884	Unigene57568_All	3	1	1	---		
885	Unigene3311_All	1	1	1	---	1	
886	CL3749.Contig1_All	1	1	1	---		
886	Unigene5140_All	1	1	1			
886	Unigene5139_All	1	1	1			
887	Unigene757_All	1	1	1	---	1	
888	CL8976.Contig1_All	1	1	1	---	1	
888	CL8976.Contig2_All	1	1	1			
889	Unigene12247_All	1	1	1	---	1	
890	Unigene9673_All	1	1	1	---		
891	Unigene63599_All	1	1	1	---	1	
892	CL2926.Contig1_All	1	1	1	---		
892	CL2926.Contig2_All	1	1	1			

893	CL3398.Contig1_All	2	1	1	0.043	2	
894	Unigene4559_All	1	1	1	---	1	
895	Unigene74748_All	1	1	1	---	1	
896	Unigene22603_All	1	1	1	---	1	
897	Unigene4553_All	1	1	1	---	1	
898	Unigene18215_All	2	2	2	---	1	
899	CL1146.Contig3_All	2	1	1	---	1	
900	CL5660.Contig2_All	2	1	1	---	1	
900	CL5660.Contig3_All	2	1	1			
901	Unigene18411_All	1	1	1	---		
902	Unigene821_All	1	1	1	---		
903	Unigene56229_All	1	1	1	---		
903	Unigene66706_All	1	1	1			
904	CL186.Contig1_All	1	1	1	---	1	
905	Unigene4457_All	1	1	1	---		
906	Unigene9688_All	1	1	1	---		
907	Unigene41842_All	2	1	1	---		
908	CL9589.Contig1_All	4	1	1	---		
908	CL9589.Contig2_All	4	1	1			
909	Unigene18858_All	1	1	1	---	1	
910	Unigene53922_All	1	1	1	---		
911	CL1117.Contig1_All	1	1	1	---	1	
911	CL1117.Contig3_All	1	1	1			
911	CL1117.Contig5_All	1	1	1			
911	CL1117.Contig7_All	1	1	1			
912	CL8904.Contig1_All	3	1	1	2.665	3	*
913	Unigene13007_All	2	1	1	0.436	2	*
914	Unigene17404_All	2	1	1	---		
915	Unigene21521_All	1	1	1	---		
916	Unigene40576_All	1	1	1	---		
917	CL8201.Contig2_All	1	1	1	---		
918	Unigene75597_All	3	1	1	---		
919	Unigene68447_All	1	1	1	---	1	
920	Unigene10504_All	1	1	1	---		
921	Unigene23392_All	1	1	1	---		
922	CL2671.Contig1_All	1	2	1	---		
923	Unigene41309_All	1	1	1	---		
924	CL328.Contig1_All	1	1	1	---		
924	CL328.Contig3_All	1	1	1			
925	Unigene125_All	1	1	1	---	1	
925	Unigene76858_All	1	1	1			

926	CL6186.Contig1_All	1	1	1	---		
927	CL8786.Contig1_All	2	1	1	---		
927	CL8786.Contig2_All	2	1	1			
928	Unigene14837_All	1	1	1	---		
929	Unigene7829_All	1	1	1	---		
930	CL2622.Contig1_All	1	1	1	---	1	
931	Unigene3373_All	1	1	1	---	1	
932	CL5382.Contig1_All	2	2	2	---		
933	Unigene33837_All	1	1	1	---		
934	Unigene17384_All	1	1	1	---		
935	Unigene7116_All	1	1	1	---		
936	Unigene1977_All	1	1	1	---		
937	CL4390.Contig1_All	1	1	1	---		
938	Unigene18380_All	1	1	1	---		
939	CL9289.Contig1_All	2	2	2	---		
939	CL9289.Contig2_All	2	2	2			
940	CL7424.Contig2_All	1	1	1	---	1	
941	CL9381.Contig1_All	1	1	1	---		
941	CL9381.Contig2_All	1	1	1			
942	Unigene18118_All	1	1	1	---		
942	Unigene57918_All	1	1	1			
943	CL748.Contig1_All	1	1	1	---		
943	Unigene50454_All	1	1	1			
944	Unigene17391_All	1	1	1	---		
945	Unigene65139_All	1	1	1	---		
946	Unigene54328_All	6	1	1	0.434	2	*
947	Unigene14845_All	1	1	1	---		
948	Unigene56203_All	1	1	1	---		
949	Unigene18112_All	2	1	1	---		
950	Unigene9766_All	1	1	1	---		
951	Unigene12195_All	1	1	1	---		
952	Unigene25623_All	1	1	1	---		
953	Unigene802_All	1	1	1	---	1	
954	CL10003.Contig2_All	1	1	1	---		
955	CL1092.Contig1_All	1	1	1	---		
956	Unigene5390_All	1	1	1	---		
957	CL3329.Contig1_All	1	1	1	---		
958	Unigene25111_All	1	1	1	---		
959	Unigene3570_All	1	1	1	---		
960	Unigene9648_All	1	1	1	---		
961	CL6395.Contig1_All	1	1	1	---		

962	Unigene132_All	1	1	1	---		
963	Unigene9785_All	1	1	1	---		
964	Unigene12180_All	1	1	1	---		
965	Unigene18512_All	3	1	1	---		
966	Unigene22596_All	1	1	1	---		
967	Unigene17507_All	2	2	2	---		
968	CL1529.Contig1_All	1	1	1	---	1	
968	CL1529.Contig2_All	1	1	1			
969	CL9515.Contig1_All	2	1	1	---		
970	CL937.Contig1_All	4	1	1	---		
970	CL937.Contig3_All	4	1	1			
971	Unigene47476_All	3	1	1	---		
972	Unigene21149_All	4	1	1	---		
973	Unigene67316_All	1	1	1	---		
974	Unigene22304_All	1	1	1	---		
975	Unigene18299_All	4	1	1	3.154	4	*
976	Unigene2912_All	1	1	1	---		
977	Unigene4445_All	1	1	1	---		
978	CL4137.Contig1_All	1	1	1	---		
979	CL10007.Contig1_All	1	1	1	---		
980	Unigene48460_All	1	1	1	---		
981	CL8390.Contig1_All	1	1	1	---		
981	CL8390.Contig2_All	1	1	1			
982	CL4950.Contig1_All	1	1	1	---		
983	Unigene2727_All	1	1	1	---		
984	Unigene844_All	1	1	1	---		
985	CL391.Contig1_All	1	1	1	---		
985	Unigene17438_All	1	1	1			
985	CL391.Contig2_All	1	1	1			
986	Unigene44581_All	1	1	1	---		
987	Unigene64946_All	1	1	1	---		
988	Unigene6339_All	2	1	1	---		
989	Unigene12355_All	1	1	1	---	1	
990	Unigene66371_All	2	1	1	---		
991	Unigene73408_All	2	1	1	---		
992	CL3641.Contig3_All	1	1	1	---		
993	Unigene4774_All	1	1	1	---		
994	Unigene2780_All	3	1	1	---		
995	Unigene65093_All	1	1	1	---		
996	Unigene20732_All	2	1	1	---		
997	CL7298.Contig2_All	2	1	1	---		

998	Unigene9636_All	1	1	1	---		
999	CL8272.Contig1_All	1	1	1	---		
1000	CL2679.Contig2_All	1	1	1	---		
1000	Unigene41080_All	1	1	1			
1000	CL2679.Contig4_All	1	1	1			
1000	CL2679.Contig3_All	1	1	1			
1001	Unigene23339_All	1	1	1	---		
1002	CL7333.Contig1_All	1	1	1	---		
1003	Unigene25147_All	1	1	1	---		
1004	CL8946.Contig1_All	1	1	1	---		
1004	Unigene78203_All	1	1	1			
1005	Unigene9394_All	2	1	1	---		
1006	Unigene72730_All	2	1	1	---		
1007	Unigene17487_All	1	1	1	---		
1008	Unigene1044_All	1	1	1	---	1	
1009	Unigene24911_All	1	1	1	---		
1010	Unigene68624_All	1	1	1	---		
1011	CL2732.Contig1_All	1	1	1	---		
1011	CL2732.Contig2_All	1	1	1			
1011	CL2732.Contig4_All	1	1	1			
1011	CL2732.Contig3_All	1	1	1			
1011	CL2732.Contig5_All	1	1	1			
1012	Unigene56640_All	1	1	1	---		
1013	Unigene18429_All	1	1	1	---		
1014	Unigene23375_All	1	1	1	---		
1015	Unigene41752_All	2	1	1	---		
1016	Unigene12326_All	1	1	1	---		
1017	Unigene11133_All	1	1	1	---		
1018	Unigene13221_All	2	2	2	---		
1019	CL1001.Contig3_All	1	1	1	---		
1020	CL5685.Contig1_All	1	1	1	---	1	
1021	Unigene7402_All	1	1	1	---		
1022	Unigene17392_All	1	1	1	---		
1023	Unigene8506_All	1	1	1	---		
1024	CL8672.Contig1_All	1	1	1	---		
1025	Unigene45935_All	1	1	1	---		
1026	CL7377.Contig1_All	1	1	1	---		
1027	Unigene70627_All	2	1	1	---		
1028	Unigene63840_All	1	1	1	---		
1029	CL3969.Contig1_All	1	1	1	---	1	
1030	Unigene22078_All	1	1	1	---		

1031	CL2608.Contig2_All	1	1	1	---		
1031	CL2608.Contig7_All	1	1	1			
1031	CL2608.Contig4_All	1	1	1			
1031	Unigene15550_All	1	1	1			
1032	CL8304.Contig1_All	1	1	1	---		
1033	CL6143.Contig1_All	1	1	1	---		
1033	CL6143.Contig2_All	1	1	1			
1034	Unigene23620_All	1	1	1	---		
1035	CL8616.Contig1_All	1	1	1	---		
1036	Unigene11154_All	1	1	1	---		
1037	Unigene17380_All	1	1	1	---		
1038	CL9642.Contig1_All	1	1	1	---		
1038	CL9642.Contig2_All	1	1	1			
1039	Unigene45665_All	1	1	1	---		
1040	Unigene46628_All	1	1	1	---	1	
1041	Unigene18463_All	1	1	1	---		
1042	Unigene10472_All	1	1	1	---		
1043	Unigene47423_All	1	1	1	---		
1044	CL2022.Contig2_All	1	1	1	---		
1045	Unigene25215_All	1	1	1	---		
1046	Unigene70_All	1	1	1	---		
1047	CL8899.Contig1_All	1	1	1	---		
1047	CL8899.Contig2_All	1	1	1			
1048	Unigene34296_All	1	1	1	---		
1049	Unigene18401_All	3	1	1	---		
1050	Unigene39646_All	1	1	1	---		
1051	CL1024.Contig1_All	1	1	1	---		
1051	CL1024.Contig2_All	1	1	1			
1052	Unigene7810_All	1	1	1	---		
1053	Unigene69484_All	1	1	1	---		
1054	Unigene62_All	1	1	1	---	1	
1055	Unigene2977_All	1	1	1	---		
1055	CL7369.Contig1_All	1	1	1			
1056	CL3386.Contig1_All	1	1	1	---		
1056	CL3386.Contig2_All	1	1	1			
1057	Unigene1906_All	1	1	1	---		
1058	Unigene11052_All	1	1	1	---		
1059	Unigene7727_All	1	1	1	---		
1060	Unigene23489_All	1	1	1	---		
1061	Unigene42178_All	1	1	1	---		
1062	Unigene23917_All	1	1	1	---		

1063	Unigene51940_All	1	1	1	---		
1063	Unigene51941_All	1	1	1			
1064	CL4421.Contig1_All	1	1	1	---		
1065	Unigene14532_All	1	1	1	---		
1066	Unigene10543_All	1	1	1	---	1	
1067	CL439.Contig1_All	1	1	1	---		
1067	CL439.Contig2_All	1	1	1			
1068	CL4398.Contig1_All	1	1	1	---		
1069	CL1687.Contig1_All	1	1	1	---		
1070	Unigene79344_All	1	1	1	---		
1071	Unigene13739_All	1	1	1	---		
1072	CL967.Contig1_All	1	1	1	---		
1073	Unigene23624_All	1	1	1	---		
1074	Unigene62379_All	1	1	1	---		
1075	Unigene53781_All	1	1	1	---		
1076	Unigene18052_All	1	1	1	---		
1077	Unigene54963_All	1	1	1	---	1	
1078	Unigene61470_All	1	1	1	---		

Table S5 Differentially expressed peptides and their corresponding mRNAs in response to diapause.

		transcriptome								
		-10~-	-5~-10	-2~-5	-1~-2	1~2	2~5	5~10	10~+	total
itraq	3+	1	0	2	0	1	2	1	0	7
	2~3	0	0	3	2	3	5	4	0	17
	1.2~2	0	1	3	3	7	6	2	1	23
	-1.2~-2	0	2	3	9	19	10	4	4	51
	-2~-3	1	3	2	4	3	3	5	2	23
	-3~-	0	3	3	1	2	3	1	0	13
	total	2	9	16	19	35	29	17	7	134

Table S6 Correlation between mRNAs and proteins with same trend.					
Protein	Sig	diff_Protein	log2(D/N)	Fdr	diff_gene
CL426.Contig1_All	*	-	-1.03469	6.05E-68	-
Unigene16201_All	*	-	-1.19366	0	-
CL9226.Contig1_All	*	-	-2.30287	9.53E-134	-
Unigene25176_All	*	+	1.950881	0	+
CL2093.Contig3_All	*	-	-6.55504	0	-
Unigene10351_All	*	+	1.01211	0	+
Unigene21458_All	*	+	5.846586	0	+
Unigene18931_All	*	-	-3.7348	0	-
CL8904.Contig1_All	*	+	7.242743	8.25E-266	+
CL2165.Contig1_All	*	-	-8.2323	0	-
Unigene18299_All	*	+	1.806612	1.32E-296	+
Unigene4410_All	*	-	-1.10899	8.23E-177	-
CL2165.Contig3_All	*	-	-6.92913	0	-
Unigene5103_All	*	+	2.354244	8.00E-41	+
Unigene25985_All	*	-	-10.214	0	-
Unigene4448_All	*	-	-1.25744	0	-
CL1845.Contig2_All	*	+	1.758298	0	+
Unigene5485_All	*	+	2.953686	0	+
CL8832.Contig1_All	*	-	-7.85827	0	-
Unigene15870_All	*	+	4.0626	0	+
Unigene23376_All	*	+	1.992083	6.28E-260	+
Unigene53826_All	*	+	4.619027	1.22E-30	+
Unigene11986_All	*	-	-1.11934	0	-
CL929.Contig1_All	*	-	-1.14951	2.91E-266	-
CL2947.Contig1_All	*	+	3.116328	2.95E-55	+
Unigene25254_All	*	+	1.85856	0	+
Unigene22597_All	*	+	1.076161	0	+
Unigene51240_All	*	+	2.237466	2.75E-09	+
Unigene4416_All	*	-	-3.57647	0	-
CL9597.Contig1_All	*	-	-1.447	4.43E-18	-
CL1988.Contig2_All	*	+	6.904746	0	+
CL2826.Contig2_All	*	+	2.770843	6.72E-77	+
CL5772.Contig1_All	*	+	6.861945	2.13E-67	+
CL1425.Contig1_All	*	+	2.0413	1.67E-21	+
CL3110.Contig1_All	*	-	-1.16011	2.04E-187	-
Unigene22800_All	*	-	-2.39654	0	-
Unigene12989_All	*	+	1.135465	5.24E-50	+
CL90.Contig1_All	*	-	-2.5504	0	-
CL2165.Contig5_All	*	-	-9.7988	0	-
Unigene12208_All	*	-	-2.63515	0	-
CL1897.Contig3_All	*	+	11.61333	4.51E-15	+

Unigene15993_All	*	-	-5.89573	0	-
Unigene6729_All	*	-	-6.57174	0	-
Unigene9762_All	*	+	3.609198	0	+
CL3314.Contig2_All	*	-	-8.61154	0	-
Unigene4478_All	*	+	5.624423	0	+
CL5476.Contig1_All	*	+	3.200827	6.67E-250	+
CL5757.Contig1_All	*	-	-1.45295	2.78E-102	-
Unigene14775_All	*	-	-1.26641	0	-
Unigene7159_All	*	+	2.725123	0	+
Unigene43565_All	*	+	5.26735	9.17E-11	+
Unigene15598_All	*	+	1.248107	1.96E-30	+
Unigene6800_All	*	+	3.880615	0	+
Unigene5800_All	*	+	1.964442	0	+
CL9361.Contig1_All	*	+	3.469684	0	+
Unigene56003_All	*	-	-2.44702	9.04E-14	-
CL179.Contig1_All	*	-	-2.03904	0	-

Table S7 Correlation between mRNAs and proteins with opposite trend.

Protein	Sig	diff_Protein	log2(D/N)	Fdr	diff_gene
Unigene19952_All	*	-	1.498071	0	+
Unigene23762_All	*	+	-2.232	3.25E-15	-
Unigene750_All	*	+	-1.85643	1.96E-52	-
Unigene14913_All	*	-	1.187217	1.80E-300	+
CL1543.Contig1_All	*	-	7.592809	6.31E-228	+
Unigene4463_All	*	+	-2.61628	0	-
CL6507.Contig1_All	*	-	2.132329	2.56E-285	+
Unigene72_All	*	-	1.329067	7.85E-154	+
Unigene17372_All	*	-	1.089984	0	+
Unigene54462_All	*	-	4.090367	1.04E-73	+
Unigene76_All	*	+	-1.12416	6.04E-77	-
Unigene23627_All	*	-	1.223036	0	+
CL7121.Contig1_All	*	-	1.499937	0	+
Unigene10471_All	*	+	-2.23852	2.17E-61	-
Unigene22582_All	*	-	1.105436	1.54E-131	+
Unigene16304_All	*	-	1.145481	0	+
CL1728.Contig1_All	*	-	12.57594	1.07E-148	+
Unigene13138_All	*	-	1.636462	1.36E-140	+
CL1161.Contig1_All	*	-	6.6594	5.52E-116	+
CL4299.Contig1_All	*	-	5.176037	2.70E-66	+
Unigene38965_All	*	+	-3.21219	0.000300664	-
Unigene57126_All	*	-	3.714204	9.50E-45	+
CL4537.Contig2_All	*	-	1.783301	0	+
CL6.Contig1_All	*	+	-2.9511	4.54E-28	-

Unigene5336_All	*	-	1.816834	0	+
Unigene22928_All	*	+	-1.58843	2.02E-08	-
Unigene29395_All	*	+	-2.90275	2.98E-09	-
CL9117.Contig3_All	*	-	4.407063	0	+
CL5100.Contig1_All	*	-	11.32531	1.90E-31	+
Unigene12194_All	*	-	4.388627	0	+
Unigene344_All	*	-	1.058267	1.46E-140	+
CL246.Contig1_All	*	-	1.28848	0	+
Unigene61997_All	*	-	10.89004	1.89E-16	+
CL2488.Contig2_All	*	-	6.626593	0	+
CL1759.Contig2_All	*	-	11.34668	5.30E-17	+
Unigene10672_All	*	+	-1.22934	6.38E-90	-
CL2415.Contig1_All	*	-	1.953562	0	+
CL5772.Contig3_All	*	-	1.126666	0	+
CL329.Contig1_All	*	-	1.051912	0	+
CL3076.Contig1_All	*	-	3.00752	4.80E-37	+
CL1485.Contig1_All	*	-	1.459939	2.25E-05	+
CL5398.Contig2_All	*	-	3.133699	1.82E-300	+
Unigene22611_All	*	-	2.160838	0	+
Unigene19998_All	*	-	1.195261	0	+
Unigene23329_All	*	-	1.416074	8.14E-206	+
Unigene20610_All	*	+	-6.78641	0	-
Unigene8061_All	*	-	1.187448	3.62E-230	+
Unigene5797_All	*	-	6.625439	0	+
Unigene39054_All	*	+	-11.9253	9.22E-12	-
Unigene20056_All	*	-	3.377324	0	+
CL5083.Contig1_All	*	-	2.559428	0	+
CL5638.Contig3_All	*	-	1.064804	2.53E-121	+
Unigene54328_All	*	-	2.585468	1.40E-127	+
Unigene25192_All	*	+	-1.2046	0	-
Unigene25234_All	*	-	3.622014	0	+
CL8649.Contig1_All	*	-	3.024677	0	+
CL620.Contig1_All	*	-	7.227863	0	+
Unigene23412_All	*	-	1.229576	0	+
Unigene36258_All	*	+	-3.18399	2.21E-10	-
